# Pyrazole-based lamellarin O analogues: synthesis, biological evaluation and structure–activity relationships†

**DOI:** 10.1039/d3ra00972f

**Published:** 2023-03-10

**Authors:** Karolina Dzedulionytė, Nina Fuxreiter, Ekaterina Schreiber-Brynzak, Asta Žukauskaitė, Algirdas Šačkus, Verena Pichler, Eglė Arbačiauskienė

**Affiliations:** a Department of Organic Chemistry, Faculty of Chemical Technology, Kaunas University of Technology Radvilėnų pl. 19 LT-50254 Kaunas Lithuania egle.arbaciauskiene@ktu.lt; b Department of Pharmaceutical Sciences, Division of Pharmaceutical Chemistry, Faculty of Life Sciences, University of Vienna Althanstraße 14 1090 Vienna Austria verena.pichler@univie.ac.at; c Department of Chemical Biology, Faculty of Science, Palacký University Šlechtitelů 27 CZ-78371 Olomouc Czech Republic; d Institute of Synthetic Chemistry, Faculty of Chemical Technology, Kaunas University of Technology K. Baršausko g. 59 LT-51423 Kaunas Lithuania

## Abstract

A library of pyrazole-based lamellarin O analogues was synthesized from easily accessible 3(5)-aryl-1*H*-pyrazole-5(3)-carboxylates which were subsequently modified by bromination, *N*-alkylation and Pd-catalysed Suzuki cross-coupling reactions. Synthesized ethyl and methyl 3,4-diaryl-1-(2-aryl-2-oxoethyl)-1*H*-pyrazole-5-carboxylates were evaluated for their physicochemical property profiles and *in vitro* cytotoxicity against three human colorectal cancer cell lines HCT116, HT29, and SW480. The most active compounds inhibited cell proliferation in a low micromolar range. Selected ethyl 3,4-diaryl-1-(2-aryl-2-oxoethyl)-1*H*-pyrazole-5-carboxylates were further investigated for their mode of action. Results of combined viability staining *via* Calcein AM/Hoechst/PI and fluorescence-activated cell sorting data indicated that cell death was triggered in a non-necrotic manner mediated by mainly G2/M-phase arrest.

## Abstract 2

Isosteric pyrrole–pyrazole exchange in the natural alkaloid lamellarin O resulted in 18 fully characterized derivatives. Obtained compounds were investigated as potent agents against human colon cancer cell lines HCT116, HT29 and SW480.

## Graphical abstract

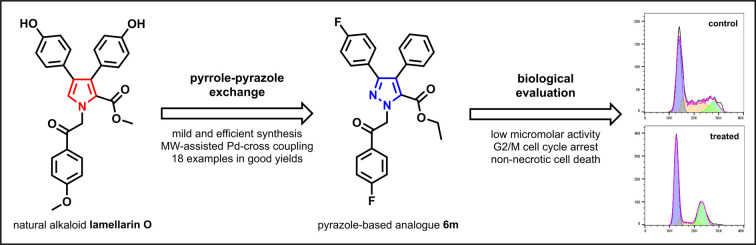

## Introduction

**Fig. 1:**
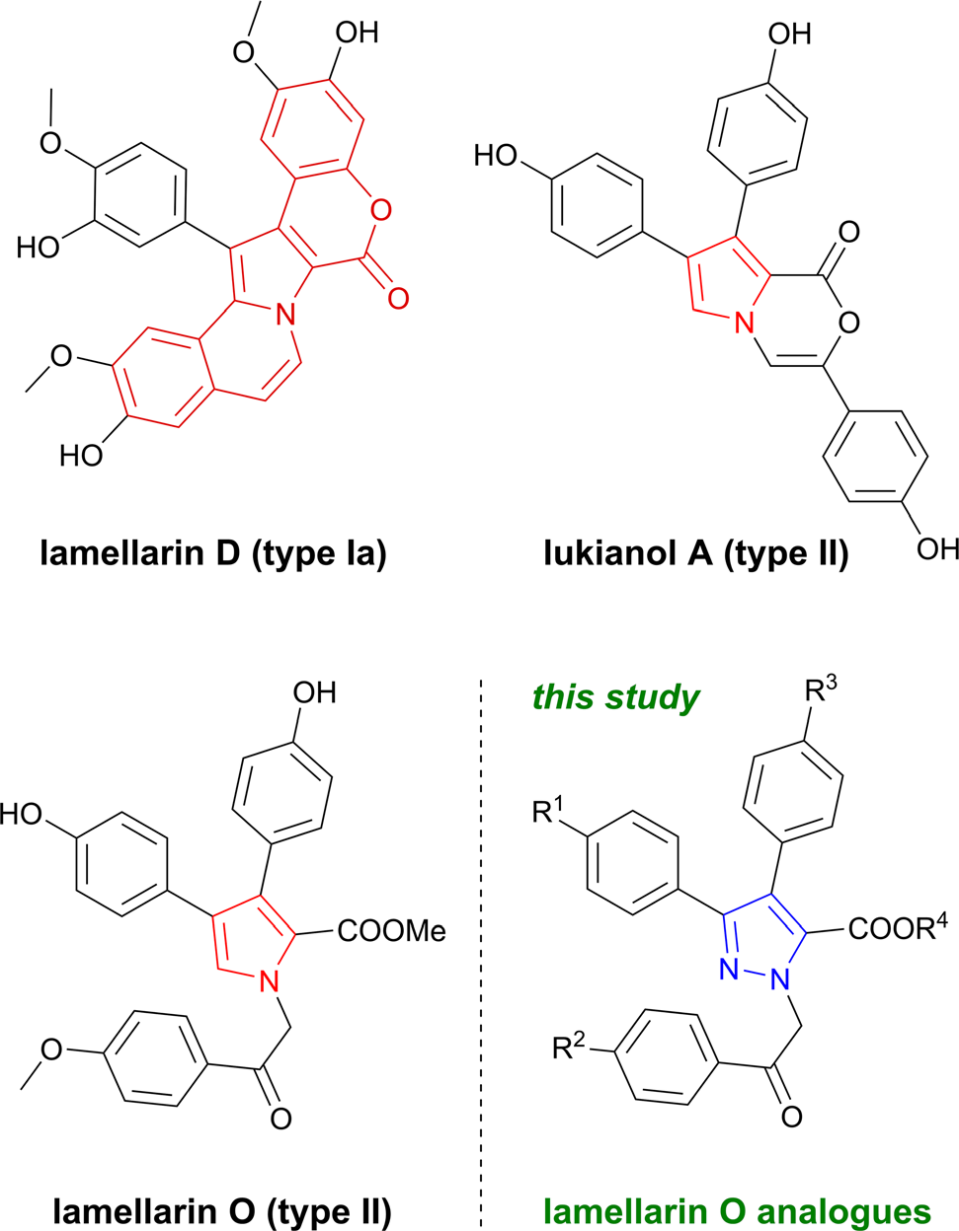
Relevant lamellarin alkaloid representatives of type I and type II bearing a characteristic central pyrrole core and novel pyrazole containing analogues.

Lamellarins are a group of natural marine-derived alkaloids with a characteristic central pyrrole moiety and widely reported biological activity. Since their discovery in 1985, more than 50 compounds of this family have been isolated from various marine organisms, mainly, but not exclusively, sponges and ascidians.^1–3^ Most lamellarins contain 6*H*[1]benzo-pyrano[4′,3′:4,5]pyrrolo[2,1-*a*]isoquinolinone chromophore and fall into two subtypes – 5,6-saturated and 5,6-unsaturated lamellarins, designated as type Ia and type Ib, respectively. Structurally less complex type II lamellarins form a smaller class of compounds bearing 3,4-diarylpyrrole-2-carboxylate fragment (Fig. 1).^4,5^

The biological spectrum of lamellarins is manifold, with pronounced cytotoxic activity.^6–11^ In detail, lamellarin D (Fig. 1), a leading compound in the family of type I, induces its anticancer activity through topoisomerase I inhibition and mitochondrial targeting that triggers cell death.^12–15^ Lamellarin O was investigated by Huang *et al.*^16^ As reported, cytotoxicity effects of some natural lamellarins were assessed towards colorectal cancer SW620 and its multi-drug resistant daughter cell line SW620/Ad300. Lamellarin O exhibited moderate cytotoxicity against aforementioned cells at IC_50_ of 20.0 μM and 22.3 μM respectively. Whereas lukianol A, a natural cyclized lactone of lamellarin O, is known to exhibit cytotoxicity against human epidermoid carcinoma cell lines.^17^ Other lamellarins can reverse multidrug resistance and consequently promote therapeutic activity of conventional cytotoxic drugs towards chemoresistant tumours.^5,18^

The unbridled key goal is, besides appropriate strategies to synthetically approach these natural compounds,^17,19–21^ to create new analogues as innovative drug-like compounds with potent antitumor activities.^22–24^ Zheng *et al.* reported synthesis and investigation of novel glycosylated lamellarin D compounds wherein glycosyl moieties improve important physicochemical properties of active compounds, especially the solubility in water.^25^ Another study revealed A-ring modified lamellarin N analogues as potent noncovalent inhibitors of EGFR T790M/L858R mutant, which is responsible for non-small cell lung cancer resistance.^26^ More recent investigation on A-ring modified azalamellarins revealed that synthetic analogues selectively inhibit the proliferation of EGFR T790M/L858R mutant cells over EGFR WT cells.^27^ Moreover, Klumthong *et al.* presented a diversity-oriented synthesis of azalamellarins, where lactone-to-lactam modification resulted in increased cytotoxicity against HeLa cervical cancer cells.^28^

Following these significant discoveries, interest in lamellarin based research has been growing and remains highly relevant. The structure of lamellarin O is easily accessible for replacement of the central pyrrole ring to design new derivatives with structural similarities like shape and electronic configuration by other five membered ring systems. Pyrazole is a versatile moiety taking place in various biologically active compounds as well as in-use pharmaceuticals.^29–31^ Replacement of the central pyrrole to pyrazole is expected to change *e.g.*, the energy of the highest occupied molecular orbital (HOMO) which is associated with increased metabolic stability.^32^

In continuation of our previous works devoted to synthesis and investigation of pyrazole derivatives,^33–41^ in this study we ought to synthesize and investigate various functionalized pyrazole derivatives of lamellarin O. The goal was based on the scaffold hopping of the pyrrole ring in natural lamellarin O to its pyrazole counterpart. Synthetic strategy involves 3,5-substituted pyrazole formation and pyrazole functionalization at 4-position by Pd-catalysed Suzuki cross-coupling reaction. Obtained compounds were evaluated for their physicochemical properties and further investigated as potent agents against human colon cancer cell lines HCT116, HT29 and SW480. Moreover, after structure–activity relationship determination, the most cytotoxic compounds were used to investigate their mode of action in the before mentioned cell lines.

## Results and discussion

### Chemistry

**Scheme 1:**
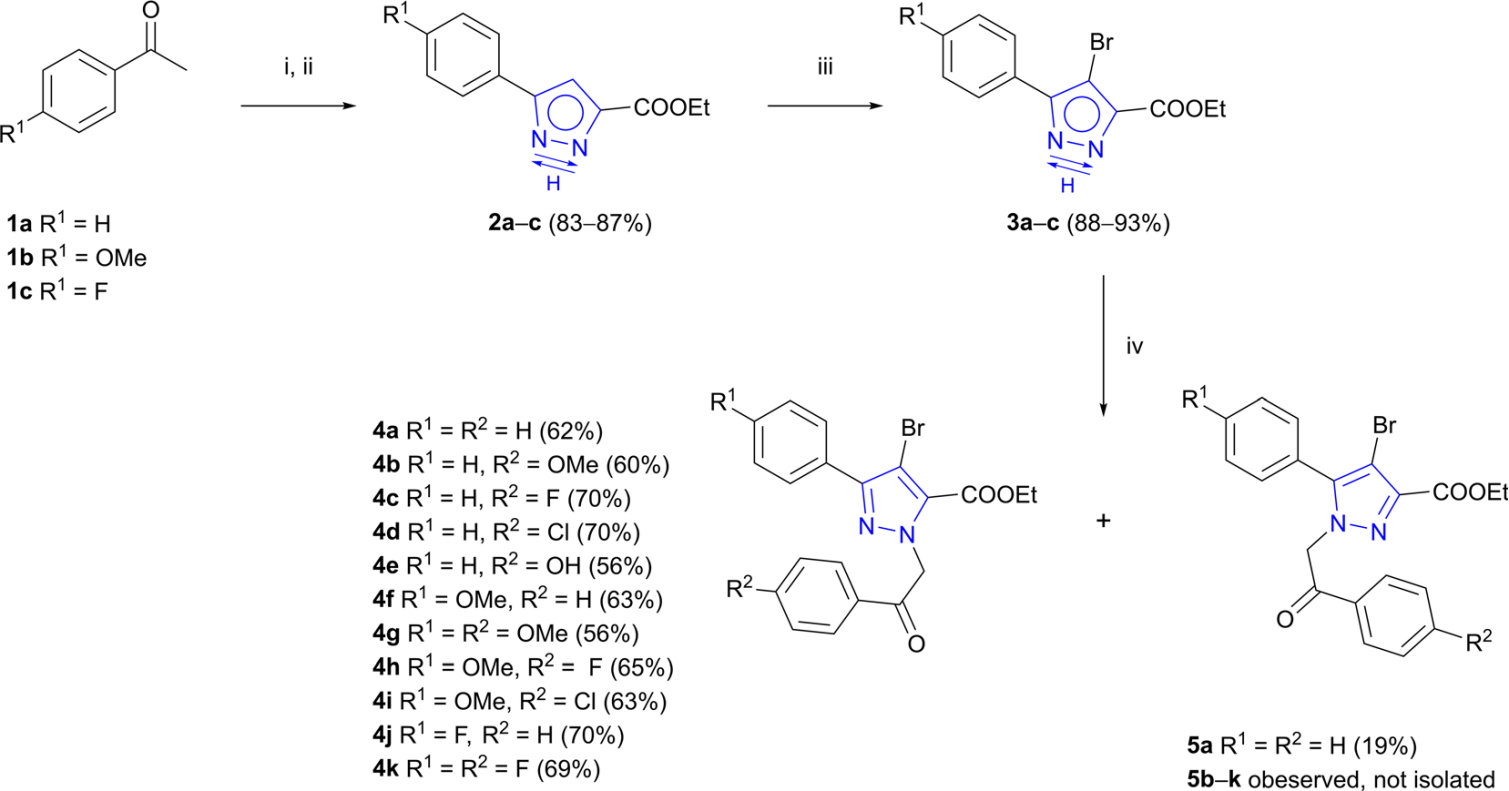
Synthesis of intermediates 4a–k. Reagents and conditions: (i) diethyl oxalate, NaOEt, EtOH, rt, 16 h;^42^ (ii) NH_2_NH_2_·H_2_O, AcOH, EtOH, rt, 16 h;^42^ (iii) NBS, DCM, 35 °C, 16 h;^43^ (iv) appropriate 2-bromoacetophenone, Na_2_CO_3_, DMF, 60–70 °C, 5–8 h.

As outlined in Scheme 1, Claisen condensation of commercially available acetophenones 1a–c with diethyl oxalate and subsequent cyclocondensation with hydrazine hydrate afforded 3(5)-aryl-1*H*-pyrazole-5(3)-carboxylates 2a–c in good yields as previously described by Wu *et al.*^42^

For introduction of phenyl substituents at C-4 of pyrazole ring, a halogen atom at the indicated position was introduced beforehand. 3(5)-Aryl-1*H*-pyrazole-5(3)-carboxylates 2a–c underwent bromination reaction using NBS in DCM and products 3a–c were obtained in 88–93% yields.^43^ Thereafter, two alternative pathways could be employed – either by first forming C–C bond *via* cross-coupling reaction and subsequently conducting *N*-alkylation, or *vice versa*. Multiple experiments were carried out towards Suzuki cross-coupling with pyrazole 3a bearing free –NH group (see ESI, Table S1†). Unfortunately, most C–C bond formation reactions resulted in complex mixtures and product yield did not exceed 22%. To tackle this problem, functionalization of free –NH group had to be performed in the first place.^44^

It is known that NH-pyrazoles usually exhibit annular *N*,*N*-prototropy.^45^ Typically, *N*-alkylation of asymmetrically ring-substituted 1*H*-pyrazoles results in the formation of a mixture of regioisomeric *N*-substituted products,^46–49^ therefore regioselective *N*-alkylation requires optimisation of reaction conditions. In this work, the goal was to carry out alkylation of 3(5)-aryl-4-bromo-1*H*-pyrazole-5(3)-carboxylates 3a–c in a regioselective manner to obtain desired isomers 4a–k as major products. Experiments were carried out with 3(5)-aryl-1*H*-pyrazole-5(3)-carboxylate 3a and the influence of the solvent and/or base on the regiochemical outcome of the reaction was evaluated. Comparison of Na_2_CO_3_, K_2_CO_3_ and NaH bases using DMF or ACN as a solvent revealed that combination of Na_2_CO_3_ and less polar DMF gave the best regioselectivity ratio of isomers 4a and 5a. Therefore, reaction conditions with Na_2_CO_3_-DMF system were applied for the synthesis of intermediates 4a–k.

**Fig. 2:**
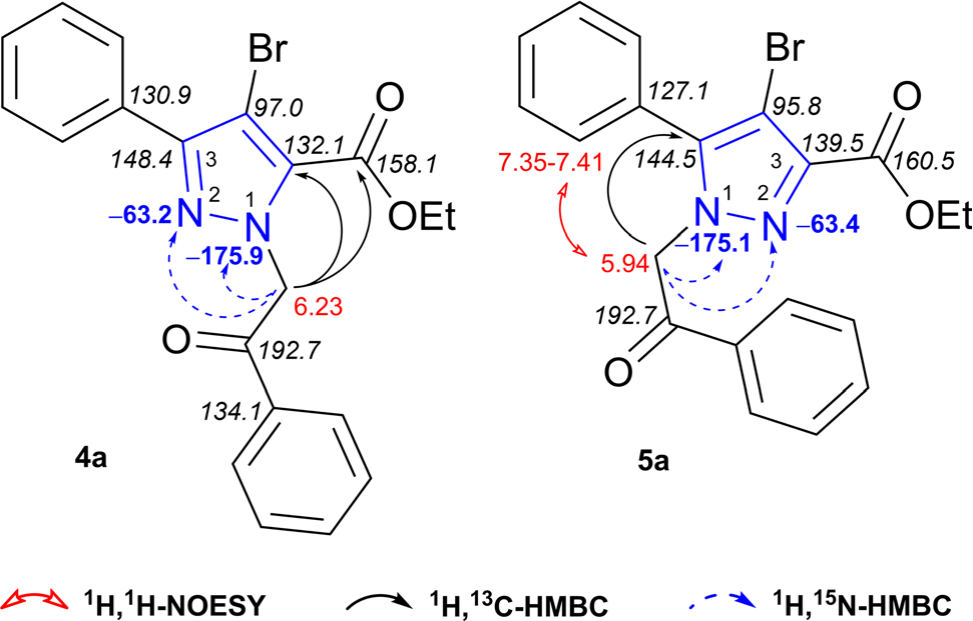
^1^H (red), ^13^C (italic), ^15^N (blue) NMR chemical shifts and relevant ^1^H, ^13^C-HMBC, ^1^H, ^15^N-HMBC, ^1^H, ^1^H-NOESY correlations of regioisomers 4a and 5a.

*N*-Alkylated products 4a and 5a were fully characterized based on ^1^H, ^1^H-COSY, ^1^H, ^1^H-NOESY, ^1^H, ^13^C-HSQC, ^1^H, ^13^C- and ^1^H, ^15^N-HMBC experimental data (Fig. 2). To assign the regiochemistry of isomers, ^1^H, ^13^C-HMBC experiment was fundamental. Obtained data of regioisomer 4a revealed strong heteronuclear three-bond correlation between NCH_2_ protons at *δ* 6.23 ppm and annular C-5 of pyrazole at *δ* 132.1 ppm. Another correlation for distinguishing regioisomers is a long-range coupling between the same NCH_2_ protons at *δ* 6.23 ppm and carboxylate ester carbon at *δ* 158.1 ppm. Minor isomer 5a can be easily identified in a similar manner. A strong three-bond coupling was observed between NCH_2_ protons at *δ* 5.94 ppm and pyrazole C-5 at *δ* 144.5 ppm. Distinct NOE was observed between NCH_2_ protons at *δ* 5.94 and 5-phenyl ring 2(6)-H protons at *δ* 7.35–7.41 ppm, which confirms their proximity in space. The absence of heteronuclear correlation between NCH_2_ protons and carbon atom from carboxylate ester indicates the structure of 5-phenyl-1*H*-pyrazole-3-carboxylate 5a.

Synthesized 3-aryl-4-bromo-1*H*-pyrazole-5-carboxylate in-termediates 4a–k were used for the final derivatization step *i.e.*, construction of C–C bond *via* Pd-catalysed Suzuki cross-coupling reaction. As the efficiency and yield of transition metal catalysed reactions are influenced by various factors such as catalysts, solvents, bases, ligands, or other additives,^50–53^ optimization was carried out beforehand using carboxylate 4a as a model compound (Table 1). For the C–C coupling Pd(PPh_3_)_4_ was employed as a catalyst. Investigation started with the use of K_3_PO_4_ in DMF and water as a co-solvent for the dissolution of inorganic base. In the initial attempt, reaction mixture was stirred at 100 °C for 16 h affording hydrolysed product 1-(2-oxo-2-phenylethyl)-3,4-diphenyl-1*H*-pyrazole-5-carboxylic acid (6a′) in 53% yield (Table 1, entry 1). As reaction with K_3_PO_4_ in DMF/H_2_O resulted in successful C–C bond formation, it was repeated using MW-assisted heating (Table 1, entry 2). In the latter experiment hydrolysed product was once again isolated as a sole reaction product, however, the outcome of the reaction has improved and reached 77% yield.

**Table 1:** Optimization of Suzuki cross-coupling reaction conditions using 4a as a model compound

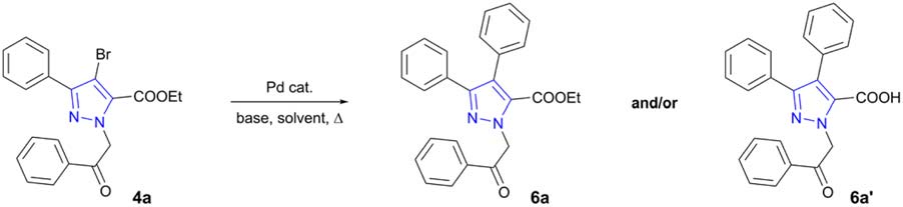								
Entry	Catalyst	Base	Solvent	Heating	Temperature (°C)	Time (h)	Yield^a^ (%)	
							6a	6a′ b
1	Pd(PPh_3_)_4_	K_3_PO_4_	DMF/H_2_O	Conventional	100	16	—	53
2	Pd(PPh_3_)_4_	K_3_PO_4_	DMF/H_2_O	MW-assisted	140	1	—	77
3	Pd(PPh_3_)_4_	K_3_PO_4_	Dioxane/H_2_O	Conventional	100	24	51	38
4	Pd(PPh_3_)_4_	K_3_PO_4_	Dioxane/H_2_O	MW-assisted	100	1	78	—
5	Pd(PPh_3_)_4_	Na_2_CO_3_ (sat.)	Toluene/EtOH	Conventional	80	24	16	—
6	Pd(PPh_3_)_4_	Cs_2_CO_3_	DMF/H_2_O	MW-assisted	140	1	—	73
7	Pd(PPh_3_)_4_	Cs_2_CO_3_	Dioxane/H_2_O	MW-assisted	100	1	84	—
8	Pd(OAc)_2_	Cs_2_CO_3_	Dioxane/H_2_O	MW-assisted	100	1	50	—

a Isolated yield.

b Hydrolysed product.

Further investigations involved multiple experiments using K_3_PO_4_ in less polar dioxane or dioxane/water solvent systems. Using conventional heating (Table 1, entry 3) both desired carboxylate ester 6a and hydrolysed product 6a′ were isolated in 51% and 38% yields, respectively. To our satisfaction, reaction with K_3_PO_4_ in dioxane/water system under MW irradiation proceeded without the undesired hydrolysis, giving rise to 6a in 78% yield (Table 1, entry 4). Additional experiment using saturated Na_2_CO_3_ solution in toluene/EtOH mixture^54^ was carried out giving very low 16% yield of 6a (Table 1, entry 5). Few more reactions were investigated using Cs_2_CO_3_ as a base (Table 1, entries 6 and 7) and Pd(OAc)_2_ as a catalyst (Table 1, entry 8), revealing Pd(PPh_3_)_4_ and Cs_2_CO_3_–dioxane–H_2_O system as the most suitable approach for C–C bond formation. In contrast to a cross-coupling under conventional heating, MW-assisted heating dramatically shortened reaction times, formed relatively pure products, and increased overall yields. Therefore, MW irradiation took a significant role in successful formation of the target compounds.

**Scheme 2:**
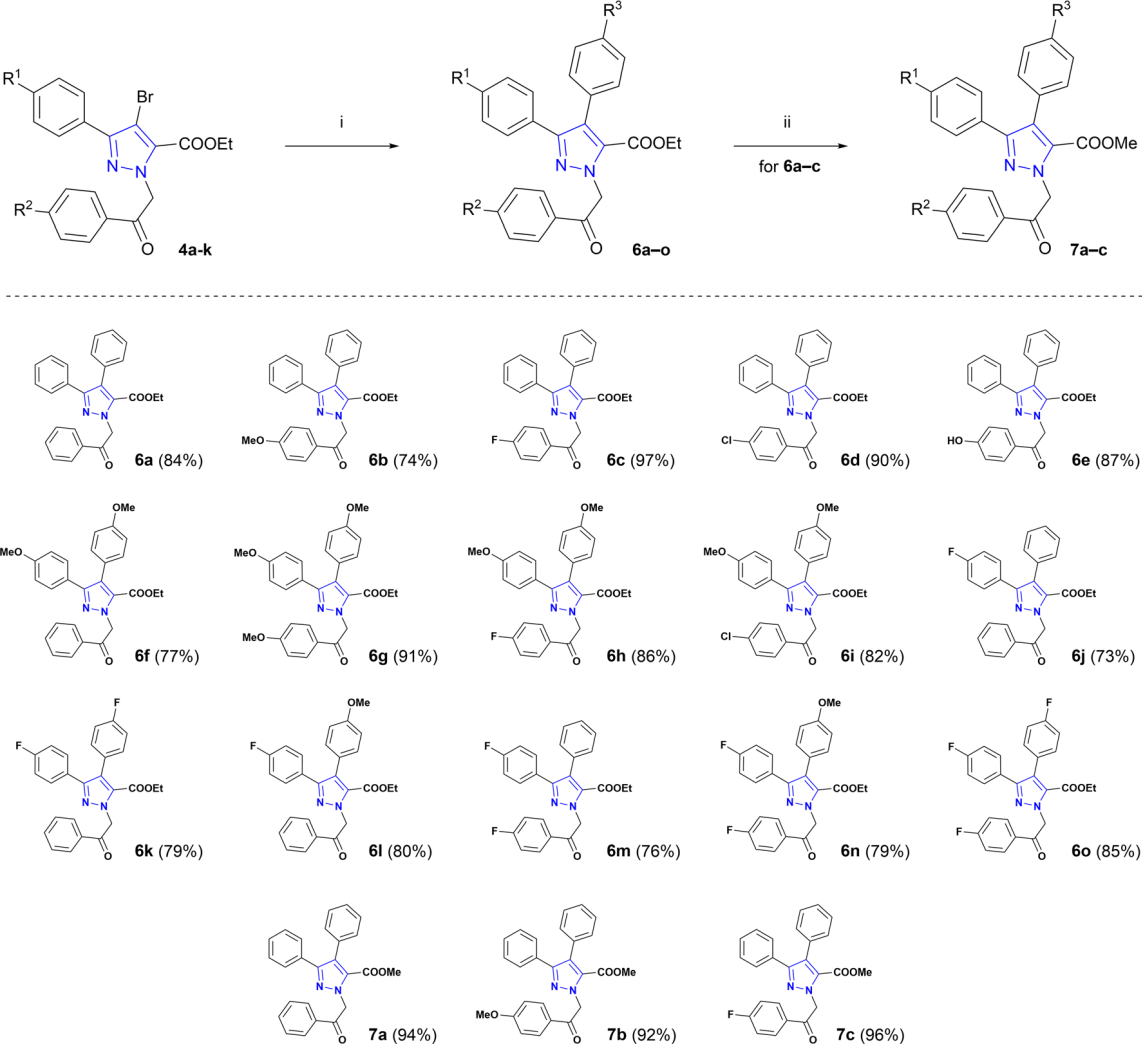
Synthesis of target pyrazole core-bearing lamellarin O analogues. Reagents and conditions: (i) appropriate phenylboronic acid, Pd(PPh_3_)_4_, Cs_2_CO_3_, dioxane, H_2_O, MW, 100 °C, 1 h; (ii) K_2_CO_3_, MeOH, reflux, 3 h.

Optimized Suzuki cross-coupling reaction conditions were applied to evaluate the scope of the reaction and to couple phenyl-, 4-methoxyphenyl- and 4-fluorophenylboronic acids with *N*-alkylated pyrazole-5-carboxylates 4a–k (Scheme 2). Microwave-assisted Suzuki cross-coupling was successfully exploited to synthesize a library of ethyl 3,4-diaryl-1-(2-aryl-2-oxoethyl)-1*H*-pyrazole-5-carboxylates 6a–o in 73–97% yields. Additionally, three 3,4-diaryl-1*H*-pyrazole-5-carboxylates 6a–c underwent transesterification in the presence of K_2_CO_3_ in refluxing methanol to derive methyl carboxylates 7a–c in 92–96% yields.

### Physicochemical properties

Estimation of the drug-likeness is facilitated by the evaluation of physicochemical properties and their causal relationships to predict pharmacokinetics. Despite the development of the rule of five reported by Lipinski *et al.*^55^ to identify key properties for potential bioavailability in drug design, natural products and drugs based on naturally occurring compounds were not explicitly included in this systematization.^56^

The lipophilicity of compounds 6a–o and 7a–c was estimated using both, high throughput chromatographic method employing an octadecyl–poly(vinyl alcohol) stationary phase^57^ and computational methods (Table 2). Besides, topological polar surface area (tPSA), p*K*_a_ of strongest acid as well as hydrogen bond donors (HBD) and acceptors (HBA) were calculated (see ESI, Table S2†). The calculated log *P* (*c* log *P*) and measured HPLC–log *P* were in a narrow range of 4.81–5.84 and 3.803–4.784, respectively, with the hydroxy-substituted derivative 6e being the least lipophilic one. All natural products, lamellarin O, lamellarin D, lamellarin I and lukianol A had a lower lipophilicity in the range of 3.79–4.57 for *c* log *P*. A similar trend was observed for the calculated tPSA values, where the pyrazole-based derivatives presented a reduced polarity. The *N*-substitution pattern on the pyrazole ring, based on the structural similarity to lamellarin O, was the main reason for the increase of lipophilicity. Once the nitrogen in position 1 is substituted, the pyrazole loses the amphoteric properties^58^ in parallel to the lower HOMO energy of the pyrazole *versus* pyrrole ring.^32^

**Table 2:** Comparison of HPLC–log *P* and *c* log *P* data of ethyl and methyl 3,4-diaryl-1-(2-aryl-2-oxoethyl)-1*H*-pyrazole-5-carboxylates 6a–o and 7a–c

Compound	HPLC–log *P*^a^	*c* log *P*^b^
Lamellarin O	—	4.00
Lukianol A	—	4.57
Lamellarin D	—	3.79
Lamellarin I	—	4.17
6a	4.381 ± 0.002	5.28
6b	4.468 ± 0.006	5.15
6c	4.453 ± 0.005	5.44
6d	4.711 ± 0.006	5.84
6e	3.803 ± 0.012	4.89
6f	4.462 ± 0.005	5.02
6g	4.531 ± 0.004	4.90
6h	4.531 ± 0.006	5.18
6i	4.784 ± 0.005	5.58
6j	4.490 ± 0.006	5.44
6k	4.546 ± 0.005	5.59
6l	4.499 ± 0.006	5.31
6m	4.545 ± 0.006	5.59
6n	4.564 ± 0.004	5.47
6o	4.598 ± 0.004	5.75
7a	4.279 ± 0.005	4.94
7b	4.347 ± 0.007	4.81
7c	4.327 ± 0.007	5.10

a Data is provided as a mean value ± standard deviation (SD) of at least three independent experiments.

b Calculated using ChemDraw 13.0.

Taken together, being as close as possible to the structure of lamellarin O, all pyrazole-based derivatives are outliers of Lipinski's rule of five. However, it is reported that natural products and compounds derived from natural products are among the most favourable exceptions from the Lipinski's rule of five.^59^

### Biological evaluation

Synthesized ethyl and methyl 3,4-diaryl-1-(2-aryl-2-oxoethyl)-1*H*-pyrazole-5-carboxylates 6a–o and 7a–c were first evaluated for their cytotoxicity against three human colorectal cancer cell lines HCT116, HT29 and SW480 (Table 3). Six out of eighteen compounds did not reach GI_50_ in the given 20 μM concentration range because of their wide therapeutic window and low solubility in cell culture medium. Ethyl 3,4-diaryl-1-(2-aryl-2-oxoethyl)-1*H*-pyrazole-5-carboxylates 6c, h, j, k, m–o, and methyl 1-[2-(4-fluorophenyl)-2-oxoethyl]-3,4-diphenyl-1*H*-py-razole-5-carboxylate (7c) have shown activity in the low micromolar range towards all tested cell lines. In most of the cases compounds were more active towards HCT116 cells, except for ethyl 3,4-diaryl-1-(2-aryl-2-oxoethyl)-1*H*-pyrazole-5-carboxylates 6m–o which have shown slightly higher cytotoxicity against SW480 cells. As expected, compounds bearing fluorine substituent demonstrated the best results.^60^ Fluorine substitution at R^2^ had the highest impact on advantageous cytotoxicity, whereas fluorine substitution on R^1^ and R^3^ had only minor or no effects. Among them, compound 6m proved to be the most active and reached lowest GI_50_ concentrations of 1.456 μM, 2.688 μM, and 1.441 μM towards HCT116, HT29 and SW480 cells, respectively.

**Table 3:** *In vitro* cytotoxicity of synthesized compounds

Compound	General structure	R^1^	R^2^	R^3^	R^4^	GI_50_ ± SD^a^ (μM)		
						HCT116	HT29	SW480
6a	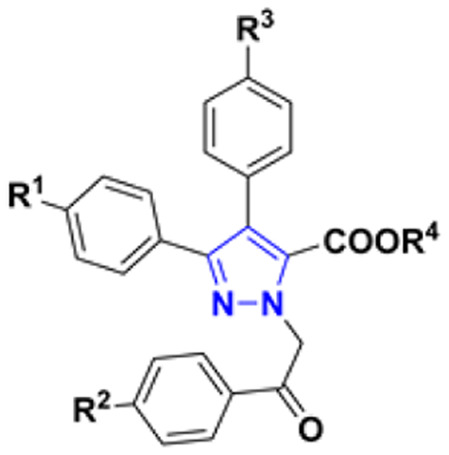	H	H	H	Et	5.462 ± 0.393	>20	>20
6b		H	OMe	H	Et	>20	>20	>20
6c		H	F	H	Et	1.964 ± 0.266	4.979 ± 2.620	5.523 ± 3.719
6d		H	Cl	H	Et	14.459 ± 3.915	>20	>20
6e		H	OH	H	Et	>20	>20	>20
6f		OMe	H	OMe	Et	2.293 ± 0.218	>20	>20
6g		OMe	OMe	OMe	Et	>20	>20	>20
6h		OMe	F	OMe	Et	2.072 ± 1.450	4.759 ± 2.331	4.759 ± 2.331
6i		OMe	Cl	OMe	Et	>20	>20	>20
6j		F	H	H	Et	3.751 ± 1.056	13.757 ± 0.861	9.243 ± 2.147
6k		F	H	F	Et	3.386 ± 0.620	11.125 ± 2.619	7.807 ± 2.017
6l		F	H	OMe	Et	5.051 ± 1.164	>20	8.275 ± 2.339
6m		F	F	H	Et	1.456 ± 0.247	2.688 ± 0.110	1.441 ± 0.173
6n		F	F	OMe	Et	2.097 ± 0.198	6.783 ± 0.463	1.641 ± 0.022
6o		F	F	F	Et	2.362 ± 0.039	6.666 ± 2.121	2.088 ± 0.032
7a		H	H	H	Me	>20	>20	>20
7b		H	OMe	H	Me	>20	>20	>20
7c		H	F	H	Me	2.699 ± 0.689	9.725 ± 3.711	>20

a Data is provided as a mean value ± standard deviation (SD) of at least three independent experiments.

Comparing synthesized ethyl carboxylates 6a–c with their methyl esters 7a–c it was noticed that ethyl group positively affects activity of the compounds. Interestingly, compound 6a appeared to be selective towards HCT116, and 6c, bearing fluorine atom at 4′-position of acetophenone, was active against all tested cell lines. In comparison, compounds 7a, b showed no activity at all, whereas methyl carboxylate 7c was less active than its counterpart 6c.

**Fig. 3:**
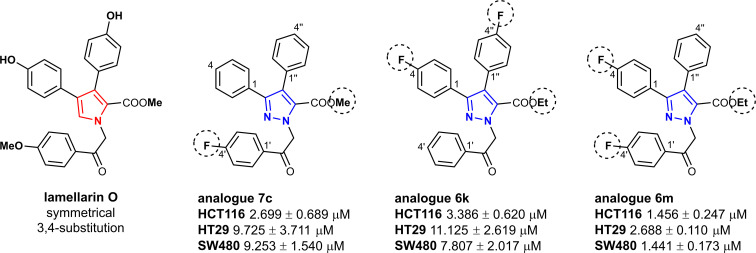
Structure and activity comparison of compounds 6k, 6m, and 7c.

Influence of the substituent at 4′-position (Fig. 3) was evaluated in pyrazoles having identical substituents at 3- and 4-position. Interestingly, ethyl 3,4-diaryl-1-(2-aryl-2-oxoethyl)-1*H*-pyrazole-5-carboxylates 6a and 6f with no substituents at 4′-position were more active compared to those possessing Cl, OH or OMe groups and, as anticipated, fluorine substituent improved cytotoxicity of both 6c and 6h towards all cell lines. Additionally, presence of OMe group at both 4- and 4′′-positions of compound 6f improved activity against HCT116 cells two-fold compared to carboxylate 6a. In case of ethyl 3,4-diaryl-1-(2-aryl-2-oxoethyl)-1*H*-pyrazole-5-carboxylates 6j, l, m, n containing fluorine atom, it was noticed that compounds with different substituents at 3,4-positions do not lose their activity. On the contrary, most active compound 6m possessed 4-fluorophenyl and phenyl substituents in the 3- and 4-positions of the pyrazole ring respectively.

The significant cytotoxicity of pyrazole-based derivatives motivated us to investigate the mode of action of the most potent compounds. First, we evaluated the compounds by a conventional live/dead counterstain including propidium iodide (PI), Hoechst 33258 and Calcein AM.^61^ Calcein AM is a well-described cell-permeable dye staining living cells, whereas PI can only bind to DNA when the cellular membrane is disrupted and allows the visualization of necrotic cells. Hoechst 33258 stain intensity increases during apoptosis due to nuclear condensation indicating apoptosis. The absence of any PI staining of the cells indicated the absence of necrotic and late-apoptotic cells for all tested compounds in all three colorectal cancer cells (ESI Fig. S134–S136†).

**Fig. 4:**
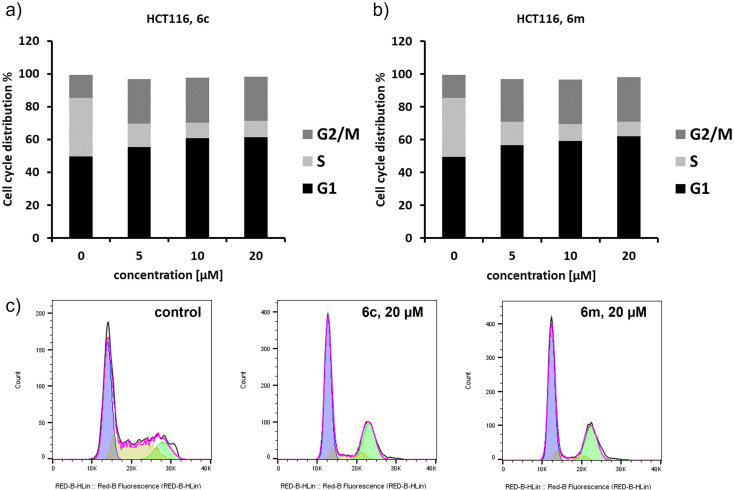
Cell cycle analysis in HCT116 cells after treatment with 6c and 6m: (a) dose-dependent increase of G1 and G2/M phase for 6c; (b) dose-dependent increase of G1 and G2/M phase for 6m; (c) representative histograms for the negative control, and at 20 μM of 6c and 6m. Data is provided as mean value of at least three independent experiments.

Cell cycle distribution was determined using flow cytometry in order to verify if chosen ethyl 3,4-diaryl-1-(2-aryl-2-oxoethyl)-1*H*-pyrazole-5-carboxylates 6c and 6m could induce cell cycle arrest. HCT116 cells were treated for 72 h with the respective compounds in a dose-dependent manner. As shown in Fig. 4, treatment of HCT116 cells with target compounds resulted in an induction of G1 and predominantly G2/M cell cycle arrest for both compounds already for the lowest concentration of 5 μM. Compared to negative control cells, the G1 phase increased significantly from 49.5 ± 1.8% to 63.2 ± 1.4% and 61.0 ± 1.8% for 6c and 6m respectively. In parallel, the G2/M phase increased from 14.2 ± 1.1% to 25.0 ± 0.5% and 26.9 ± 0.8%, respectively. No significant increase of cell debris was observed over the whole concentration range. Cell cycle arrest in the G2 phase was also described for lamellarin D in P388 leukaemia cells,^62^ whereas to the best of our knowledge no mode of action studies were published for lamellarin O. However, it seems likely that our newly synthesized isosteric analogues of lamellarin O and lamellarin D exhibit comparable cytotoxicity and cellular effects.

Additionally, plasma protein binding (PPB) experiments by means of a high-throughput HPLC method applying albumin-bound stationary phase were performed with selected ethyl 3,4-diaryl-1-(2-aryl-2-oxoethyl)-1*H*-pyrazole-5-carboxylates 6a, c, f, h, m–o. Compounds 6a, c, m–o showed very strong (above 95%) binding to human serum albumin (see ESI, Table S3†). The relationship between %PPB and calculated log *P* values correlated – increased compound lipophilicity results in stronger binding to albumin. However, studied compounds bind differently than would have been expected only from their lipophilicity, as the HPLC–log *P* values did not show linear correlation with %PPB data. High PPB decreases the free plasma fraction of the drug being part of the free drug hypothesis stating that only the unbound fraction of a drug can unfold their biological efficacy.^63^ This hypothesis is opposing the evidence of albumin being a versatile drug carrier *via* the enhanced permeability and retention (EPR) effect for oncological issues and that 45% of newly approved drugs between 2003 and 2013 possessed PPB of >95%.^64^

## Materials and methods

### Chemistry

#### General

The reagents and solvents were purchased from commercial suppliers and used without further purification unless indicated otherwise. Microwave-assisted reactions were conducted using a CEM Discover Synthesis Unit (CEM Corp., Matthews, NC, USA). The purification of the reaction mixtures was performed using flash chromatography on a glass column with silica gel (high-purity grade (9385), 60 Å, 230–400 mesh, Merck KGaA, Darmstadt, Germany). For thin layer chromatography, ALUGRAM® pre-coated TLC plates (Silica gel 60 F254, MACHEREY-NAGEL GmbH & Co. KG, Düren, Germany) were employed. Melting points were determined using DigiMelt MPA160 apparatus (Nyköping, Sweden) and are uncorrected. The IR spectra were recorded on a Brüker TENSOR 27 (Brüker Optik GmbH, Ettlingen, Germany) spectrometer using KBr pellets. NMR spectra were recorded using Brüker Avance III spectrometer (400 MHz for ^1^H NMR, 100 MHz for ^13^C NMR, 40 MHz for ^15^N NMR; Brüker BioSpin AG, Fallanden, Switzerland) at 25 °C. Residual solvent signals were used as internal standards, *i.e.*, for DMSO-d_6_*δ*^1^_H_ = 2.50 and *δ*^13^_C_ = 39.52, for CDCl_3_*δ*^1^_H_ = 7.26 and *δ*^13^_C_ = 77.16, for acetone-d_6_*δ*^1^_H_ = 2.05 and *δ*^13^_C_ = 29.84. A neat external nitromethane standard was used to recalculate ^15^N chemical shifts. The full and unambiguous assignments of the ^1^H, ^13^C, ^15^N-NMR resonances were achieved using standard Brüker software and a combination of advanced NMR spectroscopic techniques. High-resolution mass spectra were recorded on a micrOTOF-Q III Brüker spectrometer (Brüker Daltonik GmbH, Bremen, Germany) in electrospray ionization mode.

#### Synthesis of ethyl 3(5)-aryl-1*H*-pyrazole-5(3)-carboxylates 2a–c (ref. 42)

To a 0.5 M solution of sodium ethoxide (1.1 eq.) in ethanol, appropriate acetophenone 1a–c (1 eq.) and diethyl oxalate (1 eq.) were added, and resulting mixture was stirred at room temperature for 16 h under argon atmosphere. Upon completion, reaction mixture was quenched with 1 M HCl solution until neutral pH and extracted with EtOAc. Combined organic layers were washed with brine, dried over anhydrous Na_2_SO_4_, filtered and concentrated under reduced pressure. The residue was purified by column chromatography on silica gel (Hex/EtOAc 15/1, v/v). Resulting ketoester (1 eq.) was dissolved in a mixture of EtOH/AcOH (7/3, v/v) to obtain 0.2 M solution. Hydrazine hydrate (1.1 eq.; 55% aqueous solution) was added, and the reaction mixture was stirred at room temperature for 16 h. Subsequently, solvents were evaporated, the resulting residue was dissolved in EtOAc and washed with 10% aqueous NaHCO_3_ solution and brine, sequentially dried over anhydrous Na_2_SO_4_, filtered and concentrated under reduced pressure. The residue was purified by column chromatography on silica gel (Hex/EtOAc/MeOH gradient from 8/1/0.1 to 1/1/0.1, v/v/v) to give corresponding pyrazoles 2a–c in 83–87% yield.

#### Synthesis of ethyl 3(5)-aryl-4-bromo-1*H*-pyrazole-5(3)-carboxylates 3a–c (ref. 43)

An appropriate pyrazole 2a–c (1 eq.) was dissolved in DCM (0.2 M), NBS (1.5 eq.) was added, and the reaction mixture was stirred at 35 °C for 16 h. After full conversion of the starting material reaction mixture was washed with water and brine. Organic phase was separated, dried over anhydrous Na_2_SO_4_, filtered and concentrated under reduced pressure. Crude was purified by column chromatography (gradient from Hex/EtOAc 8/1 to 4/1, v/v) to give products 3a–c in 88–93% yield.

#### Synthesis of ethyl 3-aryl-4-bromo-1-(2-arylethyl-2-oxo)-1*H*-pyrazole-5-carboxylates 4a–k, 5a

An appropriate pyrazole 3a–c (1 mmol) was dissolved in dry DMF (0.7 M), Na_2_CO_3_ (2 mmol), corresponding 2-bromoacetophenone (1.05 mmol) was added, and the reaction mixture was stirred at 70 °C for 8 h. After full conversion of the starting material, reaction mixture was diluted with EtOAc and washed with water and brine. Organic phase was separated, dried over anhydrous Na_2_SO_4_, filtered and concentrated under reduced pressure. Crude was purified by column chromatography to yield products 4a–k, 5a.

##### Ethyl 4-bromo-1-(2-oxo-2-phenylethyl)-3-phenyl-1*H*-pyrazole-5-carboxylate (4a)

White solid, yield 62% (256 mg). *R*_f_ = 0.69 (*n*-hexane/ethyl acetate 7/3, v/v), mp 93–94 °C. IR (KBr) *ν*_max_, cm^−1^: 2982, 2952, 1704 (C=O), 1448, 1269, 1091, 688. ^1^H NMR (400 MHz, DMSO-d_6_) *δ*_H_ ppm: 1.15 (t, *J* = 7.1 Hz, 3H, CH_3_), 4.23 (q, *J* = 7.1 Hz, 2H, OCH_2_), 6.23 (s, 2H, NCH_2_), 7.43–7.54 (m, 3H, 3-Ph 3,4,5-H), 7.58–7.64 (m, 2H, C(O)Ph 3,5-H), 7.71–7.77 (m, 1H, C(O)Ph 4-H), 7.79–7.84 (m, 2H, 3-Ph 2, 6-H), 8.06–8.10 (m, 2H, C(O)Ph 2,6-H). ^13^C NMR (101 MHz, DMSO-d_6_) *δ*_C_ ppm: 13.7 (CH_3_), 60.3 (NCH_2_), 61.5 (OCH_2_), 97.0 (C-4), 127.8 (3-Ph C-2,6), 128.1 (C(O)Ph C-2,6), 128.6 (3-Ph C-3,5), 128.7 (3-Ph C-4), 129.0 (C(O)Ph C-3,5), 130.9 (3-Ph C-1), 132.1 (C-5), 134.1 (C(O)Ph C-1), 134.2 (C(O)Ph C-4), 148.4 (C-3), 158.1 (COO), 192.7 (C=O). ^15^N NMR (40 MHz, DMSO-d_6_) *δ*_N_ ppm: −175.9 (N-1), −63.2 (N-2). HRMS (ESI) for C_20_H_17_BrN_2_NaO_3_ ([M + Na]^+^): calcd *m*/*z* 435.0315, found *m*/*z* 435.0316.

##### Ethyl 4-bromo-1-[2-(4-methoxyphenyl)-2-oxoethyl]-3-phenyl-1*H*-pyrazole-5-carboxylate (4b)

White solid, yield 60% (266 mg). *R*_f_ = 0.61 (*n*-hexane/ethyl acetate 7/3, v/v), mp 131–132 °C. IR (KBr) *ν*_max_, cm^−1^: 3035, 3006, 2977, 2947, 1714 (C=O), 1601, 1449, 1174, 694. ^1^H NMR (400 MHz, CDCl_3_) *δ*_H_ ppm: 1.26 (t, *J* = 7.1 Hz, 3H, CH_3_), 3.82 (s, 3H, OCH_3_), 4.26 (q, *J* = 7.1 Hz, 2H, OCH_2_), 5.95 (s, 2H, NCH_2_), 6.87–6.95 (m, 2H, C(O)Ph 3,5-H), 7.28–7.41 (m, 3H, 3-Ph 3,4,5-H), 7.75–7.82 (m, 2H, 3-Ph 2,6-H), 7.86–7.94 (m, 2H, C(O)Ph 2,6-H). ^13^C NMR (101 MHz, CDCl_3_) *δ*_C_ ppm: 14.1 (CH_3_), 55.7 (OCH_3_), 59.7 (NCH_2_), 61.8 (OCH_2_), 98.3 (C-4), 114.3 (C(O)Ph C-3,5), 127.6 (C(O)Ph C-1), 128.44 (3-Ph C-3,5), 128.46 (3-Ph C-2,6), 128.7 (3-Ph C-4), 130.4 (C(O)Ph C-2,6), 131.5 (3-Ph C-1), 132.7 (C-5), 150.0 (C-3), 159.4 (COO), 164.3 (C(O)Ph C-4), 190.1 (C=O). ^15^N NMR (40 MHz, CDCl_3_) *δ*_N_ ppm: −179.4 (N-1), −65.7 (N-5). HRMS (ESI) for C_21_H_19_BrN_2_NaO_4_ ([M + Na]^+^): calcd *m*/*z* 465.0420, found *m*/*z* 465.0423.

##### Ethyl 4-bromo-1-[2-(4-fluorophenyl)-2-oxoethyl]-3-phenyl-1*H*-pyrazole-5-carboxylate (4c)

White solid, yield 70% (302 mg). *R*_f_ = 0.64 (*n*-hexane/ethyl acetate 7/1, v/v), mp 96–97 °C. IR (KBr) *ν*_max_, cm^−1^: 3071, 2985, 2946, 1704 (C=O), 1595, 1090, 836, 699. ^1^H NMR (400 MHz, CDCl_3_) *δ*_H_ ppm: 1.35 (t, *J* = 7.1 Hz, 3H, CH_3_), 4.34 (q, *J* = 7.1 Hz, 2H, OCH_2_), 6.03 (s, 2H, NCH_2_), 7.16–7.24 (m, 2H, C(O)Ph 3,5-H), 7.37–7.49 (m, 3H, 3-Ph 3,4,5-H), 7.82–7.89 (m, 2H, 3-Ph 2,6-H), 7.99–8.06 (m, 2H, C(O)Ph 2,6-H). ^13^C NMR (101 MHz, CDCl_3_) *δ*_C_ ppm: 14.1 (CH_3_), 59.8 (NCH_2_), 61.9 (OCH_2_), 98.4 (C-4), 116.38 (d, ^2^*J*_CF_ = 22.1 Hz, C(O)Ph C-3,5), 128.44 (3-Ph C-2,6), 128.47 (3-Ph C-3,5), 128.8 (3-Ph C-4), 130.84 (d, ^3^*J*_CF_ = 9.5 Hz, C(O)Ph C-2,6), 131.05 (d, ^4^*J*_CF_ = 3.1 Hz, C(O)Ph C-1), 131.41 (3-Ph C-1), 132.5 (C-5), 150.2 (C-3), 159.4 (COO), 166.40 (d, *J*_CF_ = 256.4 Hz, C(O)Ph C-4), 190.3 (C=O). ^15^N NMR (40 MHz, CDCl_3_) *δ*_N_ ppm: −180.0 (N-1), −65.7 (N-2). HRMS (ESI) for C_20_H_16_BrFN_2_NaO_3_ ([M + Na]^+^): calcd *m*/*z* 453.0221, found *m*/*z* 453.0218.

##### Ethyl 4-bromo-1-[2-(4-chlorophenyl)-2-oxoethyl]-3-phenyl-1*H*-pyrazole-5-carboxylate (4d)

White solid, yield 70% (313 mg). *R*_f_ = 0.77 (*n*-hexane/ethyl acetate 7/3, v/v), mp 87–88 °C. IR (KBr) *ν*_max_, cm^−1^: 3061, 2983, 2941, 1707 (C=O), 1580, 1226, 1092, 697. ^1^H NMR (400 MHz, CDCl_3_) *δ*_H_ ppm: 1.35 (t, *J* = 7.1 Hz, 3H, CH_3_), 4.33 (q, *J* = 7.1 Hz, 2H, OCH_2_), 6.01 (s, 2H, NCH_2_), 7.38–7.52 (m, 5H, 3-Ph 3,4,5-H; C(O)Ph 3,5-H), 7.84–7.89 (m, 2H, 3-Ph 2,6-H), 7.90–7.95 (m, 2H, C(O)Ph 2,6-H). ^13^C NMR (101 MHz, CDCl_3_) *δ*_C_ ppm: 14.1 (CH_3_), 59.8 (NCH_2_), 61.9 (OCH_2_), 98.4 (C-4), 128.41 (3-Ph C-2,6), 128.46 (3-Ph C-3,5), 128.8 (3-Ph C-4), 129.47 (C(O)Ph C-3,5), 129.48 (C(O)Ph C-2,6), 131.4 (3-Ph C-1), 132.5 (C-5), 132.9 (C(O)Ph C-1), 140.7 (C(O)Ph C-4), 150.1 (C-3), 159.4 (COO), 190.7 (C=O). ^15^N NMR (40 MHz, CDCl_3_) *δ*_N_ ppm: −180.1 (N-1), −65.6 (N-2). HRMS (ESI) for C_20_H_16_BrClN_2_NaO_3_ ([M + Na]^+^): calcd *m*/*z* 468.9925, found *m*/*z* 468.9923.

##### Ethyl 4-bromo-1-[2-(4-hydroxyphenyl)-2-oxoethyl]-3-phenyl-1*H*-pyrazole-5-carboxylate (4e)

White solid, yield 56% (240 mg). *R*_f_ = 0.63 (*n*-hexane/ethyl acetate 1/1, v/v), mp 191–192 °C. IR (KBr) *ν*_max_, cm^−1^: 3377 (OH), 2984, 2945, 1707 (C=O), 1580, 1176, 1090, 837. ^1^H NMR (400 MHz, DMSO-d_6_) *δ*_H_ ppm: 1.15 (t, *J* = 7.1 Hz, 3H, CH_3_), 4.22 (q, *J* = 7.1 Hz, 2H, OCH_2_), 6.10 (s, 2H, NCH_2_), 6.88–6.96 (m, 2H, C(O)Ph 3,5-H), 7.41–7.54 (m, 3H, 3-Ph 3,4,5-H), 7.76–7.83 (m, 2H, 3-Ph 2,6-H), 7.91–7.99 (m, 2H, C(O)Ph 2,6-H), 10.60 (s, 1H, OH). ^13^C NMR (101 MHz, DMSO-d_6_) *δ*_C_ ppm: 13.7 (CH_3_), 60.0 (NCH_2_), 61.5 (OCH_2_), 96.84 (C-4), 115.6 (C(O)Ph C-3,5), 125.6 (C(O)Ph C-1), 127.8 (3-Ph C-2,6), 128.6 (3-Ph C-3,5), 128.7 (3-Ph C-4), 130.8 (C(O)Ph C-2,6), 131.0 (3-Ph C-1), 132.3 (C-5), 148.3 (C-3), 158.1 (COO), 163.0 (C(O)Ph C-4), 190.5 (C=O). ^15^N NMR (40 MHz, DMSO-d_6_) *δ*_N_ ppm: −175.1 (N-1), −63.4 (N-2). HRMS (ESI) for C_20_H_17_BrN_2_NaO_4_ ([M + Na]^+^): calcd *m*/*z* 451.0264, found *m*/*z* 451.0265.

##### Ethyl 4-bromo-3-(4-methoxyphenyl)-1-(2-oxo-2-phenylethyl)-1*H*-pyrazole-5-carboxylate (4f)

White solid, yield 63% (279 mg). *R*_f_ = 0.79 (*n*-hexane/ethyl acetate 7/3, v/v), mp 152–153 °C. IR (KBr) *ν*_max_, cm^−1^: 3068, 2955, 2835, 1704 (C=O), 1455, 1251, 1177, 1093, 1029, 841. ^1^H NMR (400 MHz, DMSO-d_6_) *δ*_H_ ppm: 1.14 (t, *J* = 7.1 Hz, 3H, CH_3_), 3.81 (s, 3H, OCH_3_), 4.22 (q, *J* = 7.0 Hz, 2H, OCH_2_), 6.19 (s, 2H, NCH_2_), 7.01–7.11 (m, 2H, 3-Ph 3,5-H), 7.56–7.65 (m, 2H, C(O)Ph 3,5-H), 7.70–7.79 (m, 3H, C(O)Ph 4-H; 3-Ph 2,6-H), 8.03–8.11 (m, 2H, C(O)Ph 2,6-H). ^13^C NMR (101 MHz, DMSO-d_6_) *δ*_C_ ppm: 13.7 (CH_3_), 55.2 (OCH_3_), 60.3 (NCH_2_), 61.5 (OCH_2_), 96.7 (C-4), 114.0 (3-Ph C-3,5), 123.3 (3-Ph C-1), 128.1 (C(O)Ph C-2,6), 129.0 (C(O)Ph C-3,5), 129.2 (3-Ph C-2,6), 132.0 (C-5), 134.15 (C(O)Ph C-1), 134.23 (C(O)Ph C-4), 148.3 (C-3), 158.1 (COO), 159.6 (3-Ph C-4), 192.8 (C=O). ^15^N NMR (40 MHz, DMSO-d_6_) *δ*_N_ ppm: −177.1 (N-1), −64.3 (N-2). HRMS (ESI) for C_21_H_19_BrN_2_NaO_4_ ([M + Na]^+^): calcd *m*/*z* 465.0420, found *m*/*z* 465.0418.

##### Ethyl 4-bromo-3-(4-methoxyphenyl)-1-[2-(4-methoxyphenyl)-2-oxoethyl]-1*H*-pyrazole-5-carboxylate (4g)

White solid, yield 56% (265 mg). *R*_f_ = 0.59 (*n*-hexane/ethyl acetate 7/3, v/v), mp 109–110 °C. IR (KBr) *ν*_max_, cm^−1^: 2991, 2945, 2839, 1709 (C=O), 1689, 1234, 1176, 1030, 840. ^1^H NMR (400 MHz, CDCl_3_) *δ*_H_ ppm: 1.33 (t, *J* = 7.1 Hz, 3H, CH_3_), 3.85 (s, 3H, Ph 4-OCH_3_), 3.89 (s, 3H, C(O)Ph 4-OCH_3_), 4.32 (q, *J* = 7.0 Hz, 2H, OCH_2_), 6.00 (s, 2H, NCH_2_), 6.92–7.04 (m, 4H, C(O)Ph 3,5-H; 3-Ph 3,5-H), 7.74–7.85 (m, 2H, 3-Ph 2,6-H), 7.93–8.00 (m, 2H, C(O)Ph 2,6-H). ^13^C NMR (101 MHz, CDCl_3_) *δ*_C_ ppm: 14.1 (CH_3_), 55.4 (3-Ph 4-OCH_3_), 55.7 (C(O)Ph 4-OCH_3_), 59.6 (NCH_2_), 61.8 (OCH_2_), 98.0 (C-4), 113.9 (3-Ph C-3,5), 114.3 (C(O)Ph C-3,5), 124.1 (3-Ph C-1), 127.6 (C(O)Ph C-1), 129.8 (3-Ph C-2,6), 130.4 (C(O)Ph C-2,6), 132.5 (C-5), 149.8 (C-3), 159.4 (COO), 160.0 (3-Ph C-4), 164.3 (C(O)Ph C-4), 190.2 (C=O). ^15^N NMR (40 MHz, CDCl_3_) *δ*_N_ ppm: −180.3 (N-1), −67.0 (N-2). HRMS (ESI) for C_22_H_21_BrN_2_NaO_5_ ([M + Na]^+^): calcd *m*/*z* 495.0526, found *m*/*z* 495.0523.

##### Ethyl 4-bromo-1-[2-(4-fluorophenyl)-2-oxoethyl]-3-(4-methoxy-phenyl)-1*H*-pyrazole-5-carboxylate (4h)

White solid, yield 65% (230 mg). *R*_f_ = 0.73 (*n*-hexane/ethyl acetate 3/2, v/v), mp 157–158 °C. IR (KBr) *ν*_max_, cm^−1^: 3083 and 3073 (doublet), 2951, 2934, 2837, 1704 (C=O), 1598, 1268, 839, 611 and 591 (doublet). ^1^H NMR (400 MHz, CDCl_3_) *δ*_H_ ppm: 1.37 (t, *J* = 7.1 Hz, 3H, CH_3_), 3.87 (s, 3H, OCH_3_), 4.35 (q, *J* = 7.0 Hz, 2H, OCH_2_), 6.04 (s, 2H, NCH_2_), 6.96–7.03 (m, 2H, 3-Ph 3,5-H), 7.18–7.26 (m, 2H, C(O)Ph 3,5-H), 7.78–7.85 (m, 2H, 3-Ph 2,6-H), 8.01–8.08 (m, 2H, C(O)Ph 2,6-H). ^13^C NMR (101 MHz, CDCl_3_) *δ*_C_ ppm: 14.1 (CH_3_), 55.4 (OCH_3_), 59.8 (NCH_2_), 61.9 (OCH_2_), 98.2 (C-4), 113.9 (3-Ph C-3,5), 116.37 (d, ^2^*J*_CF_ = 22.1 Hz, C(O)Ph C-3,5), 124.0 (3-Ph C-1), 129.8 (3-Ph C-2,6), 130.83 (d, ^3^*J*_CF_ = 9.5 Hz, C(O)Ph C-2,6), 131.09 (d, ^4^*J*_CF_ = 3.0 Hz, C(O)Ph C-1), 132.4 (C-5), 150.0 (C-3), 159.5 (COO), 160.1 (3-Ph C-4), 166.39 (d, *J*_CF_ = 256.3 Hz, C(O)Ph C-4), 190.4 (C=O). ^15^N NMR (40 MHz, CDCl_3_) *δ*_N_ ppm: −180.9 (N-1). −67.1 (N-2). HRMS (ESI) for C_21_H_18_BrFN_2_NaO_4_ ([M + Na]^+^): calcd *m*/*z* 483.0326, found *m*/*z* 483.0332.

##### Ethyl 4-bromo-1-[2-(4-chlorophenyl)-2-oxoethyl]-3-(4-methoxy-phenyl)-1*H*-pyrazole-5-carboxylate (4i)

White solid, yield 63% (301 mg). *R*_f_ = 0.70 (*n*-hexane/ethyl acetate 7/3, v/v), mp 119–120 °C. IR (KBr) *ν*_max_, cm^−1^: 2993, 2952, 1705 (C=O), 1252, 1177, 1092, 1025, 842. ^1^H NMR (400 MHz, DMSO-d_6_) *δ*_H_ ppm: 1.15 (t, *J* = 7.1 Hz, 3H, CH_3_), 3.81 (s, 3H, OCH_3_), 4.22 (q, *J* = 7.1 Hz, 2H, OCH_2_), 6.19 (s, 2H, NCH_2_), 7.01–7.10 (m, 2H, 3-Ph 3,5-H), 7.66–7.77 (m, 4H, C(O)Ph 3,5-H; 3-Ph 2,6-H), 8.05–8.11 (m, 2H, C(O)Ph 2,6-H). ^13^C NMR (101 MHz, DMSO-d_6_) *δ*_C_ ppm: 13.7 (CH_3_), 55.2 (OCH_3_), 60.2 (NCH_2_), 61.5 (OCH_2_), 96.7 (C-4), 114.0 (3-Ph C-3,5), 123.3 (3-Ph C-1), 129.14 (3-Ph C-2,6), 129.17 (C(O)Ph C-3,5), 130.0 (C(O)Ph C-2,6), 131.9 (C-5), 132.9 (C(O)Ph C-1), 139.2 (C(O)Ph C-4), 148.3 (C-3), 158.1 (COO), 159.6 (3-Ph C-4), 192.0 (C=O). ^15^N NMR (40 MHz, DMSO-d_6_) *δ*_N_ ppm: −177.2 (N-1), −64.5 (N-2). HRMS (ESI) for C_21_H_18_BrClN_2_NaO_4_ ([M + Na]^+^): calcd *m*/*z* 499.0031, found *m*/*z* 499.0029.

##### Ethyl 4-bromo-3-(4-fluorophenyl)-1-(2-oxo-2-phenylethyl)-1*H*-pyrazole-5-carboxylate (4j)

White solid, yield 70% (302 mg). *R*_f_ = 0.70 (*n*-hexane/ethyl acetate 7/3, v/v), mp 97–98 °C. IR (KBr) *ν*_max_, cm^−1^: 3001 and 2982 (doublet), 2938, 2360, 2342, 1708 (C=O), 1445, 1255, 1226, 1165, 844, 756, 688. ^1^H NMR (400 MHz, CDCl_3_) *δ*_H_ ppm: 1.34 (t, *J* = 7.1 Hz, 3H, CH_3_), 4.33 (q, *J* = 7.1 Hz, 2H, OCH_2_), 6.06 (s, 2H, NCH_2_), 7.09–7.18 (m, 2H, 3-Ph 3,5-H), 7.49–7.57 (m, 2H, C(O)Ph 3,5-H), 7.61–7.69 (m, 1H, C(O)Ph 4-H), 7.81–7.89 (m, 2H, 3-Ph 2,6-H), 7.97–8.03 (m, 2H, C(O)Ph 2,6-H). ^13^C NMR (101 MHz, CDCl_3_) *δ*_C_ ppm: 14.1 (CH_3_), 60.0 (NCH_2_), 61.9 (COO), 98.2 (C-4), 115.49 (d, ^2^*J*_CF_ = 21.6 Hz, 3-Ph C-3,5), 127.60 (d, ^4^*J*_CF_ = 3.3 Hz, 3-Ph C-1), 128.1 (C(O)Ph C-2,6), 129.2 (C(O)Ph C-3,5), 130.31 (d, ^3^*J*_CF_ = 8.4 Hz, 3-Ph C-2,6), 132.7 (C-5), 134.3 (C(O)Ph C-4), 134.6 (C(O)Ph C-1), 149.2 (C-3), 159.30 (COO), 163.12 (d, *J*_CF_ = 248.1 Hz, 3-Ph C-4), 191.7 (C=O). ^15^N NMR (40 MHz, CDCl_3_) *δ*_N_ ppm: −179.8 (N-1), −66.0 (N-2). HRMS (ESI) for C_20_H_16_BrFN_2_NaO_3_ ([M + Na]^+^): calcd *m*/*z* 453.0221, found *m*/*z* 453.0225.

##### Ethyl 4-bromo-3-(4-fluorophenyl)-1-[2-(4-fluorophenyl)-2-oxo-ethyl]-1*H*-pyrazole-5-carboxylate (4k)

White solid, yield 69% (310 mg). *R*_f_ = 0.77 (*n*-hexane/ethyl acetate 3/2, v/v), mp 99–100 °C. IR (KBr) *ν*_max_, cm^−1^: 3084, 2989, 2943, 1708 (C=O), 1598, 1443, 1233, 841. ^1^H NMR (400 MHz, DMSO-d_6_) *δ*_H_ ppm: 1.15 (t, *J* = 7.1 Hz, 3H, CH_3_), 4.23 (q, *J* = 7.0 Hz, 2H, OCH_2_), 6.21 (s, 2H, NCH_2_), 7.31–7.39 (m, 2H, 3-Ph 3,5-H), 7.41–7.49 (m, 2H, C(O)Ph 3,5-H), 7.81–7.88 (m, 2H, 3-Ph 2,6-H), 8.13–8.20 (m, 2H, C(O)Ph 2,6-H). ^13^C NMR (101 MHz, DMSO-d_6_) *δ*_C_ ppm: 13.7 (CH_3_), 60.3 (NCH_2_), 61.6 (OCH_2_), 96.9 (C-4), 115.59 (d, ^2^*J*_CF_ = 21.7 Hz, 3-Ph C-3,5), 116.15 (d, ^2^*J*_CF_ = 22.0 Hz, C(O)Ph C-3,5), 127.38 (d, ^4^*J*_CF_ = 3.2 Hz, 3-Ph C-1), 129.99 (d, ^3^*J*_CF_ = 8.5 Hz, 3-Ph C-2,6), 130.89 (d, ^4^*J*_CF_ = 2.8 Hz, C(O)Ph C-1), 131.24 (d, ^3^*J*_CF_ = 9.7 Hz, C(O)Ph C-2,6), 132.2 (C-5), 147.6 (C-3), 158.0 (COO), 162.28 (d, *J*_CF_ = 246.0 Hz, 3-Ph C-4), 165.59 (d, *J*_CF_ = 253.0 Hz, C(O)Ph C-4), 191.39 (C=O). ^15^N NMR (40 MHz, DMSO-d_6_) *δ*_N_ ppm: −175.9 (N-1), −63.7 (N-2). HRMS (ESI) for C_20_H_15_BrF_2_N_2_NaO_3_ ([M + Na]^+^): calcd *m*/*z* 471.0126, found *m*/*z* 471.0130.

##### Ethyl 4-bromo-1-(2-oxo-2-phenylethyl)-5-phenyl-1*H*-pyrazole-3-carboxylate (5a)

Pale yellow solid, yield 19% (79 mg). *R*_f_ = 0.35 (*n*-hexane/ethyl acetate 7/3, v/v), mp 148–149 °C. IR (KBr) *ν*_max_, cm^−1^: 2981, 2952, 1714 (C=O), 1460 and 1451 (doublet), 1226, 1054. ^1^H NMR (400 MHz, DMSO-d_6_) *δ*_H_ ppm: 1.31 (t, *J* = 7.1 Hz, 3H, CH_3_), 4.33 (q, *J* = 7.1 Hz, 2H, OCH_2_), 5.94 (s, 2H, NCH_2_), 7.35–7.41 (m, 2H, 3-Ph 2,6-H), 7.45–7.56 (m, 5H, 3-Ph 3,4,5-H; C(O)Ph 3,5-H), 7.65–7.71 (m, 1H, C(O)Ph 4-H), 7.89–7.95 (m, 2H, C(O)Ph 2,6-H). ^13^C NMR (101 MHz, DMSO-d_6_) *δ*_C_ ppm: 14.1 (CH_3_), 57.8 (NCH_2_), 60.7 (OCH_2_), 95.8 (C-4), 127.1 (3-Ph C-1), 128.1 (C(O)Ph C-2,6), 128.90 (3-Ph C-3,5), 128.94 (C(O)Ph C-3-5), 129.6 (3-Ph C-2,6), 129.9 (3-Ph C-4), 133.8 (C(O)Ph C-1), 134.4 (C(O)Ph C-4), 139.5 (C-3), 144.5 (C-5), 160.5 (COO), 192.7 (C=O). ^15^N NMR (40 MHz, DMSO-d_6_) *δ*_N_ ppm: −175.1 (N-1), −63.4 (N-2). HRMS (ESI) for C_20_H_17_BrN_2_NaO_3_ ([M + Na]^+^): calcd *m*/*z* 435.0315, found *m*/*z* 435.0313.

#### General procedure for synthesis of 1-(2-oxo-2-phenylethyl)-3,4-diphenyl-1*H*-pyrazole-5-carboxylic acid (6a′)

Ethyl 4-bromo-1-(2-oxo-2-phenylethyl)-3-phenyl-1*H*-pyrazole-5-carboxylate 4a (0.5 mmol) was dissolved in degassed mixture of DMF and water (5/1, v/v, 0.03 M), phenylboronic acid (0.6 mmol), K_3_PO_4_ (1.5 mmol) and Pd(PPh_3_)_4_ (0.025 mmol) were added, and reaction mixture was stirred in MW reactor at 140 °C for 1 h. Reaction mixture was filtered through the pad of celite. Filtrate was diluted with EtOAc and washed with water and brine. Organic phase was separated, dried over anhydrous Na_2_SO_4_, filtered, and concentrated under reduced pressure. Crude was purified by column chromatography (gradient from Hex/EtOAc 8/1 to 1/2, v/v) to yield coupled product 6a′.

##### 1-(2-Oxo-2-phenylethyl)-3,4-diphenyl-1*H*-pyrazole-5-carboxylic acid (6a′)

White solid, yield 77% (147 mg). *R*_f_ = 0.46 (ethyl acetate), mp 209–210 °C. IR (KBr) *ν*_max_, cm^−1^: 3058, 3029, 2989, 2940, 2556, 1716 (C=O), 1702 (C=O), 1449, 1228, 1089, 700. ^1^H NMR (400 MHz, DMSO-d_6_) *δ*_H_ ppm: 6.21 (s, 2H, CH_2_), 7.19–7.42 (m, 10H, 3-Ph 2,3,4,5,6-H; 4-Ph 2,3,4,5,6-H), 7.56–7.65 (m, 2H, C(O)Ph 3,5-H), 7.68–7.78 (m, 1H, C(O)Ph 4-H), 8.03–8.13 (m, 2H, C(O)Ph 2,6-H), 13.16 (s, 1H, COOH). ^13^C NMR (101 MHz, DMSO-d_6_) *δ*_C_ ppm: 59.3 (CH_2_), 123.8 (C-4), 127.3 (4-Ph C-4), 127.5 (4-Ph C-2,6), 127.7 (3-Ph C-4), 128.0 (3-Ph C-2,6), 128.1 (3-Ph C-3,5), 128.2 (C(O)Ph C-2,6), 129.0 (C(O)Ph C-3,5), 130.4 (4-Ph C-3,5), 132.2 (3-Ph C-1), 132.4 (C-5), 133.0 (4-Ph C-1), 134.0 (C(O)Ph C-4), 134.5 (C(O)Ph C-1), 147.8 (C-3), 160.8 (COOH), 193.2 (C=O). ^15^N NMR (40 MHz, DMSO-d_6_) *δ*_N_ ppm: −177.9 (N-1), −65.8 (N-2). HRMS (ESI) for C_24_H_19_N_2_O_3_ ([M + H]^+^): calcd *m*/*z* 383.1390, found *m*/*z* 383.1389.

#### General procedure for synthesis of ethyl 3,4-diaryl-1-(2-aryl-2-oxoethyl)-1*H*-pyrazole-5-carboxylates (6a–o)

An appropriate compound 4a–k (0.5 mmol) was dissolved in degassed mixture of dioxane and water (5/1, v/v, 0.03 M), appropriate aryl boronic acid (0.6 mmol), Cs_2_CO_3_ (1.5 mmol) and Pd(PPh_3_)_4_ (0.025 mmol) were added, and reaction mixture was stirred in MW reactor at 100 °C for 1 h. Reaction mixture was filtered through the pad of celite. Filtrate was diluted with EtOAc and washed with water and brine. Organic phase was separated, dried over anhydrous Na_2_SO_4_, filtered and concentrated under reduced pressure. Crude was purified by column chromatography (gradient from Hex/EtOAc 20/1 to 8/1, v/v) to yield coupled products 6a–o.

##### Ethyl 1-(2-oxo-2-phenylethyl)-3,4-diphenyl-1*H*-pyrazole-5-carboxylate (6a)

White solid, yield 84% (172 mg). *R*_f_ = 0.63 (*n*-hexane/ethyl acetate 7/3, v/v), mp 112–113 °C. IR (KBr) *ν*_max_, cm^−1^: 3063, 2987, 2932, 1701 (C=O), 1449, 1226, 1096, 763, 708. ^1^H NMR (400 MHz, CDCl_3_) *δ*_H_ ppm: 0.92 (t, *J* = 7.1 Hz, 3H, CH_3_), 4.03 (q, *J* = 7.1 Hz, 2H, OCH_2_), 6.13 (s, 2H, NCH_2_), 7.20–7.24 (m, 3H, 3-Ph 3,4,5-H), 7.28–7.37 (m, 5H, 4-Ph 2,3,4,5,6-H), 7.38–7.43 (m, 2H, 3-Ph 2,6-H), 7.50–7.56 (m, 2H, C(O)Ph 3,5-H), 7.61–7.67 (m, 1H, C(O)Ph 4-H), 8.01–8.06 (m, 2H, C(O)Ph 2,6-H). ^13^C NMR (101 MHz, CDCl_3_) *δ*_C_ ppm: 13.5 (CH_3_), 59.2 (NCH_2_), 61.0 (OCH_2_), 125.2 (C-4), 127.4 (4-Ph C-4), 127.8 (3-Ph C-4), 127.9 (4-Ph C-2,6), 128.12 (3-Ph C-2,6), 128.14 (C(O)Ph C-2,6), 128.3 (3-Ph C-3,5), 129.1 (C(O)Ph C-3,4), 130.8 (4-Ph C-3,5), 131.8 (C-5), 132.5 (3-Ph C-1), 133.3 (4-Ph C-1), 134.1 (C(O)Ph C-4), 134.9 (C(O)Ph C-1), 149.5 (C-3), 160.4 (COO), 192.5 (C=O). ^15^N NMR (40 MHz, CDCl_3_) *δ*_N_ ppm: −181.7 (N-1), −67.6 (N-2). HRMS (ESI) for C_26_H_22_N_2_NaO_3_ ([M + Na]^+^): calcd *m*/*z* 433.1523, found *m*/*z* 433.1523.

##### Ethyl 1-[2-(4-methoxyphenyl)-2-oxoethyl]-3,4-diphenyl-1*H*-pyrazole-5-carboxylate (6b)

White solid, yield 74% (163 mg). *R*_f_ = 0.28 (*n*-hexane/ethyl acetate 4/1, v/v), mp 144–145 °C. IR (KBr) *ν*_max_, cm^−1^: 3053, 2989, 2962, 2837, 1708 (C=O), 1601, 1235, 1176, 1095, 838, 699. ^1^H NMR (400 MHz, CDCl_3_) *δ*_H_ ppm: 0.91 (t, *J* = 6.8 Hz, 3H, CH_3_), 3.90 (s, 3H, OCH_3_), 3.98–4.08 (m, 2H, OCH_2_), 6.09 (s, 2H, NCH_2_), 6.95–7.06 (m, 2H, C(O)Ph 3,5-H), 7.18–7.25 (m, 3H, 3-Ph 3,4,5-H), 7.28–7.47 (m, 7H, 3-Ph 2,6-H; 4-Ph 2,3,4,5,6-H), 7.95–8.08 (m, 2H, C(O)Ph 2,6-H). ^13^C NMR (101 MHz, CDCl_3_) *δ*_C_ ppm: 13.6 (CH_3_), 55.7 (OCH_3_), 58.9 (CH_2_), 61.0 (OCH_2_), 114.3 (C(O)Ph C-3,5), 125.1 (C-4), 127.4 (4-Ph C-4), 127.78 (3-Ph C-4), 127.86 (C(O)Ph C-1), 127.91 (4-Ph C-2,6), 128.1 (3-Ph C-2,6), 128.3 (3-Ph C-3,5), 130.5 (C(O)Ph C-2,6), 130.8 (4-Ph C-3,5), 132.0 (C-5), 132.5 (3-Ph C-1), 133.3 (4-Ph C-1), 149.3 (C-3), 160.4 (COO), 164.2 (C(O)Ph C-4), 190.8 (C=O). ^15^N NMR (40 MHz, CDCl_3_) *δ*_N_ ppm: −181.0 (N-1), −68.5 (N-2). HRMS (ESI) for C_27_H_24_N_2_NaO_4_ ([M + Na]^+^): calcd *m*/*z* 463.1628, found *m*/*z* 463.1631.

##### Ethyl 1-[2-(4-fluorophenyl)-2-oxoethyl]-3,4-diphenyl-1*H*-pyrazole-5-carboxylate (6c)

White solid, yield 97% (208 mg). *R*_f_ = 0.71 (*n*-hexane/ethyl acetate 7/3, v/v), mp 114–115 °C. IR (KBr) *ν*_max_, cm^−1^: 3060, 2984, 2941, 1707 (C=O), 1598, 1231, 1095, 837, 699. ^1^H NMR (400 MHz, CDCl_3_) *δ*_H_ ppm: 0.92 (t, *J* = 7.1 Hz, 3H, CH_3_), 4.03 (q, *J* = 7.1 Hz, 2H, OCH_2_), 6.10 (s, 2H, NCH_2_), 7.17–7.25 (m, 5H, C(O)Ph 3,5-H; 3-Ph 3,4,5-H), 7.28–7.43 (m, 7H, 3-Ph 2,6-H; 4-Ph 2,3,4,5,6-H), 8.02–8.10 (m, 2H, C(O)Ph 2,6-H). ^13^C NMR (101 MHz, CDCl_3_) *δ*_C_ ppm: 13.5 (CH_3_), 59.0 (NCH_2_), 61.1 (OCH_2_), 116.32 (d, ^2^*J*_CF_ = 22.1 Hz, C(O)Ph C-3,5), 125.2 (C-4), 127.5 (4-Ph C-4), 127.9 (3-Ph C-4), 128.0 (4-Ph C-2,6), 128.1 (3-Ph C-2,6), 128.3 (3-Ph C-3,5), 130.8 (4-Ph C-3,5), 130.85 (d, ^3^*J*_CF_ = 9.7 Hz, C(O)Ph C-2,6), 131.31 (d, ^4^*J*_CF_ = 3.0 Hz, C(O)Ph C-1), 131.8 (C-5), 132.4 (3-Ph C-1), 133.2 (4-Ph C-1), 149.5 (C-4), 160.5 (COO), 166.33 (d, *J* = 256.1 Hz, C(O)Ph C-4), 190.9 (C=O). ^15^N NMR (40 MHz, CDCl_3_) *δ*_N_ ppm: −181.8 (N-1), −67.6 (N-2). ^19^F NMR (376 MHz, CDCl_3_) *δ*_F_ ppm: −103.4 (C(O)Ph 4-F). HRMS (ESI) for C_26_H_21_FN_2_NaO_3_ ([M + Na]^+^): calcd *m*/*z* 451.1428, found *m*/*z* 451.1431.

##### Ethyl 1-[2-(4-chlorophenyl)-2-oxoethyl]-3,4-diphenyl-1*H*-pyrazole-5-carboxylate (6d)

White solid, yield 90% (200 mg). *R*_f_ = 0.74 (*n*-hexane/ethyl acetate 7/3, v/v), mp 135–136 °C. IR (KBr) *ν*_max_, cm^−1^: 3062, 2999 and 2992 (doublet), 2939, 1702 (C=O), 1224, 1094, 696. ^1^H NMR (400 MHz, CDCl_3_) *δ*_H_ ppm: 0.91 (t, *J* = 7.1 Hz, 3H, CH_3_), 4.03 (q, *J* = 7.1 Hz, 2H, OCH_2_), 6.08 (s, 2H, NCH_2_), 7.19–7.24 (m, 3H, 3-Ph 3,4,5-H), 7.28–7.44 (m, 7H, 3-Ph 2,6-H; 4-Ph 2,3,4,5,6-H), 7.47–7.55 (m, 2H, C(O)Ph 3,5-H), 7.92–8.00 (m, 2H, C(O)Ph 2,6-H). ^13^C NMR (101 MHz, CDCl_3_) *δ*_C_ ppm: 13.4 (CH_3_), 58.9 (NCH_2_), 60.9 (OCH_2_), 125.2 (C-4), 127.3 (4-Ph C-4), 127.7 (3-Ph C-4), 127.8 (4-Ph C-2,6), 128.0 (3-Ph C-2,6), 128.2 (3-Ph C-3,5), 129.3 (C(O)Ph C-3,5), 129.4 (C(O)Ph C-2,6), 130.7 (4-Ph C-3,5), 131.6 (C-5), 132.3 (3-Ph C-1), 133.04 (C(O)Ph C-1), 133.07 (4-Ph C-1), 140.4 (C(O)Ph C-4), 149.4 (C-3), 160.3 (COO), 191.3 (C=O). ^15^N NMR (40 MHz, CDCl_3_) *δ*_N_ ppm: −182.0 (N-1), −67.7 (N-2). HRMS (ESI) for C_26_H_21_ClN_2_NaO_3_ ([M + Na]^+^): calcd *m*/*z* 467.1133, found *m*/*z* 467.1134.

##### Ethyl 1-[2-(4-hydroxyphenyl)-2-oxoethyl]-3,4-diphenyl-1*H*-pyrazole-5-carboxylate (6e)

White solid, yield 87% (186 mg). *R*_f_ = 0.68 (*n*-hexane/ethyl acetate 1/1, v/v), mp 143–144 °C. IR (KBr) *ν*_max_, cm^−1^: 3300 (OH), 3059, 2984, 2949, 1709 (C=O), 1603, 1234, 1170, 700. ^1^H NMR (400 MHz, DMSO-d_6_) *δ*_H_ ppm: 0.81 (t, *J* = 7.1 Hz, 3H, CH_3_), 3.95 (q, *J* = 7.0 Hz, 2H, OCH_2_), 6.11 (s, 2H, NCH_2_), 6.88–6.99 (m, 2H, C(O)Ph 3,5-H), 7.20–7.43 (m, 10H, 3-Ph 2,3,4,5,6-H; 4-Ph 2,3,4,5,6-H), 7.94–8.01 (m, 2H, C(O)Ph 2,6-H), 10.56 (s, 1H, OH). ^13^C NMR (101 MHz, DMSO-d_6_) *δ*_C_ ppm: 13.2 (CH_3_), 58.9 (NCH_2_), 60.6 (OCH_2_), 115.5 (C(O)Ph C-3,5), 124.1 (C-4), 125.8 (C(O)Ph C-1), 127.42 (3-Ph C-2,6; 4-Ph C-4), 127.7 (3-Ph C-4), 128.0 (4-Ph C-2,6), 128.3 (3-Ph C-3,5), 130.3 (4-Ph C-3,5), 130.7 (C(O)Ph C-2,6), 131.5 (3-Ph C-1), 132.2 (C-5), 132.8 (4-Ph C-1), 147.6 (C-3), 159.2 (COO), 162.8 (C(O)Ph C-4), 190.8 (C=O). ^15^N NMR (40 MHz, DMSO-d_6_) *δ*_N_ ppm: −177.7 (N-1), −64.9 (N-2). HRMS (ESI) for C_26_H_22_N_2_NaO_4_ ([M + Na]^+^): calcd *m*/*z* 449.1472, found *m*/*z* 449.1469.

##### Ethyl 3,4-bis(4-methoxyphenyl)-1-(2-oxo-2-phenylethyl)-1*H*-pyrazole-5-carboxylate (6f)

White solid, yield 77% (181 mg). *R*_f_ = 0.40 (*n*-hexane/ethyl acetate 4/1, v/v), mp 62–63 °C. IR (KBr) *ν*_max_, cm^−1^: 2982, 2939, 2836, 1706 (C=O), 1450, 1248, 1176, 1093, 1032, 834. ^1^H NMR (400 MHz, CDCl_3_) *δ*_H_ ppm: 0.97 (t, *J* = 7.1 Hz, 3H, CH_3_), 3.77 (s, 3H, 3-Ph 4-OCH_3_), 3.84 (s, 3H, 4-Ph 4-OCH_3_), 4.05 (q, *J* = 7.1 Hz, 2H, OCH_2_), 6.11 (s, 2H, NCH_2_), 6.72–6.80 (m, 2H, 3-Ph 3,5-H), 6.84–6.94 (m, 2H, 4-Ph 3,5-H), 7.19–7.25 (m, 2H, 4-Ph 2,6-H), 7.30–7.37 (m, 2H, 3-Ph 2,6-H), 7.48–7.57 (m, 2H, C(O)Ph 3,5-H), 7.60–7.68 (m, 1H, C(O)Ph 4-H), 7.99–8.07 (m, 2H, C(O)Ph 2,6-H). ^13^C NMR (101 MHz, CDCl_3_) *δ*_C_ ppm: 13.7 (CH_3_), 55.3 (3-Ph 4-OCH_3_), 55.4 (4-Ph 4-OCH_3_), 59.4 (NCH_2_), 61.0 (OCH_2_), 113.4 (4-Ph C-3,5), 113.7 (3-Ph C-3,5), 124.5 (C-4), 125.1 (3-Ph C-1), 125.4 (4-Ph C-1), 128.1 (C(O)Ph C-2,6), 129.1 (C(O)Ph C-3,5), 129.4 (3-Ph C-2,6), 131.8 (C-5), 132.0 (4-Ph C-2,6), 134.0 (C(O)Ph C-4), 134.9 (C(O)Ph C-1), 149.4 (C-3), 159.0 (3-Ph C-4), 159.3 (4-Ph C-4), 160.5 (COO), 192.5 (C=O). ^15^N NMR (40 MHz, CDCl_3_) *δ*_N_ ppm: −183.6 (N-1), −71.6 (N-2). HRMS (ESI) for C_28_H_26_N_2_NaO_5_ ([M + Na]^+^): calcd *m*/*z* 493.1734, found *m*/*z* 493.1732.

##### Ethyl 3,4-bis(4-methoxyphenyl)-1-[2-(4-methoxyphenyl)-2-oxoethyl]-1*H*-pyrazole-5-carboxylate (6g)

White solid, yield 91% (228 mg). *R*_f_ = 0.29 (*n*-hexane/ethyl acetate 4/1, v/v), mp 67–68 °C. IR (KBr) *ν*_max_, cm^−1^: 2938, 2837, 1707 (C=O), 1699, 1601, 1320, 1245, 1174, 1092, 1031, 835. ^1^H NMR (400 MHz, CDCl_3_) *δ*_H_ ppm: 0.96 (t, *J* = 7.1 Hz, 3H, CH_3_), 3.76 (s, 3H, 4-Ph 4-OCH_3_), 3.84 (s, 3H, 3-Ph 4-OCH_3_), 3.90 (s, 3H, C(O)Ph 4-OCH_3_), 4.05 (q, *J* = 7.1 Hz, 2H, OCH_2_), 6.05 (s, 2H, NCH_2_), 6.73–6.79 (m, 2H, 3-Ph 3,5-H), 6.85–6.91 (m, 2H, 4-Ph 3,5-H), 6.97–7.02 (m, 2H, C(O)Ph 3,5-H), 7.19–7.25 (m, 2H, 4-Ph 2,6-H), 7.30–7.36 (m, 2H, 3-Ph 2,6-H), 7.98–8.03 (m, 2H, C(O)Ph 2,6-H). ^13^C NMR (101 MHz, CDCl_3_) *δ*_C_ ppm: 13.6 (CH_3_), 55.16 (3-Ph 4-OCH_3_), 55.23 (4-Ph 4-OCH_3_), 55.6 (C(O)Ph 4-OCH_3_), 58.7 (NCH_2_), 60.8 (OCH_2_), 113.2 (4-Ph C-3,5), 113.6 (3-Ph C-3,5), 114.1 (C(O)Ph C-3,5), 124.3 (C-4), 125.2 (3-Ph C-1), 125.4 (4-Ph C-1), 127.8 (C(O)Ph C-1), 129.2 (3-Ph C-2,6), 130.3 (C(O)Ph C-2,6), 131.6 (C-5), 131.9 (4-Ph C-2,6), 149.2 (C-3), 158.8 (3-Ph C-4), 159.1 (4-Ph C-4), 160.4 (COO), 164.1 (C(O)Ph C-4), 190.8 (C=O). ^15^N NMR (40 MHz, CDCl_3_) *δ*_N_ ppm: −182.9 (N-1), −69.1 (N-2). HRMS (ESI) for C_29_H_28_N_2_NaO_6_ ([M + Na]^+^): calcd *m*/*z* 523.1840, found *m*/*z* 523.1840.

##### Ethyl 1-[2-(4-fluorophenyl)-2-oxoethyl]-3,4-bis(4-methoxy-phenyl)-1*H*-pyrazole-5-carboxylate (6h)

White solid, yield 66% (161 mg). *R*_f_ = 0.67 (*n*-hexane/ethyl acetate 3/2, v/v), mp 104–105 °C. IR (KBr) *ν*_max_, cm^−1^: 2998, 2940, 2836, 1705 (C=O), 1597, 1436, 1249, 1178, 1091, 1033, 839. ^1^H NMR (400 MHz, DMSO-d_6_) *δ*_H_ ppm: 0.84 (t, *J* = 7.1 Hz, 3H, CH_3_), 3.71 (s, 3H, 3-Ph 4-OCH_3_), 3.78 (s, 3H, 4-Ph 4-OCH_3_), 3.96 (q, *J* = 7.1 Hz, 2H, OCH_2_), 6.17 (s, 2H, NCH_2_), 6.80–6.87 (m, 2H, 3-Ph 3,5-H), 6.91–6.97 (m, 2H, 4-Ph 3,5-H), 7.13–7.19 (m, 2H, 4-Ph 2,6-H), 7.23–7.30 (m, 2H, 3-Ph 2,6-H), 7.40–7.48 (m, 2H, C(O)Ph 3,5-H), 8.15–8.22 (m, 2H, C(O)Ph 2,6-H). ^13^C NMR (101 MHz, DMSO-d_6_) *δ*_C_ ppm: 13.4 (CH_3_), 55.1 (2 × OCH_3_), 59.2 (NCH_2_), 60.6 (OCH_2_), 113.5 (4-Ph C-3,5), 113.8 (3-Ph C-3,5), 116.14 (d, ^2^*J*_CF_ = 22.0 Hz, C(O)Ph C-3,5), 123.5 (C-4), 124.71 (3-Ph C-1), 124.73 (4-Ph C-1), 128.7 (3-Ph C-2,6), 131.16, 131.20 and 131.26 (m, C-5, C(O)Ph C-1,2,6), 131.5 (4-Ph C-2,6), 147.9 (C-3), 158.6 (4-Ph 4-OCH_3_), 158.9 (3-Ph 4-OCH_3_), 159.4 (COO), 165.56 (d, *J*_CF_ = 252.9 Hz, C(O)Ph C-4), 191.9 (C=O). ^15^N NMR (40 MHz, DMSO-d_6_) *δ*_N_ ppm: −180.0 (N-1), −66.2 (N-1). ^19^F NMR (376 MHz, DMSO-d_6_) *δ*_F_ ppm: −104.5 (C(O)Ph 4-F). HRMS (ESI) for C_28_H_25_FN_2_NaO_5_ ([M + Na]^+^): calcd *m*/*z* 511.1640, found *m*/*z* 511.1639.

##### Ethyl 1-[2-(4-chlorophenyl)-2-oxoethyl]-3,4-bis(4-methoxy-phenyl)-1*H*-pyrazole-5-carboxylate (6i)

White solid, yield 82% (207 mg). *R*_f_ = 0.35 (*n*-hexane/ethyl acetate 4/1, v/v), mp 132–133 °C. IR (KBr) *ν*_max_, cm^−1^: 2982, 2939, 2836, 1706 (C=O), 1450, 1248, 1176, 1092, 1032, 834. ^1^H NMR (400 MHz, CDCl_3_) *δ*_H_ ppm: 0.96 (t, *J* = 7.1 Hz, 3H, CH_3_), 3.76 (s, 3H, 3-Ph 4-OCH_3_), 3.84 (s, 3H, 4-Ph 4-OCH_3_), 4.04 (q, *J* = 7.1 Hz, 2H, OCH_2_), 6.04 (s, 2H, NCH_2_), 6.72–6.81 (m, 2H, 3-Ph 3,5-H), 6.84–6.93 (m, 2H, 4-Ph 3,5-H), 7.17–7.24 (m, 2H, 4-Ph 2,6-H), 7.29–7.37 (m, 2H, 3-Ph 2,6-H), 7.45–7.53 (m, 2H, C(O)Ph 3,5-H), 7.90–8.00 (m, 2H, C(O)Ph 2,6-H). ^13^C NMR (101 MHz, CDCl_3_) *δ*_C_ ppm: 13.6 (CH_3_), 55.16 (3-Ph 4-OCH_3_), 55.22 (4-Ph 4-OCH_3_), 58.9 (NCH_2_), 60.9 (OCH_2_), 113.3 (4-Ph C-3,5), 113.6 (3-Ph C-3,5), 124.4 (C-4), 125.0 (3-Ph C-1), 125.3 (4-Ph C-1), 129.2 (3-Ph C-2,6), 129.3 (C(O)Ph C-3,5), 129.4 (C(O)Ph C-2,6), 131.4 (C-5), 131.8 (4-Ph C-2,6), 133.1 (C(O)Ph C-1), 140.4 (C(O)Ph C-4), 149.4 (C-3), 158.9 (4-Ph C-4), 159.17 (3-Ph C-4), 160.4 (COO), 191.4 (C=O). ^15^N NMR (40 MHz, CDCl_3_) *δ*_N_ ppm: −183.5 (N-1), −69.1 (N-2). HRMS (ESI) for C_28_H_25_ClN_2_NaO_5_ ([M + Na]^+^): calcd *m*/*z* 527.1344, found *m*/*z* 527.1346.

##### Ethyl 3-(4-fluorophenyl)-1-(2-oxo-2-phenylethyl)-4-phenyl-1*H*-pyrazole-5-carboxylate (6j)

White solid, yield 73% (156 mg). *R*_f_ = 0.74 (*n*-hexane/ethyl acetate 7/3, v/v), mp 135–136 °C. IR (KBr) *ν*_max_, cm^−1^: 3063, 2978, 2955, 1716 (C=O), 1523, 1449, 1305, 1222, 1096, 846, 768. ^1^H NMR (400 MHz, DMSO-d_6_) *δ*_H_ ppm: 0.80 (t, *J* = 7.1 Hz, 3H, CH_3_), 3.95 (q, *J* = 7.0 Hz, 2H, OCH_2_), 6.22 (s, 2H, NCH_2_), 7.06–7.18 (m, 2H, 3-Ph 3,5-H), 7.22–7.45 (m, 7H, 3-Ph 2,6-H; 4-Ph 2,3,4,5,6-H), 7.58–7.67 (m, 2H, C(O)Ph 3,5-H), 7.70–7.79 (m, 1H, C(O)Ph 4-H), 8.05–8.16 (m, 2H, C(O)Ph 2,6-H). ^13^C NMR (101 MHz, DMSO-d_6_) *δ*_C_ ppm: 13.2 (CH_3_), 59.3 (NCH_2_), 60.7 (OCH_2_), 115.29 (d, ^2^*J*_CF_ = 21.5 Hz, 3-Ph C-3,5), 124.0 (C-4), 127.5 (4-Ph C-4), 128.1 (4-Ph C-3,5; C(O)Ph C-2,6), 128.62 (d, ^4^*J*_CF_ = 3.1 Hz, 3-Ph C-4), 129.0 (C(O)Ph C-3,5), 129.43 (d, ^3^*J*_CF_ = 8.2 Hz, 3-Ph C-2,6), 130.3 (4-Ph C-2,6), 131.4 (C-5), 132.5 (4-Ph C-1), 134.1 (C(O)Ph C-4), 134.3 (C(O)Ph C-1), 146.9 (C-3), 159.2 (COO), 161.72 (d, *J*_CF_ = 245.2 Hz, 3-Ph C-4), 193.0 (C=O). ^15^N NMR (40 MHz, DMSO-d_6_) *δ*_N_ ppm: −178.1 (N-1), −65.2 (N-2). ^19^F NMR (376 MHz, DMSO-d_6_) *δ*_F_ ppm: −114.0 (3-Ph 4-F). HRMS (ESI) for C_26_H_21_FN_2_NaO_3_ ([M + Na]^+^): calcd *m*/*z* 451.1428, found *m*/*z* 451.1427.

##### Ethyl 3,4-bis(4-fluorophenyl)-1-(2-oxo-2-phenylethyl)-1*H*-pyrazole-5-carboxylate (6k)

White solid, yield 79% (176 mg). *R*_f_ = 0.71 (*n*-hexane/ethyl acetate 7/3, v/v), mp 103–104 °C. IR (KBr) *ν*_max_, cm^−1^: 3063, 2976, 2949, 1704 (C=O), 1524, 1442, 1223, 1095, 856, 602. ^1^H NMR (400 MHz, DMSO-d_6_) *δ*_H_ ppm: 0.84 (t, *J* = 7.0 Hz, 3H, CH_3_), 3.97 (q, *J* = 7.0 Hz, 2H, OCH_2_), 6.22 (s, 2H, NCH_2_), 7.10–7.38 (m, 8H, 3-Ph 2,3,5,6-H; 4-Ph 2,3,5,6-H), 7.58–7.66 (m, 2H, C(O)Ph 3,5-H), 7.71–7.78 (m, 1H, C(O)Ph 4-H), 8.06–8.13 (m, 2H, C(O)Ph 2,6-H). ^13^C NMR (101 MHz, DMSO-d_6_) *δ*_C_ ppm: 13.2 (CH_3_), 59.4 (NCH_2_), 60.7 (NCH_2_), 115.01 (d, ^2^*J*_CF_ = 21.4 Hz, 4-Ph C-3,5), 115.38 (d, ^2^*J*_CF_ = 21.6 Hz, 3-Ph C-3,5), 123.0 (C-4), 128.1 (C(O)Ph C-2,6), 128.47 (d, ^4^*J*_CF_ = 3.2 Hz, 3-Ph C-1), 128.77 (d, ^4^*J*_CF_ = 3.2 Hz, 4-Ph C-1), 129.0 (C(O)Ph C-3,5), 129.50 (d, ^3^*J*_CF_ = 8.3 Hz, 3-Ph C-2,6), 131.5 (C-5), 132.40 (d, ^3^*J*_CF_ = 8.2 Hz, 4-Ph C-2,6) 134.1 (C(O)Ph C-1), 134.3 (C(O)Ph C-4), 147.1 (C-3), 159.1 (COO), 161.67 (d, *J*_CF_ = 244.1 Hz, 4-Ph C-4), 161.76 (d, *J*_CF_ = 245.2 Hz, 3-Ph C-4), 193.0 (C=O). ^15^N NMR (40 MHz, DMSO-d_6_) *δ*_N_ ppm: −177.8 (N-1), −65.1 (N-2). ^19^F NMR (376 MHz, DMSO-d_6_) *δ*_F_ ppm: −114.7 (4-F), −113.9 (4-F). HRMS (ESI) for C_26_H_20_F_2_N_2_NaO_3_ ([M + Na]^+^): calcd *m*/*z* 469.1334, found *m*/*z* 469.1334.

##### Ethyl 3-(4-fluorophenyl)-4-(4-methoxyphenyl)-1-(2-oxo-2-phenylethyl)-1*H*-pyrazole-5-carboxylate (6l)

White solid, yield 80% (183 mg). *R*_f_ = 0.68 (*n*-hexane/ethyl acetate 3/2, v/v), mp 87–88 °C. IR (KBr) *ν*_max_, cm^−1^: 2984, 2941, 2837, 1724, 1702 (C=O), 1440, 1223, 1176, 1087, 832. ^1^H NMR (400 MHz, CDCl_3_) *δ*_H_ ppm: 0.97 (t, *J* = 7.1 Hz, 3H, CH_3_), 3.84 (s, 3H, OCH_3_), 4.06 (q, *J* = 7.1 Hz, 2H, OCH_2_), 6.11 (s, 2H, NCH_2_), 6.86–6.96 (m, 4H, 3-Ph 3,5-H; 4-Ph 3,5-H), 7.18–7.24 (m, 2H, 4-Ph 2,6-H), 7.35–7.41 (m, 2H, 3-Ph 2,6-H), 7.50–7.57 (m, 2H, C(O)Ph 3,5-H), 7.61–7.68 (m, 1H, C(O)Ph 4-H), 7.98–8.08 (m, 2H, C(O)Ph 2,6-H). ^13^C NMR (101 MHz, CDCl_3_) *δ*_C_ ppm: 13.7 (CH_3_), 55.4 (OCH_3_), 59.2 (NCH_2_), 61.1 (OCH_2_), 113.5 (4-*Pc* C-3,5), 115.26 (d, ^2^*J*_CF_ = 21.5 Hz, 3-Ph C-3,5), 124.7 (C-4), 125.0 (4-Ph C-1), 128.1 (C(O)Ph C-2,6), 128.67 (d, ^4^*J*_CF_ = 3.2 Hz, 3-Ph C-1), 129.1 (C(O)Ph C-3,5), 129.88 (d, ^3^*J*_CF_ = 8.1 Hz, 3-Ph C-2,6), 131.88 (C-5), 131.91 (4-Ph C-2,6), 134.1 (C(O)Ph C-4), 134.8 (C(O)Ph C-1), 148.7 (C-3), 159.1 (4-Ph C-4), 160.4 (COO), 162.55 (d, *J*_CF_ = 247.1 Hz, 3-Ph C-4), 192.4 (C=O). ^15^N NMR (40 MHz, CDCl_3_) *δ*_N_ ppm: −182.2 (N-1), −70.0 (N-2). ^19^F NMR (376 MHz, CDCl_3_) *δ*_F_ ppm: −114.2 (3-Ph 4-F). HRMS (ESI) for C_27_H_23_FN_2_NaO_4_ ([M + Na]^+^): calcd *m*/*z* 481.1534, found *m*/*z* 481.1535.

##### Ethyl 3-(4-fluorophenyl)-1-[2-(4-fluorophenyl)-2-oxoethyl]-4-phenyl-1*H*-pyrazole-5-carboxylate (6m)

White solid, yield 76% (170 mg). *R*_f_ = 0.64 (*n*-hexane/ethyl acetate 3/2, v/v), mp 104–105 °C. IR (KBr) *ν*_max_, cm^−1^: 3059, 2999, 2962, 1711 (C=O), 1599, 1232, 1156, 1095, 840. ^1^H NMR (400 MHz, DMSO-d_6_) *δ*_H_ ppm: 0.80 (t, *J* = 7.1 Hz, 3H, CH_3_), 3.95 (q, *J* = 7.1 Hz, 2H, OCH_2_), 6.21 (s, 2H, NCH_2_), 7.07–7.17 (m, 2H, 3-Ph 3,5-H), 7.21–7.51 (m, 9H, 3-Ph 2,6-H; 4-Ph 2,3,4,5,6-H; C(O)Ph 3,5-H), 8.16–8.22 (m, 2H, C(O)Ph 2,6-H). ^13^C NMR (101 MHz, DMSO-d_6_) *δ*_C_ ppm: 13.2 (CH_3_), 59.3 (NCH_2_), 60.7 (OCH_2_), 115.29 (d, ^2^*J*_CF_ = 21.6 Hz, 3-Ph C-3,5), 116.12 (d, ^2^*J*_CF_ = 22.0 Hz, C(O)Ph C-3,5), 124.0 (C-4), 127.5 (4-Ph C-4), 128.1 (4-Ph C-3,5), 128.60 (d, ^4^*J*_CF_ = 3.1 Hz, 3-Ph C-1), 129.42 (d, ^4^*J*_CF_ = 8.3 Hz, 3-Ph C-2,6), 130.3 (4-Ph C-2,6), 131.10 (d, ^4^*J*_CF_ = 2.8 Hz, C(O)Ph C-1), 131.21 (d, ^3^*J*_CF_ = 9.6 Hz, C(O)Ph C-2,6), 131.4 (C-5), 132.5 (4-Ph C-1), 147.0 (C-3), 159.2 (COO), 161.72 (d, *J*_CF_ = 245.1 Hz, 3-Ph C-4), 165.54 (d, *J*_CF_ = 252.9 Hz, C(O)Ph C-4), 191.7 (C=O). ^15^N NMR (40 MHz, DMSO-d_6_) *δ*_N_ ppm: −177.7 (N-1), −65.2 (N-2). ^19^F NMR (376 MHz, DMSO-d_6_) *δ*_F_ ppm: −114.0 (4-F), −104.4 (4-F). HRMS (ESI) for C_26_H_20_F_2_N_2_NaO_3_ ([M + Na]^+^): calcd *m*/*z* 469.1334, found *m*/*z* 469.1332.

##### Ethyl 3-(4-fluorophenyl)-1-[2-(4-fluorophenyl)-2-oxoethyl]-4-(4-methoxyphenyl)-1*H*-pyrazole-5-carboxylate (6n)

White solid, yield 79% (188 mg). *R*_f_ = 0.69 (*n*-hexane/ethyl acetate x/x, v/v), mp 81–82 °C. IR (KBr) *ν*_max_, cm^−1^: 2993, 2956, 2840, 1706 (C=O), 1597, 1440, 1232, 1093, 844. ^1^H NMR (400 MHz, CDCl_3_) *δ*_H_ ppm: 0.97 (t, *J* = 7.1 Hz, 3H, CH_3_), 3.85 (s, 3H, OCH_3_), 4.05 (q, *J* = 7.1 Hz, 2H, OCH_2_), 6.07 (s, 2H, NCH_2_), 6.86–6.96 (m, 4H, 4-Ph 3,5-H; C(O)Ph 3,5-H), 7.16–7.24 (m, 4H, 3-Ph 3,5-H; 4-Ph 2,6-H), 7.33–7.42 (m, 2H, 3-Ph 2,6-H), 8.01–8.11 (m, 2H, C(O)Ph 2,6-H). ^13^C NMR (101 MHz, CDCl_3_) *δ*_C_ ppm: 13.6 (CH_3_), 55.3 (OCH_3_), 58.9 (NCH_2_), 61.0 (OCH_2_), 113.4 (4-Ph C-3,5), 115.16 (d, ^2^*J*_CF_ = 21.5 Hz, C(O)Ph C-3,5), 116.23 (d, ^2^*J*_CF_ = 22.0 Hz, 3-Ph C-3,5), 124.6 (C-4), 124.9 (4-Ph C-1), 128.51 (d, ^4^*J*_CF_ = 3.3 Hz, 3-Ph C-1), 129.75 (d, ^3^*J*_CF_ = 8.1 Hz, 3-Ph C-2,6), 130.73 (d, ^3^*J*_CF_ = 9.5 Hz, C(O)Ph C-2,6), 131.15 (d, ^4^*J*_CF_ = 3.1 Hz, C(O)Ph C-1), 131.7 (C-5), 131.8 (4-Ph C-2,6), 148.7 (C-3), 159.0 (4-Ph C-4), 160.3 (COO), 162.45 (d, *J*_CF_ = 247.1 Hz, 3-Ph C-4), 166.24 (d, *J*_CF_ = 256.1 Hz, C(O)Ph C-4), 190.8 (C=O). ^15^N NMR (40 MHz, CDCl_3_) *δ*_N_ ppm: −182.8 (N-1), −69.2 (N-2). ^19^F NMR (376 MHz, CDCl_3_) *δ*_F_ ppm: −114.2 (4-F), −103.3 (4-F). HRMS (ESI) for C_27_H_22_F_2_N_2_NaO_4_ ([M + Na]^+^): calcd *m*/*z* 499.1440, found *m*/*z* 499.1439.

##### Ethyl 3,4-bis(4-fluorophenyl)-1-[2-(4-fluorophenyl)-2-oxoethyl]-1*H*-pyrazole-5-carboxylate (6o)

White solid, yield 85% (197 mg). *R*_f_ = 0.62 (*n*-hexane/ethyl acetate 7/3, v/v), mp 105–106 °C. IR (KBr) *ν*_max_, cm^−1^: 3000, 2984, 2946, 1710 (C=O), 1599, 1444, 1231, 840. ^1^H NMR (400 MHz, DMSO-d_6_) *δ*_H_ ppm: 0.84 (t, *J* = 7.1 Hz, 3H, CH_3_), 3.97 (q, *J* = 7.0 Hz, 2H, OCH_2_), 6.22 (s, 2H, NCH_2_), 7.09–7.38 (m, 8H, 3-Ph 2,3,5,6-H; 3-Ph 2,3,5,6-H), 7.40–7.51 (m, 2H, C(O)Ph 3,5-H), 8.12–8.25 (m, 2H, C(O)Ph 2,6-H). ^13^C NMR (101 MHz, DMSO-d_6_) *δ*_C_ ppm: 13.2 (CH_3_), 59.3 (NCH_2_), 60.8 (OCH_2_), 115.02 (d, ^2^*J*_CF_ = 21.4 Hz, 4-Ph C-3,5), 115.39 (d, ^2^*J*_CF_ = 21.5 Hz, 3-Ph C-3,5), 116.13 (d, ^2^*J*_CF_ = 22.0 Hz, C(O)Ph C-3,5), 123.0 (C-4), 128.45 (d, ^4^*J*_CF_ = 3.2 Hz, 3-Ph C-1), 128.75 (d, ^4^*J*_CF_ = 3.3 Hz, 4-Ph C-1), 129.50 (d, ^3^*J*_CF_ = 8.3 Hz, 3-Ph C-2,6), 131.09 (d, ^4^*J*_CF_ = 2.7 Hz, C(O)Ph C-1), 131.21 (d, ^3^*J*_CF_ = 9.6 Hz, C(O)Ph C-2,6), 131.5 (C-5), 132.40 (d, ^3^*J*_CF_ = 8.3 Hz, 4-Ph C-2,6), 147.1 (C-3), 159.1 (COO), 161.67 (d, *J*_CF_ = 244.1 Hz, 4-Ph C-4), 161.77 (d, *J*_CF_ = 245.3 Hz, 3-Ph C-4), 165.55 (d, *J*_CF_ = 253.0 Hz, C(O)Ph C-4), 191.69 (C=O). ^15^N NMR (40 MHz, DMSO-d_6_) *δ*_N_ ppm: −177.7 (N-1), −65.1 (N-2). ^19^F NMR (376 MHz, DMSO-d_6_) *δ*_F_ ppm: −114.7 (4-F), −113.9 (4-F), −104.4 (4-F). HRMS (ESI) for C_26_H_19_F_3_N_2_NaO_3_ ([M + Na]^+^): calcd *m*/*z* 487.1240, found *m*/*z* 487.1238.

#### General procedure for synthesis of methyl 3,4-diaryl-1-(2-aryl-2-oxoethyl)-1*H*-pyrazole-5-carboxylates (7a–c)

An appropriate pyrazole derivative 6a–c (0.25 mmol) was dissolved in MeOH (0.05 M), K_2_CO_3_ (0.125 mmol) was added, and mixture was refluxed for 3 h. After full conversion solvent was partially evaporated, residue was diluted with EtOAc and washed with water and brine. Organic phase was separated, dried over anhydrous Na_2_SO_4_, filtered off and concentrated under reduced pressure. Crude was purified by column chromatography (Hex/EtOAc 20/1, v/v) to yield products 7a–c.

##### Methyl 1-(2-oxo-2-phenylethyl)-3,4-diphenyl-1*H*-pyrazole-5-carboxylate (7a)

White solid, yield 94% (93 mg). *R*_f_ = 0.63 (*n*-hexane/ethyl acetate 7/3, v/v), mp 138–139 °C. IR (KBr) *ν*_max_, cm^−1^: 3059, 2976, 2939, 1716 (C=O), 1451, 1355, 1319, 1231, 1100, 774 and 757 (doublet), 705 and 691 (doublet). ^1^H NMR (400 MHz, acetone-d_6_) *δ*_H_ ppm: 3.53 (s, 3H, CH_3_), 6.24 (s, 2H, CH_2_), 7.19–7.45 (m, 10H, 3-Ph 2,3,4,5,6-H; 4-Ph 2,3,4,5,6-H), 7.59–7.68 (m, 2H, C(O)Ph 3,5-H), 7.70–7.78 (m, 1H, C(O)Ph 4-H), 8.12–8.19 (m, 2H, C(O)Ph 2,6-H). ^13^C NMR (101 MHz, acetone-d_6_) *δ*_C_ ppm: 52.0 (NCH_2_), 60.1 (OCH_2_), 125.4 (C-4), 128.2 (4-Ph C-4), 128.5 (3-Ph C-4), 128.7 (4-Ph C-2,6), 128.8 (3-Ph 2,6-H), 128.9 (3-Ph 3,5-H; C(O)Ph C-2,6), 129.8 (C(O)Ph C-3,5), 131.4 (4-Ph C-3,5), 132.4 (C-5), 133.7 (3-Ph C-1), 134.1 (4-Ph C-1), 134.8 (C(O)Ph C-4), 135.9 (C(O)Ph C-1), 149.5 (C-3), 161.2 (COO), 193.4 (C=O). ^15^N NMR (40 MHz, acetone-d_6_) *δ*_N_ ppm: −178.7 (N-1), −63.3 (N-2). HRMS (ESI) for C_25_H_20_N_2_NaO_3_ ([M + Na]^+^): calcd *m*/*z* 419.1366, found *m*/*z* 419.1367.

##### Methyl 1-[2-(4-methoxyphenyl)-2-oxoethyl]-3,4-diphenyl-1*H*-pyrazole-5-carboxylate (7b)

White solid, yield 92% (98 mg). *R*_f_ = 0.49 (*n*-hexane/ethyl acetate 7/3, v/v), mp 163–164 °C. IR (KBr) *ν*_max_, cm^−1^: 3025, 2962, 2836, 1712 (C=O), 1601, 1237, 1176, 1096, 698. ^1^H NMR (400 MHz, acetone-d_6_) *δ*_H_ ppm: 3.53 (s, 3H, COOCH_3_), 3.94 (s, 3H, OCH_3_), 6.17 (s, 2H, CH_2_), 7.09–7.17 (m, 2H, C(O)Ph 3,5-H), 7.20–7.43 (m, 10H, 3-Ph 2,3,4,5,6-H; 4-Ph 2,3,4,5,6-H), 8.09–8.17 (m, 2H, C(O)Ph 2,6-H). ^13^C NMR (101 MHz, acetone-d_6_) *δ*_C_ ppm: 51.9 (COOC̲H_3_), 56.1 (OCH_3_), 59.8 (CH_2_), 115.0 (C(O)Ph C-3,5), 125.3 (C-4), 128.2 (4-Ph C-4), 128.4 (3-Ph C-4), 128.68 (4-Ph C-2,6), 128.72 (C(O)Ph C-1), 128.8 (3-Ph C-2,6), 128.9 (3-Ph C-3,5), 131.2 (C(O)Ph C-2,6), 131.4 (4-Ph C-3,5), 132.5 (C-5), 133.7 (3-Ph C-1), 134.2 (4-Ph C-1), 149.4 (C-3), 161.2 (COO), 165.2 (C(O)Ph C-4), 191.5 (C=O). ^15^N NMR (40 MHz, acetone-d_6_) *δ*_N_ ppm: −178.2 (N-1), −63.5 (N-2). HRMS (ESI) for C_26_H_22_N_2_NaO_4_ ([M + Na]^+^): calcd *m*/*z* 449.1472, found *m*/*z* 449.1470.

##### Methyl 1-[2-(4-fluorophenyl)-2-oxoethyl]-3,4-diphenyl-1*H*-pyrazole-5-carboxylate (7c)

White solid, yield 96% (100 mg). *R*_f_ = 0.66 (*n*-hexane/ethyl acetate 7/3, v/v), mp 114–115 °C. IR (KBr) *ν*_max_, cm^−1^: 3061, 2929, 1720 (C=O), 1596, 1232, 1086, 841, 700. ^1^H NMR (400 MHz, acetone-d_6_) *δ*_H_ ppm: 3.56 (s, 3H, CH_3_), 6.23 (s, 2H, CH_2_), 7.13–7.20 (m, 2H, C(O)Ph 3,5-H), 7.23–7.29 (m, 3H, 3-Ph 3,4,5-H), 7.31–7.41 (m, 4H, 3-Ph 2,6-H; 4-Ph 2,6-H), 7.59–7.66 (m, 2H, 4-Ph 3, 5H), 7.70–7.77 (m, 1H, 4-Ph 4-H), 8.12–8.18 (m, 2H, C(O)Ph 2,6-H). ^13^C NMR (101 MHz, acetone-d_6_) *δ*_C_ ppm: 52.0 (CH_3_), 60.2 (CH_2_), 115.64 (d, ^2^*J*_CF_ = 21.6 Hz, C(O)Ph C-3,5), 124.3 (C-4), 128.6 (4-Ph C-4), 128.7 (3-Ph C-4), 128.9 (3-Ph C-3,5), 129.0 (4-Ph C-2,6), 129.8 (3-Ph C-2,6), 130.29 (d, ^4^*J*_CF_ = 3.4 Hz, C(O)Ph C-1), 132.4 (4-Ph C-3,5), 133.39 (d, ^3^*J*_CF_ = 8.2 Hz, C(O)Ph C-2,6), 133.5 (3-Ph C-1), 134.8 (4-Ph C-1), 135.9 (C-5), 149.6 (C-3), 161.1 (COO), 163.13 (d, *J*_CF_ = 244.5 Hz, C(O)Ph C-4), 193.3 (C=O). ^15^N NMR (40 MHz, acetone-d_6_) *δ*_N_ ppm: −178.3 (N-1), −63.2 (N-2). ^19^F NMR (376 MHz, acetone-d_6_) *δ*_F_ ppm: −116.5 (C(O)Ph 4-F). HRMS (ESI) for C_25_H_19_FN_2_NaO_3_ ([M + Na]^+^): calcd *m*/*z* 437.1272, found *m*/*z* 437.1269.

### Determination of HPLC–log *P*

HPLC–log *P* values were determined using Shimadzu HPLC system equipped with apHera C18 column (10 × 6 mm, 5 μm, Supelco, Bellefonte, PA, USA). Each sample was dissolved in the internal standard mixture, consisting of triphenylene (99.9%, Carl Roth) and toluene (≥98%, Sigma-Aldrich). Analysis was performed at 1.5 mL min^−1^ flow rate in the linear gradient where mobile phase consisted of methanol and 0.01 M phosphate buffer (pH 7.4). The HPLC–log *P* values were calculated from the measured retention times using previously published equation.^57^

### Plasma protein binding (PPB)

Retention times of the analytes were measured with Shimadzu HPLC system on the CHIRALPAK®HAS stationary phase (50 × 3 mm, 5 μm, Chiral Technologies, DAICEL Group, Europe SAS, France). The mobile phase A consisted of 50 mM aqueous ammonium acetate buffer (pH 7.4) and phase B of 2-propanol according to Valko *et al.*^65^ Analysis was performed at prolonged 1 mL min^−1^ flow rate in the linear gradient. Retention capacity factors (*k*′) were calculated by using DMSO or a substance with 0% HAS binding for systems' dead time (*R*_*t*_0__). The system was calibrated by injecting the reference compounds: acetylsalicylic acid (CAS 69-72-7), betamethasone (CAS 378-44-9), budesonide (CAS 5133-22-3), carbamazepine (CAS 298-46-4), cimetidine (CAS 51481-61-9), ciprofloxacin (CAS 85721-33-1), indomethacin (CAS 53-86-1), isoniazid (CAS 54-85-3), metronidazole (CAS 443-48-1), nicardipine (CAS 55985-32-5), nizatidine (CAS 76963-41-2) and warfarin (CAS 81-81-2) obtained from Sigma-Aldrich, diclofenac (CAS 15307-86-5) from EMD Chemicals Inc., flumazenil (CAS 78755-81-4) from ABX and ketoprofen (CAS 22071-15-4) from LKT Labs. The logarithmic capacity factors of the references' Rt (log(*k*′)) on the HSA column were plotted against the %PPB values from literature. The slope and the intercept were used to convert the log(*k*′) of the compounds (6a, c, f, h, m–o) to %PPB using the regression equation.^66^

### Biological evaluation

#### Cell cultures

SW480 (human colon adenocarcinoma, adherent, epithelial, ATCC number: CCL-228), HT29 (human colon adenocarcinoma, adherent, epithelial, ATCC number: HTB-38), and HCT116 (human colon adenocarcinoma, adherent, epithelial-like, ATCC number: CCL-247) cells were maintained in RPMI-1640 medium (Sigma-Aldrich). Medium was supplemented with heat-inactivated FBS (Biowest), penicillin–streptomycin (Sigma; with 10 000 units penicillin and 10 mg streptomycin per mL in 0.9% NaCl) and l-glutamine (Sigma; 200 mM solution). Cell cultures were cultivated at 37 °C, maintaining a humidified atmosphere consisting of 5% CO_2_. Gibco™ trypsin–EDTA (0.05%) was used for cell passage.

#### Cell viability (MTT assay)

To evaluate cell viability, SW480, HT29, and HCT116 cells were harvested from culture flasks by trypsinization and seeded into 96-well plates in 4000 cells per well, 3000 cells per well and 4000 cells per well densities, respectively. After 24 h preincubation period, cells were treated in triplicates with nine different doses of each compound (6a–o and 7a–c) for 96 h. After treatment compound solutions were replaced with MTT solution (MTT reagent in PBS, 5 mg L^−1^) diluted in a 6 : 1 ratio in non-supplemented RPMI-1640 medium and were additionally incubated for 2 h. After incubation, the medium was removed, and formazan product was dissolved in DMSO. Optical densities were measured at 550 nm with TECAN Infinite® M200 PRO microplate reader using a reference wavelength of 690 nm to correct unspecific absorption. The quantity of viable cells was normalized to untreated controls. The GI_50_ values were calculated from dose–response curves.

#### Viability staining *via* Calcein AM/Hoechst/PI

SW480, HT29 and HCT116 cells were seeded in 24-well plates in a cell density of 6000 cells per well and settled for 24 h. Afterwards cells were treated with 4 μM of test compounds 6a, c, f, h, m–o for 96 h. Cells were stained with propidium iodide (Sigma Aldrich, final concentration 2 μg mL^−1^) and Hoechst 33342 (ThermoFisher Scientific, final concentration 0.5 μM) for 60 min and with Calcein AM (Merck, BioReagent, final concentration 50 μM) for 15 min, washed two times with PBS and finally covered with PBS for further analysis. Fluorescence microscopic evaluation was performed on a Evos FL Cell Imaging System (ThermoFisher Scientific).

#### Fluorescence-activated cell sorting (FACS)

HCT116 cells (40 000 cells per well) were treated with the test compounds (6c, 6m) for 72 h in the concentration range of 5, 10 and 20 μM. After treatment cells were washed with DPBS (Sigma; modified, without calcium chloride and magnesium chloride) and trypsinized using a trypsin–EDTA solution (Gibco™, 0.05%). After additional washing with DPBS, cells were stained at +4 °C overnight using PI/HFS solution (50 μg mL^−1^).^67^ Samples were then analysed using Guava® easyCyte™ 8HT (Merck Millipore) flow cytometer with Guava Clean 3.1 software. The amount of debris was in the range of 9.2 ± 2.0%.

## Conclusions

Proceeding from the natural marine alkaloid lamellarin O, a scaffold hopping from a central pyrrole to pyrazole resulted in 18 fully characterized derivatives. Structure–activity relationships revealed the importance of a fluorine in the *para*-position of the phenyl substituent in phenyl-2-oxoethyl scaffold. The most cytotoxic compounds inhibited cell proliferation in the low micromolar range in three colorectal cancer cell lines, namely HCT116, HT29, and SW480. The similarity of the investigated GI_50_ values demonstrated the absence of conventional resistance mechanism between the cell lines. Pronounced effects on the cell cycle were observed resulting in G1 and predominantly G2/M phase arrest. This set of lamellarin O inspired pyrazole derivatives is an important entry for natural product-derived drug candidates.

## Author contributions

Conceptualization, E. A., A. Ž. and V.·P.; methodology, E. A., A. Ž. and V. P.; formal analysis, K. D., N. F. E. S.-B.; investigation, K. D., N. F., E. S.-B., V. P.; resources, A. Š., E. A. and V. P.; data curation, E. A. and V. P.; writing—original draft preparation, K. D.; writing—review and editing, E. A., A. Ž., A. Š. and V. P.; visualization, K. D. and V. P.; supervision, E. A., A. Ž. and V. P.; funding acquisition, V. P. and E. A. All authors have read and agreed to the published version of the manuscript.

## Conflicts of interest

There are no conflicts to declare.

## Supplementary Material

RA-013-D3RA00972F-s001
