# Supplementary material for: Pyrazole-based lamellarin O analogues: synthesis, biological evaluation and structure–activity relationships
Source: RSC Adv. 2023 Mar 10;13(12):7897–912. doi: 10.1039/d3ra00972f (PMC9999251; doi:10.1039/d3ra00972f)
Supplement: RA-013-D3RA00972F-s001 [file RA-013-D3RA00972F-s001.pdf]

Electronic Supplementary Material (ESI) for RSC Advances.
This journal is © The Royal Society of Chemistry 2023

SUPPORTING INFORMATION

Pyrazole-Based Lamellarin O Analogues: Synthesis, Biological Evaluation and Structure-Activity Relationships

Karolina Dzedulionytė[a], Nina Fuxreiter[b], Ekaterina Schreiber-Brynzak[b], Asta Žukauskaitė[c], Algirdas Šačkus[a,d], Verena Pichler[b,*], and Eglė Arbačiauskienė[a,*]

[a] *Department of Organic Chemistry, Faculty of Chemical Technology, Kaunas University of Technology, Radvilėnų pl. 19, LT-50254 Kaunas, Lithuania*

[b] *Department of Pharmaceutical Sciences, Division of Pharmaceutical Chemistry, Faculty of Life Sciences, University of Vienna, Althanstraße 14, 1090 Vienna, Austria*

[c] *Department of Chemical Biology, Faculty of Science, Palacký University, Šlechtitelů 27, CZ-78371 Olomouc, Czech Republic*

[d] *Institute of Synthetic Chemistry, Faculty of Chemical Technology, Kaunas University of Technology, K. Baršausko g. 59, LT-51423 Kaunas, Lithuania*

Table of Contents

1. Chemistry 2
2. Analytical data of intermediates **4a**–**k** and **5a** 3
3. Analytical data of target compounds **6a′**, **6a**–**o** and **7a**–**c** 27
4. Physicochemical parameters 70
5. Biological evaluation 71

1. Chemistry

Table S1. Optimisation of **3a** Suzuki cross-coupling reaction conditions with phenylboronic acid.

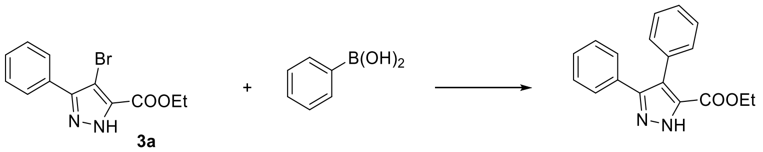

Entry	Conditions	Yield
**1**	$PhB(OH)_2$ (1.5 eq), $Pd(PPh_3)_4$ (0.05 eq), sat. N_2CO_3 solution, DMF, MW, 140 °C, 40 min	9%
**2**	$PhB(OH)_2$ (1.5 eq), $Pd(PPh_3)_4$ (0.05 eq), K_2CO_3 (3 eq), DMF, H_2O, MW, 140 °C, 60 min	11%
**3**	$PhB(OH)_2$ (1.5 eq), $Pd(PPh_3)_4$ (0.05 eq), Cs_2CO_3 (3 eq), DMF, H_2O, MW, 140 °C, 60 min	10%
**4**	$PhB(OH)_2$ (1.5 eq), $Pd(PPh_3)_4$ (0.05 eq), K_3PO_4 (3 eq), DMF, H_2O, MW, 140 °C, 60 min	22%
**5**	$PhB(OH)_2$ (1.5 eq), $Pd(PPh_3)_4$ (0.05 eq), K_3PO_4 (3 eq), DMF, MW, 140 °C, 120 min	4%
**6**	$PhB(OH)_2$ (1.5 eq), $Pd(PPh_3)_4$ (0.05 eq), K_3PO_4 (3 eq), DMF, H_2O, 100 °C, 24 h	2%
**7**	$PhB(OH)_2$ (1.5 eq), $Pd(PPh_3)_4$ (0.05 eq), K_3PO_4 (3 eq), Dioxane, H_2O, MW, 105 °C, 120 min	13%
**8**	$PhB(OH)_2$ (1.5 eq), $Pd(PPh_3)_4$ (0.05 eq), K_3PO_4 (3 eq), THF, H_2O, MW, 90 °C, 180 min	6%
**9**	$PhB(OH)_2$ (1.5 eq), $Pd(PPh_3)_4$ (0.05 eq), K_3PO_4 (3 eq), Dioxane, H_2O, 100 °C, 16 h	10%
**10**	$PhB(OH)_2$ (1.5 eq), $Pd(PPh_3)_4$ (0.05 eq), K_3PO_4 (3 eq), KBr (1.1 eq), Dioxane, reflux, 6 h	4%

2. Analytical data of intermediates 4a–k and 5a

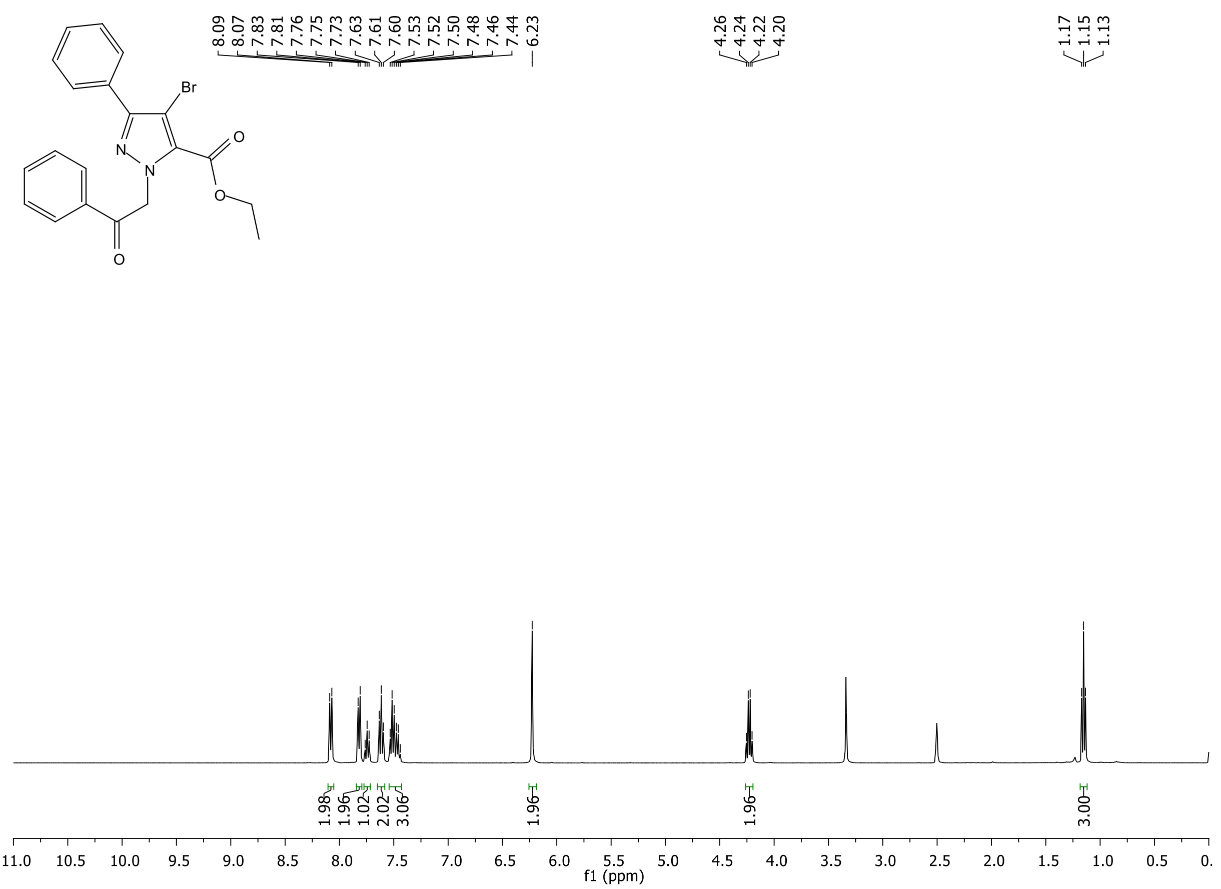

Figure S1. ^{1}H NMR spectrum (400 MHz, DMSO-d_6) of ethyl 4-bromo-1-(2-oxo-2-phenylethyl)-3-phenyl-1*H*-pyrazole-5-carboxylate (**4a**).

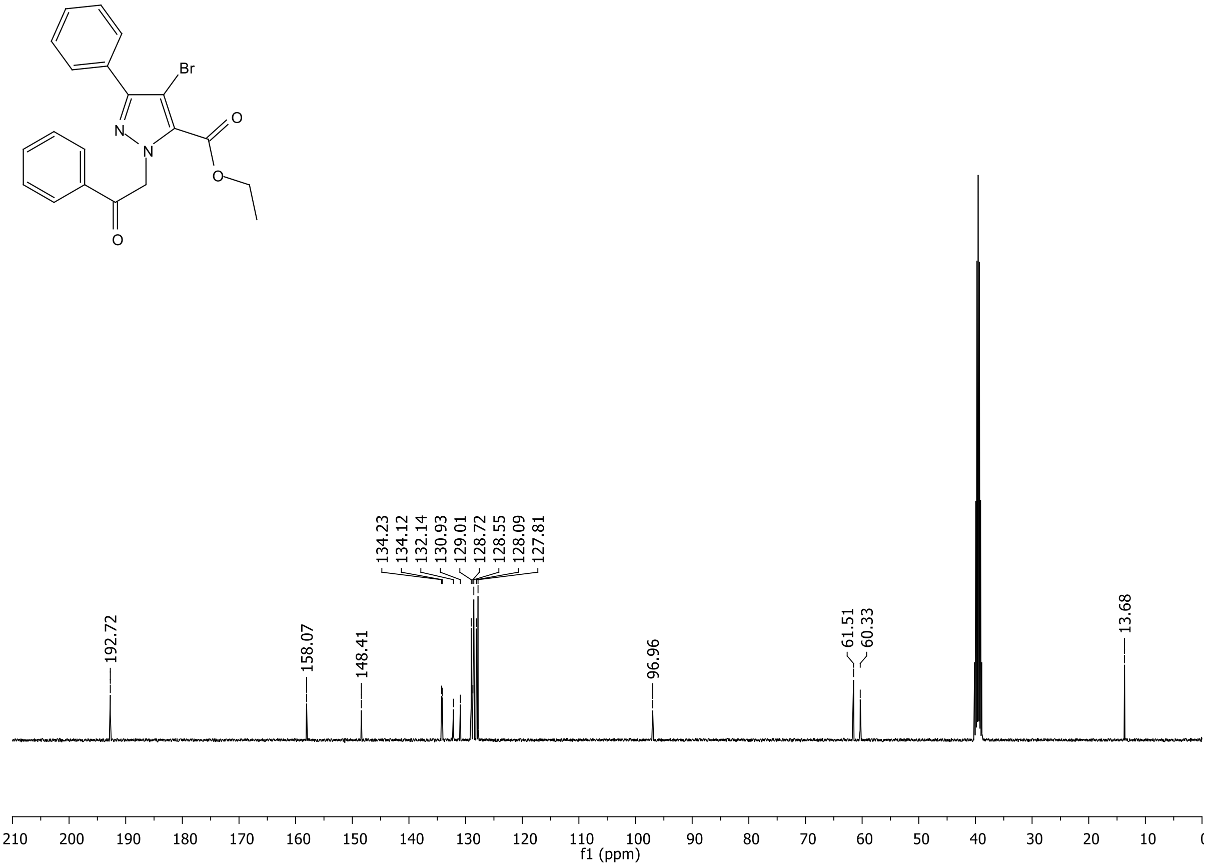

Figure S2. ^{13}C NMR spectrum (101 MHz, DMSO-d_6) of ethyl 4-bromo-1-(2-oxo-2-phenylethyl)-3-phenyl-1*H*-pyrazole-5-carboxylate (**4a**).

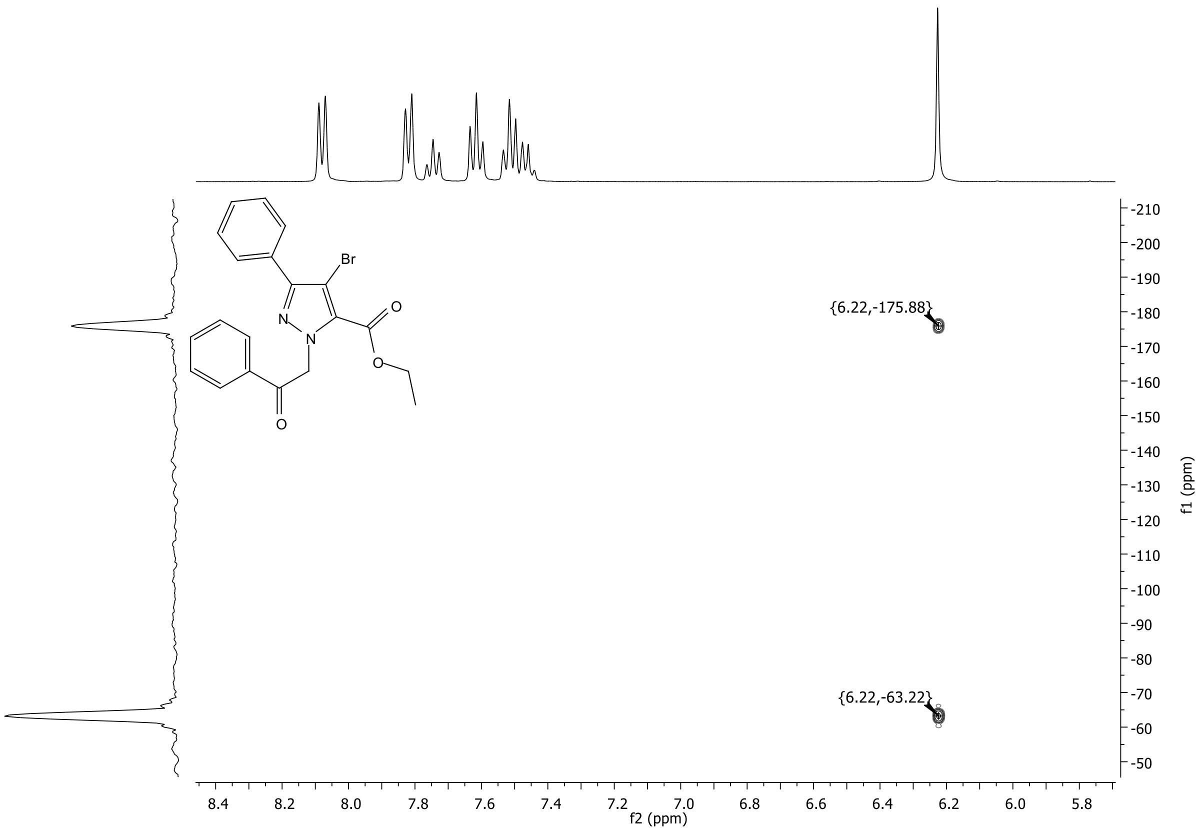

Figure S3. $^{1}H,^{15}N$-HMBC NMR spectrum (40 MHz, DMSO-d_6) of ethyl 4-bromo-1-(2-oxo-2-phenylethyl)-3-phenyl-1*H*-pyrazole-5-carboxylate (**4a**).

Compound Spectrum SmartFormula Report

Analysis Info

Analysis Name	D:\Data\KDD-257-1.d	Acquisition Date	6/17/2022 12:01:26 PM
Method	DirectInfusion_TuneLow_pos.m	Operator	hplc
Sample Name	KDD-257-1	Instrument	micrOTOF-Q III 8228888.20448
Comment	AB		

Acquisition Parameter

Source Type	ESI	Ion Polarity	Positive	Set Nebulizer	0.4 Bar
Focus	Not active	Set Capillary	4500 V	Set Dry Heater	180 °C
Scan Begin	50 m/z	Set End Plate Offset	-500 V	Set Dry Gas	4.0 l/min
Scan End	1000 m/z	Set Collision Cell RF	140.0 Vpp	Set Divert Valve	Waste

#	RT [min]	Area	Int. Type	I	S/N	Chromatogram	Max. m/z	FWHM [min]
n.a.	5.8	n.a.	Single spectrum	n.a.	n.a.	n.a.	435.0316	n.a.

+MS, 5.8min #345

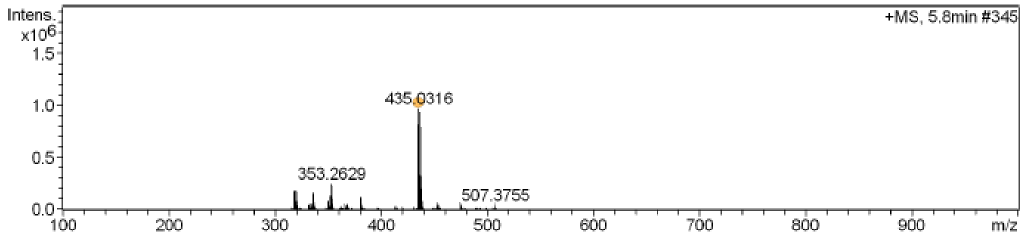

Meas. m/z	#	Ion Formula	m/z	err [ppm]	mSigma	# Sigma	Score	rdb	e⁻ Conf	N-Rule
435.0316	1	C20H17BrN2NaO3	435.0315	-0.3	25.5	1	100.00	12.5	even	ok

Figure S4. HRMS (ESI-TOF) spectrum of ethyl 4-bromo-1-(2-oxo-2-phenylethyl)-3-phenyl-1*H*-pyrazole-5-carboxylate (**4a**).

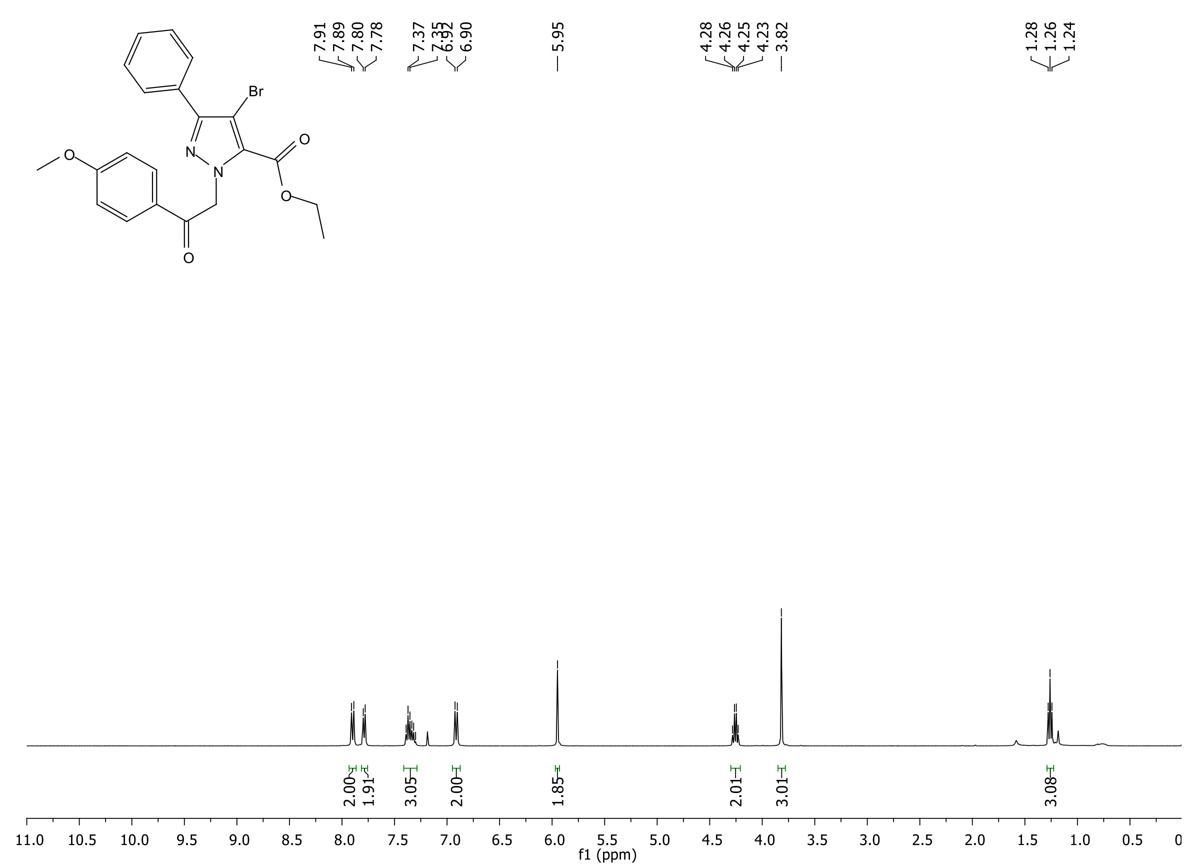

Figure S5. ^{1}H NMR spectrum (400 MHz, $CDCl_3$) of ethyl 4-bromo-1-[2-(4-methoxyphenyl)-2-oxoethyl]-3-phenyl-1*H*-pyrazole-5-carboxylate (**4b**).

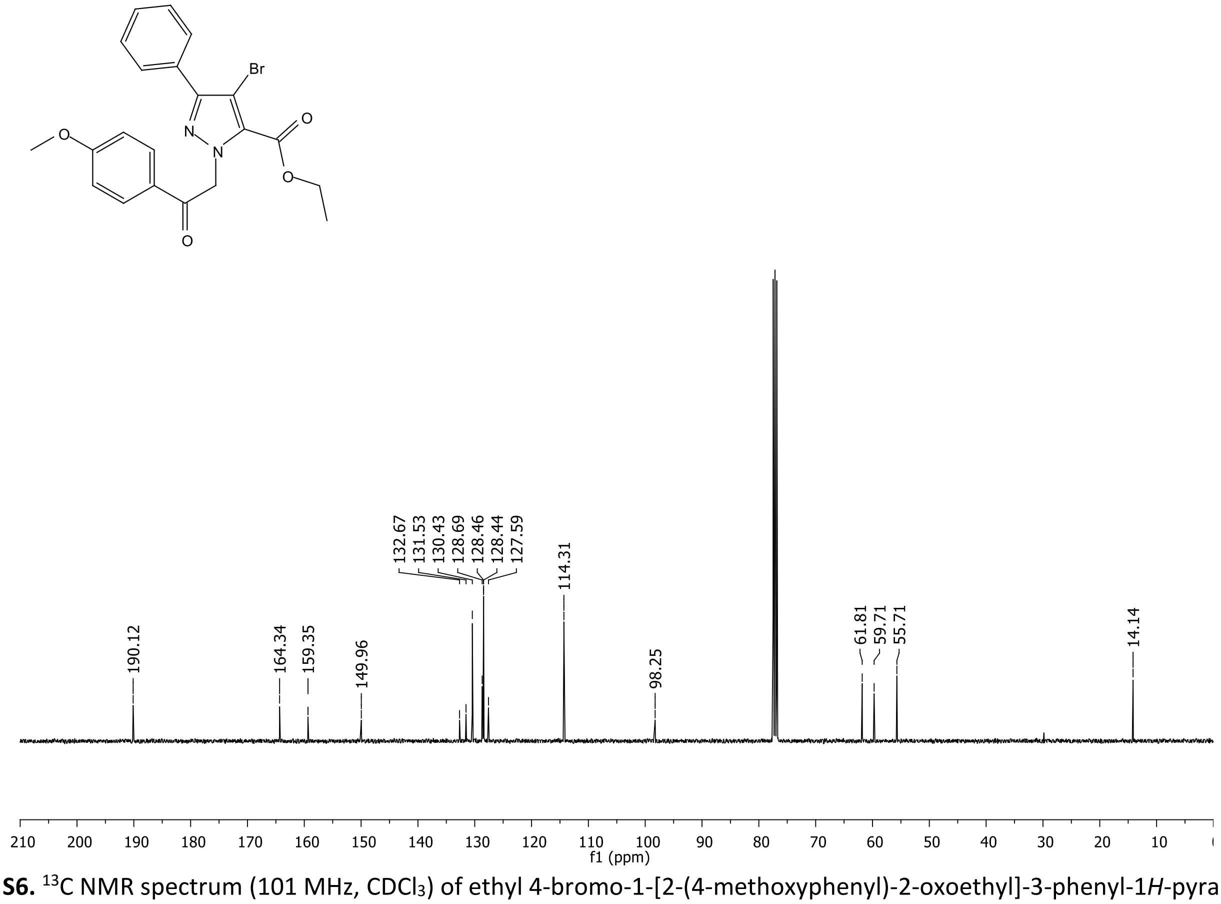

Figure S6. ^{13}C NMR spectrum (101 MHz, $CDCl_3$) of ethyl 4-bromo-1-[2-(4-methoxyphenyl)-2-oxoethyl]-3-phenyl-1*H*-pyrazole-5-carboxylate (**4b**).

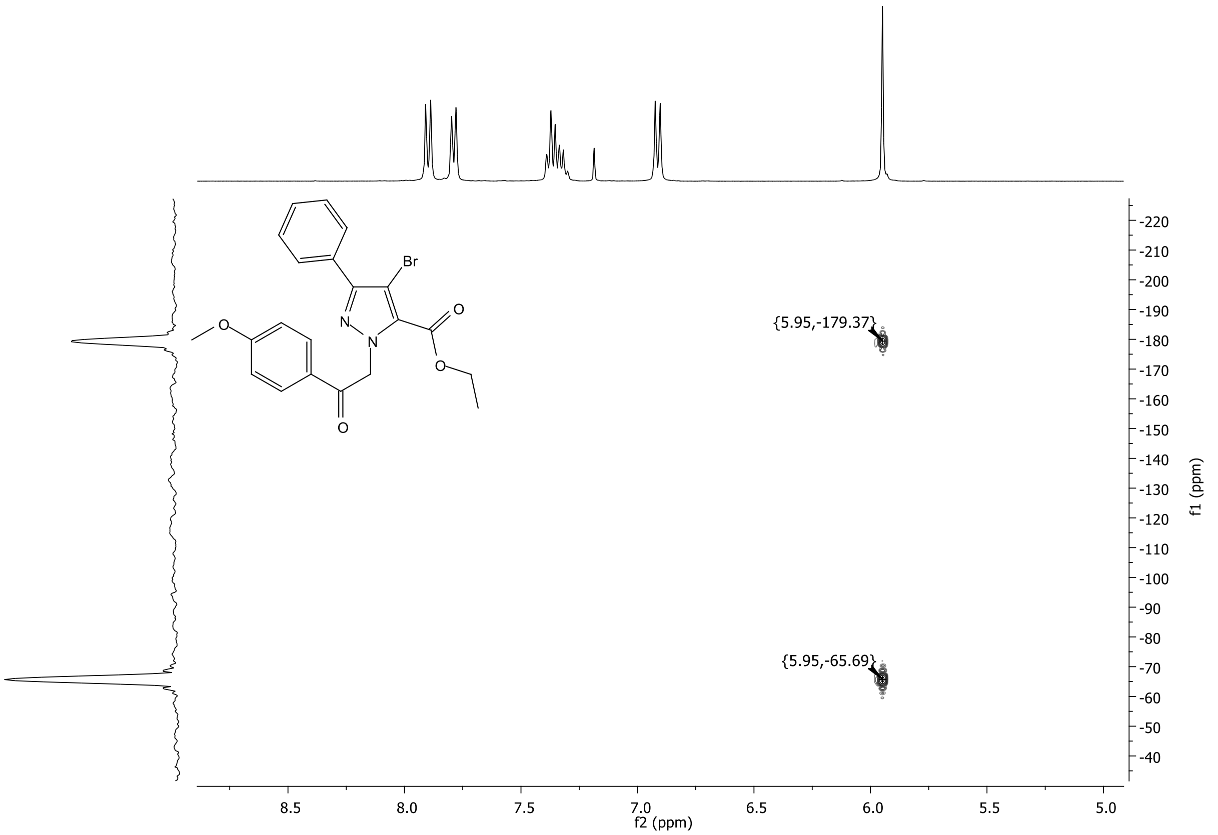

Figure S7. $^{1}H,^{15}N$-HMBC NMR spectrum (40 MHz, $CDCl_3$) of ethyl 4-bromo-1-[2-(4-methoxyphenyl)-2-oxoethyl]-3-phenyl-1*H*-pyrazole-5-carboxylate (**4b**).

Compound Spectrum SmartFormula Report

Analysis Info

Analysis Name	D:\Data\KDD-387.d	Acquisition Date	7/4/2022 4:17:23 PM
Method	DirectInfusion_TuneLow_pos.m	Operator	hplc
Sample Name	KDD-387	Instrument	micrOTOF-Q III 8228888.20448
Comment	AB		

Acquisition Parameter

Source Type	ESI	Ion Polarity	Positive	Set Nebulizer	0.4 Bar
Focus	Not active	Set Capillary	4500 V	Set Dry Heater	180 °C
Scan Begin	50 m/z	Set End Plate Offset	-500 V	Set Dry Gas	4.0 l/min
Scan End	1000 m/z	Set Collision Cell RF	140.0 Vpp	Set Divert Valve	Waste

#	RT [min]	Area	Int. Type	I	S/N	Chromatogram	Max. m/z	FWHM [min]
n.a.	6.1	n.a.	Single spectrum	n.a.	n.a.	n.a.	467.0408	n.a.

+MS, 6.1min #365

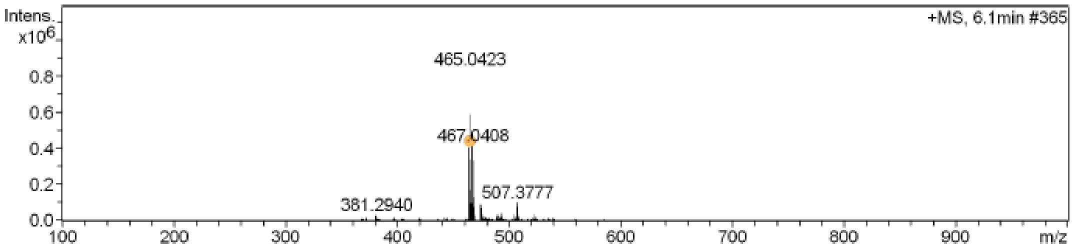

Meas. m/z	#	Ion Formula	m/z	err [ppm]	mSigma	# Sigma	Score	rdb	e⁻ Conf	N-Rule
465.0423	1	C21H19BrN2NaO4	465.0420	-0.5	4.1	1	100.00	12.5	even	ok

Figure S8. HRMS (ESI-TOF) spectrum of ethyl 4-bromo-1-[2-(4-methoxyphenyl)-2-oxoethyl]-3-phenyl-1*H*-pyrazole-5-carboxylate (**4b**).

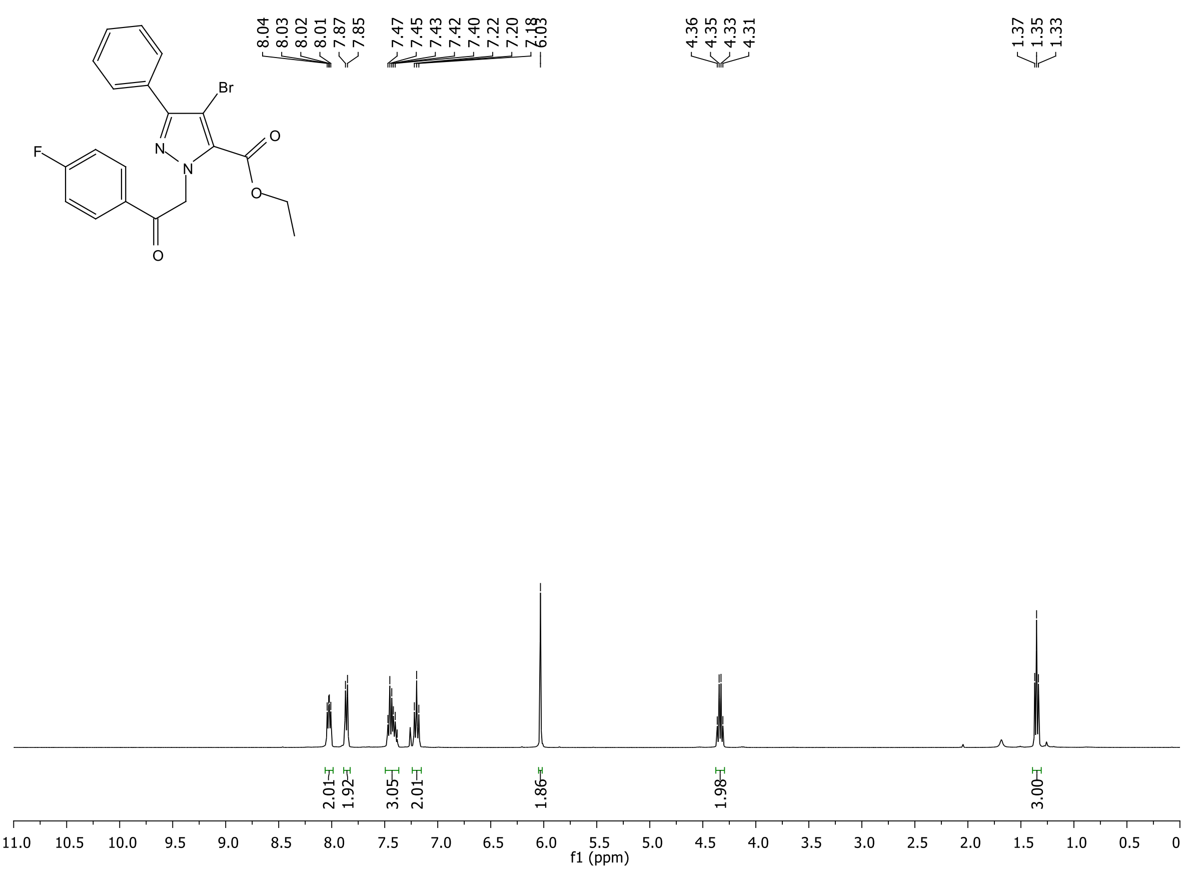

Figure S9. ^{1}H NMR spectrum (400 MHz, $CDCl_3$) of ethyl 4-bromo-1-[2-(4-fluorophenyl)-2-oxoethyl]-3-phenyl-1*H*-pyrazole-5-carboxylate (**4c**).

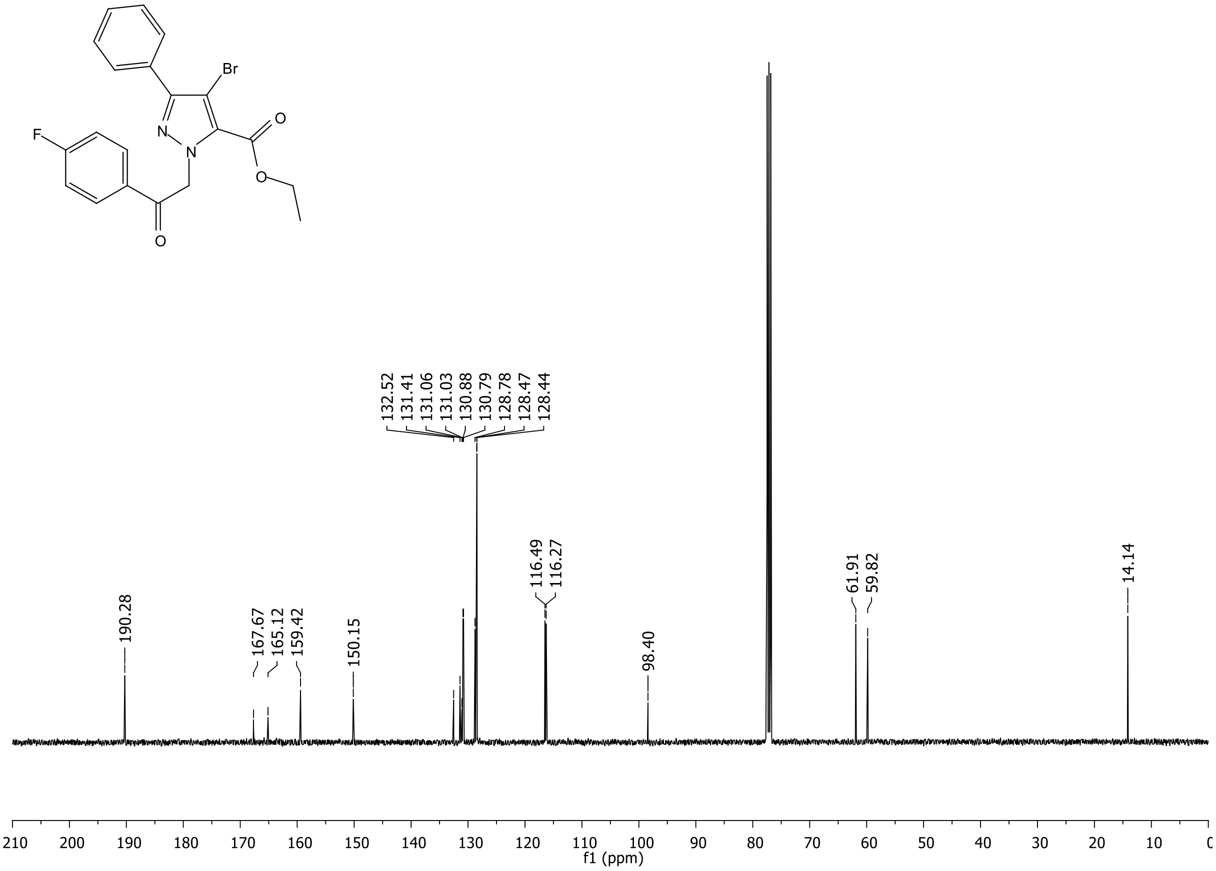

Figure S10. ^{13}C NMR spectrum (101 MHz, $CDCl_3$) of ethyl 4-bromo-1-[2-(4-fluorophenyl)-2-oxoethyl]-3-phenyl-1*H*-pyrazole-5-carboxylate (**4c**).

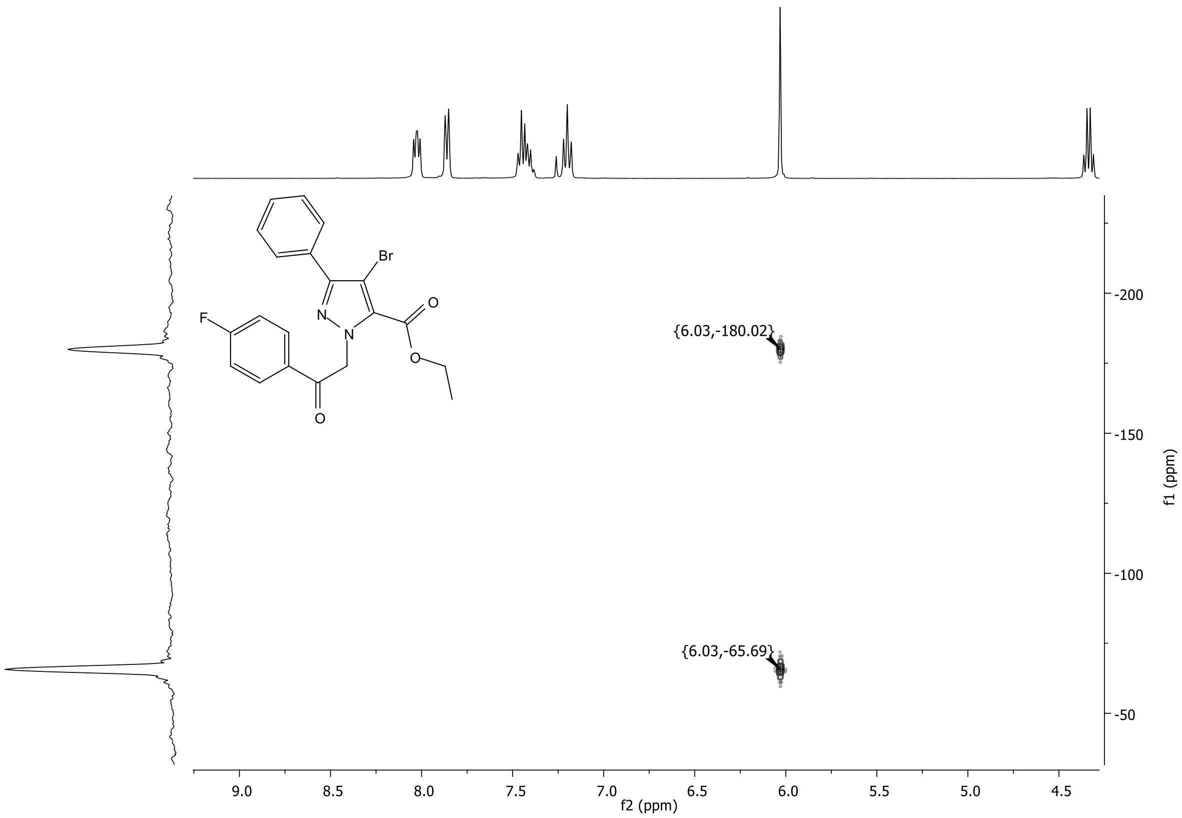

Figure S11. $^{1}H,^{15}N$-HMBC NMR spectrum (40 MHz, $CDCl_3$) of ethyl 4-bromo-1-[2-(4-fluorophenyl)-2-oxoethyl]-3-phenyl-1*H*-pyrazole-5-carboxylate (**4c**).

Compound Spectrum SmartFormula Report

Analysis Info

Analysis Name	D:\Data\KDD-315.d	Acquisition Date	6/17/2022 4:56:23 PM
Method	DirectInfusion_TuneLow_pos.m	Operator	hplc
Sample Name	KDD-315	Instrument	micrOTOF-Q III 8228888.20448
Comment	AB		

Acquisition Parameter

Source Type	ESI	Ion Polarity	Positive	Set Nebulizer	0.4 Bar
Focus	Not active	Set Capillary	4500 V	Set Dry Heater	180 °C
Scan Begin	50 m/z	Set End Plate Offset	-500 V	Set Dry Gas	4.0 l/min
Scan End	1000 m/z	Set Collision Cell RF	140.0 Vpp	Set Divert Valve	Waste

#	RT [min]	Area	Int. Type	I	S/N	Chromatogram	Max. m/z	FWHM [min]
n.a.	6.0	n.a.	Single spectrum	n.a.	n.a.	n.a.	453.0218	n.a.

+MS, 6.0min #360

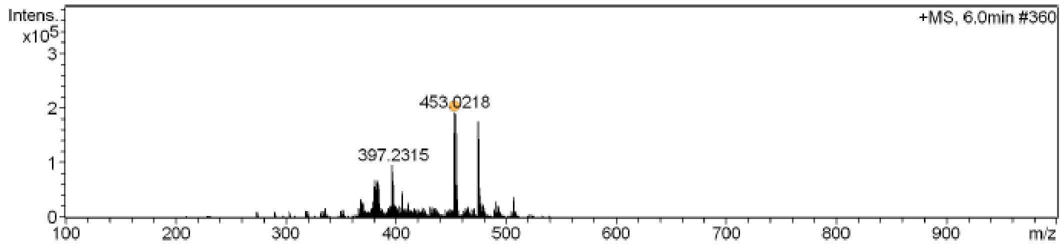

Meas. m/z	#	Ion Formula	m/z	err [ppm]	mSigma	# Sigma	Score	rdb	e⁻ Conf	N-Rule
453.0218	1	C20H16BrFN2NaO3	453.0221	-0.7	9.4	1	100.00	12.5	even	ok

Figure S12. HRMS (ESI-TOF) spectrum of ethyl 4-bromo-1-[2-(4-fluorophenyl)-2-oxoethyl]-3-phenyl-1*H*-pyrazole-5-carboxylate (**4c**).

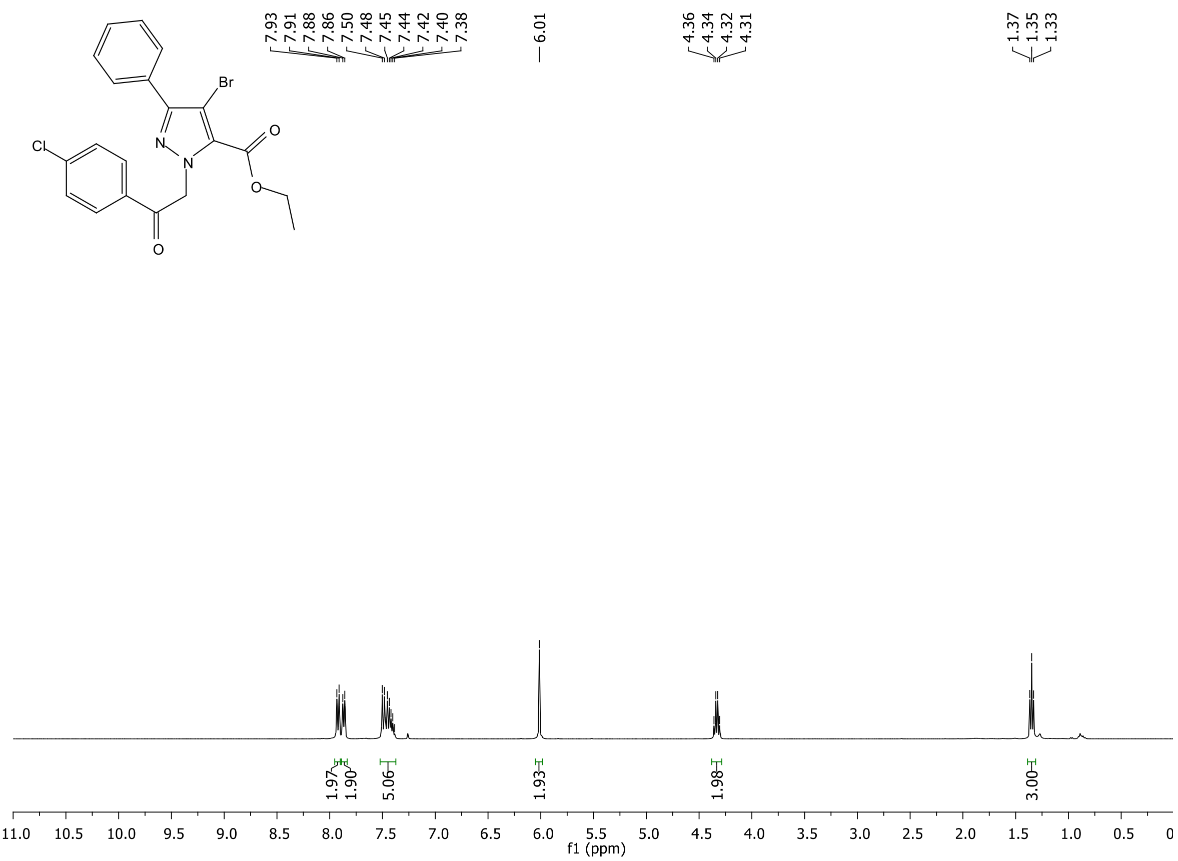

Figure S13. ^{1}H NMR spectrum (400 MHz, $CDCl_3$) of ethyl 4-bromo-1-[2-(4-chlorophenyl)-2-oxoethyl]-3-phenyl-1*H*-pyrazole-5-carboxylate (**4d**).

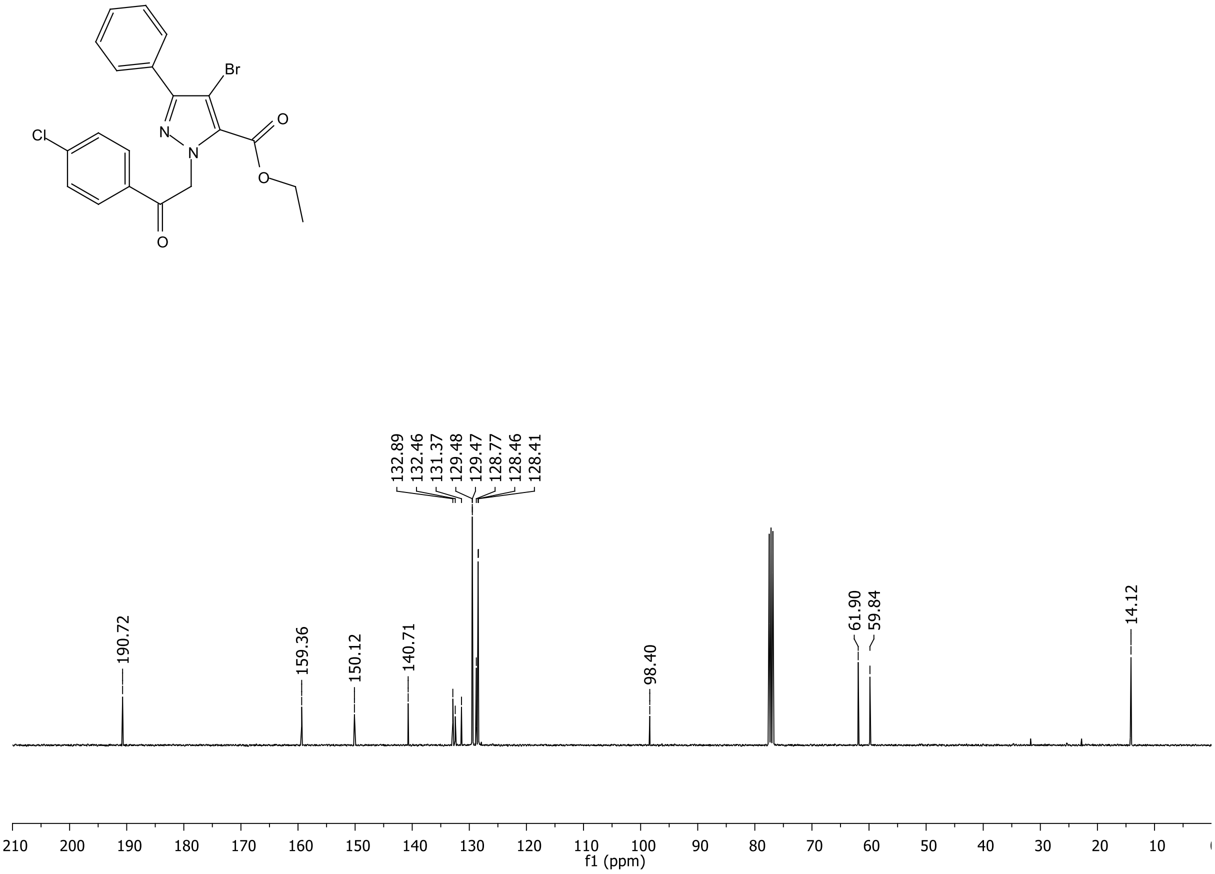

Figure S14. ^{13}C NMR spectrum (101 MHz, $CDCl_3$) of ethyl 4-bromo-1-[2-(4-chlorophenyl)-2-oxoethyl]-3-phenyl-1*H*-pyrazole-5-carboxylate (**4d**).

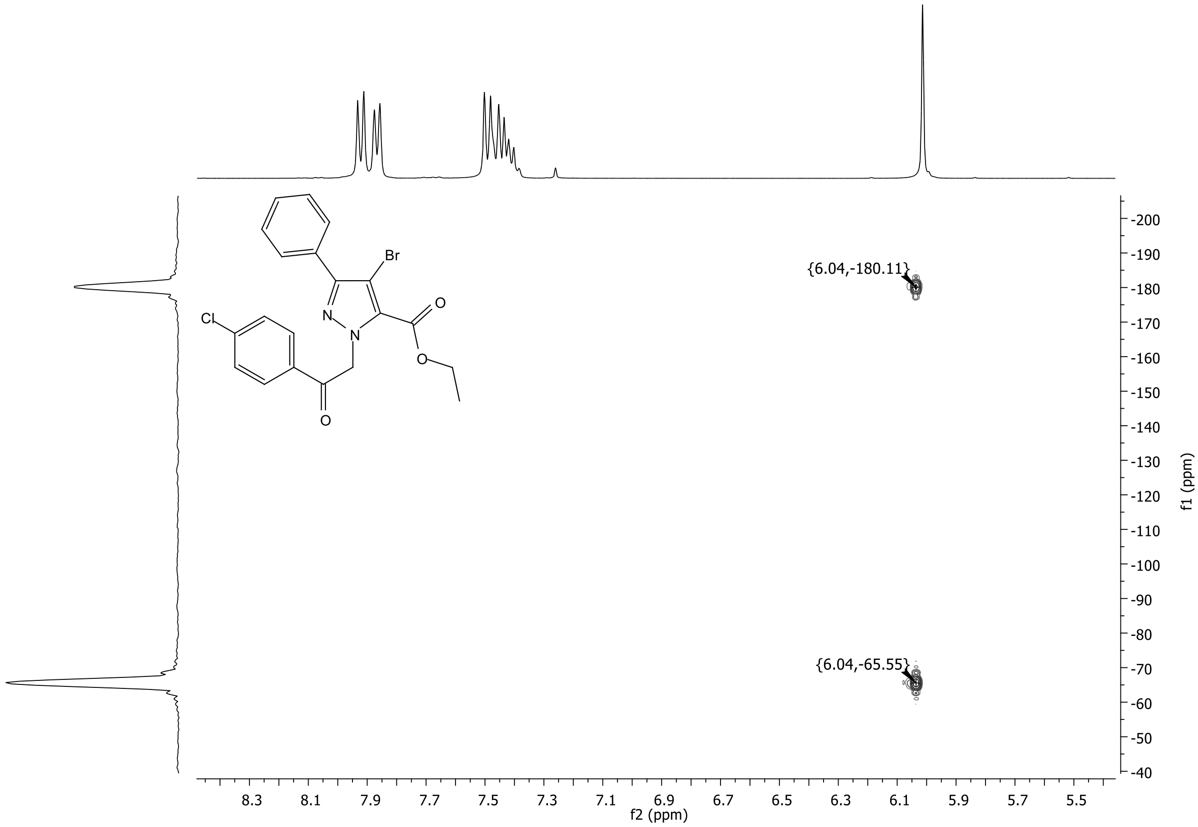

Figure S15. $^{1}H,^{15}N$-HMBC NMR spectrum (40 MHz, $CDCl_3$) of ethyl 4-bromo-1-[2-(4-chlorophenyl)-2-oxoethyl]-3-phenyl-1*H*-pyrazole-5-carboxylate (**4d**).

Compound Spectrum SmartFormula Report

Analysis Info

Analysis Name	D:\Data\KDD-388.d	Acquisition Date	7/4/2022 4:43:16 PM
Method	DirectInfusion_TuneLow_pos.m	Operator	hplc
Sample Name	KDD-388	Instrument	micrOTOF-Q III 8228888.20448
Comment	AB		

Acquisition Parameter

Source Type	ESI	Ion Polarity	Positive	Set Nebulizer	0.4 Bar
Focus	Not active	Set Capillary	4500 V	Set Dry Heater	180 °C
Scan Begin	50 m/z	Set End Plate Offset	-500 V	Set Dry Gas	4.0 l/min
Scan End	1000 m/z	Set Collision Cell RF	140.0 Vpp	Set Divert Valve	Waste

#	RT [min]	Area	Int. Type	I	S/N	Chromatogram	Max. m/z	FWHM [min]
n.a.	3.9	n.a.	Single spectrum	n.a.	n.a.	n.a.	470.9905	n.a.

+MS, 3.9min #235

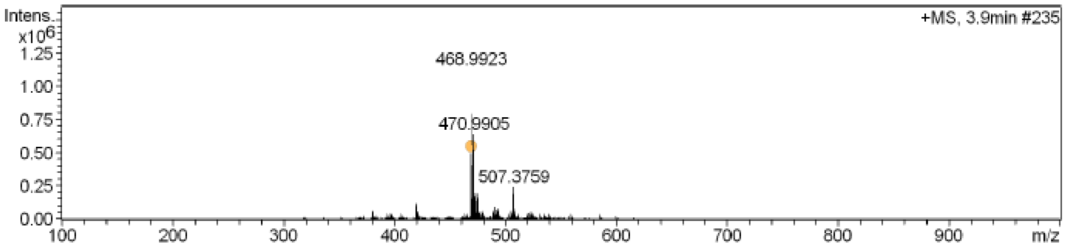

Meas. m/z	#	Ion Formula	m/z	err [ppm]	mSigma	# Sigma	Score	rdb	e⁻ Conf	N-Rule
468.9923	1	C20H16BrClN2NaO3	468.9925	-0.3	12.1	1	100.00	12.5	even	ok

Figure S16. HRMS (ESI-TOF) spectrum of ethyl 4-bromo-1-[2-(4-chlorophenyl)-2-oxoethyl]-3-phenyl-1*H*-pyrazole-5-carboxylate (**4d**).

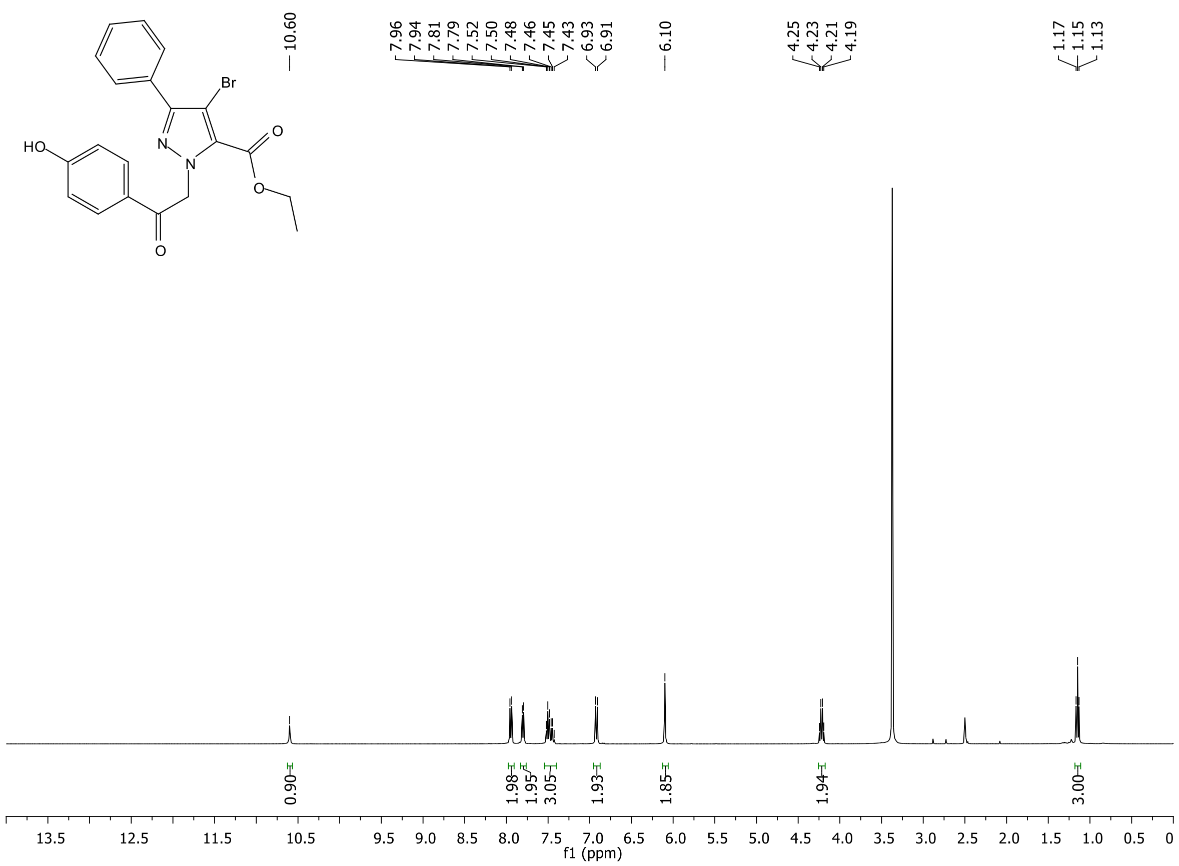

Figure S17. ^{1}H NMR spectrum (400 MHz, DMSO-d_6) of ethyl 4-bromo-1-[2-(4-hydroxyphenyl)-2-oxoethyl]-3-phenyl-1*H*-pyrazole-5-carboxylate (**4e**).

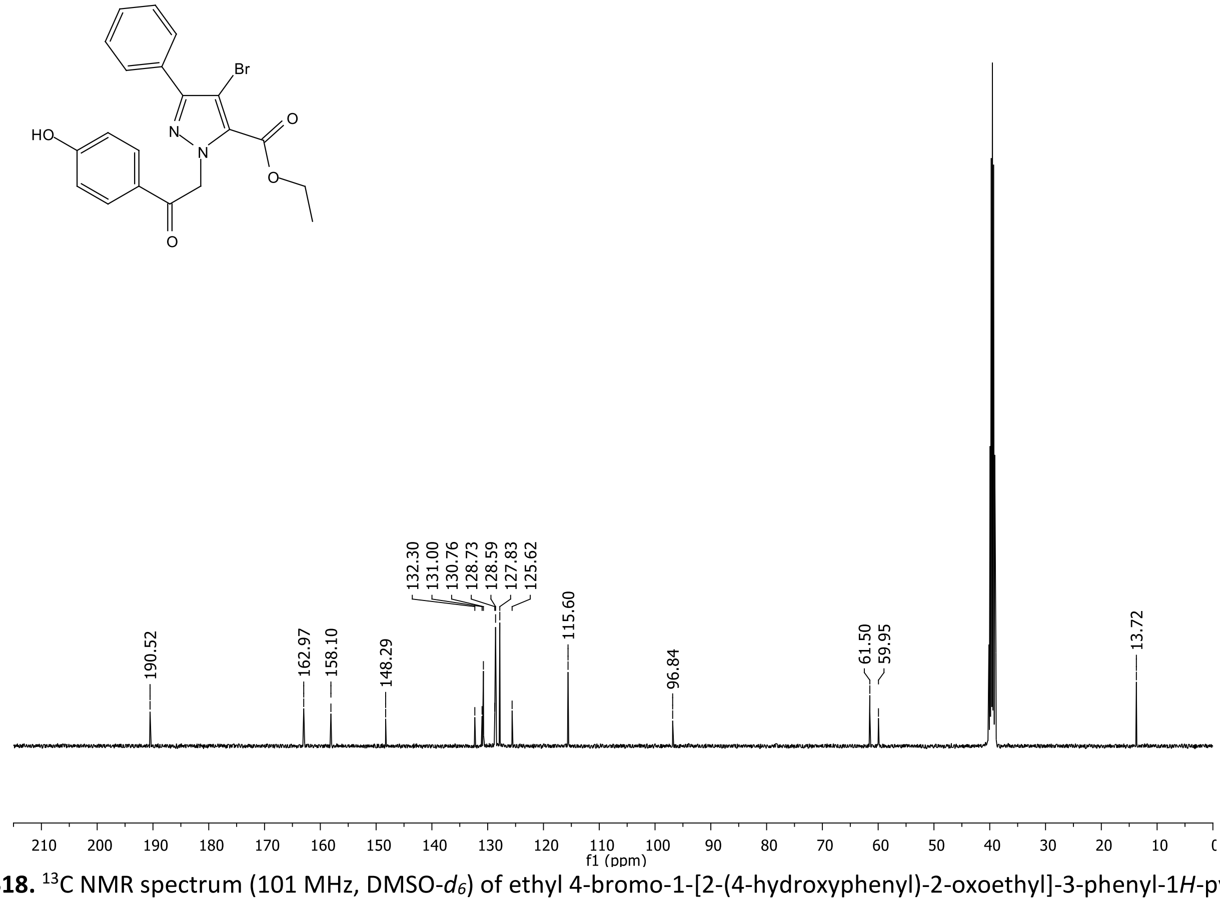

Figure S18. ^{13}C NMR spectrum (101 MHz, DMSO-d_6) of ethyl 4-bromo-1-[2-(4-hydroxyphenyl)-2-oxoethyl]-3-phenyl-1*H*-pyrazole-5-carboxylate (**4e**).

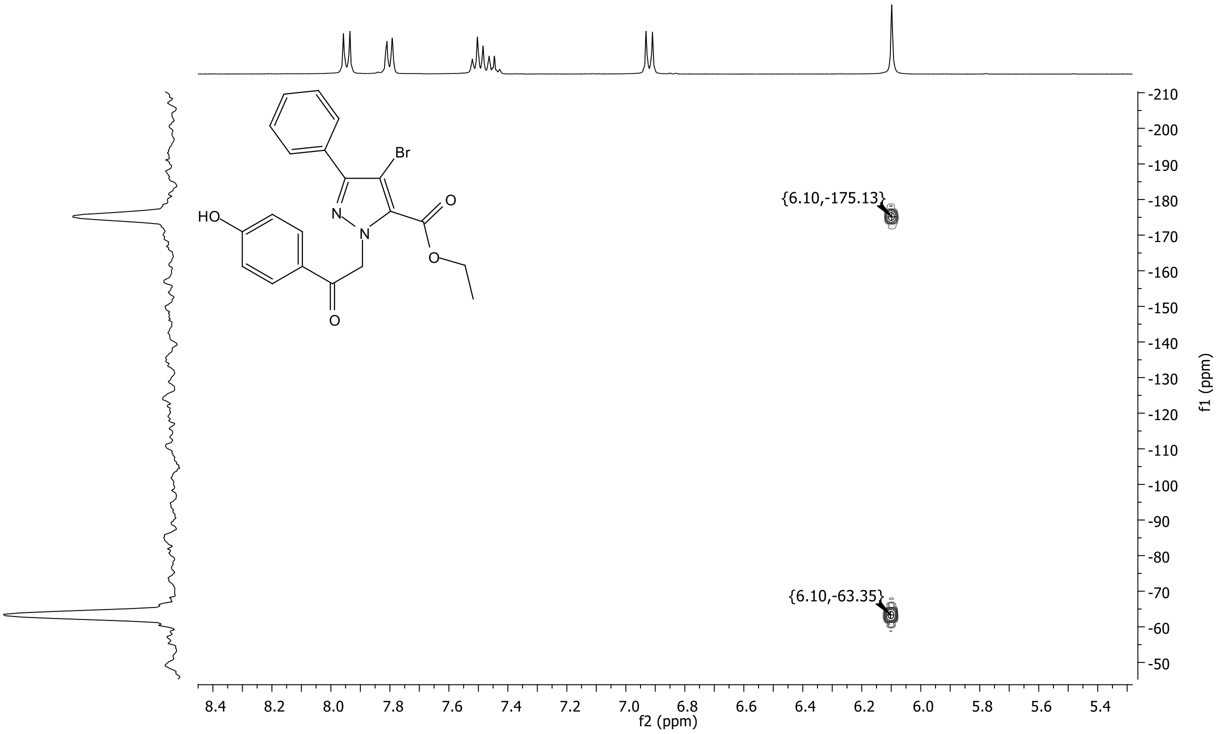

Figure S19. $^{1}H,^{15}N$-HMBC NMR spectrum (40 MHz, DMSO-d_6) of ethyl 4-bromo-1-[2-(4-hydroxyphenyl)-2-oxoethyl]-3-phenyl-1*H*-pyrazole-5-carboxylate (**4e**).

Compound Spectrum SmartFormula Report

Analysis Info

Analysis Name	D:\Data\KDD-316.d	Acquisition Date	6/17/2022 5:11:38 PM
Method	DirectInfusion_TuneLow_pos.m	Operator	hplc
Sample Name	KDD-316	Instrument	micrOTOF-Q III 8228888.20448
Comment	AB		

Acquisition Parameter

Source Type	ESI	Ion Polarity	Positive	Set Nebulizer	0.4 Bar
Focus	Not active	Set Capillary	4500 V	Set Dry Heater	180 °C
Scan Begin	50 m/z	Set End Plate Offset	-500 V	Set Dry Gas	4.0 l/min
Scan End	1000 m/z	Set Collision Cell RF	140.0 Vpp	Set Divert Valve	Waste

#	RT [min]	Area	Int. Type	I	S/N	Chromatogram	Max. m/z	FWHM [min]
n.a.	3.6	n.a.	Single spectrum	n.a.	n.a.	n.a.	453.0247	n.a.

+MS, 3.6min #216

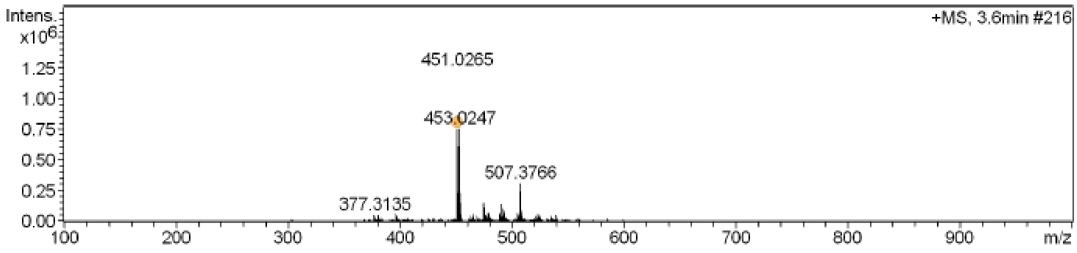

Meas. m/z	#	Ion Formula	m/z	err [ppm]	mSigma	# Sigma	Score	rdb	e⁻ Conf	N-Rule
451.0265	1	C20H17BrN2NaO4	451.0264	-0.3	15.3	3	100.00	12.5	even	ok

Figure S20. HRMS (ESI-TOF) spectrum of ethyl 4-bromo-1-[2-(4-hydroxyphenyl)-2-oxoethyl]-3-phenyl-1*H*-pyrazole-5-carboxylate (**4e**).

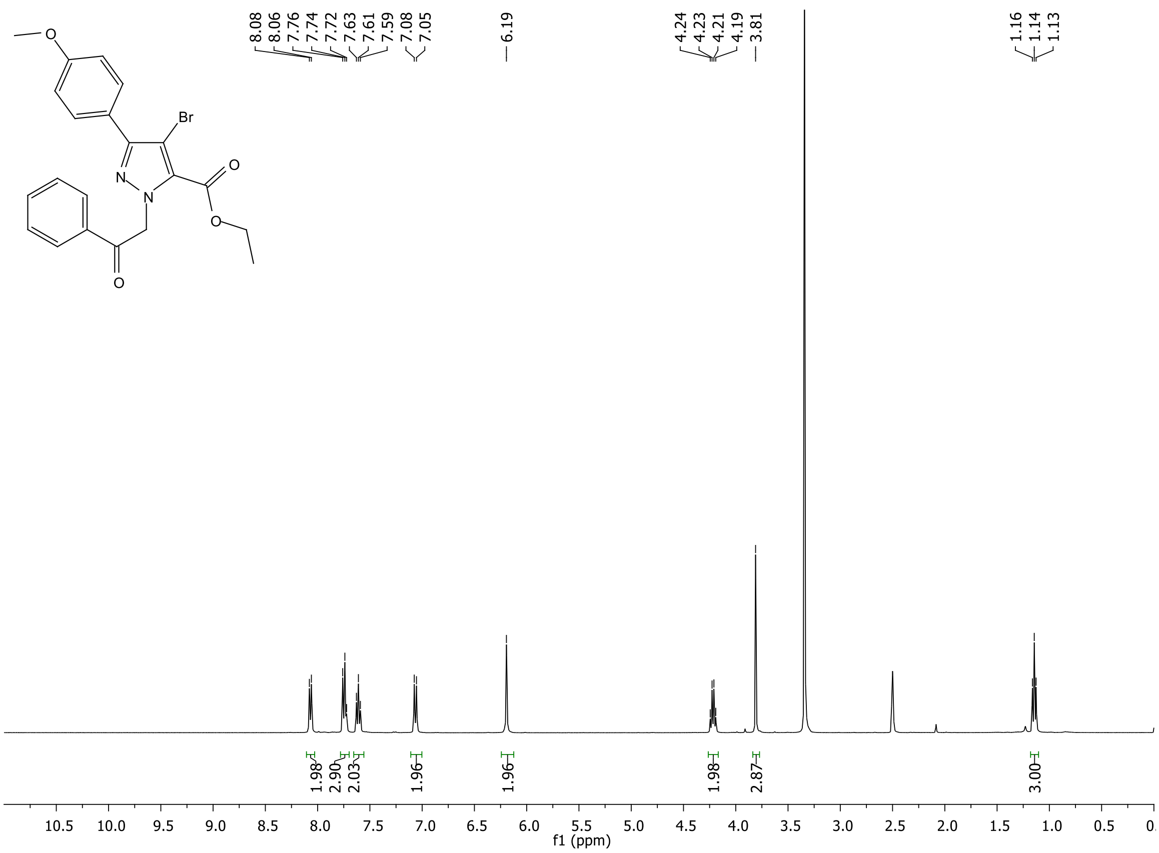

Figure S21. ^{1}H NMR spectrum (400 MHz, DMSO-d_6) of ethyl 4-bromo-3-(4-methoxyphenyl)-1-(2-oxo-2-phenylethyl)-1*H*-pyrazole-5-carboxylate (**4f**).

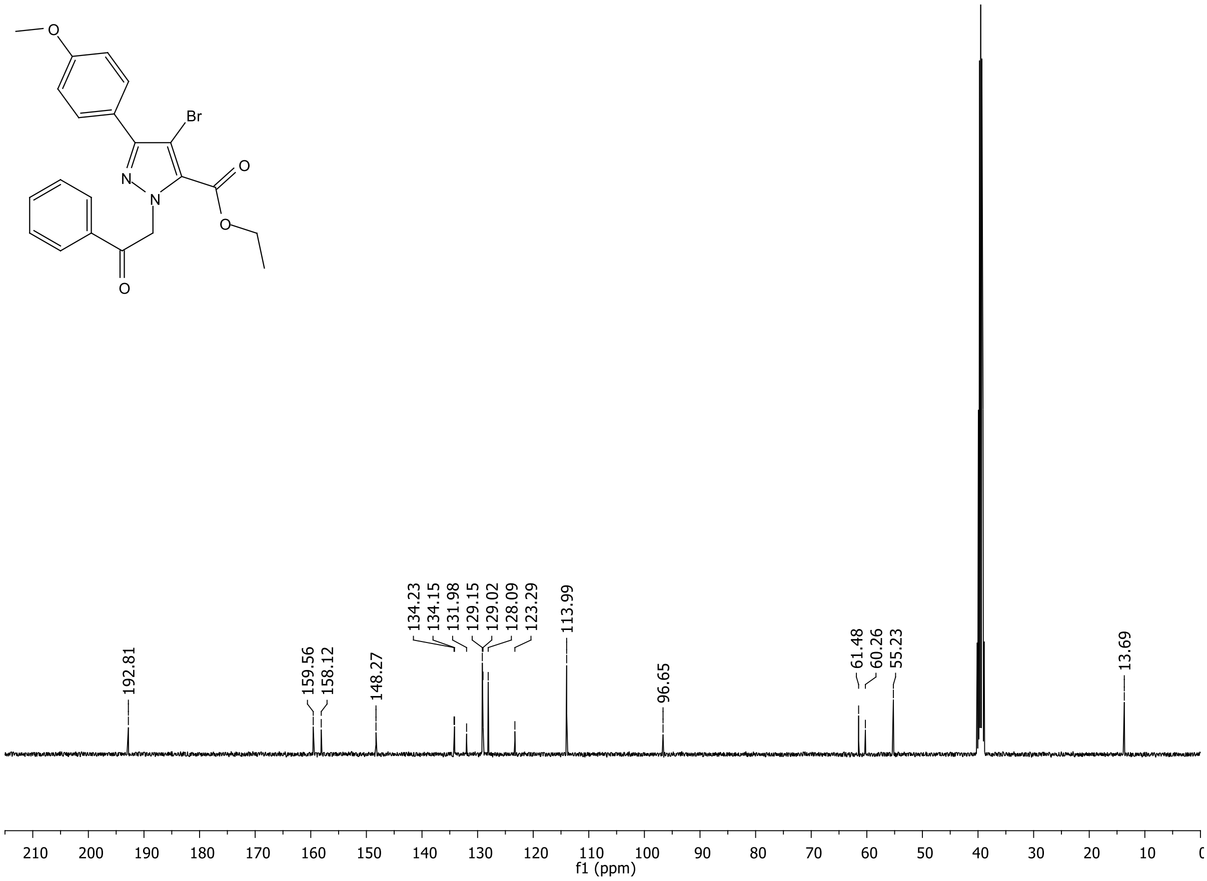

Figure S22. ^{13}C NMR spectrum (101 MHz, DMSO-d_6) of ethyl 4-bromo-3-(4-methoxyphenyl)-1-(2-oxo-2-phenylethyl)-1*H*-pyrazole-5-carboxylate (**4f**).

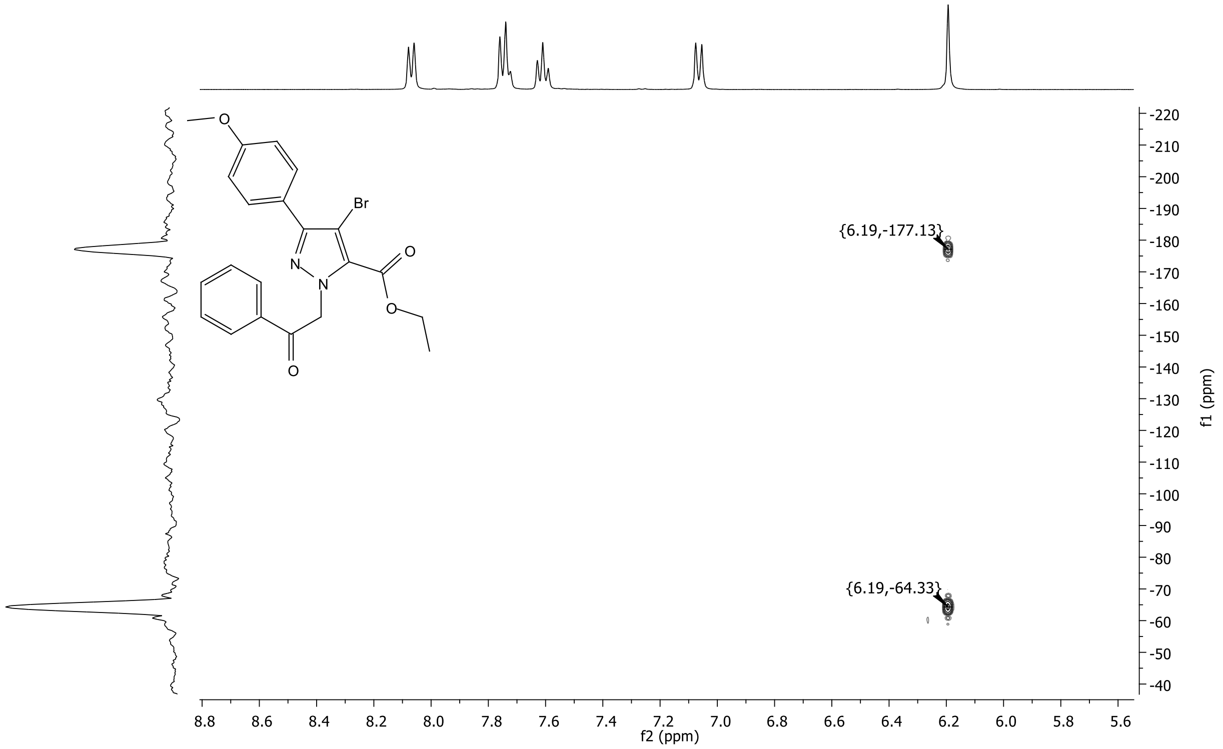

Figure S23. $^{1}H,^{15}N$-HMBC NMR spectrum (40 MHz, DMSO-d_6) of ethyl 4-bromo-3-(4-methoxyphenyl)-1-(2-oxo-2-phenylethyl)-1*H*-pyrazole-5-carboxylate (**4f**).

Compound Spectrum SmartFormula Report

Analysis Info

Analysis Name	D:\Data\KDD-302.d	Acquisition Date	6/17/2022 12:47:16 PM
Method	DirectInfusion_TuneLow_pos.m	Operator	hplc
Sample Name	KDD-302	Instrument	micrOTOF-Q III 8228888.20448
Comment	AB		

Acquisition Parameter

Source Type	ESI	Ion Polarity	Positive	Set Nebulizer	0.4 Bar
Focus	Not active	Set Capillary	4500 V	Set Dry Heater	180 °C
Scan Begin	50 m/z	Set End Plate Offset	-500 V	Set Dry Gas	4.0 l/min
Scan End	1000 m/z	Set Collision Cell RF	140.0 Vpp	Set Divert Valve	Waste

#	RT [min]	Area	Int. Type	I	S/N	Chromatogram	Max. m/z	FWHM [min]
n.a.	8.8	n.a.	Single spectrum	n.a.	n.a.	n.a.	465.0418	n.a.

+MS, 8.8min #528

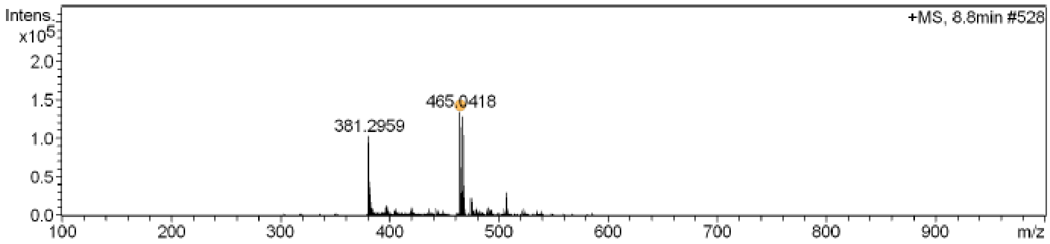

Meas. m/z	#	Ion Formula	m/z	err [ppm]	mSigma	# Sigma	Score	rdb	e⁻ Conf	N-Rule
465.0418	1	C21H19BrN2NaO4	465.0420	0.6	18.0	1	100.00	12.5	even	ok

Figure S24. HRMS (ESI-TOF) spectrum of ethyl 4-bromo-3-(4-methoxyphenyl)-1-(2-oxo-2-phenylethyl)-1*H*-pyrazole-5-carboxylate (**4f**).

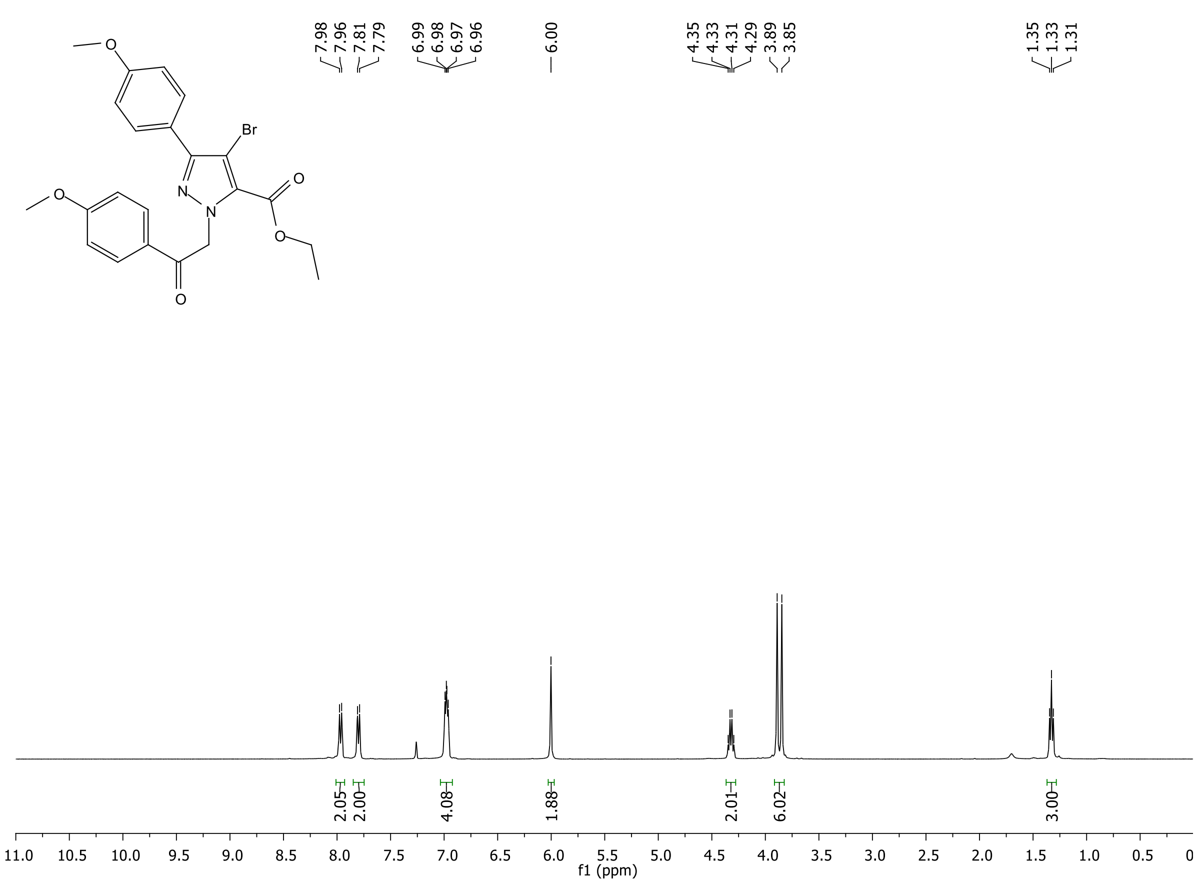

Figure S25. ^{1}H NMR spectrum (400 MHz, $CDCl_3$) of ethyl 4-bromo-3-(4-methoxyphenyl)-1-[2-(4-methoxyphenyl)-2-oxoethyl]-1*H*-pyrazole-5-carboxylate (**4g**).

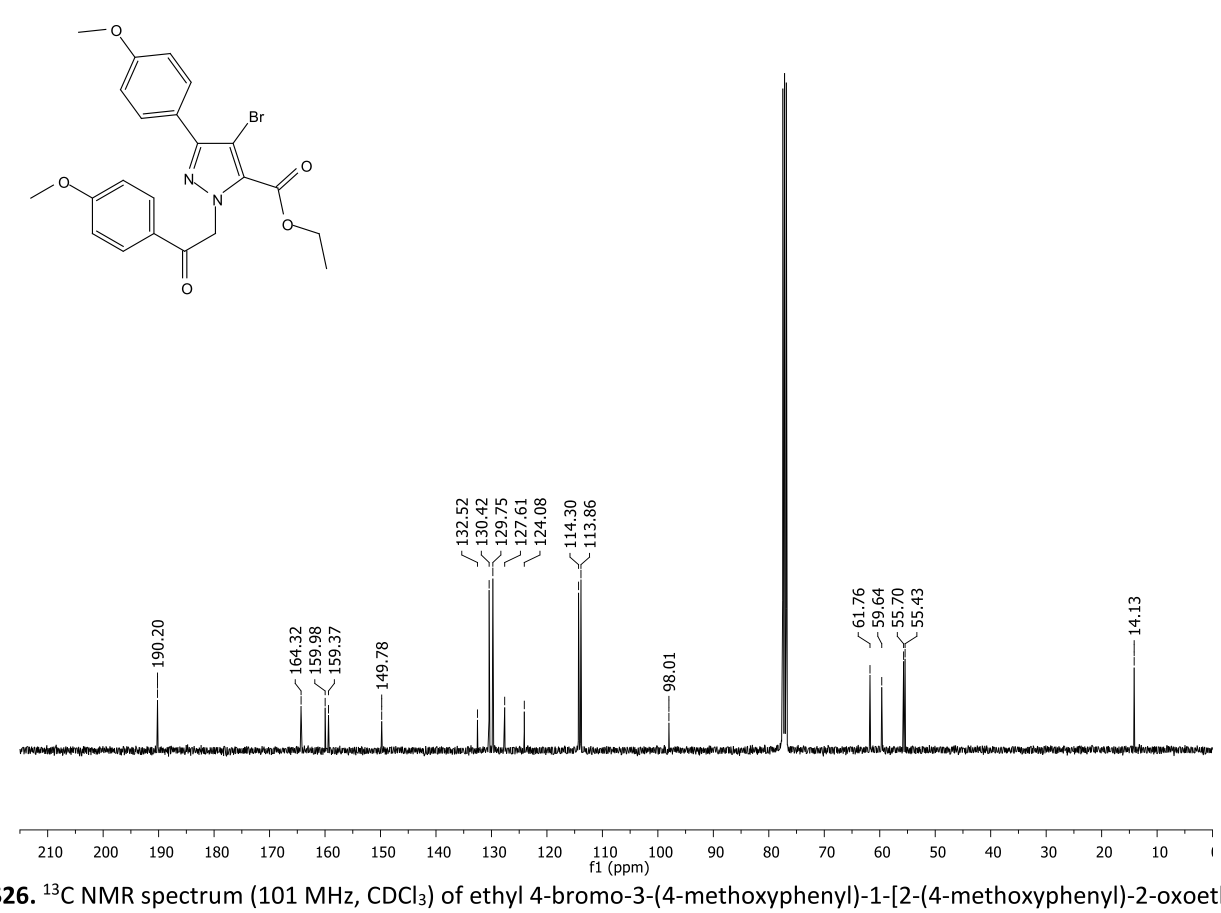

Figure S26. ^{13}C NMR spectrum (101 MHz, $CDCl_3$) of ethyl 4-bromo-3-(4-methoxyphenyl)-1-[2-(4-methoxyphenyl)-2-oxoethyl]-1*H*-pyrazole-5-carboxylate (**4g**).

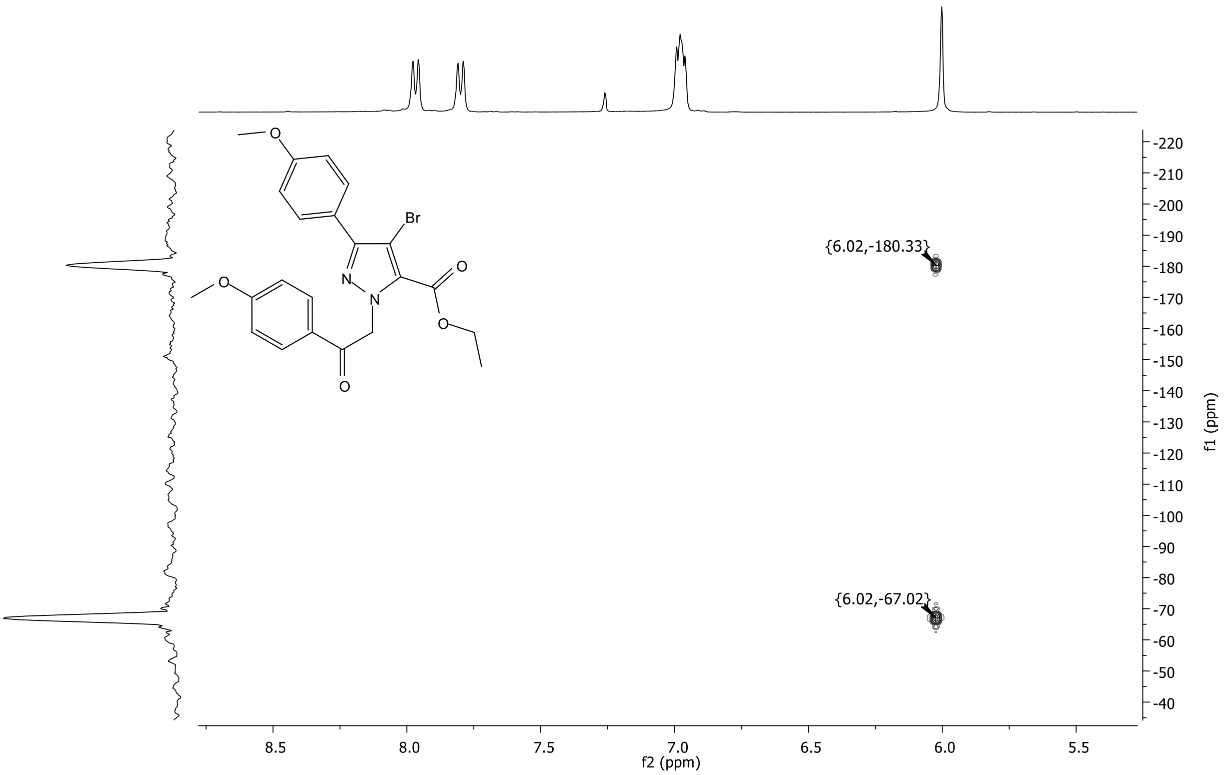

Figure S27. $^{1}H,^{15}N$-HMBC NMR spectrum (40 MHz, $CDCl_3$) of ethyl 4-bromo-3-(4-methoxyphenyl)-1-[2-(4-methoxyphenyl)-2-oxoethyl]-1*H*-pyrazole-5-carboxylate (**4g**).

Compound Spectrum SmartFormula Report

Analysis Info

Analysis Name	D:\Data\KDD-303.d	Acquisition Date	6/17/2022 1:07:02 PM
Method	DirectInfusion_TuneLow_pos.m	Operator	hplc
Sample Name	KDD-303	Instrument	micrOTOF-Q III 8228888.20448
Comment	AB		

Acquisition Parameter

Source Type	ESI	Ion Polarity	Positive	Set Nebulizer	0.4 Bar
Focus	Not active	Set Capillary	4500 V	Set Dry Heater	180 °C
Scan Begin	50 m/z	Set End Plate Offset	-500 V	Set Dry Gas	4.0 l/min
Scan End	1000 m/z	Set Collision Cell RF	140.0 Vpp	Set Divert Valve	Waste

#	RT [min]	Area	Int. Type	I	S/N	Chromatogram	Max. m/z	FWHM [min]
n.a.	5.4	n.a.	Single spectrum	n.a.	n.a.	n.a.	495.0523	n.a.

+MS, 5.4min #326

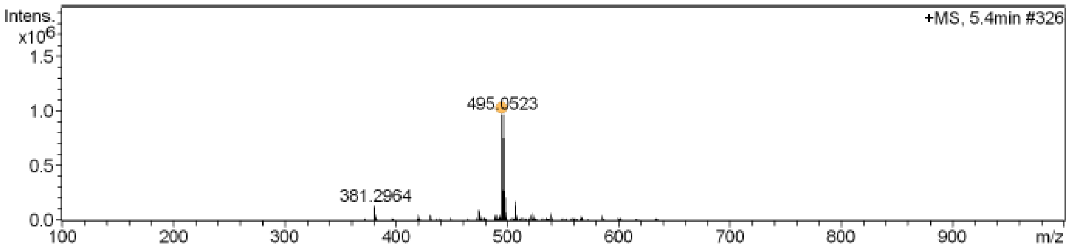

Meas. m/z	#	Ion Formula	m/z	err [ppm]	mSigma	# Sigma	Score	rdb	e⁻ Conf	N-Rule
495.0523	1	C22H21BrN2NaO5	495.0526	0.5	25.2	2	100.00	12.5	even	ok

Figure S28. HRMS (ESI-TOF) spectrum of ethyl 4-bromo-3-(4-methoxyphenyl)-1-[2-(4-methoxyphenyl)-2-oxoethyl]-1*H*-pyrazole-5-carboxylate (**4g**).

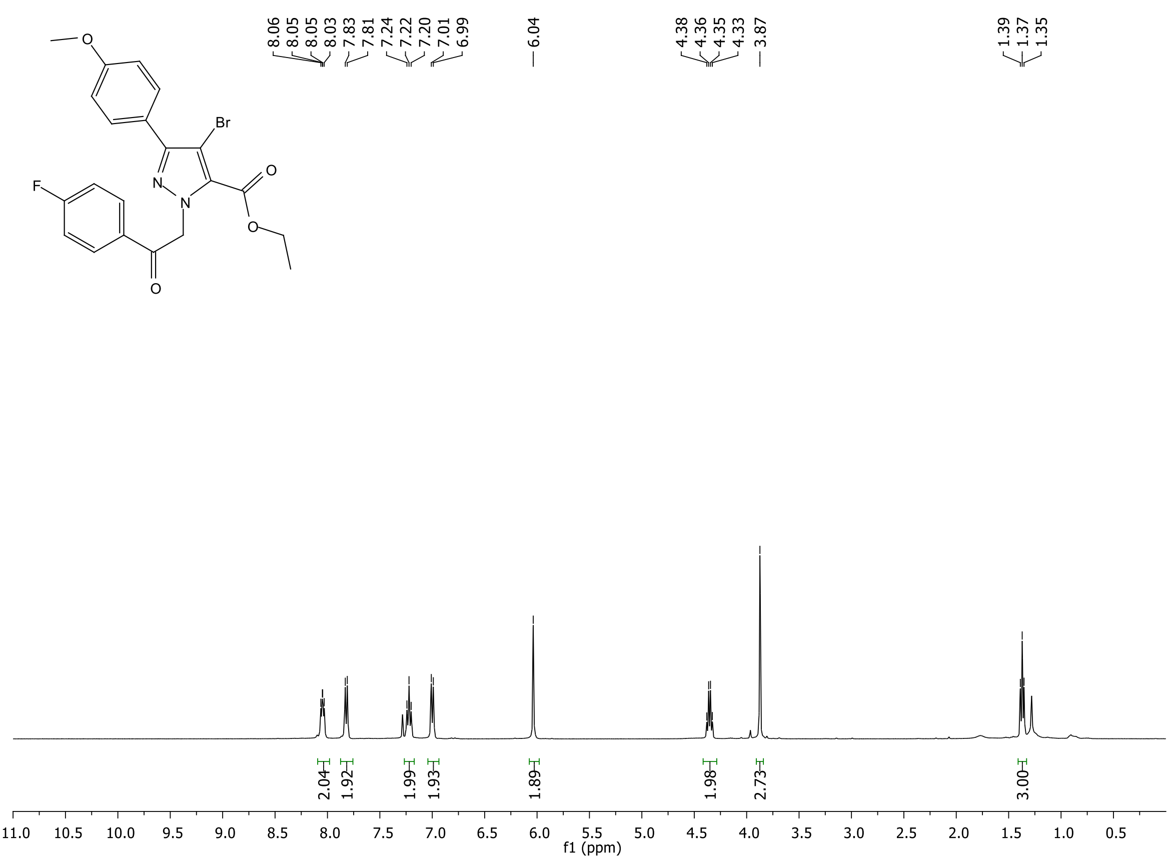

Figure S29. ^{1}H NMR spectrum (400 MHz, $CDCl_3$) of ethyl 4-bromo-1-[2-(4-fluorophenyl)-2-oxoethyl]-3-(4-methoxyphenyl)-1*H*-pyrazole-5-carboxylate (**4h**).

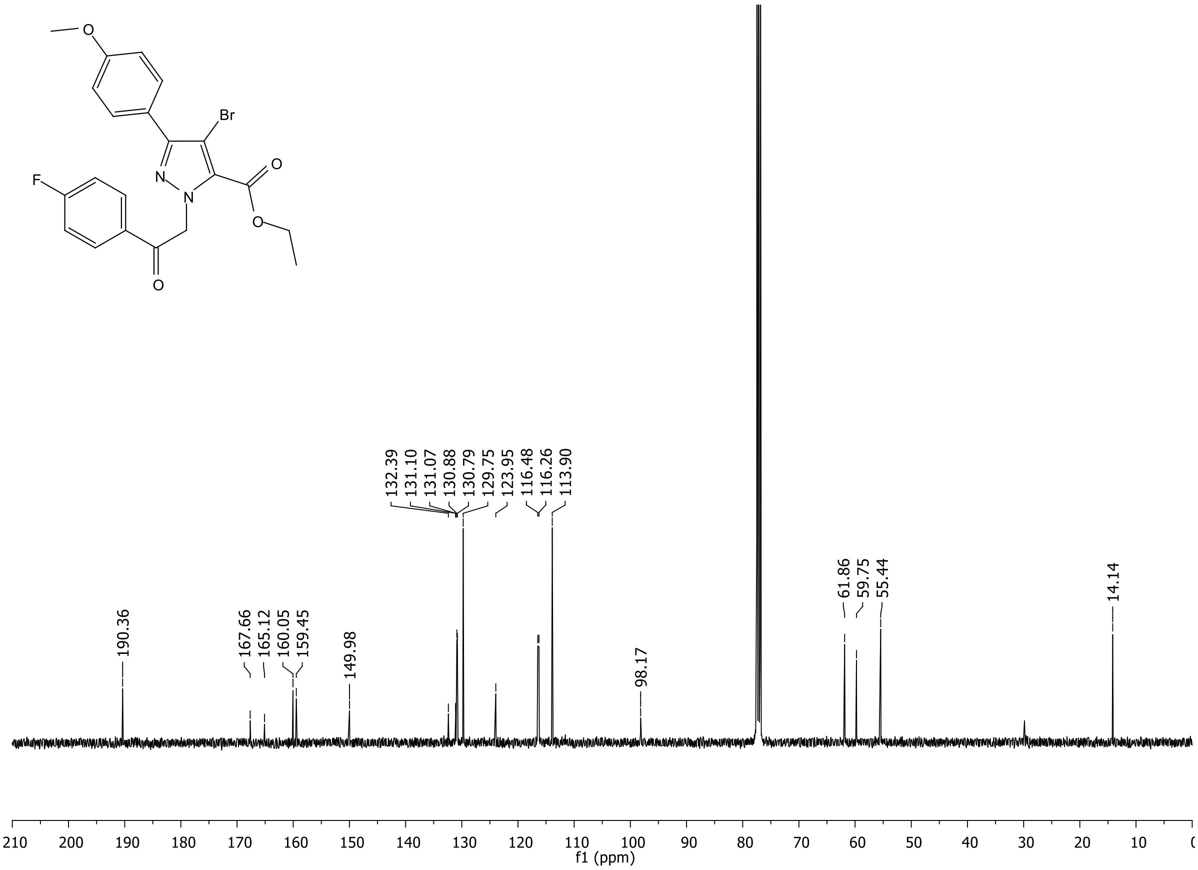

Figure S30. ^{13}C NMR spectrum (101 MHz, $CDCl_3$) of ethyl 4-bromo-1-[2-(4-fluorophenyl)-2-oxoethyl]-3-(4-methoxyphenyl)-1*H*-pyrazole-5-carboxylate (**4h**).

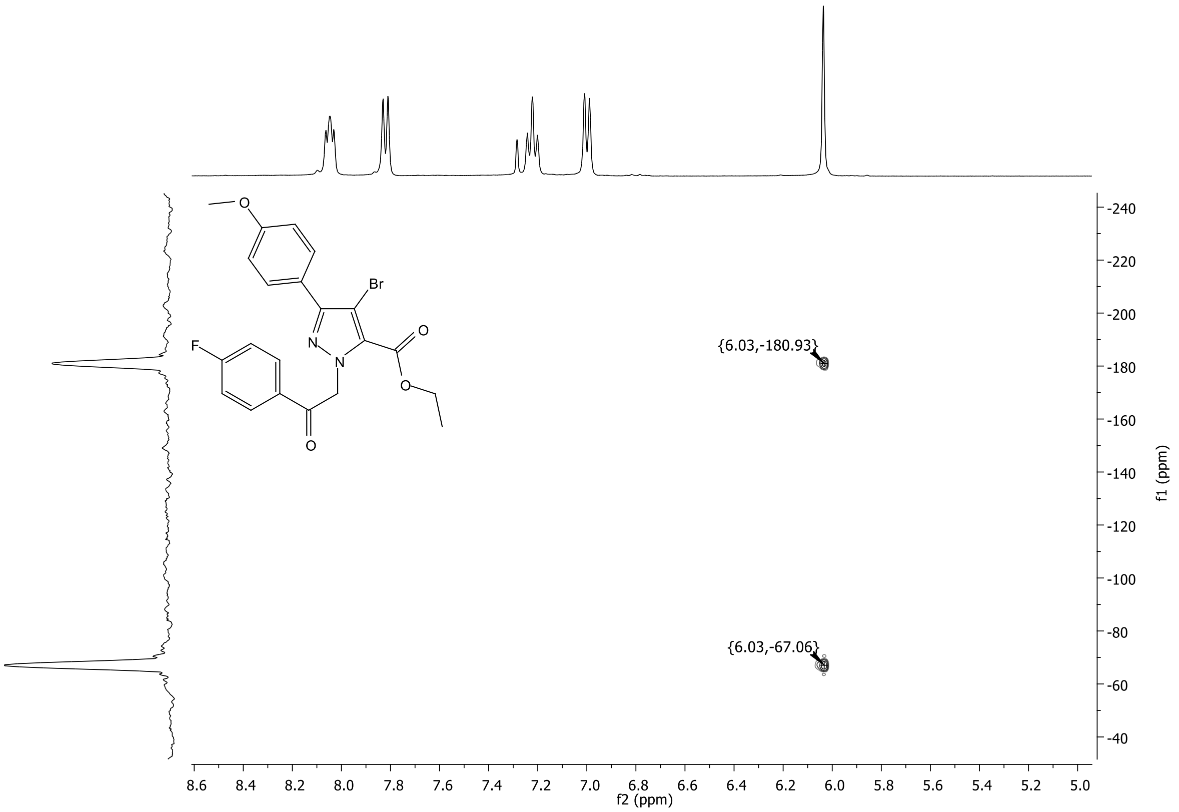

Figure S31. $^{1}H,^{15}N$-HMBC NMR spectrum (40 MHz, $CDCl_3$) of ethyl 4-bromo-1-[2-(4-fluorophenyl)-2-oxoethyl]-3-(4-methoxyphenyl)-1*H*-pyrazole-5-carboxylate (**4h**).

Compound Spectrum SmartFormula Report

Analysis Info

Analysis Name	D:\Data\KDD-327.d	Acquisition Date	6/17/2022 10:52:24 PM
Method	DirectInfusion_TuneLow_pos.m	Operator	hplc
Sample Name	KDD-327	Instrument	micrOTOF-Q III 8228888.20448
Comment	AB		

Acquisition Parameter

Source Type	ESI	Ion Polarity	Positive	Set Nebulizer	0.4 Bar
Focus	Not active	Set Capillary	4500 V	Set Dry Heater	180 °C
Scan Begin	50 m/z	Set End Plate Offset	-500 V	Set Dry Gas	4.0 l/min
Scan End	1000 m/z	Set Collision Cell RF	140.0 Vpp	Set Divert Valve	Waste

#	RT [min]	Area	Int. Type	I	S/N	Chromatogram	Max. m/z	FWHM [min]
n.a.	3.9	n.a.	Single spectrum	n.a.	n.a.	n.a.	485.0315	n.a.

+MS, 3.9min #235

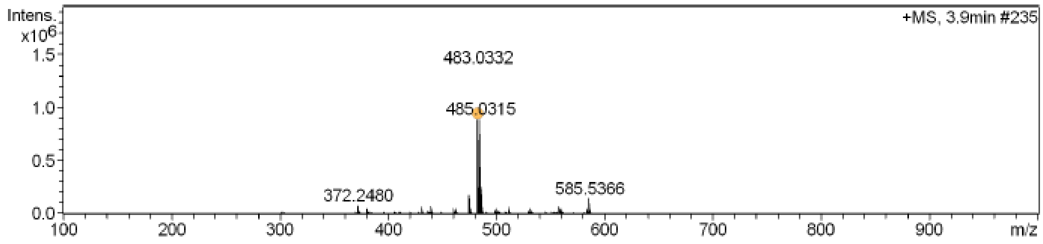

Meas. m/z	#	Ion Formula	m/z	err [ppm]	mSigma	# Sigma	Score	rdb	e⁻ Conf	N-Rule
483.0332	1	C21H18BrFN2NaO4	483.0326	-1.2	19.3	4	100.00	12.5	even	ok

Figure S32. HRMS (ESI-TOF) spectrum of ethyl 4-bromo-1-[2-(4-fluorophenyl)-2-oxoethyl]-3-(4-methoxyphenyl)-1*H*-pyrazole-5-carboxylate (**4h**).

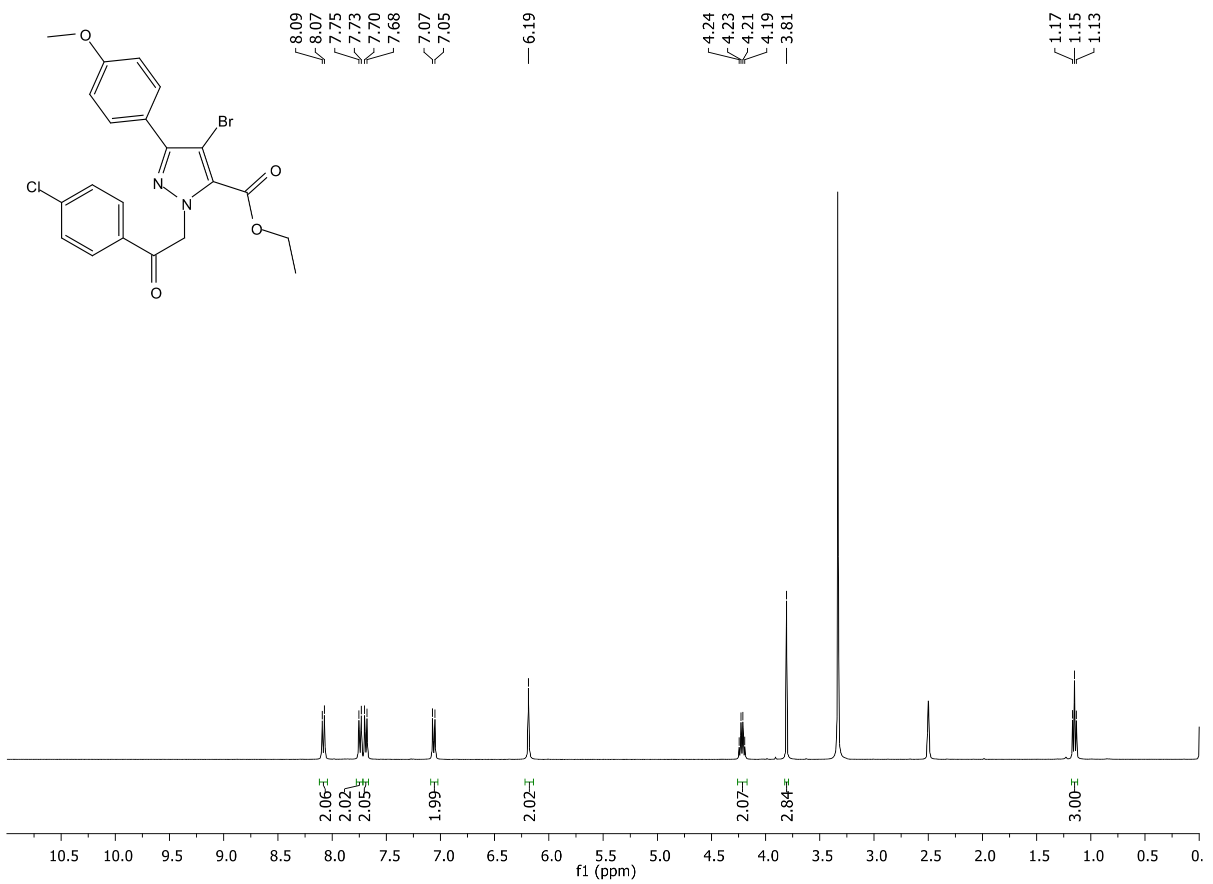

Figure S33. ^{1}H NMR spectrum (400 MHz, DMSO-d_6) of ethyl 4-bromo-1-[2-(4-chlorophenyl)-2-oxoethyl]-3-(4-methoxyphenyl)-1*H*-pyrazole-5-carboxylate (**4i**).

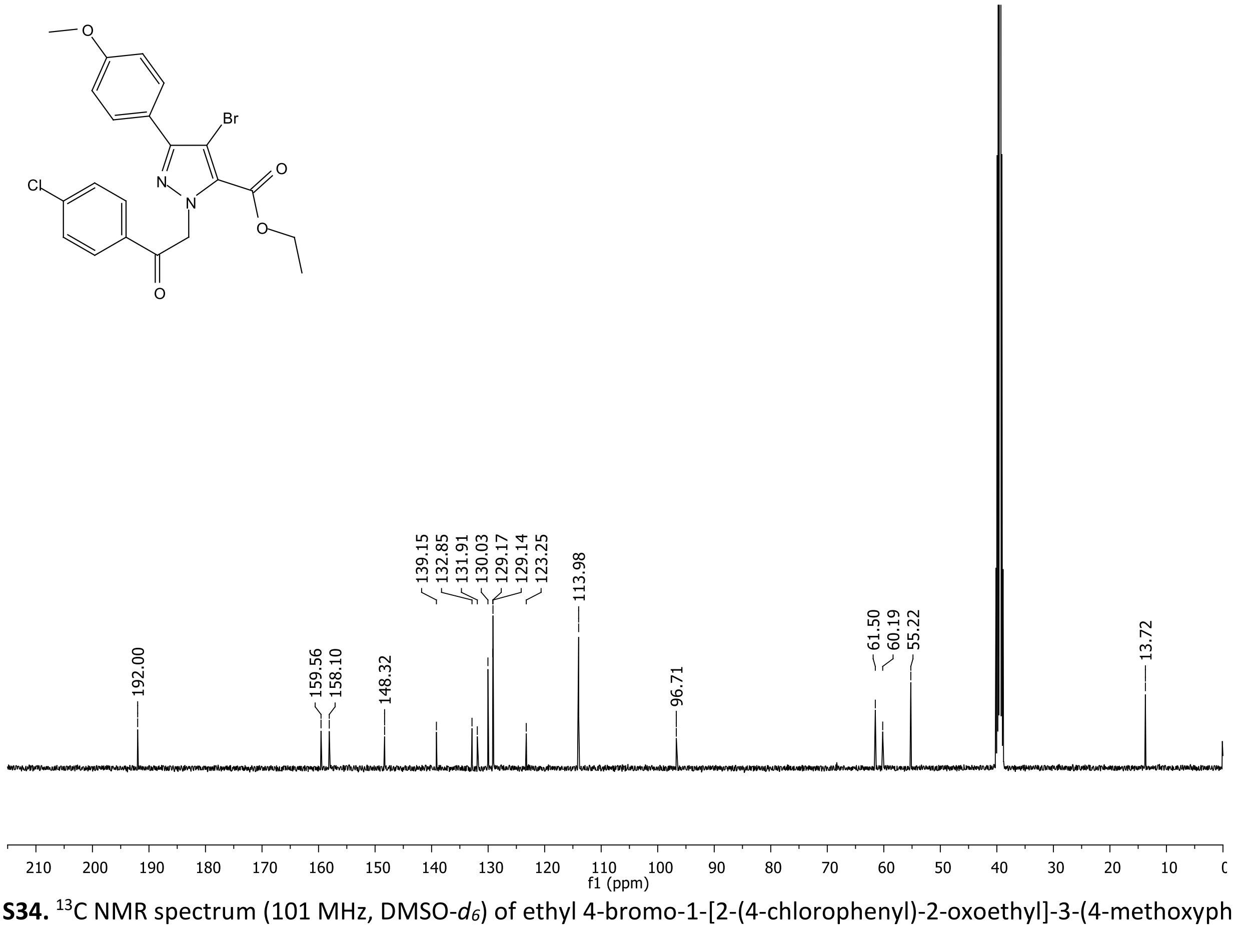

Figure S34. ^{13}C NMR spectrum (101 MHz, DMSO-d_6) of ethyl 4-bromo-1-[2-(4-chlorophenyl)-2-oxoethyl]-3-(4-methoxyphenyl)-1*H*-pyrazole-5-carboxylate (**4i**).

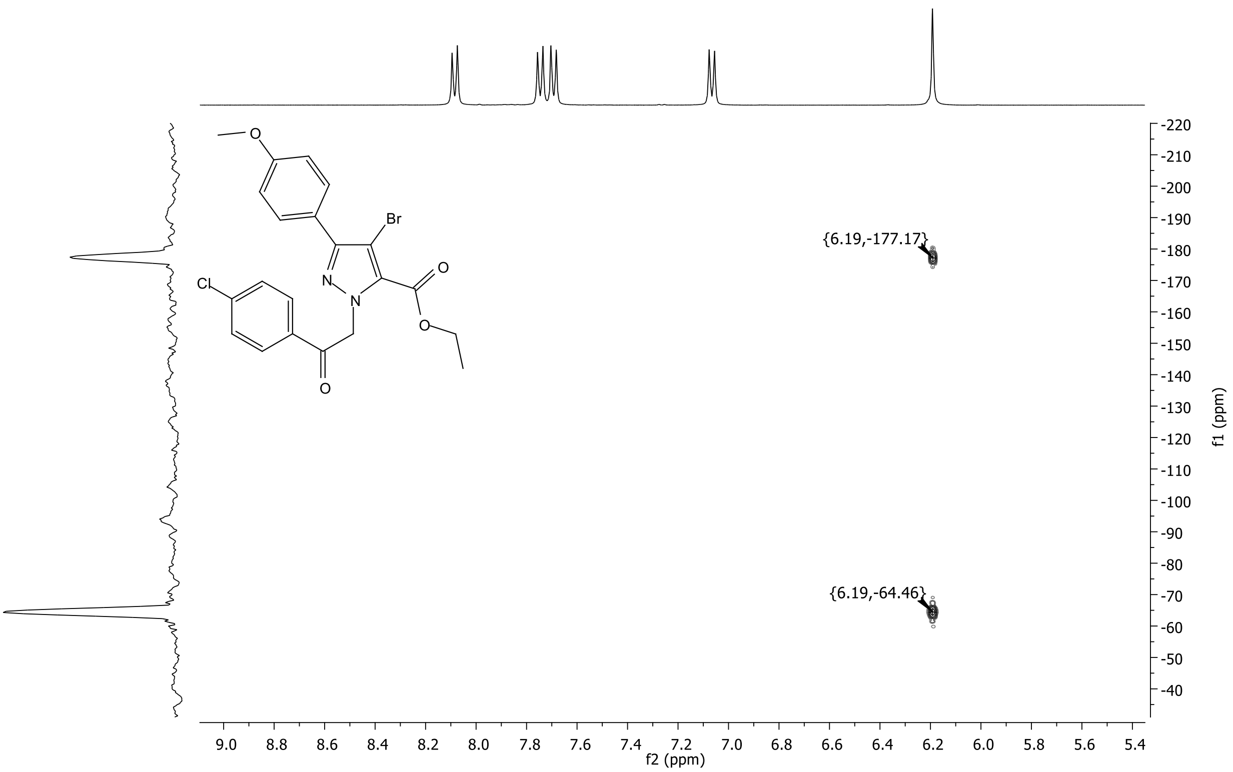

Figure S35. $^{1}H,^{15}N$-HMBC NMR spectrum (40 MHz, DMSO-d_6) of ethyl 4-bromo-1-[2-(4-chlorophenyl)-2-oxoethyl]-3-(4-methoxyphenyl)-1*H*-pyrazole-5-carboxylate (**4i**).

Compound Spectrum SmartFormula Report

Analysis Info

Analysis Name	D:\Data\KDD-307.d	Acquisition Date	6/17/2022 1:27:56 PM
Method	DirectInfusion_TuneLow_pos.m	Operator	hplc
Sample Name	KDD-307	Instrument	micrOTOF-Q III 8228888.20448
Comment	AB		

Acquisition Parameter

Source Type	ESI	Ion Polarity	Positive	Set Nebulizer	0.4 Bar
Focus	Not active	Set Capillary	4500 V	Set Dry Heater	180 °C
Scan Begin	50 m/z	Set End Plate Offset	-500 V	Set Dry Gas	4.0 l/min
Scan End	1000 m/z	Set Collision Cell RF	140.0 Vpp	Set Divert Valve	Waste

#	RT [min]	Area	Int. Type	I	S/N	Chromatogram	Max. m/z	FWHM [min]
n.a.	6.4	n.a.	Single spectrum	n.a.	n.a.	n.a.	501.0003	n.a.

+MS, 6.4min #381

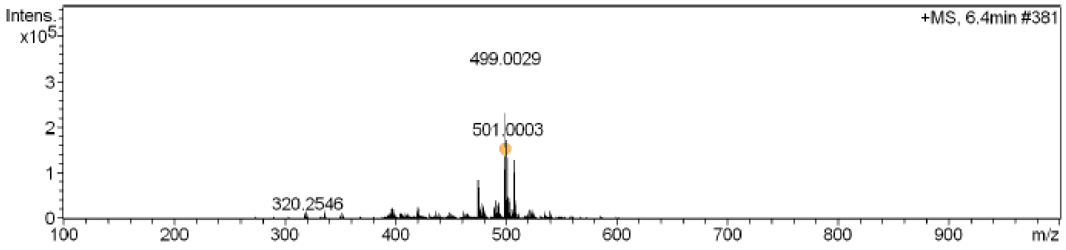

Meas. m/z	#	Ion Formula	m/z	err [ppm]	mSigma	# Sigma	Score	rdb	e⁻ Conf	N-Rule
499.0029	1	C21H18BrClN2NaO4	499.0031	0.3	19.1	1	100.00	12.5	even	ok

Figure S36. HRMS (ESI-TOF) spectrum of ethyl 4-bromo-1-[2-(4-chlorophenyl)-2-oxoethyl]-3-(4-methoxyphenyl)-1*H*-pyrazole-5-carboxylate (**4i**).

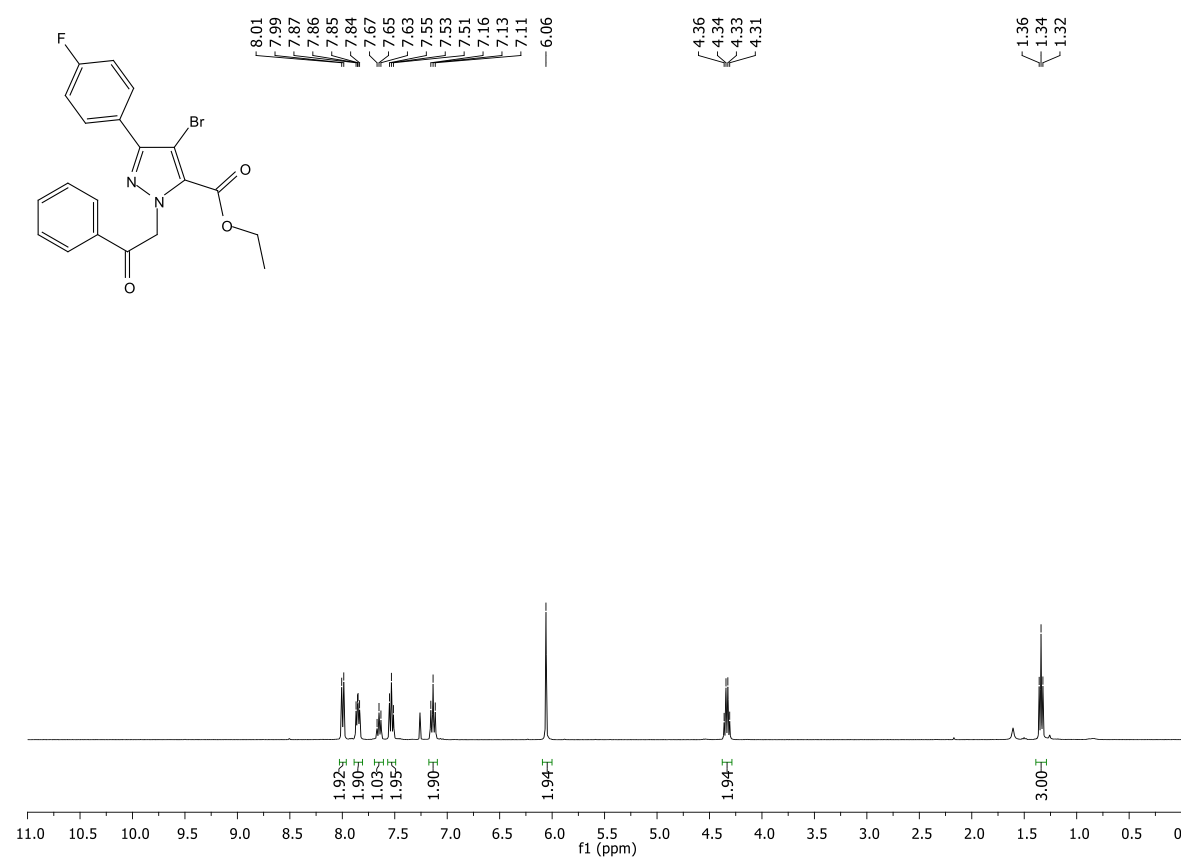

Figure S37. ^{1}H NMR spectrum (400 MHz, $CDCl_3$) of ethyl 4-bromo-3-(4-fluorophenyl)-1-(2-oxo-2-phenylethyl)-1*H*-pyrazole-5-carboxylate (**4j**).

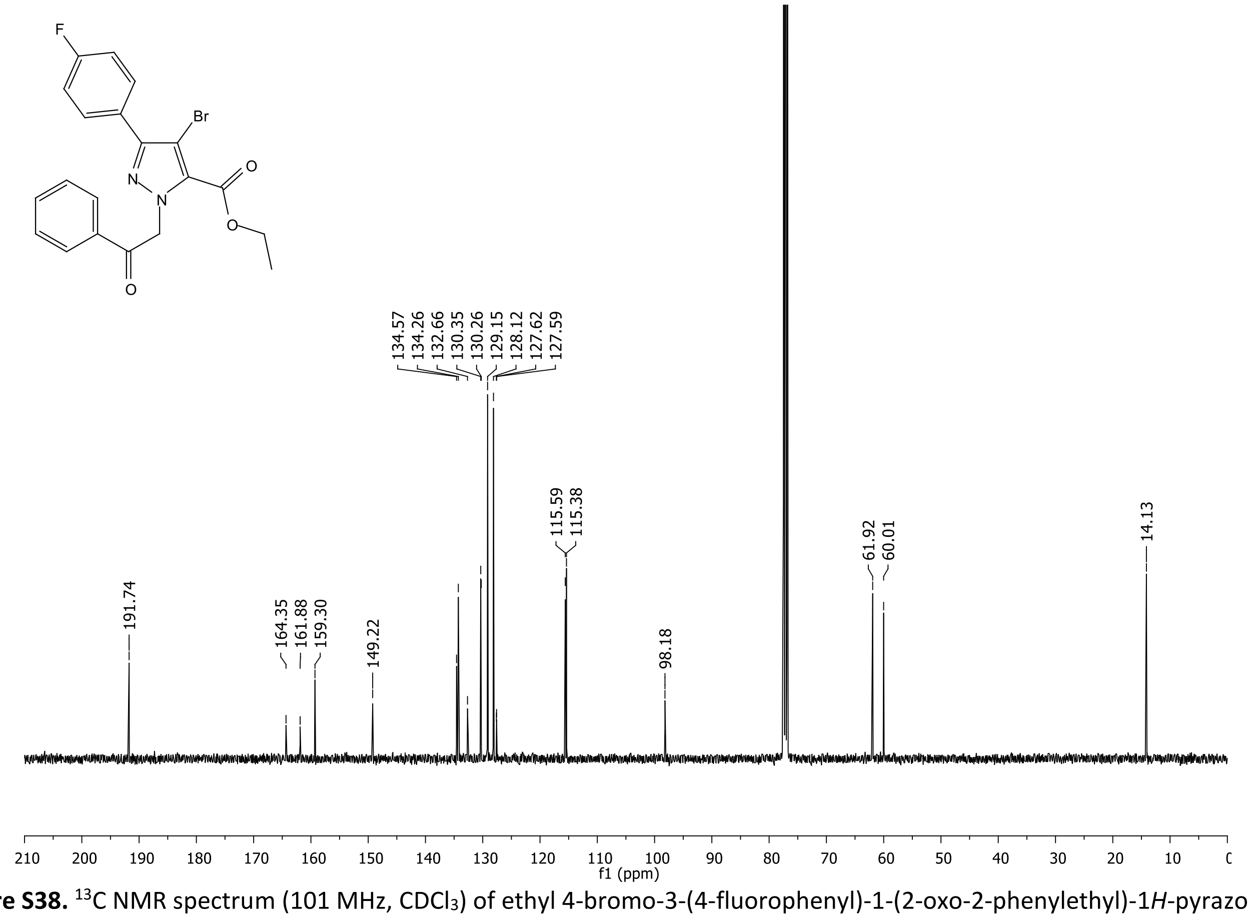

Figure S38. ^{13}C NMR spectrum (101 MHz, $CDCl_3$) of ethyl 4-bromo-3-(4-fluorophenyl)-1-(2-oxo-2-phenylethyl)-1*H*-pyrazole-5-carboxylate (**4j**).

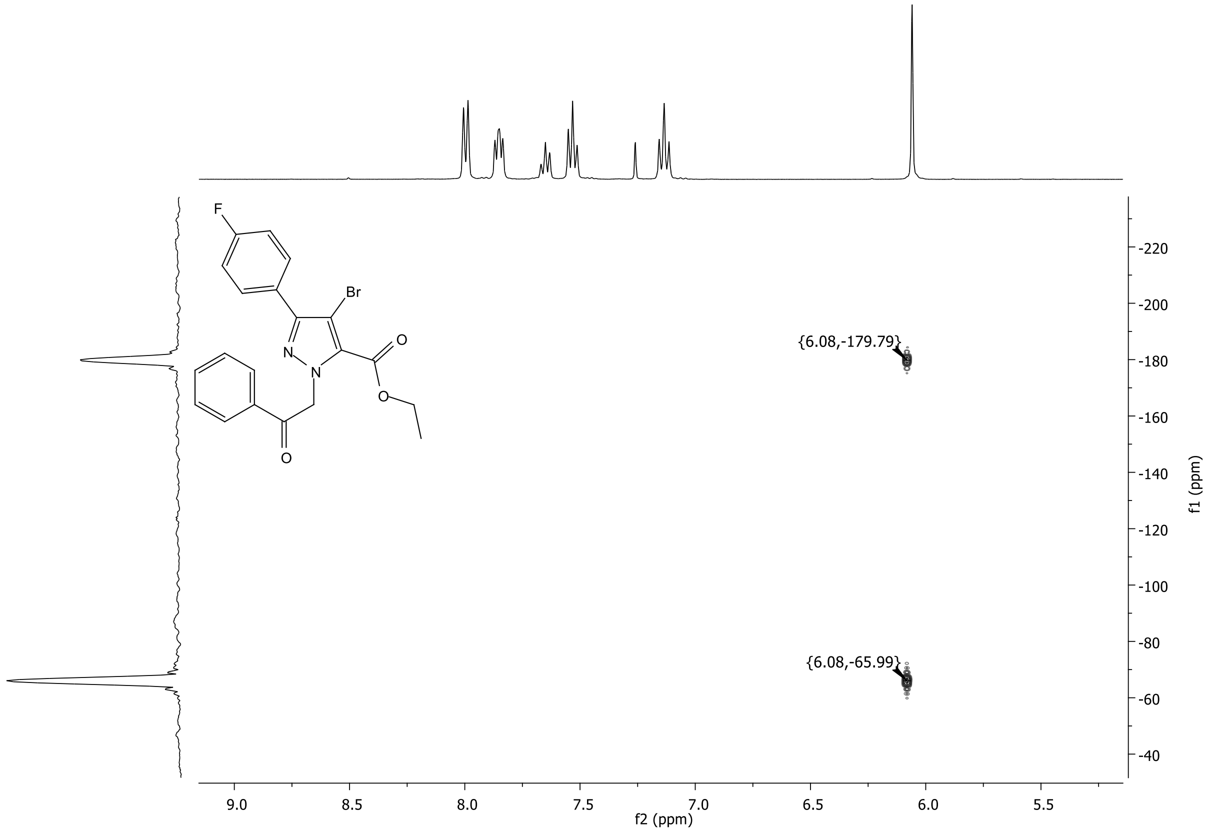

Figure S39. $^{1}H,^{15}N$-HMBC NMR spectrum (40 MHz, $CDCl_3$) of ethyl 4-bromo-3-(4-fluorophenyl)-1-(2-oxo-2-phenylethyl)-1*H*-pyrazole-5-carboxylate (**4j**).

Compound Spectrum SmartFormula Report

Analysis Info

Analysis Name	D:\Data\KDD-373.d	Acquisition Date	6/17/2022 11:11:08 PM
Method	DirectInfusion_TuneLow_pos.m	Operator	hplc
Sample Name	KDD-373	Instrument	micrOTOF-Q III 8228888.20448
Comment	AB		

Acquisition Parameter

Source Type	ESI	Ion Polarity	Positive	Set Nebulizer	0.4 Bar
Focus	Not active	Set Capillary	4500 V	Set Dry Heater	180 °C
Scan Begin	50 m/z	Set End Plate Offset	-500 V	Set Dry Gas	4.0 l/min
Scan End	1000 m/z	Set Collision Cell RF	140.0 Vpp	Set Divert Valve	Waste

#	RT [min]	Area	Int. Type	I	S/N	Chromatogram	Max. m/z	FWHM [min]
n.a.	4.4	n.a.	Single spectrum	n.a.	n.a.	n.a.	453.0225	n.a.

+MS, 4.4min #262

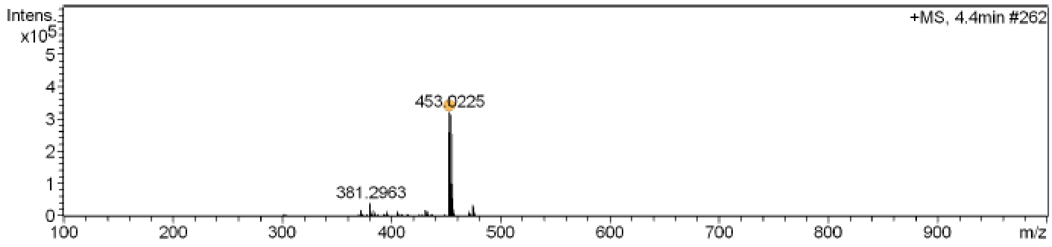

Meas. m/z	#	Ion Formula	m/z	err [ppm]	mSigma	# Sigma	Score	rdb	e⁻ Conf	N-Rule
453.0225	1	C20H16BrFN2NaO3	453.0221	1.1	12.3	1	100.00	12.5	even	ok

Figure S40. HRMS (ESI-TOF) spectrum of ethyl 4-bromo-3-(4-fluorophenyl)-1-(2-oxo-2-phenylethyl)-1*H*-pyrazole-5-carboxylate (**4j**).

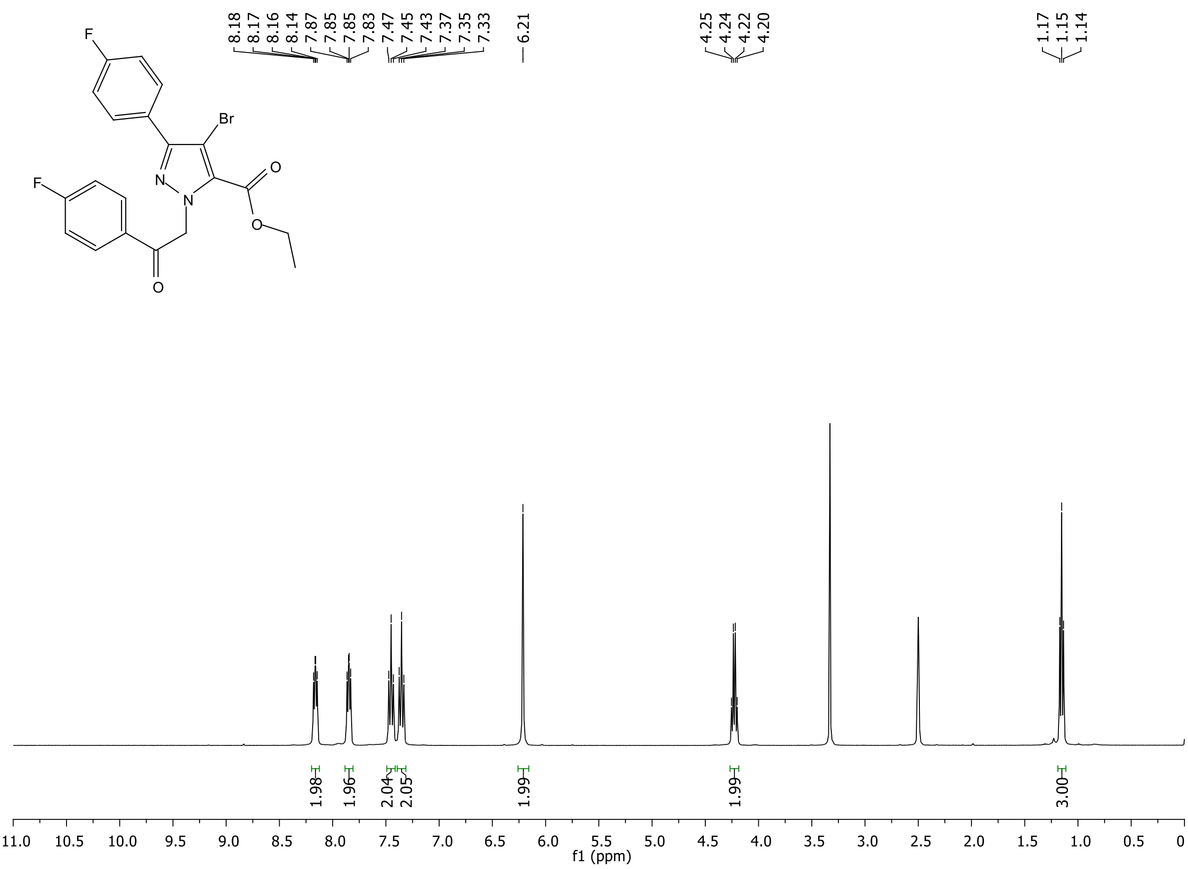

Figure S41. ^{1}H NMR spectrum (400 MHz, DMSO-d_6) of ethyl 4-bromo-3-(4-fluorophenyl)-1-[2-(4-fluorophenyl)-2-oxoethyl]-1*H*-pyrazole-5-carboxylate (**4k**).

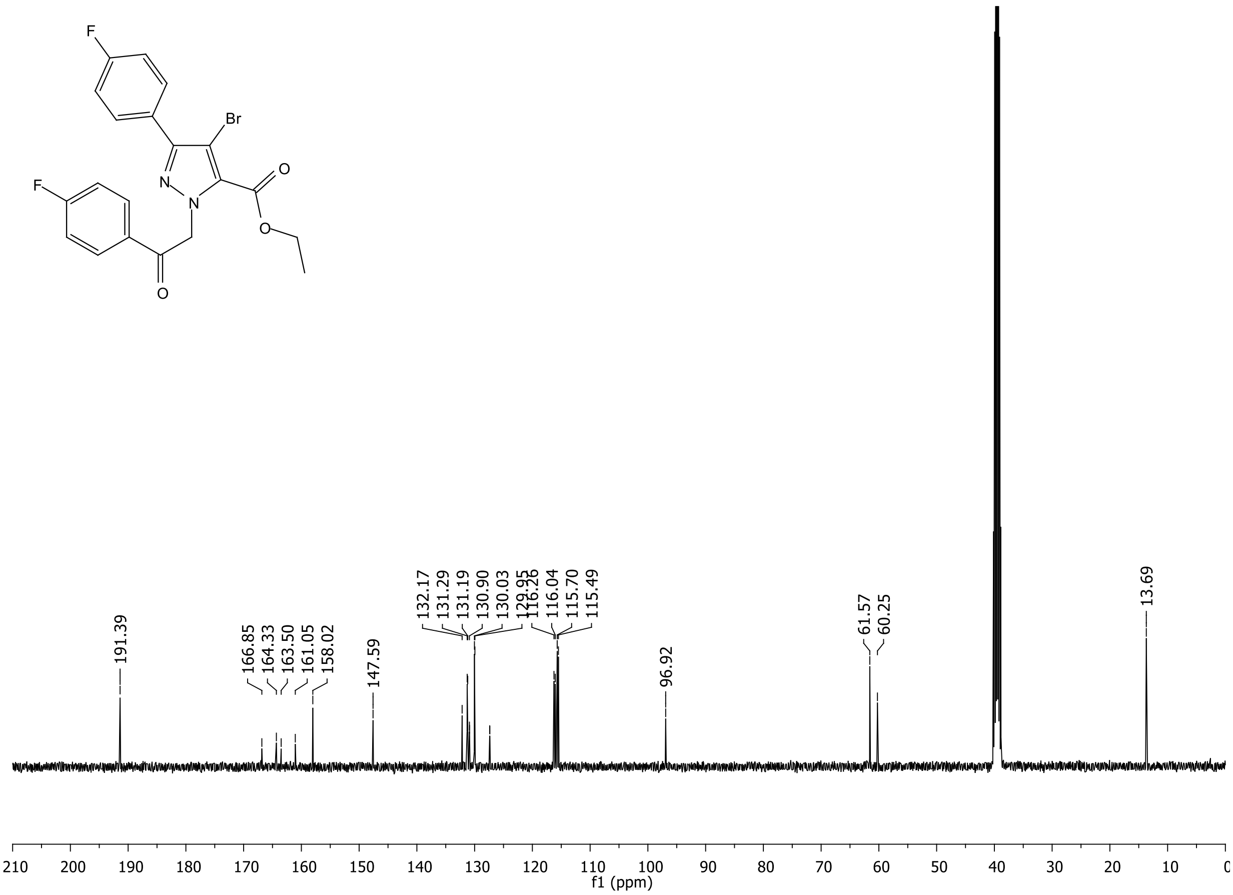

Figure S42. ^{13}C NMR spectrum (101 MHz, DMSO-d_6) of ethyl 4-bromo-3-(4-fluorophenyl)-1-[2-(4-fluorophenyl)-2-oxoethyl]-1*H*-pyrazole-5-carboxylate (**4k**).

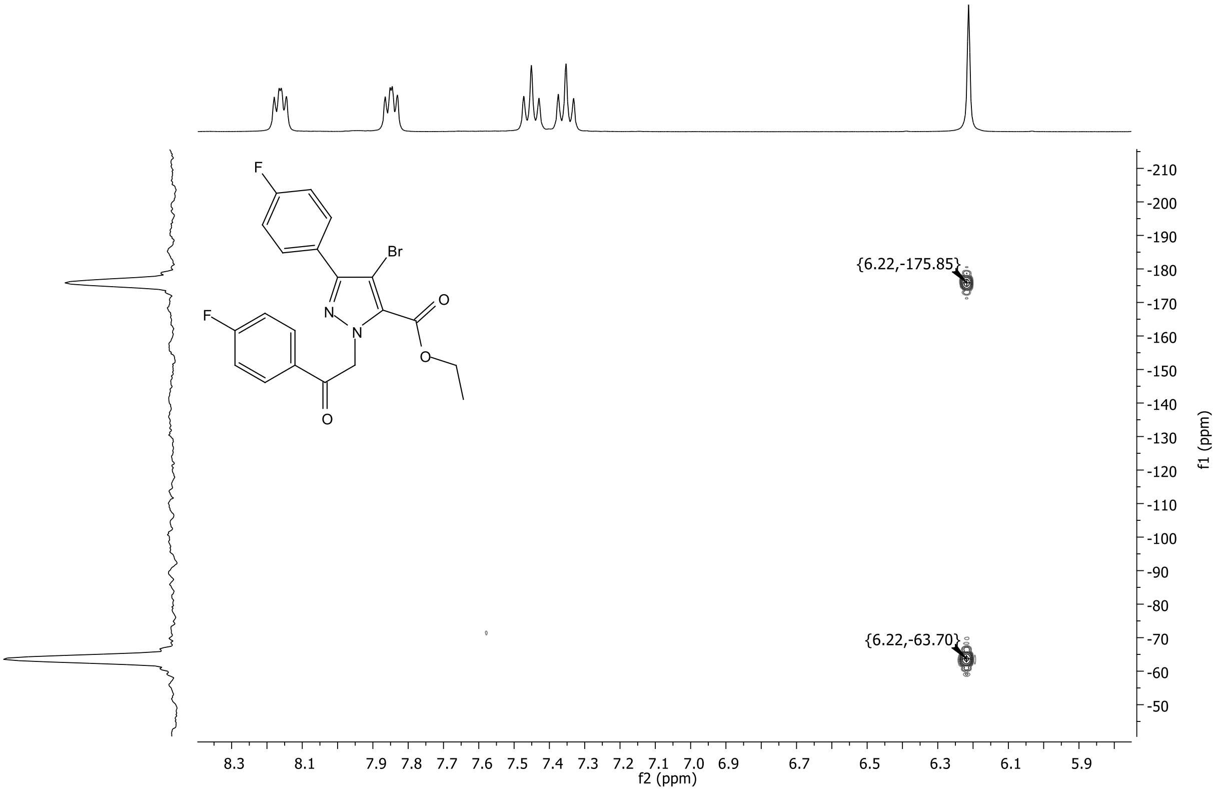

Figure S43. $^{1}H,^{15}N$-HMBC NMR spectrum (40 MHz, DMSO-d_6) of ethyl 4-bromo-3-(4-fluorophenyl)-1-[2-(4-fluorophenyl)-2-oxoethyl]-1*H*-pyrazole-5-carboxylate (**4k**).

Compound Spectrum SmartFormula Report

Analysis Info

Analysis Name	D:\Data\KDD-377.d	Acquisition Date	6/17/2022 11:25:21 PM
Method	DirectInfusion_TuneLow_pos.m	Operator	hplc
Sample Name	KDD-377	Instrument	micrOTOF-Q III 8228888.20448
Comment	AB		

Acquisition Parameter

Source Type	ESI	Ion Polarity	Positive	Set Nebulizer	0.4 Bar
Focus	Not active	Set Capillary	4500 V	Set Dry Heater	180 °C
Scan Begin	50 m/z	Set End Plate Offset	-500 V	Set Dry Gas	4.0 l/min
Scan End	1000 m/z	Set Collision Cell RF	140.0 Vpp	Set Divert Valve	Waste

#	RT [min]	Area	Int. Type	I	S/N	Chromatogram	Max. m/z	FWHM [min]
n.a.	5.4	n.a.	Single spectrum	n.a.	n.a.	n.a.	471.0130	n.a.

+MS, 5.4min #324

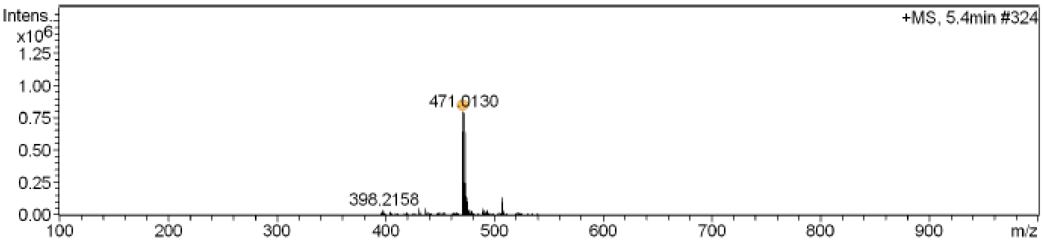

Meas. m/z	#	Ion Formula	m/z	err [ppm]	mSigma	# Sigma	Score	rdb	e¯ Conf	N-Rule
471.0130	1	C20H15BrF2N2NaO3	471.0126	-0.7	14.3	2	100.00	12.5	even	ok

Figure S44. HRMS (ESI-TOF) spectrum of ethyl 4-bromo-3-(4-fluorophenyl)-1-[2-(4-fluorophenyl)-2-oxoethyl]-1*H*-pyrazole-5-carboxylate (**4k**).

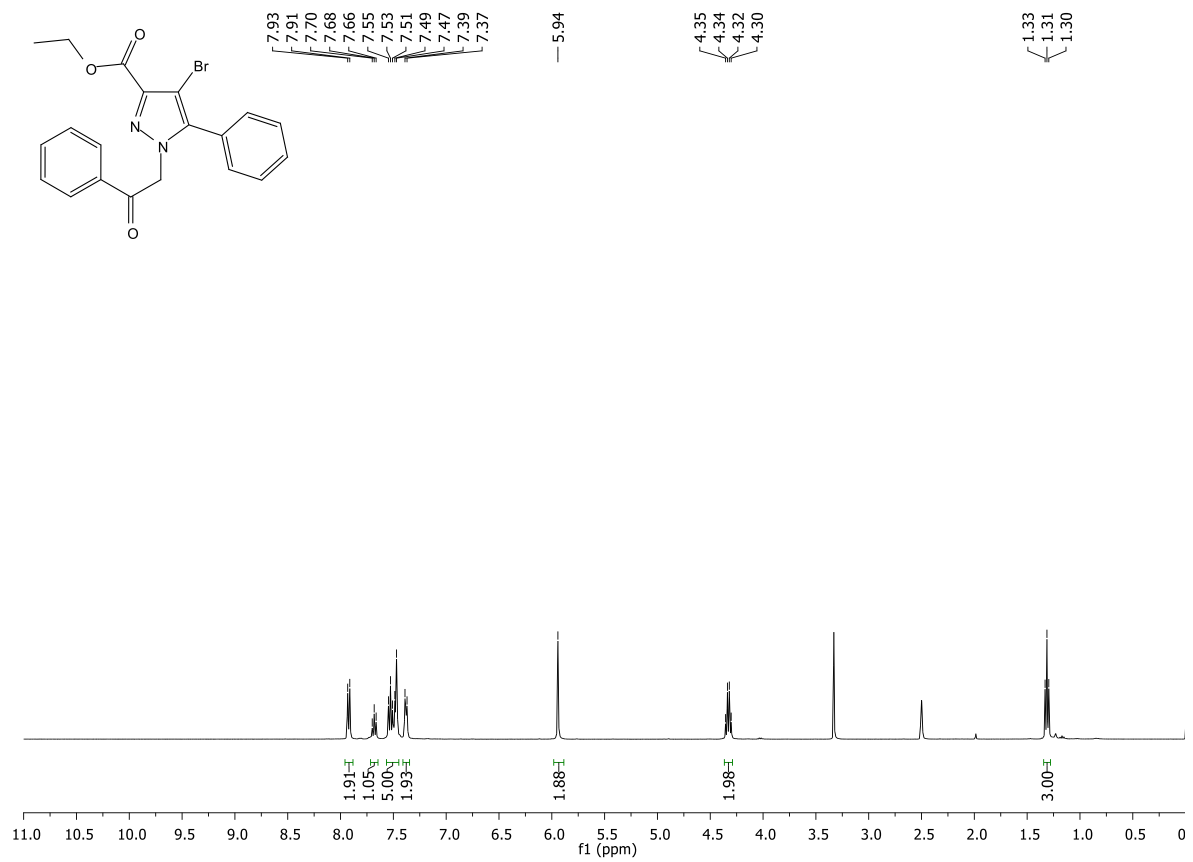

Figure S45. ^{1}H NMR spectrum (400 MHz, DMSO-d_6) of ethyl 4-bromo-1-(2-oxo-2-phenylethyl)-5-phenyl-1*H*-pyrazole-3-carboxylate (**5a**).

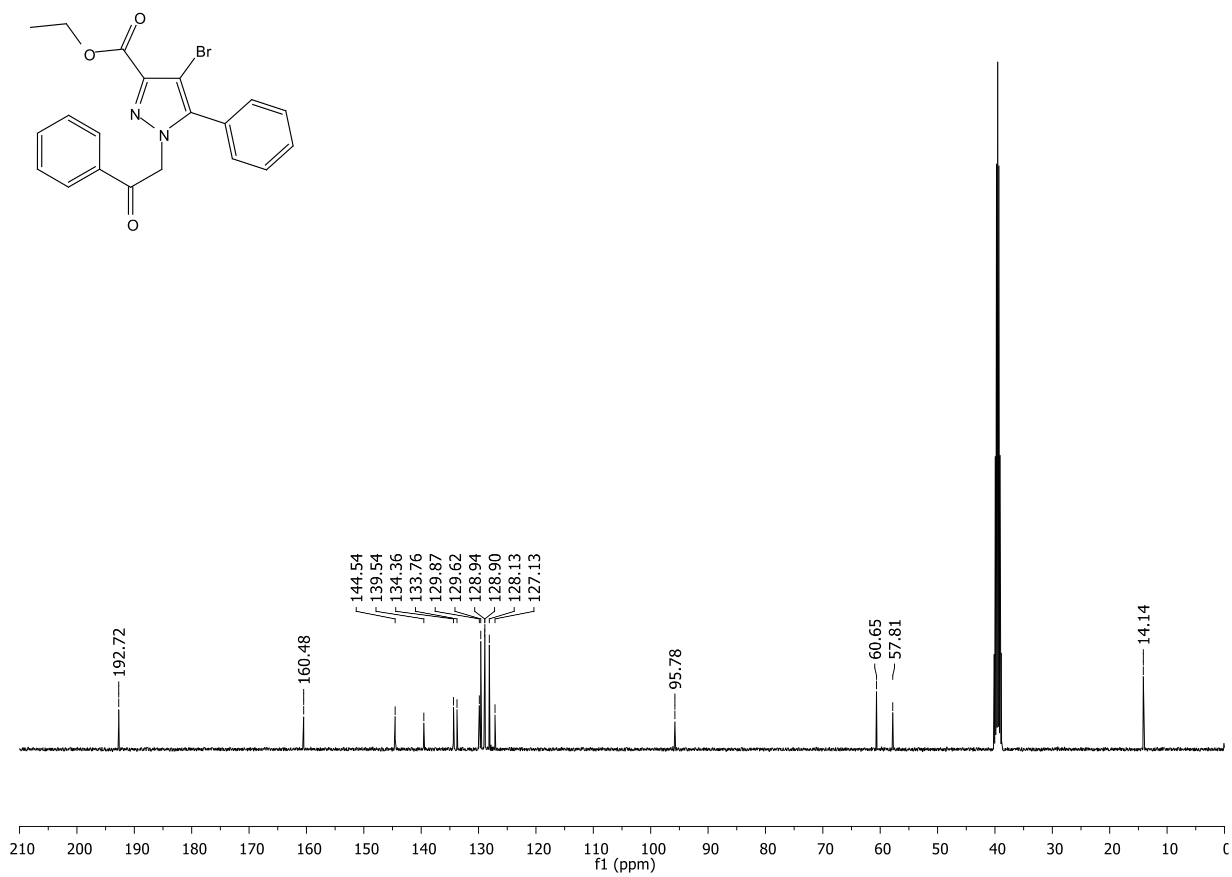

Figure S46. ^{13}C NMR spectrum (101 MHz, DMSO-d_6) of ethyl 4-bromo-1-(2-oxo-2-phenylethyl)-5-phenyl-1*H*-pyrazole-3-carboxylate (**5a**).

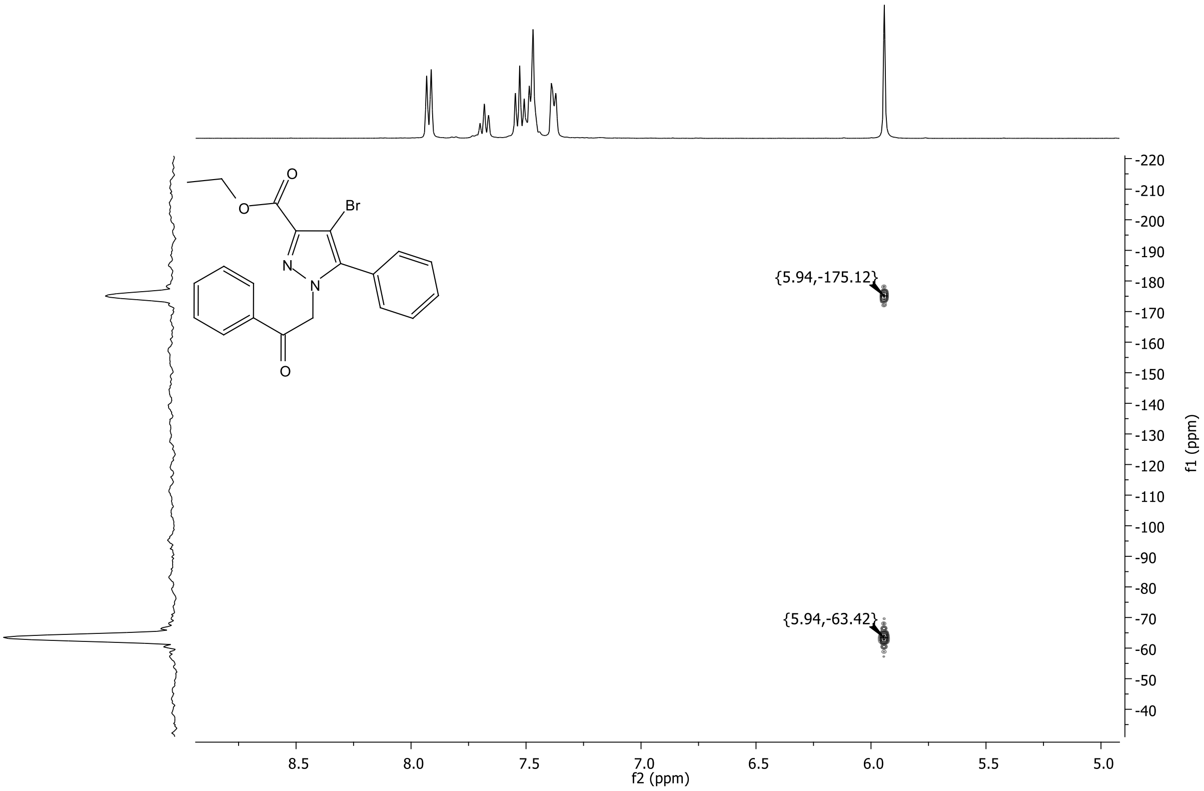

Figure S47. $^{1}H,^{15}N$-HMBC NMR spectrum (40 MHz, DMSO-d_6) of ethyl 4-bromo-1-(2-oxo-2-phenylethyl)-5-phenyl-1*H*-pyrazole-3-carboxylate (**5a**).

Compound Spectrum SmartFormula Report

Analysis Info

Analysis Name	D:\Data\KDD-257-2.d	Acquisition Date	6/17/2022 12:27:49 PM
Method	DirectInfusion_TuneLow_pos.m	Operator	hplc
Sample Name	KDD-257-2	Instrument	micrOTOF-Q III 8228888.20448
Comment	AB		

Acquisition Parameter

Source Type	ESI	Ion Polarity	Positive	Set Nebulizer	0.4 Bar
Focus	Not active	Set Capillary	4500 V	Set Dry Heater	180 °C
Scan Begin	50 m/z	Set End Plate Offset	-500 V	Set Dry Gas	4.0 l/min
Scan End	1000 m/z	Set Collision Cell RF	140.0 Vpp	Set Divert Valve	Waste

#	RT [min]	Area	Int. Type	I	S/N	Chromatogram	Max. m/z	FWHM [min]
n.a.	3.4	n.a.	Single spectrum	n.a.	n.a.	n.a.	435.0313	n.a.

+MS, 3.4min #202

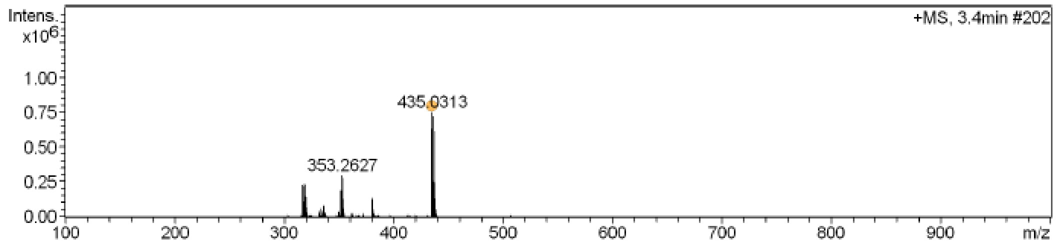

Meas. m/z	#	Ion Formula	m/z	err [ppm]	mSigma	# Sigma	Score	rdb	e⁻ Conf	N-Rule
435.0313	1	C20H17BrN2NaO3	435.0315	-0.4	19.0	1	100.00	12.5	even	ok

Figure S48. HRMS (ESI-TOF) spectrum of ethyl 4-bromo-1-(2-oxo-2-phenylethyl)-5-phenyl-1*H*-pyrazole-3-carboxylate (**5a**).

3. Analytical data of target compounds 6a′, 6a–o and 7a–c

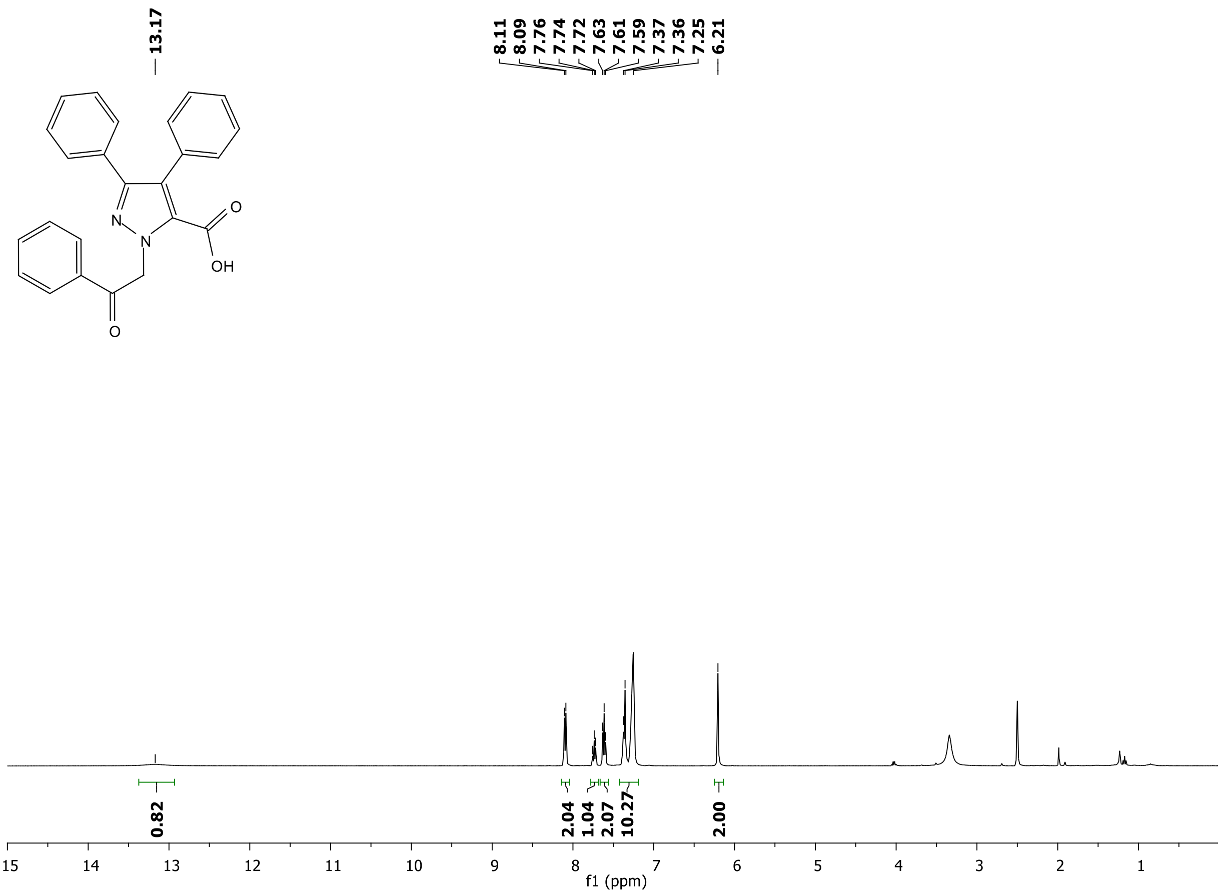

Figure S49. ^{1}H NMR spectrum (400 MHz, DMSO-d_6) of 1-(2-oxo-2-phenylethyl)-3,4-diphenyl-1*H*-pyrazole-5-carboxylic acid (**6a′**).

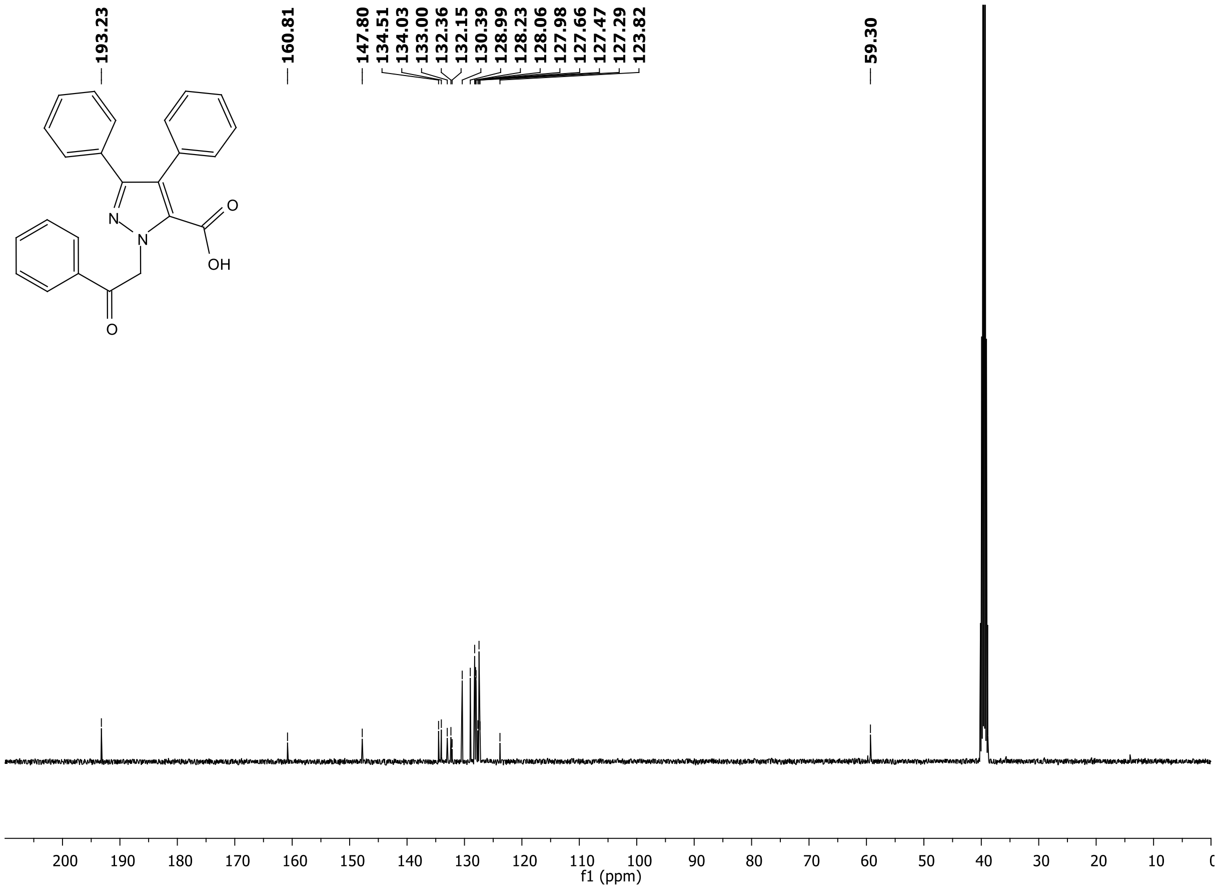

Figure S50. ^{13}C NMR spectrum (101 MHz, DMSO-d_6) of 1-(2-oxo-2-phenylethyl)-3,4-diphenyl-1*H*-pyrazole-5-carboxylic acid (**6a′**).

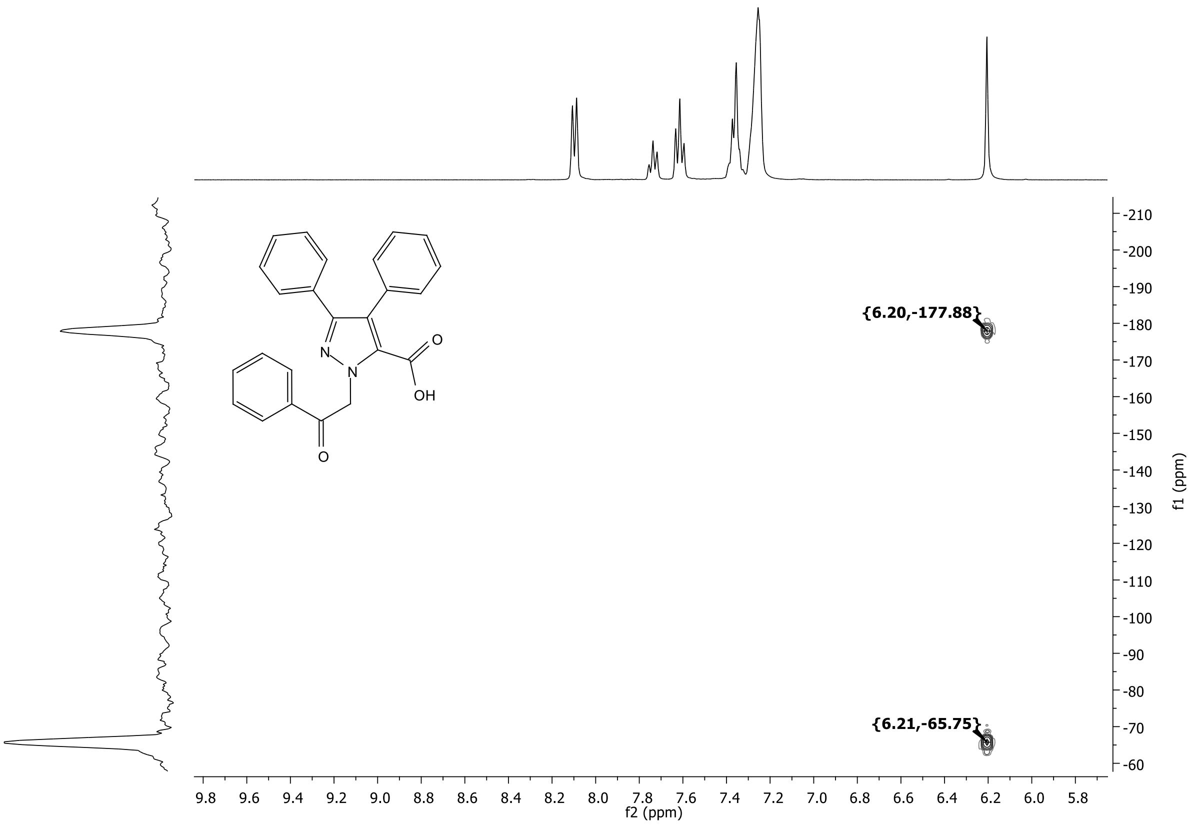

Figure S51. $^{1}H,^{15}N$-HMBC NMR spectrum (40 MHz, DMSO-d_6) of 1-(2-oxo-2-phenylethyl)-3,4-diphenyl-1*H*-pyrazole-5-carboxylic acid (**6a′**).

Qualitative Compound Report

**Data File**	221114_KDD-401_01.d	**Sample Name**	Unavailable
**Sample Type**	Unavailable	**Position**	Unavailable
**Instrument Name**	Unavailable	**User Name**	Unavailable
**Acq Method**		**Acquired Time**	Unavailable
**IRM Calibration Status**	Success	**DA Method**	test.m
**Comment**	Sample information is unavailable		

Compound Table

Compound Label	RT	Mass	Abund	Formula	Tgt Mass	Diff (ppm)
Cpd 1: C24 H18 N2 O3	7.711	382.1322	3003243	C24 H18 N2 O3	382.1317	1.13

Compound Label	m/z	RT	Algorithm	Mass
Cpd 1: C24 H18 N2 O3	383.1389	7.711	Find By Formula	382.1322

Compound Chromatograms

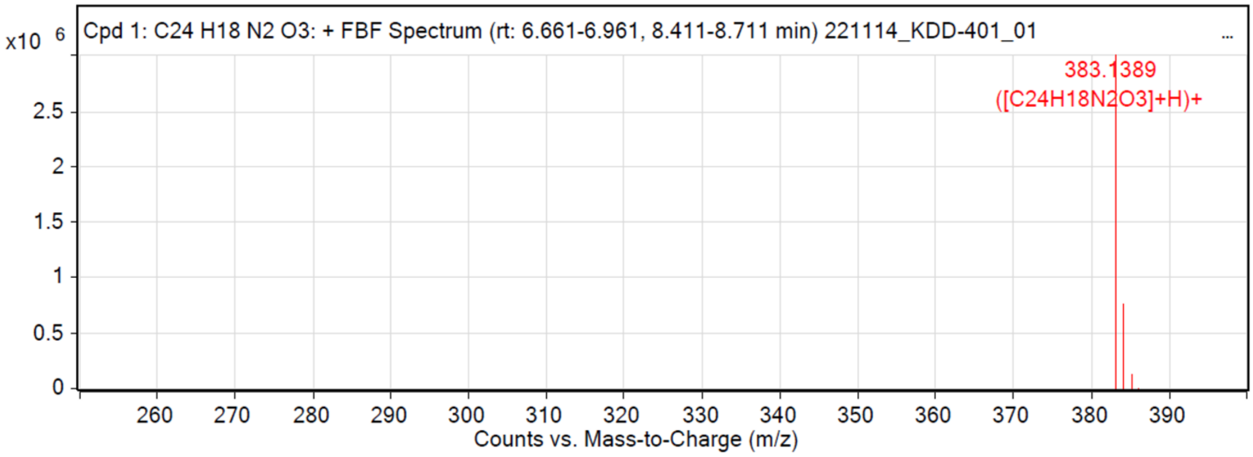

Figure S52. HRMS (ESI-TOF) spectrum of 1-(2-oxo-2-phenylethyl)-3,4-diphenyl-1*H*-pyrazole-5-carboxylic acid (**6a′**).

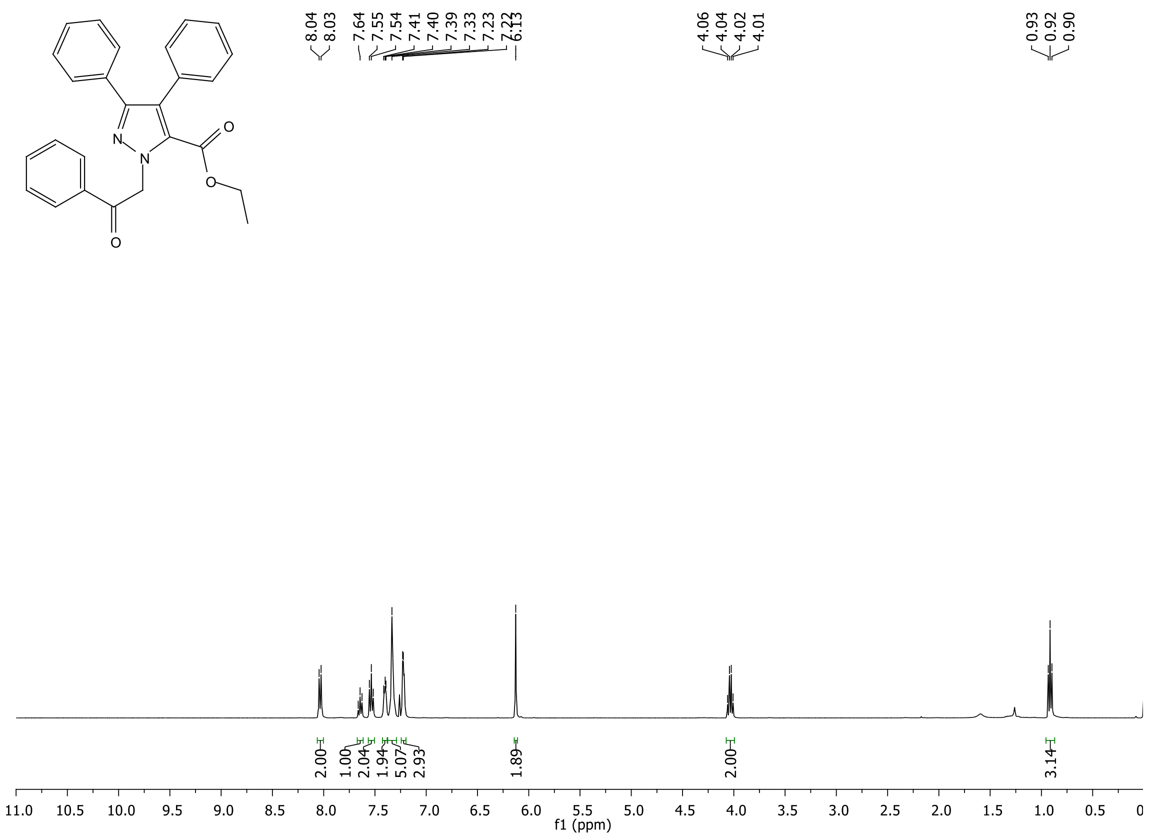

Figure S53. ^{1}H NMR spectrum (400 MHz, $CDCl_3$) of ethyl 1-(2-oxo-2-phenylethyl)-3,4-diphenyl-1*H*-pyrazole-5-carboxylate (**6a**).

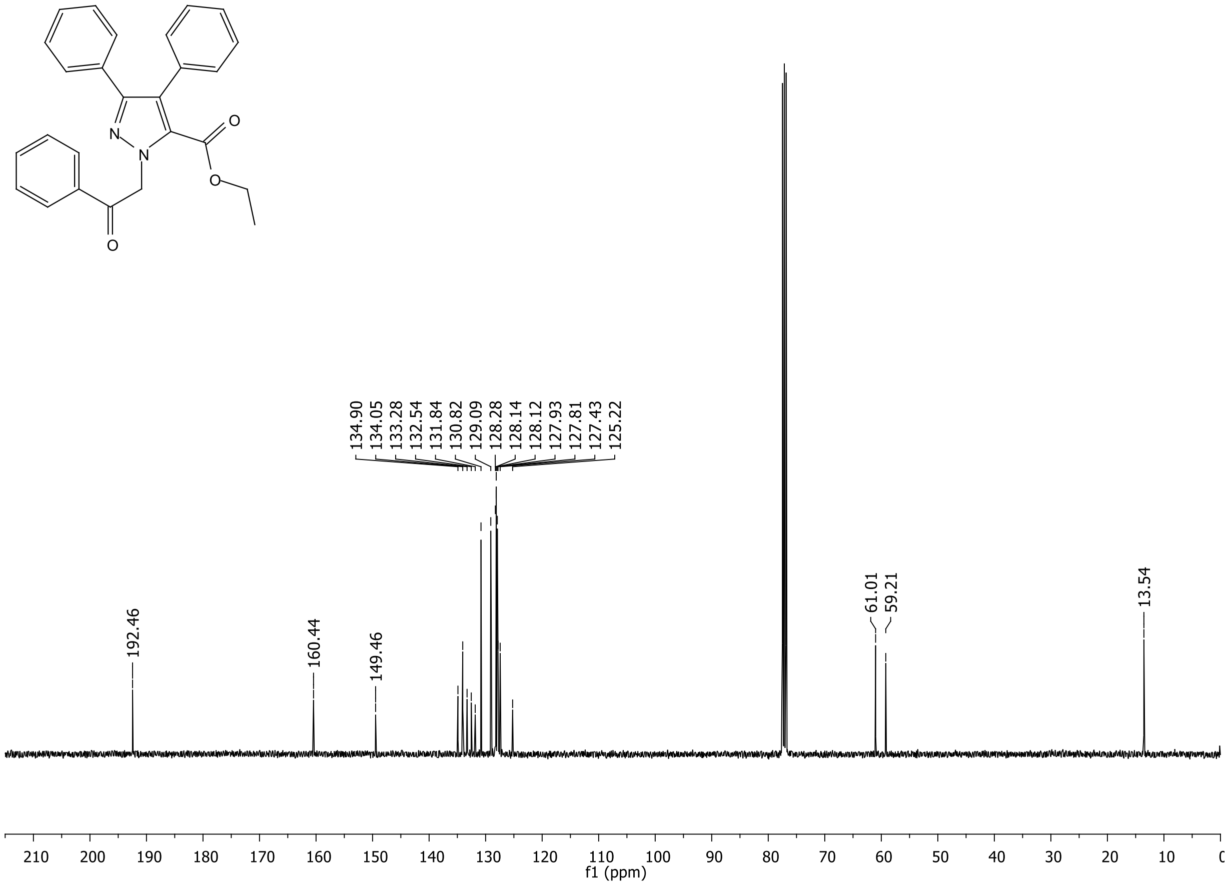

Figure S54. ^{13}C NMR spectrum (101 MHz, $CDCl_3$) of ethyl 1-(2-oxo-2-phenylethyl)-3,4-diphenyl-1*H*-pyrazole-5-carboxylate (**6a**).

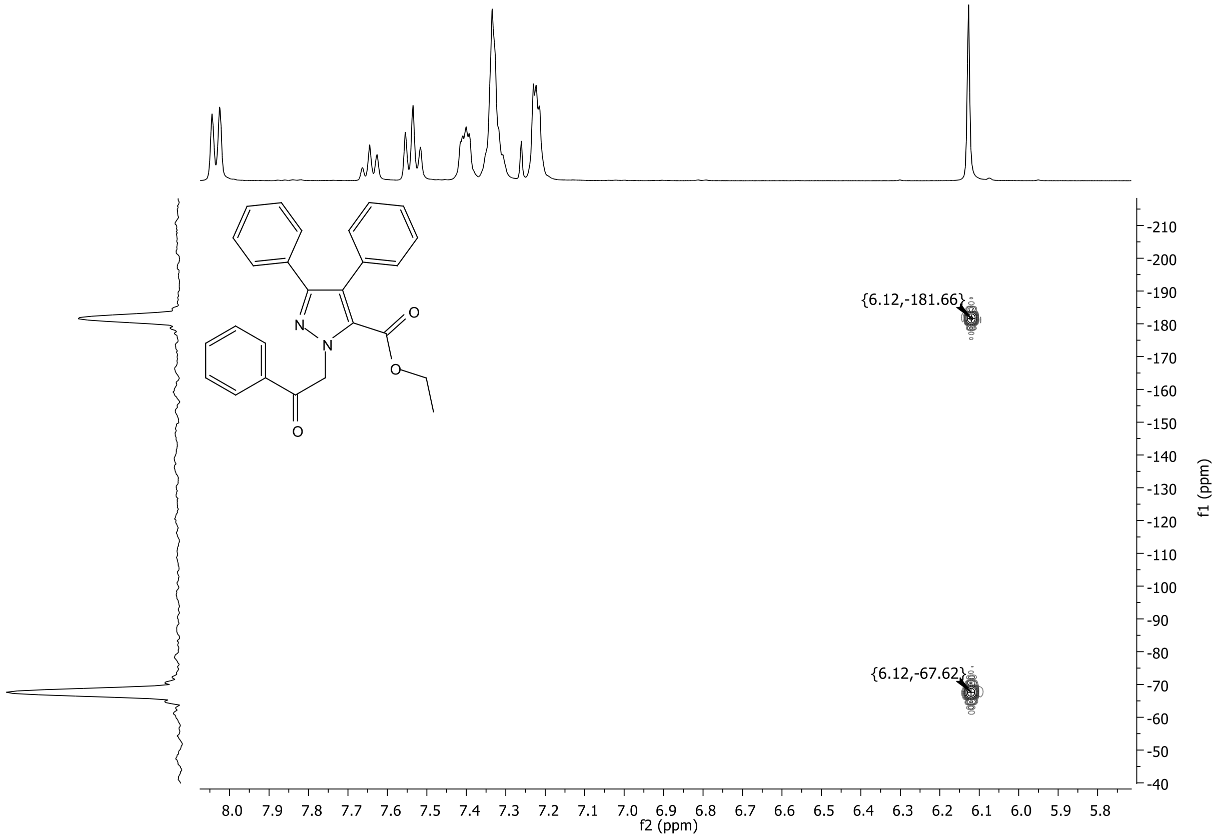

Figure S55. $^{1}H,^{15}N$-HMBC NMR spectrum (40 MHz, $CDCl_3$) of ethyl 1-(2-oxo-2-phenylethyl)-3,4-diphenyl-1*H*-pyrazole-5-carboxylate (**6a**).

Compound Spectrum SmartFormula Report

Analysis Info

Analysis Name	D:\Data\KDD-267-1.d	Acquisition Date	7/5/2022 11:30:17 PM
Method	DirectInfusion_TuneLow_pos.m	Operator	hplc
Sample Name	KDD-267-1	Instrument	micrOTOF-Q III 8228888.20448
Comment	AB		

Acquisition Parameter

Source Type	ESI	Ion Polarity	Positive	Set Nebulizer	0.4 Bar
Focus	Not active	Set Capillary	4500 V	Set Dry Heater	180 °C
Scan Begin	50 m/z	Set End Plate Offset	-500 V	Set Dry Gas	4.0 l/min
Scan End	1000 m/z	Set Collision Cell RF	140.0 Vpp	Set Divert Valve	Waste

#	RT [min]	Area	Int. Type	I	S/N	Chromatogram	Max. m/z	FWHM [min]
n.a.	3.5	n.a.	Single spectrum	n.a.	n.a.	n.a.	433.1523	n.a.

+MS, 3.5min #208

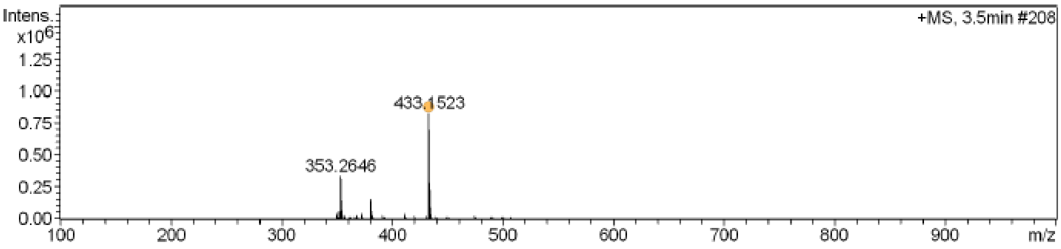

Meas. m/z	#	Ion Formula	m/z	err [ppm]	mSigma	# Sigma	Score	rdb	e⁻ Conf	N-Rule
433.1523	1	C26H22N2NaO3	433.1523	-0.0	21.6	1	100.00	16.5	even	ok

Figure S56. HRMS (ESI-TOF) spectrum of ethyl 1-(2-oxo-2-phenylethyl)-3,4-diphenyl-1*H*-pyrazole-5-carboxylate (**6a**).

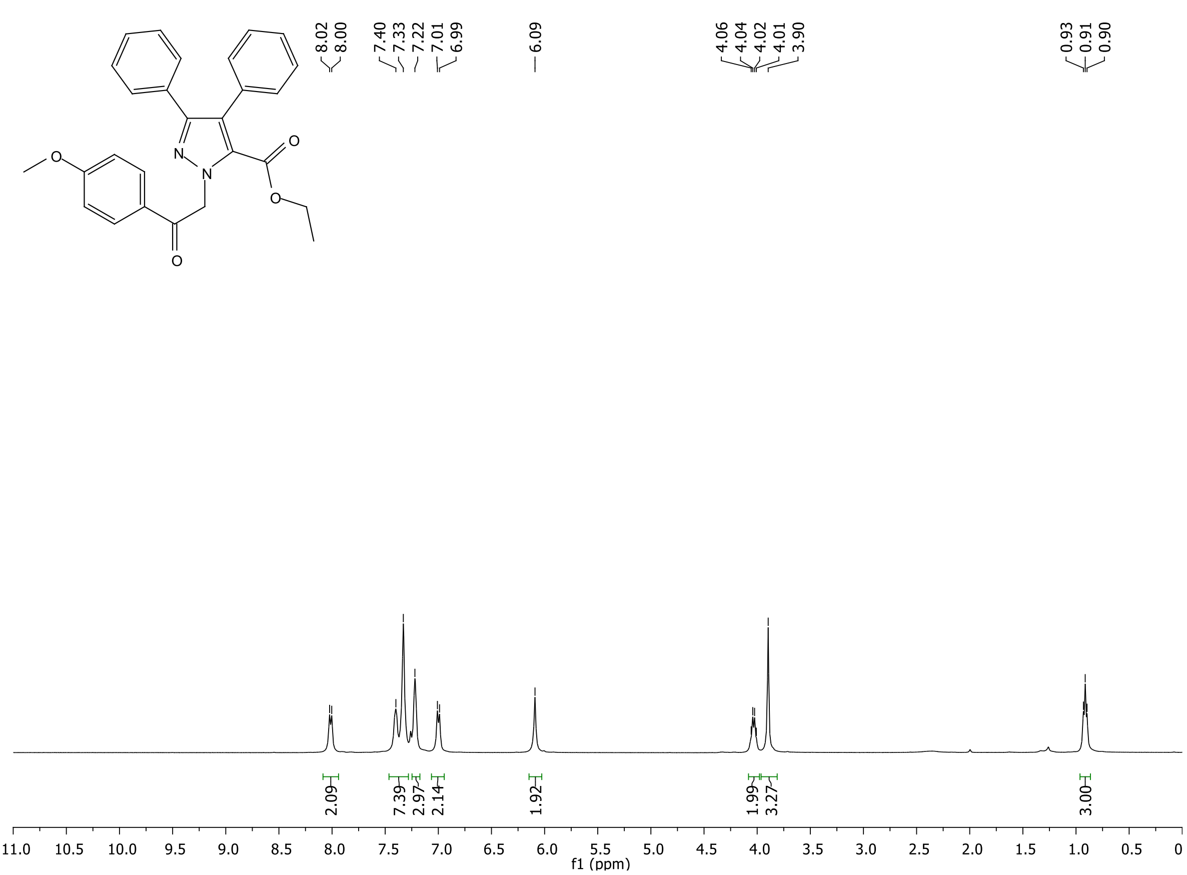

Figure S57. ^{1}H NMR spectrum (400 MHz, $CDCl_3$) of ethyl 1-[2-(4-methoxyphenyl)-2-oxoethyl]-3,4-diphenyl-1*H*-pyrazole-5-carboxylate (**6b**).

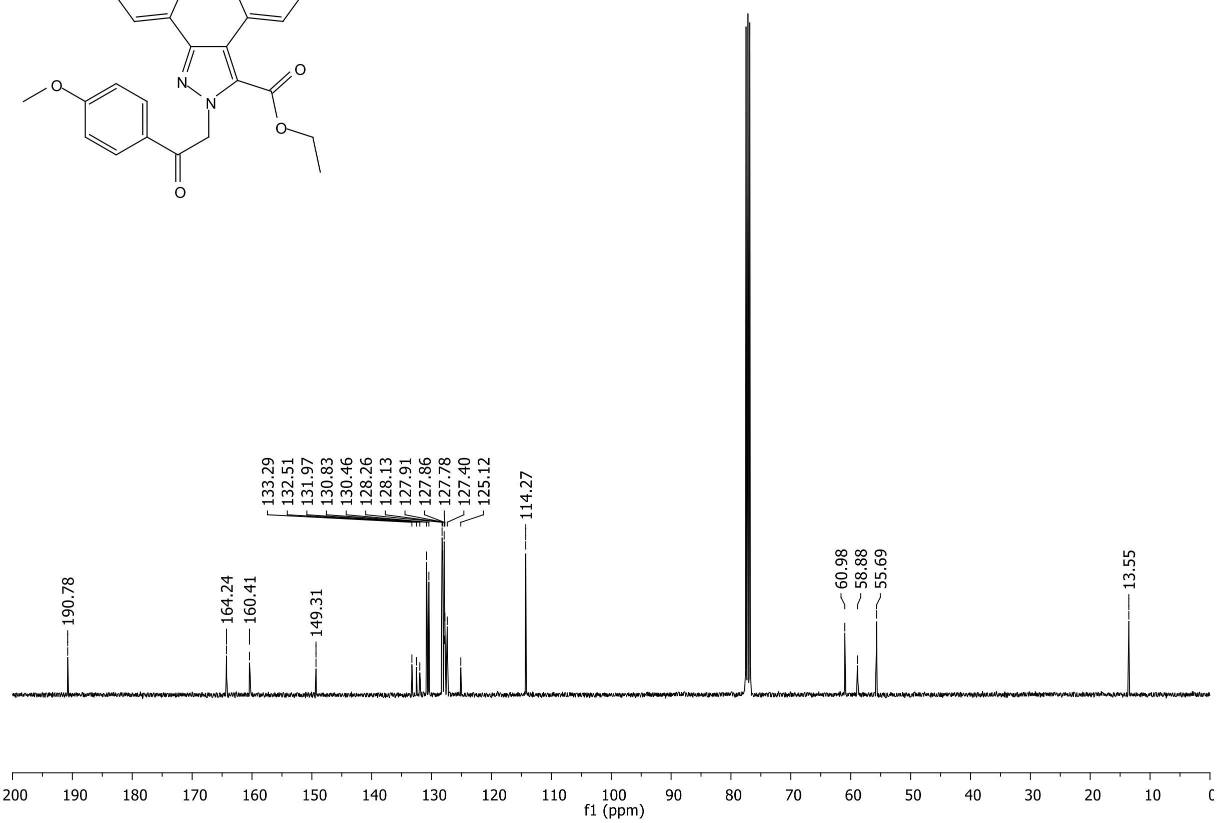

Figure S58. ^{13}C NMR spectrum (101 MHz, $CDCl_3$) of ethyl 1-[2-(4-methoxyphenyl)-2-oxoethyl]-3,4-diphenyl-1*H*-pyrazole-5-carboxylate (**6b**).

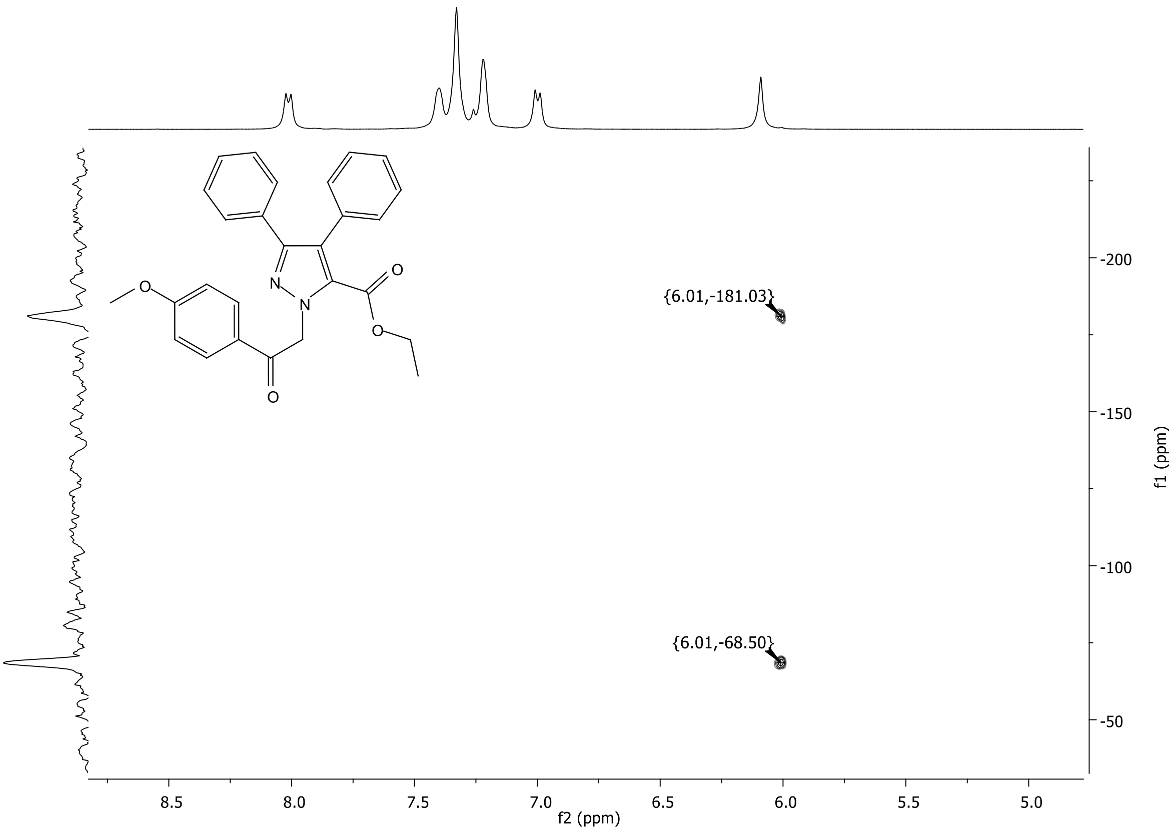

Figure S59. $^{1}H,^{15}N$-HMBC NMR spectrum (40 MHz, $CDCl_3$) of ethyl 1-[2-(4-methoxyphenyl)-2-oxoethyl]-3,4-diphenyl-1*H*-pyrazole-5-carboxylate (**6b**).

Compound Spectrum SmartFormula Report

Analysis Info

Analysis Name	D:\Data\KDD-306.d	Acquisition Date	7/29/2022 5:37:03 PM
Method	DirectInfusion_TuneLow_pos.m	Operator	hplc
Sample Name	KDD-306	Instrument	micrOTOF-Q III 8228888.20448
Comment	AB		

Acquisition Parameter

Source Type	ESI	Ion Polarity	Positive	Set Nebulizer	0.4 Bar
Focus	Not active	Set Capillary	4500 V	Set Dry Heater	180 °C
Scan Begin	50 m/z	Set End Plate Offset	-500 V	Set Dry Gas	4.0 l/min
Scan End	1000 m/z	Set Collision Cell RF	140.0 Vpp	Set Divert Valve	Waste

#	RT [min]	Area	Int. Type	I	S/N	Chromatogram	Max. m/z	FWHM [min]
n.a.	5.3	n.a.	Single spectrum	n.a.	n.a.	n.a.	463.1631	n.a.

+MS, 5.3min #318

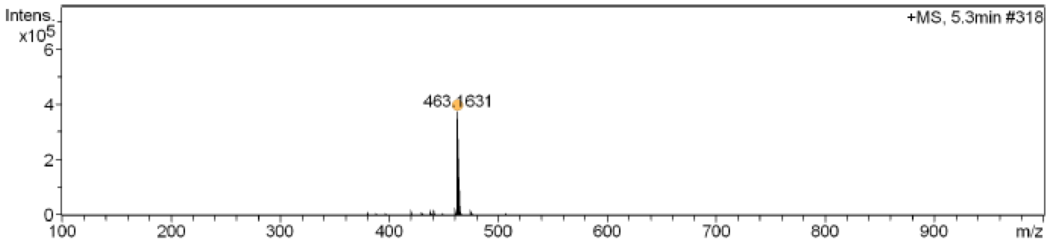

Meas. m/z	#	Ion Formula	m/z	err [ppm]	mSigma	# Sigma	Score	rdb	e⁻ Conf	N-Rule
463.1631	1	C27H24N2NaO4	463.1628	-0.6	4.0	1	100.00	16.5	even	ok

Figure S60. HRMS (ESI-TOF) spectrum of ethyl 1-[2-(4-methoxyphenyl)-2-oxoethyl]-3,4-diphenyl-1*H*-pyrazole-5-carboxylate (**6b**).

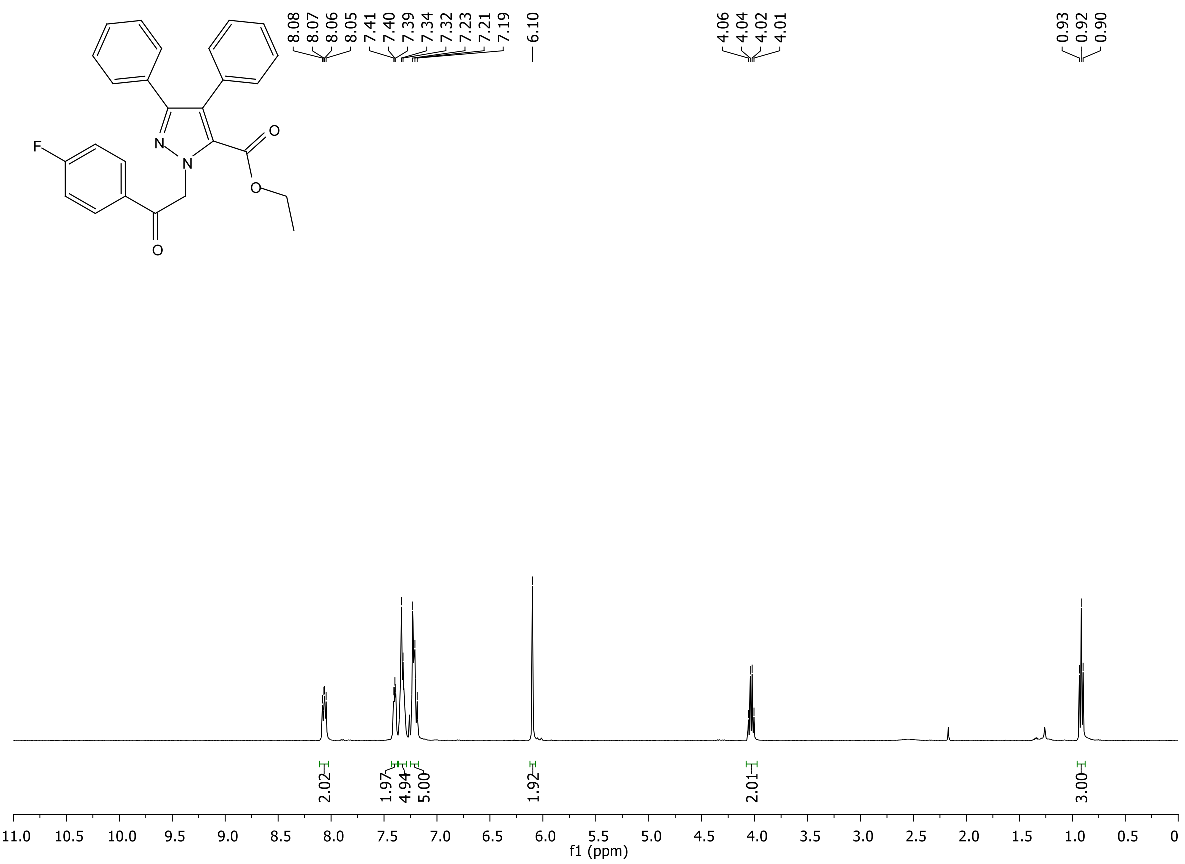

Figure S61. ^{1}H NMR spectrum (400 MHz, $CDCl_3$) of ethyl 1-[2-(4-fluorophenyl)-2-oxoethyl]-3,4-diphenyl-1*H*-pyrazole-5-carboxylate (**6c**).

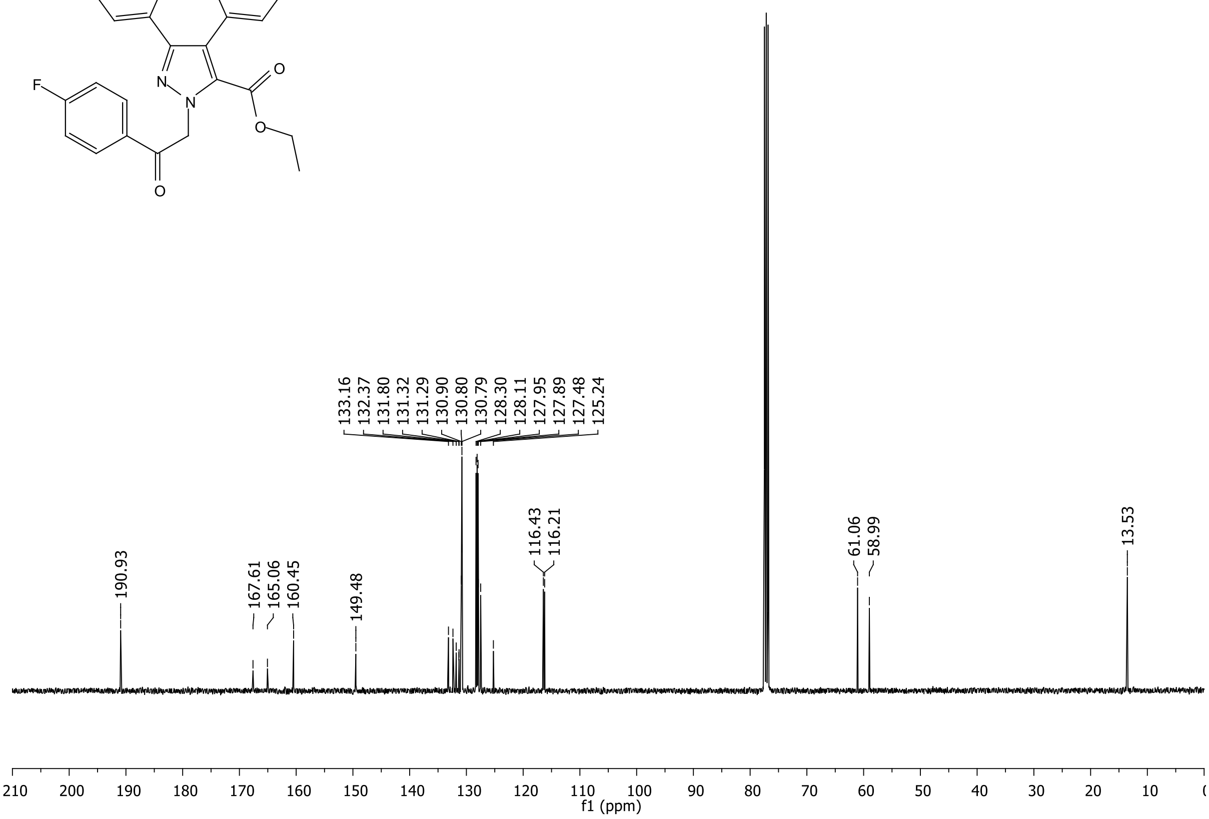

Figure S62. ^{13}C NMR spectrum (101 MHz, $CDCl_3$) of ethyl 1-[2-(4-fluorophenyl)-2-oxoethyl]-3,4-diphenyl-1*H*-pyrazole-5-carboxylate (**6c**).

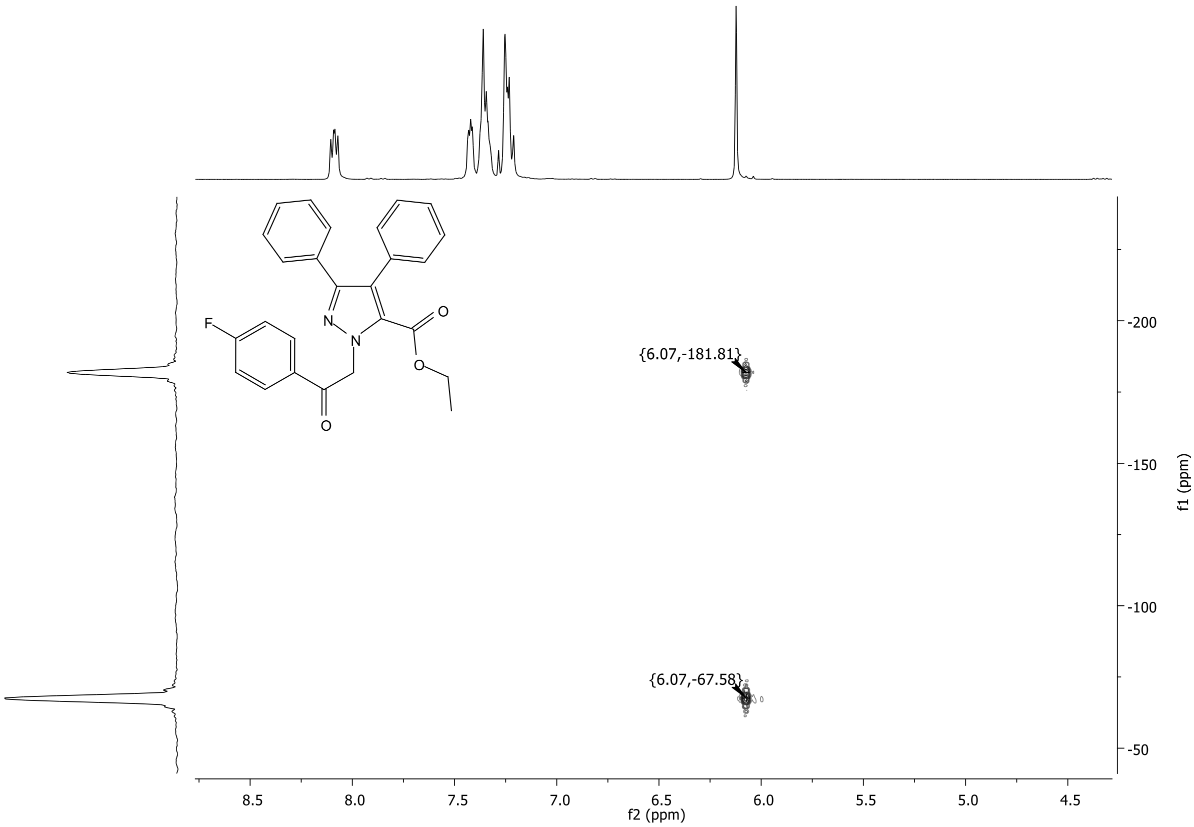

Figure S63. $^{1}H,^{15}N$-HMBC NMR spectrum (40 MHz, $CDCl_3$) of ethyl 1-[2-(4-fluorophenyl)-2-oxoethyl]-3,4-diphenyl-1*H*-pyrazole-5-carboxylate (**6c**).

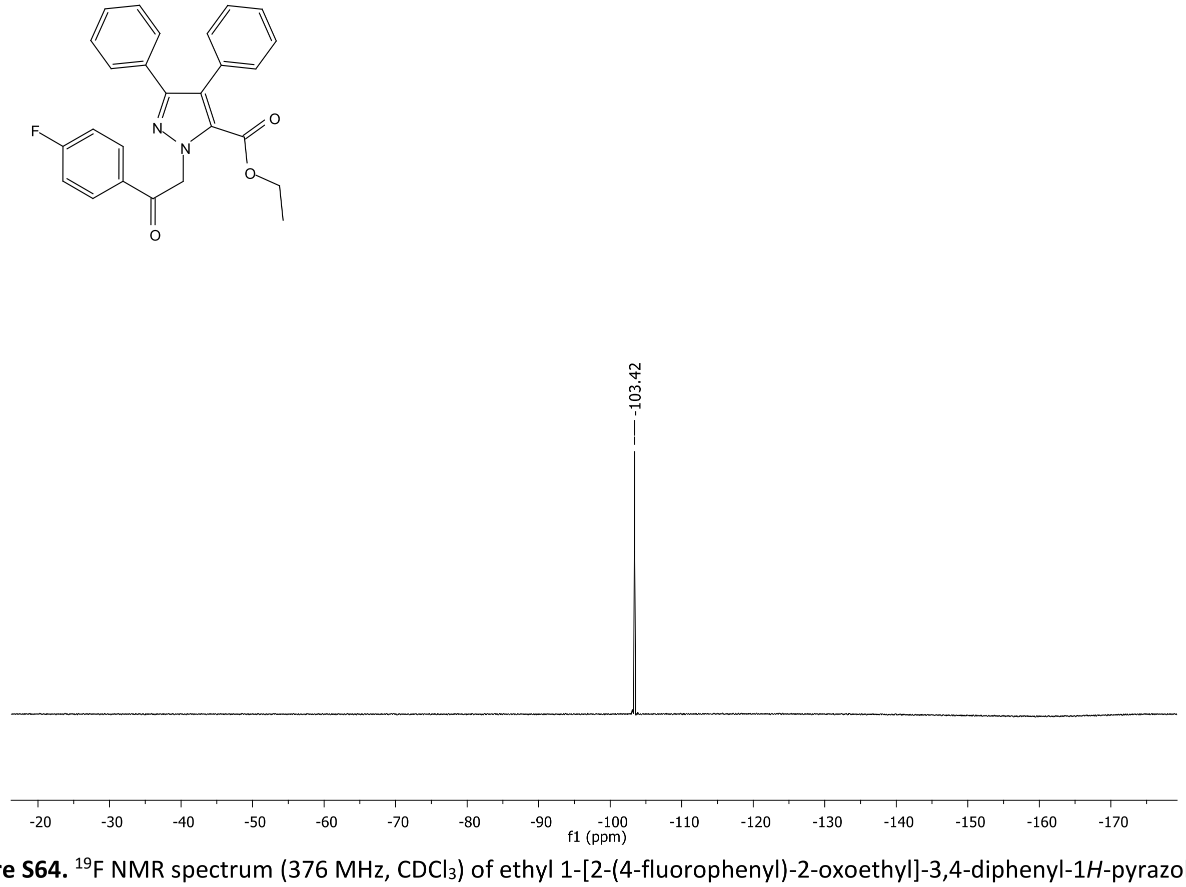

Figure S64. ^{19}F NMR spectrum (376 MHz, $CDCl_3$) of ethyl 1-[2-(4-fluorophenyl)-2-oxoethyl]-3,4-diphenyl-1*H*-pyrazole-5-carboxylate (**6c**).

Compound Spectrum SmartFormula Report

Analysis Info			
Analysis Name	D:\Data\KDD-324-1.d	Acquisition Date	7/5/2022 5:58:15 PM
Method	DirectInfusion_TuneLow_pos.m	Operator	hplc
Sample Name	KDD-324-1	Instrument	micrOTOF-Q III 8228888.20448
Comment	AB		

Acquisition Parameter

Source Type	ESI	Ion Polarity	Positive	Set Nebulizer	0.4 Bar
Focus	Not active	Set Capillary	4500 V	Set Dry Heater	180 °C
Scan Begin	50 m/z	Set End Plate Offset	-500 V	Set Dry Gas	4.0 l/min
Scan End	1000 m/z	Set Collision Cell RF	140.0 Vpp	Set Divert Valve	Waste

#	RT [min]	Area	Int. Type	I	S/N	Chromatogram	Max. m/z	FWHM [min]
n.a.	5.2	n.a.	Single spectrum	n.a.	n.a.	n.a.	451.1431	n.a.

+MS, 5.2min #309

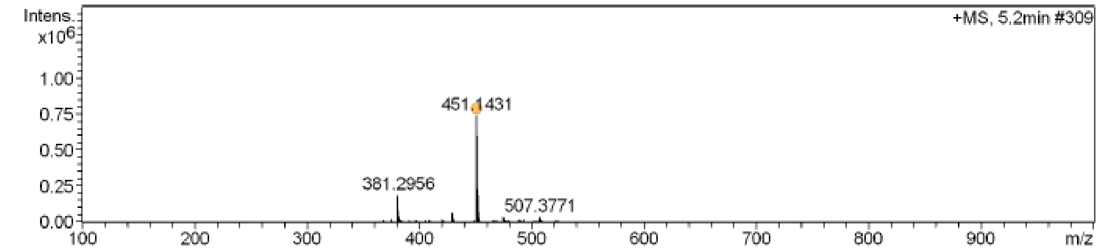

Meas. m/z	#	Ion Formula	m/z	err [ppm]	mSigma	# Sigma	Score	rdb	e⁻ Conf	N-Rule
451.1431	1	C26H21FN2NaO3	451.1428	0.7	10.8	2	100.00	16.5	even	ok

Figure S65. HRMS (ESI-TOF) spectrum of ethyl 1-[2-(4-fluorophenyl)-2-oxoethyl]-3,4-diphenyl-1*H*-pyrazole-5-carboxylate (**6c**).

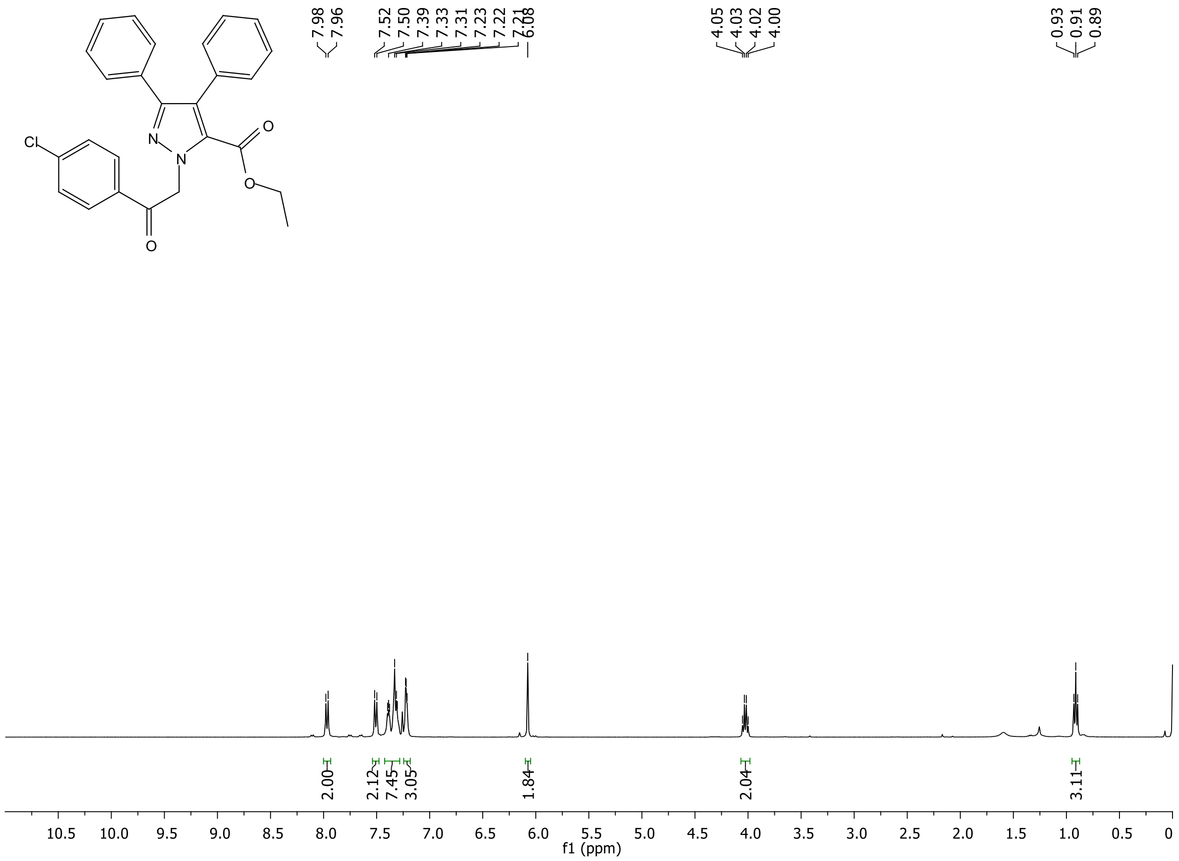

Figure S66. ^{1}H NMR spectrum (400 MHz, $CDCl_3$) of ethyl 1-[2-(4-chlorophenyl)-2-oxoethyl]-3,4-diphenyl-1*H*-pyrazole-5-carboxylate (**6d**).

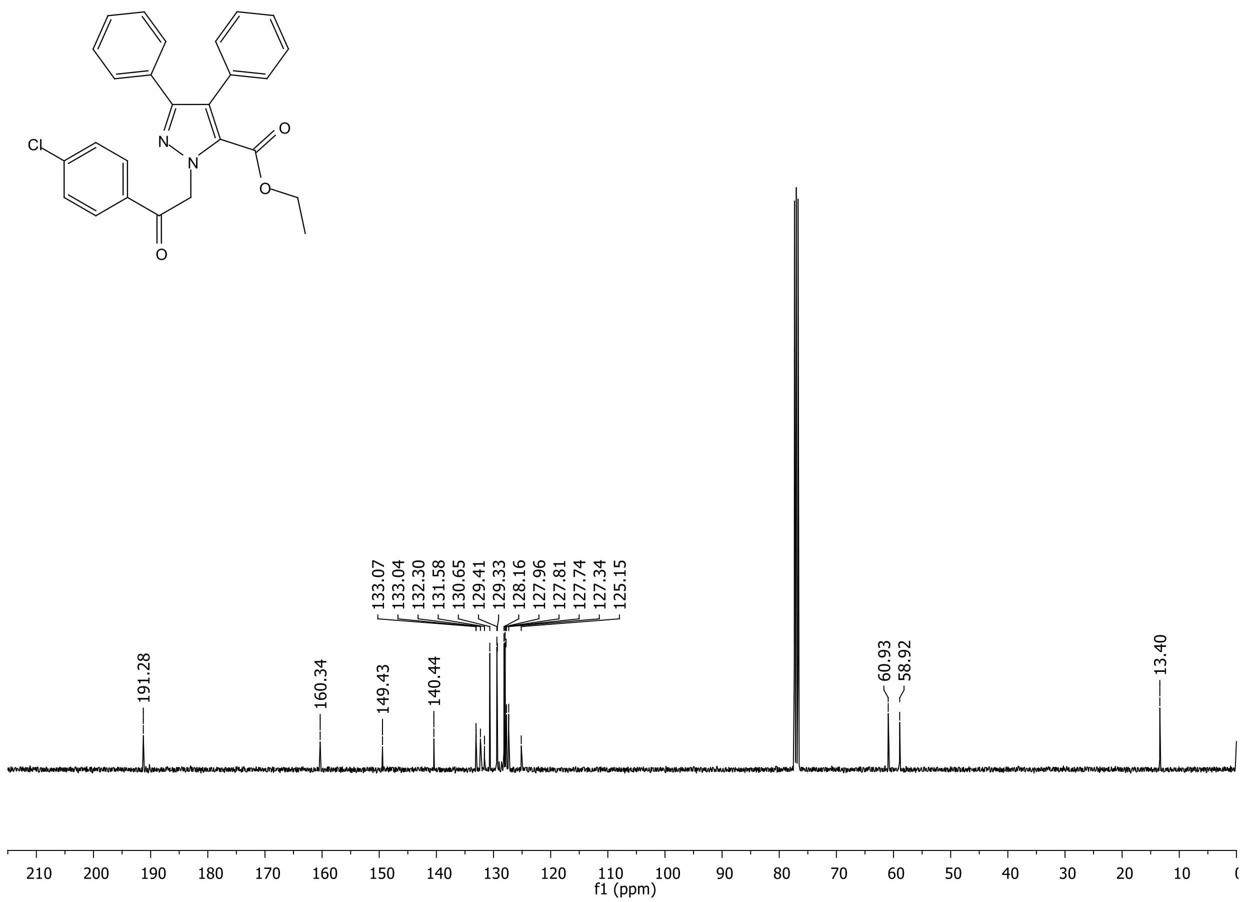

Figure S67. ^{13}C NMR spectrum (101 MHz, $CDCl_3$) of ethyl 1-[2-(4-chlorophenyl)-2-oxoethyl]-3,4-diphenyl-1*H*-pyrazole-5-carboxylate (**6d**).

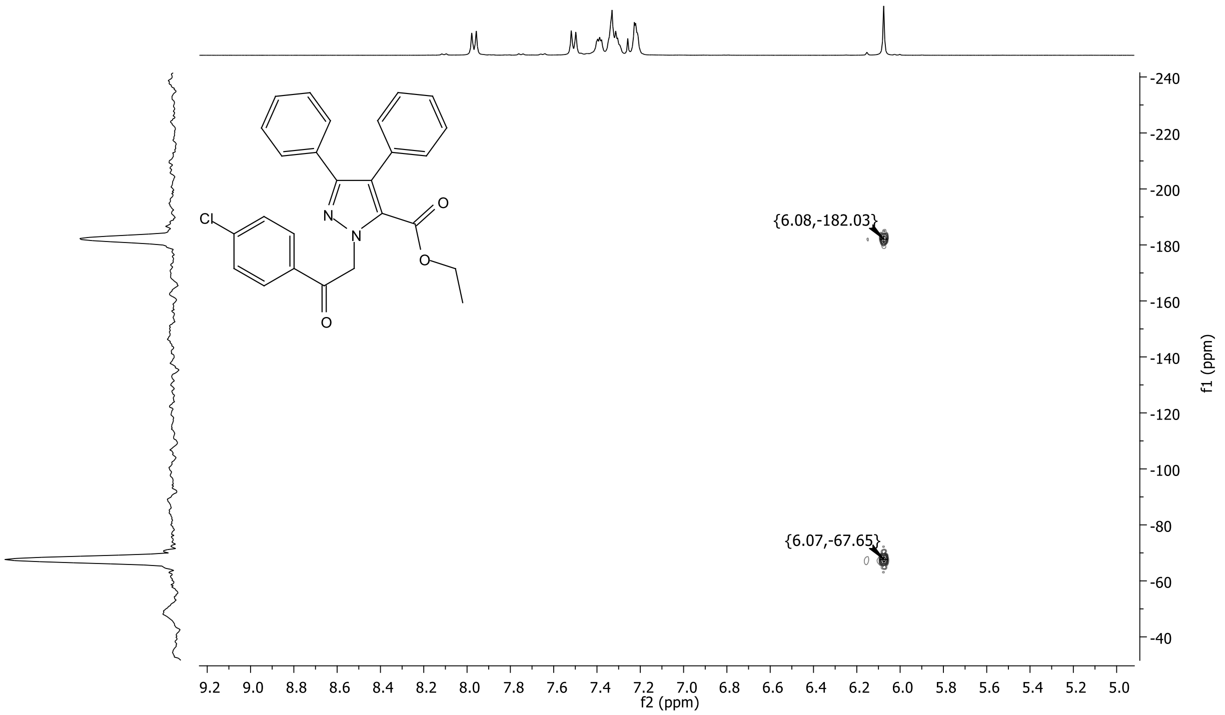

Figure S68. $^{1}H,^{15}N$-HMBC NMR spectrum (40 MHz, $CDCl_3$) of ethyl 1-[2-(4-chlorophenyl)-2-oxoethyl]-3,4-diphenyl-1*H*-pyrazole-5-carboxylate (**6d**).

Compound Spectrum SmartFormula Report

Analysis Info

Analysis Name	D:\Data\KDD-305.d	Acquisition Date	7/29/2022 5:22:45 PM
Method	DirectInfusion_TuneLow_pos.m	Operator	hplc
Sample Name	KDD-305	Instrument	micrOTOF-Q III 8228888.20448
Comment	AB		

Acquisition Parameter

Source Type	ESI	Ion Polarity	Positive	Set Nebulizer	0.4 Bar
Focus	Not active	Set Capillary	4500 V	Set Dry Heater	180 °C
Scan Begin	50 m/z	Set End Plate Offset	-500 V	Set Dry Gas	4.0 l/min
Scan End	1000 m/z	Set Collision Cell RF	140.0 Vpp	Set Divert Valve	Waste

#	RT [min]	Area	Int. Type	I	S/N	Chromatogram	Max. m/z	FWHM [min]
n.a.	5.2	n.a.	Single spectrum	n.a.	n.a.	n.a.	467.1134	n.a.

+MS, 5.2min #309

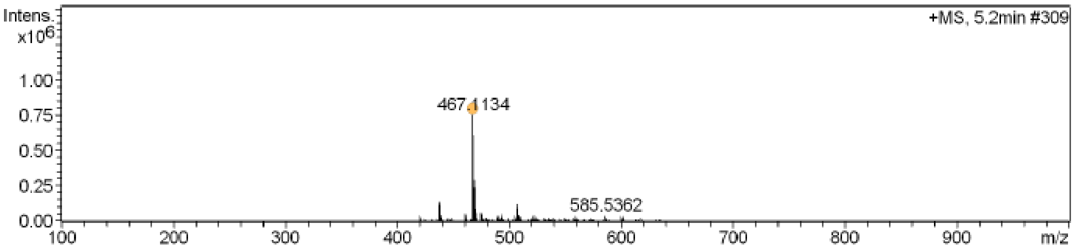

Meas. m/z	#	Ion Formula	m/z	err [ppm]	mSigma	# Sigma	Score	rdb	e⁻ Conf	N-Rule
467.1134	1	C26H21ClN2NaO3	467.1133	0.3	18.3	1	100.00	16.5	even	ok

Figure S69. HRMS (ESI-TOF) spectrum of ethyl 1-[2-(4-chlorophenyl)-2-oxoethyl]-3,4-diphenyl-1*H*-pyrazole-5-carboxylate (**6d**).

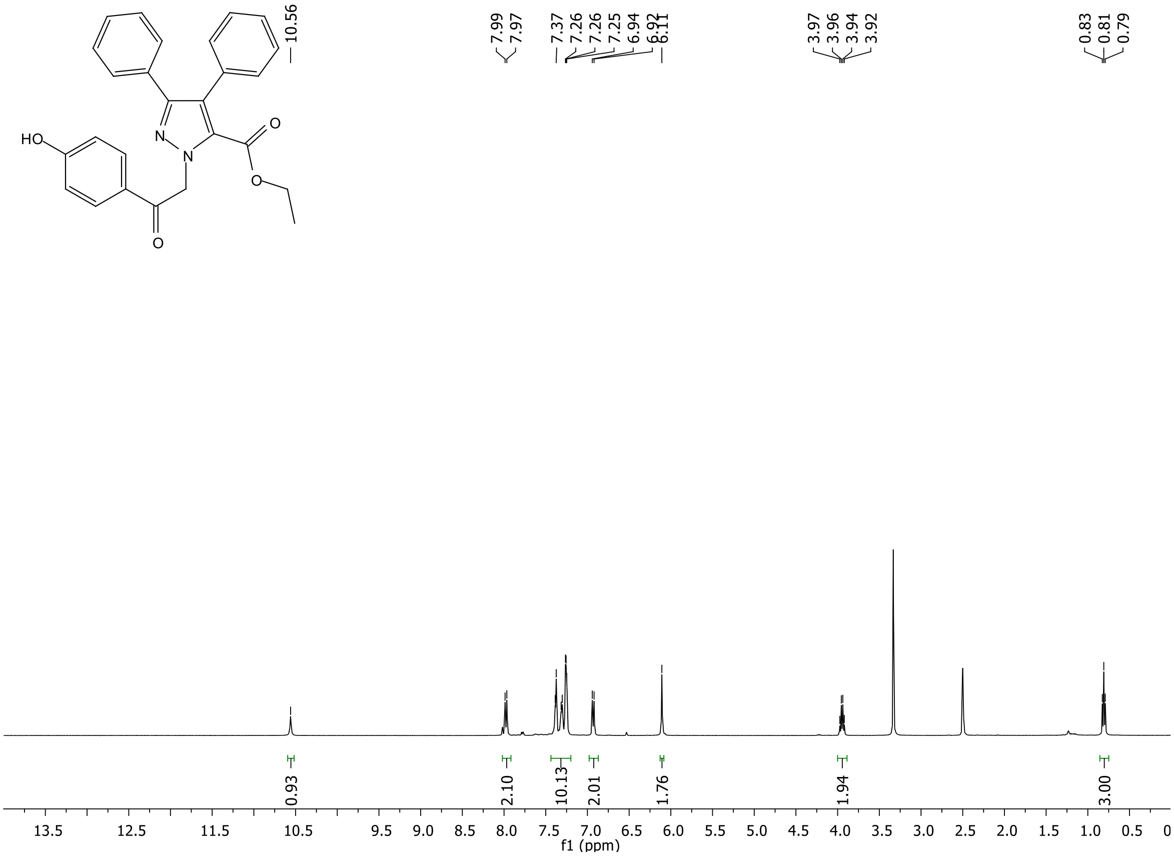

Figure S70. ^{1}H NMR spectrum (400 MHz, DMSO-d_6) of ethyl 1-[2-(4-hydroxyphenyl]-2-oxoethyl)-3,4-diphenyl-1*H*-pyrazole-5-carboxylate (**6e**).

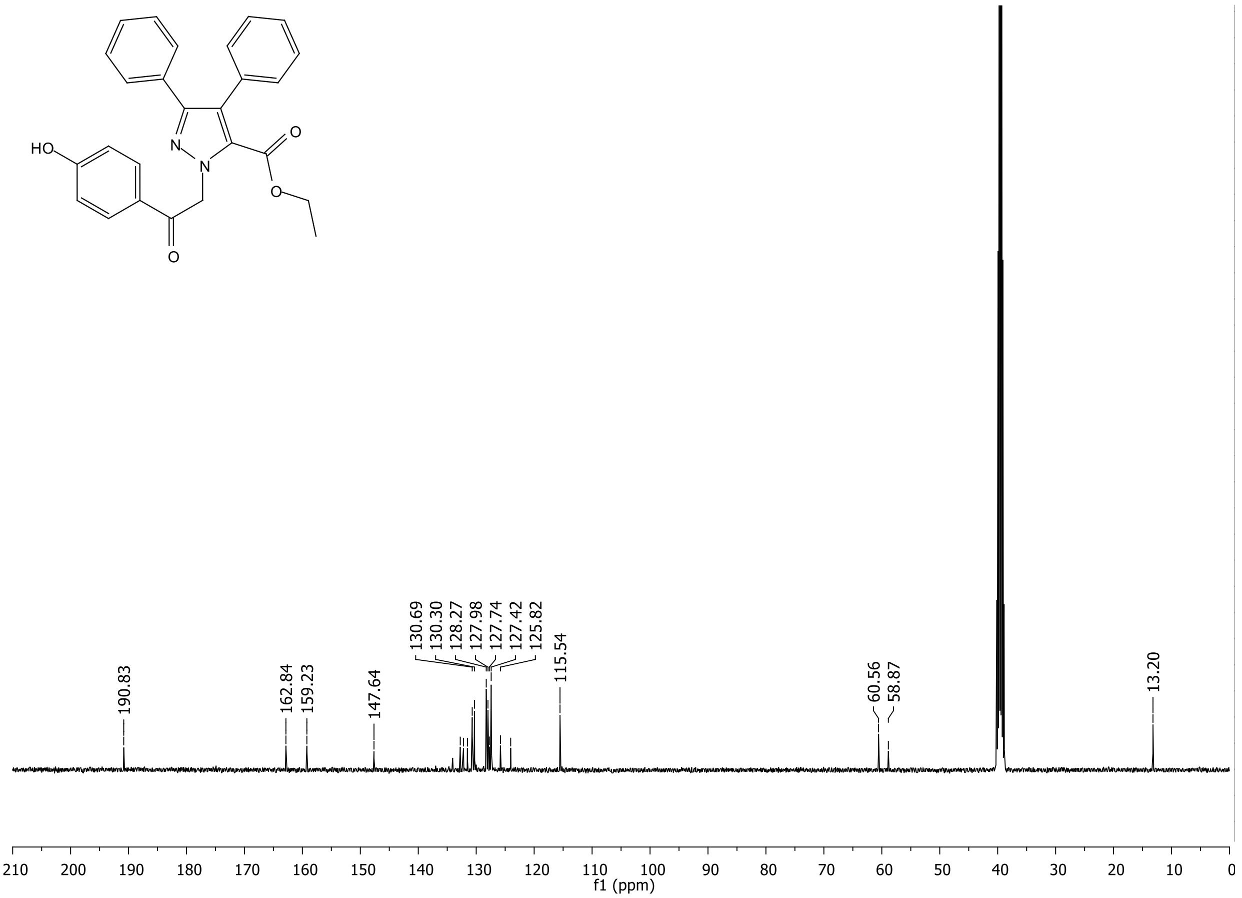

Figure S71. ^{13}C NMR spectrum (101 MHz, DMSO-d_6) of ethyl 1-[2-(4-hydroxyphenyl]-2-oxoethyl)-3,4-diphenyl-1*H*-pyrazole-5-carboxylate (**6e**).

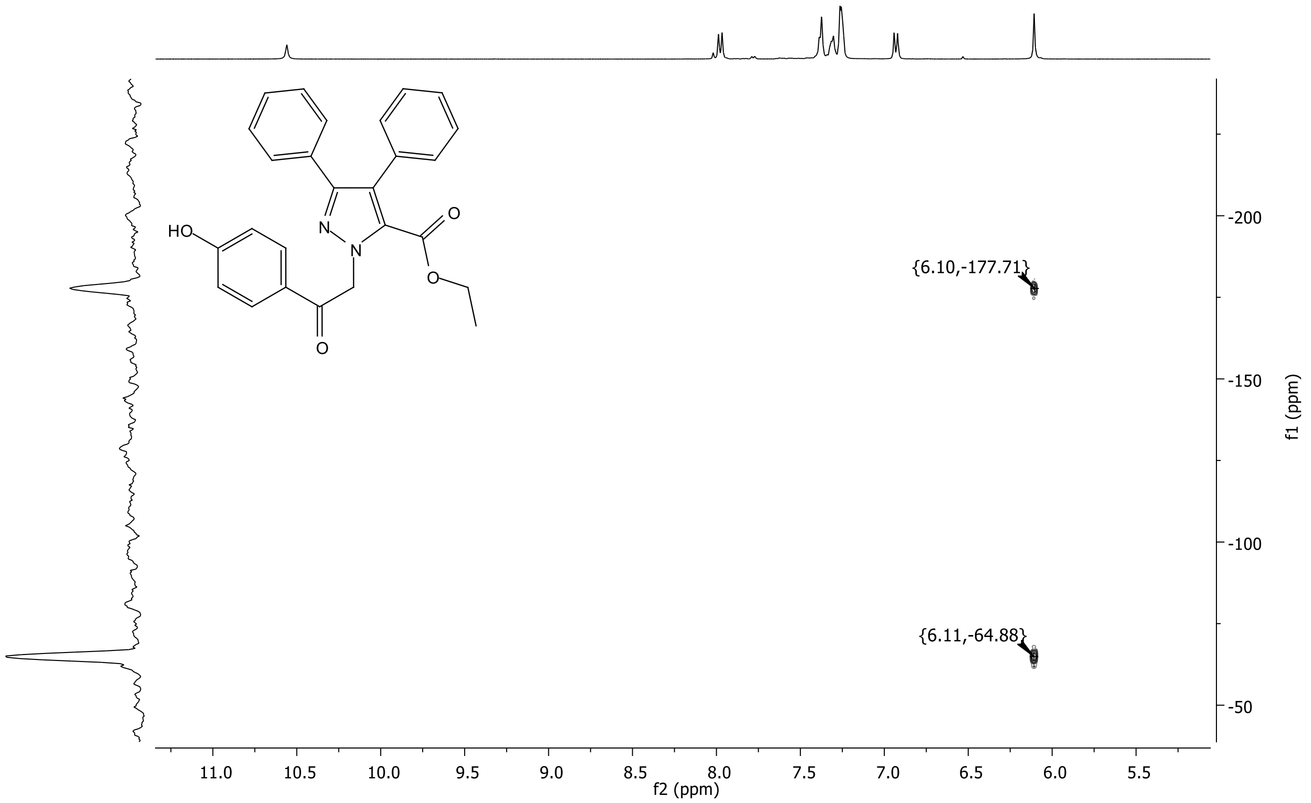

Figure S72. $^{1}H,^{15}N$-HMBC NMR spectrum (40 MHz, DMSO-d_6) of ethyl 1-[2-(4-hydroxyphenyl]-2-oxoethyl)-3,4-diphenyl-1*H*-pyrazole-5-carboxylate (**6e**).

Compound Spectrum SmartFormula Report

Analysis Info

Analysis Name	D:\Data\KDD-326.d	Acquisition Date	7/29/2022 6:09:17 PM
Method	DirectInfusion_TuneLow_pos.m		
Sample Name	KDD-326	Operator	hplc
Comment	AB	Instrument	micrOTOF-Q III 8228888.20448

Acquisition Parameter

Source Type	ESI	Ion Polarity	Positive	Set Nebulizer	0.4 Bar
Focus	Not active	Set Capillary	4500 V	Set Dry Heater	180 °C
Scan Begin	50 m/z	Set End Plate Offset	-500 V	Set Dry Gas	4.0 l/min
Scan End	1000 m/z	Set Collision Cell RF	140.0 Vpp	Set Divert Valve	Waste

#	RT [min]	Area	Int. Type	I	S/N	Chromatogram	Max. m/z	FWHM [min]
n.a.	4.8	n.a.	Single spectrum	n.a.	n.a.	n.a.	449.1469	n.a.

+MS, 4.8min #288

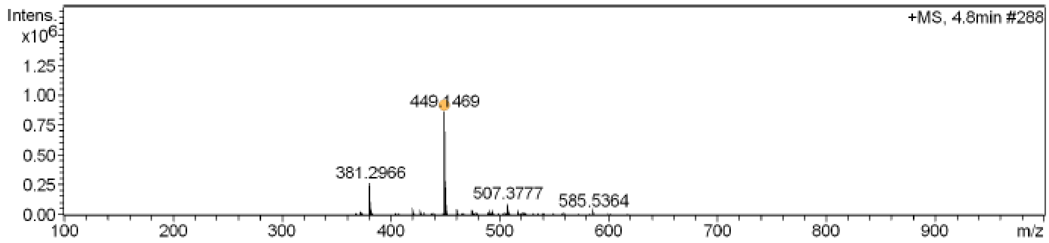

Meas. m/z	#	Ion Formula	m/z	err [ppm]	mSigma	# Sigma	Score	rdb	e⁻ Conf	N-Rule
449.1469	1	C26H22N2NaO4	449.1472	0.6	17.1	2	100.00	16.5	even	ok

Figure S73. HRMS (ESI-TOF) spectrum of ethyl 1-[2-(4-hydroxyphenyl]-2-oxoethyl)-3,4-diphenyl-1*H*-pyrazole-5-carboxylate (**6e**).

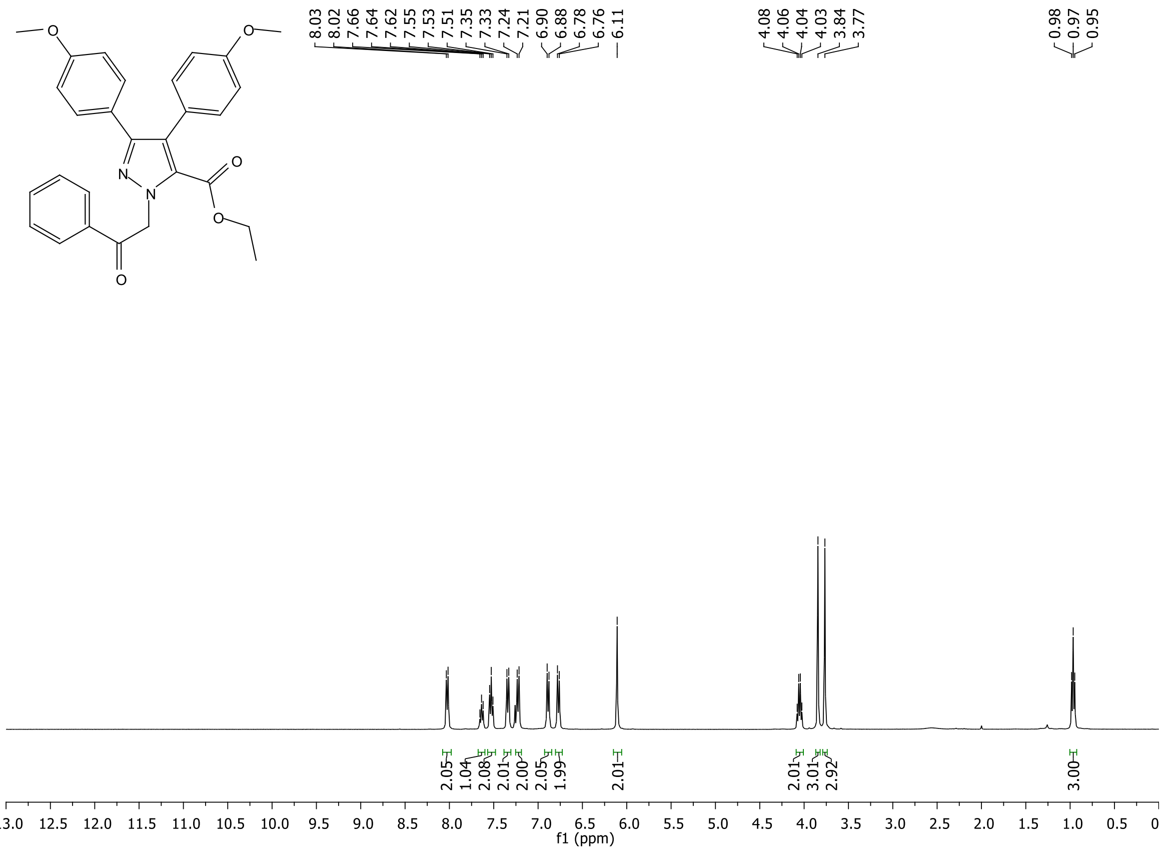

Figure S74. ^{1}H NMR spectrum (400 MHz, $CDCl_3$) of ethyl 3,4-bis(4-methoxyphenyl)-1-(2-oxo-2-phenylethyl)-1*H*-pyrazole-5-carboxylate (**6f**).

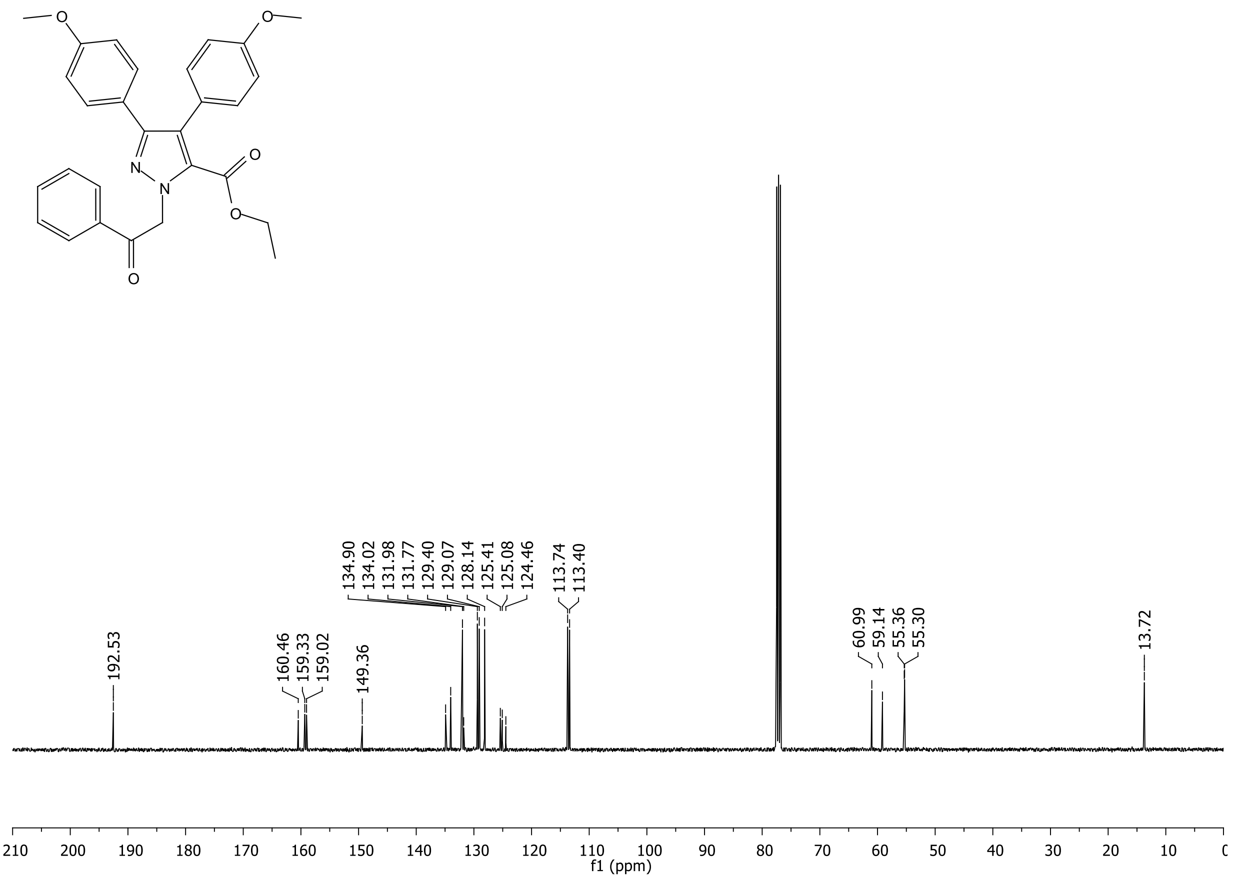

Figure S75. ^{13}C NMR spectrum (101 MHz, $CDCl_3$) of ethyl 3,4-bis(4-methoxyphenyl)-1-(2-oxo-2-phenylethyl)-1*H*-pyrazole-5-carboxylate (**6f**).

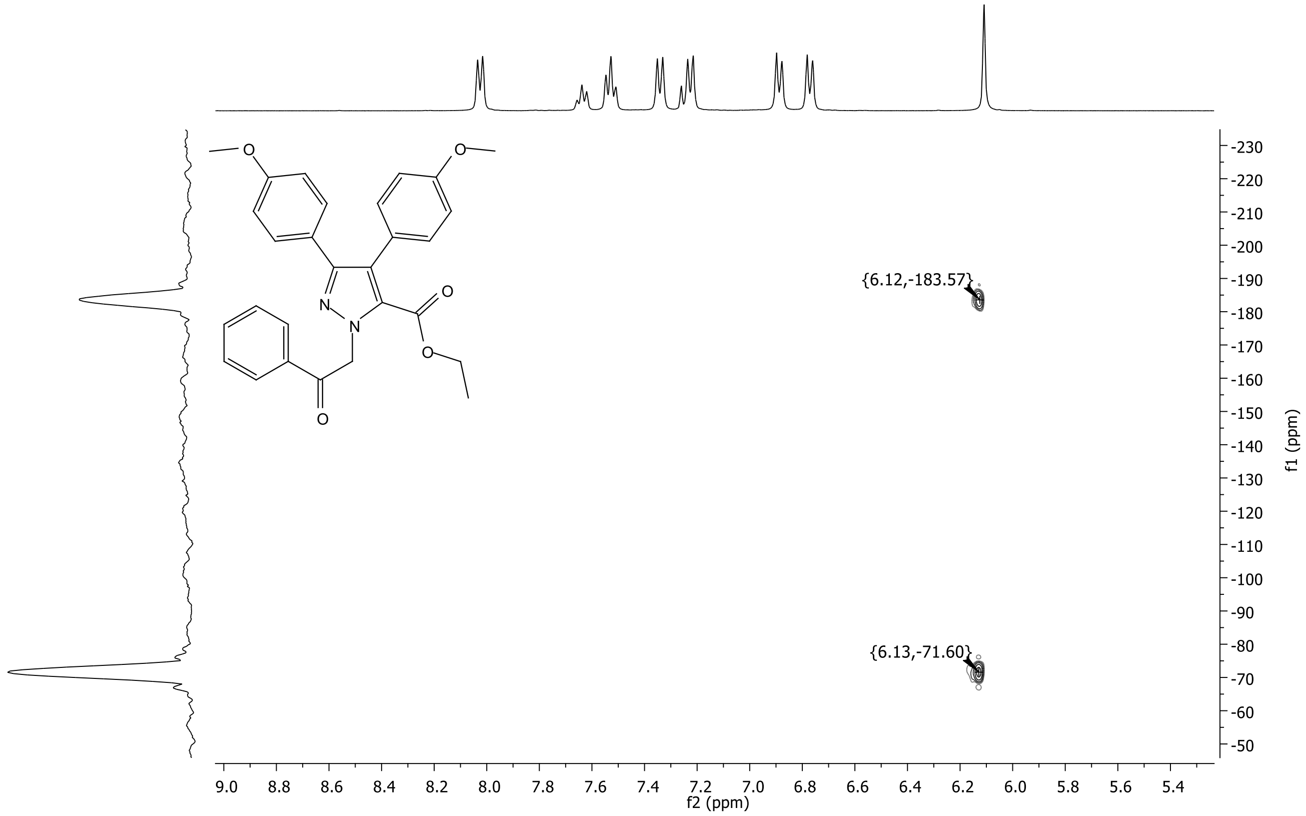

Figure S76. $^{1}H,^{15}N$-HMBC NMR spectrum (40 MHz, $CDCl_3$) of ethyl 3,4-bis(4-methoxyphenyl)-1-(2-oxo-2-phenylethyl)-1*H*-pyrazole-5-carboxylate (**6f**).

Compound Spectrum SmartFormula Report

Analysis Info

Analysis Name	D:\Data\KDD-308-2.d	Acquisition Date	7/5/2022 11:41:57 PM
Method	DirectInfusion_TuneLow_pos.m	Operator	hplc
Sample Name	KDD-308-2	Instrument	micrOTOF-Q III 8228888.20448
Comment	AB		

Acquisition Parameter

Source Type	ESI	Ion Polarity	Positive	Set Nebulizer	0.4 Bar
Focus	Not active	Set Capillary	4500 V	Set Dry Heater	180 °C
Scan Begin	50 m/z	Set End Plate Offset	-500 V	Set Dry Gas	4.0 l/min
Scan End	1000 m/z	Set Collision Cell RF	140.0 Vpp	Set Divert Valve	Waste

#	RT [min]	Area	Int. Type	I	S/N	Chromatogram	Max. m/z	FWHM [min]
n.a.	5.7	n.a.	Single spectrum	n.a.	n.a.	n.a.	493.1732	n.a.

+MS, 5.7min #344

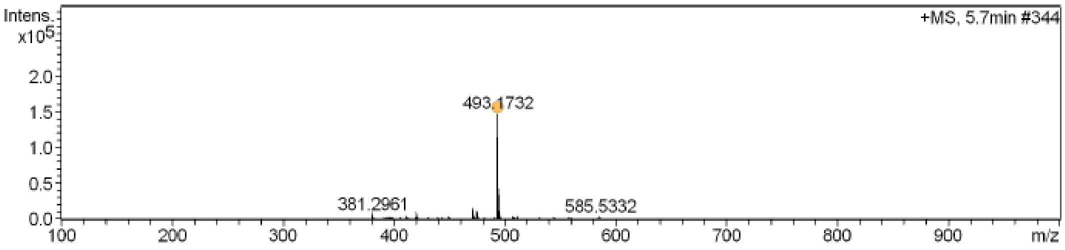

Meas. m/z	#	Ion Formula	m/z	err [ppm]	mSigma	# Sigma	Score	rdb	e⁻ Conf	N-Rule
493.1732	1	C28H26N2NaO5	493.1734	0.3	5.5	1	100.00	16.5	even	ok

Figure S77. HRMS (ESI-TOF) spectrum of ethyl 3,4-bis(4-methoxyphenyl)-1-(2-oxo-2-phenylethyl)-1*H*-pyrazole-5-carboxylate (**6f**).

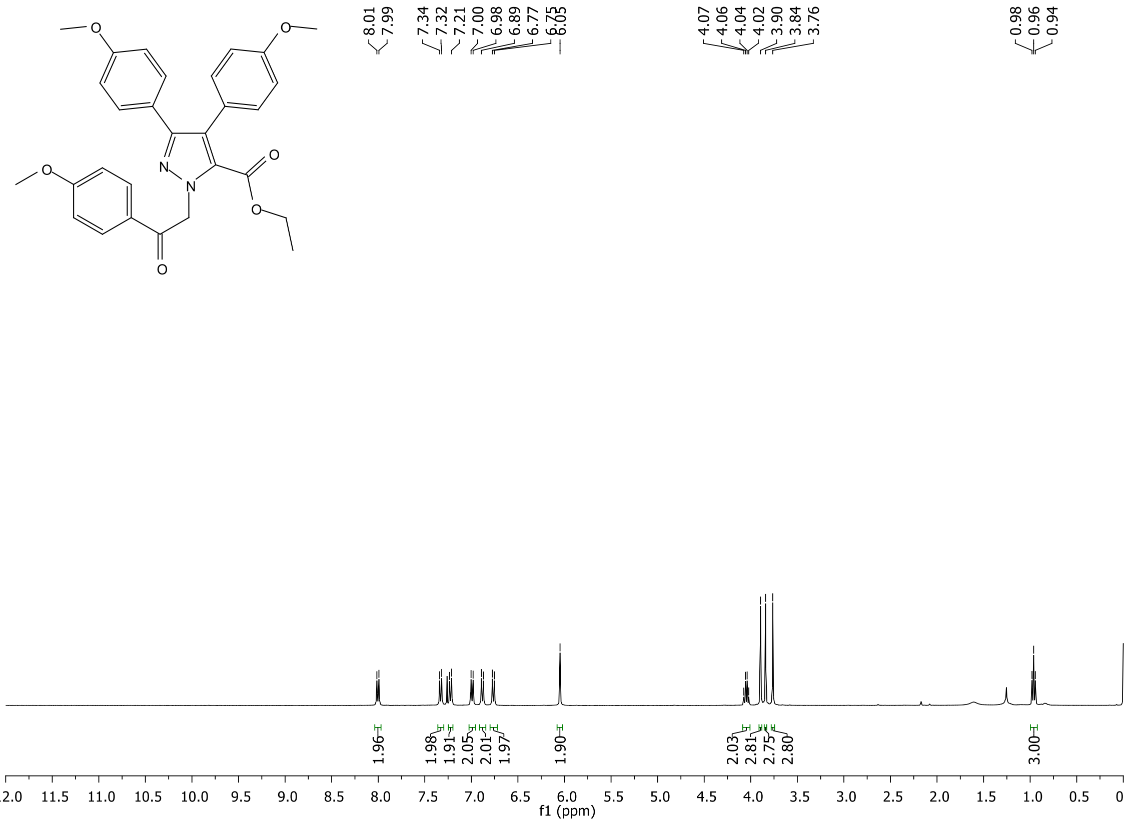

Figure S78. ^{1}H NMR spectrum (400 MHz, $CDCl_3$) of ethyl 3,4-bis(4-methoxyphenyl)-1-[2-(4-methoxyphenyl)-2-oxoethyl]-1*H*-pyrazole-5-carboxylate (**6g**).

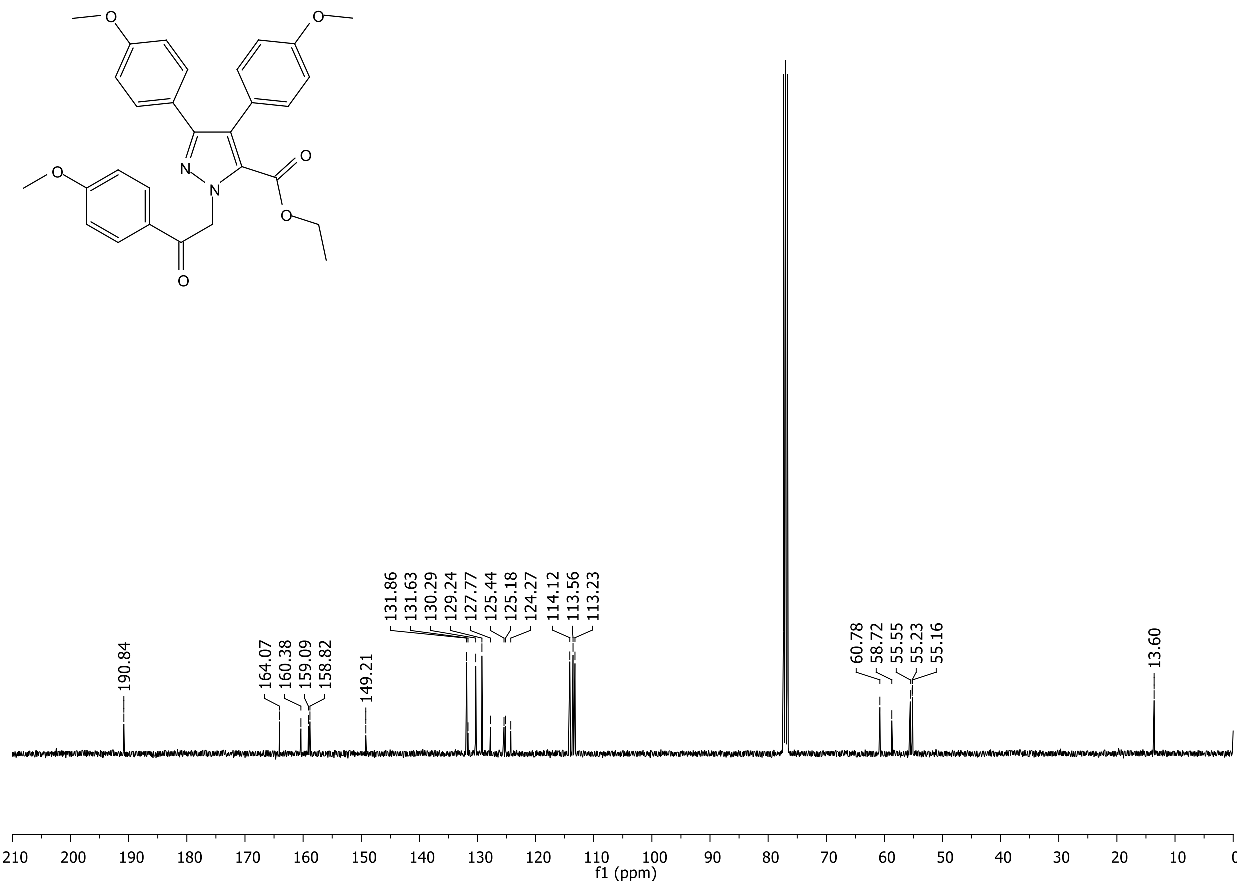

Figure S79. ^{13}C NMR spectrum (101 MHz, $CDCl_3$) of ethyl 3,4-bis(4-methoxyphenyl)-1-[2-(4-methoxyphenyl)-2-oxoethyl]-1*H*-pyrazole-5-carboxylate (**6g**).

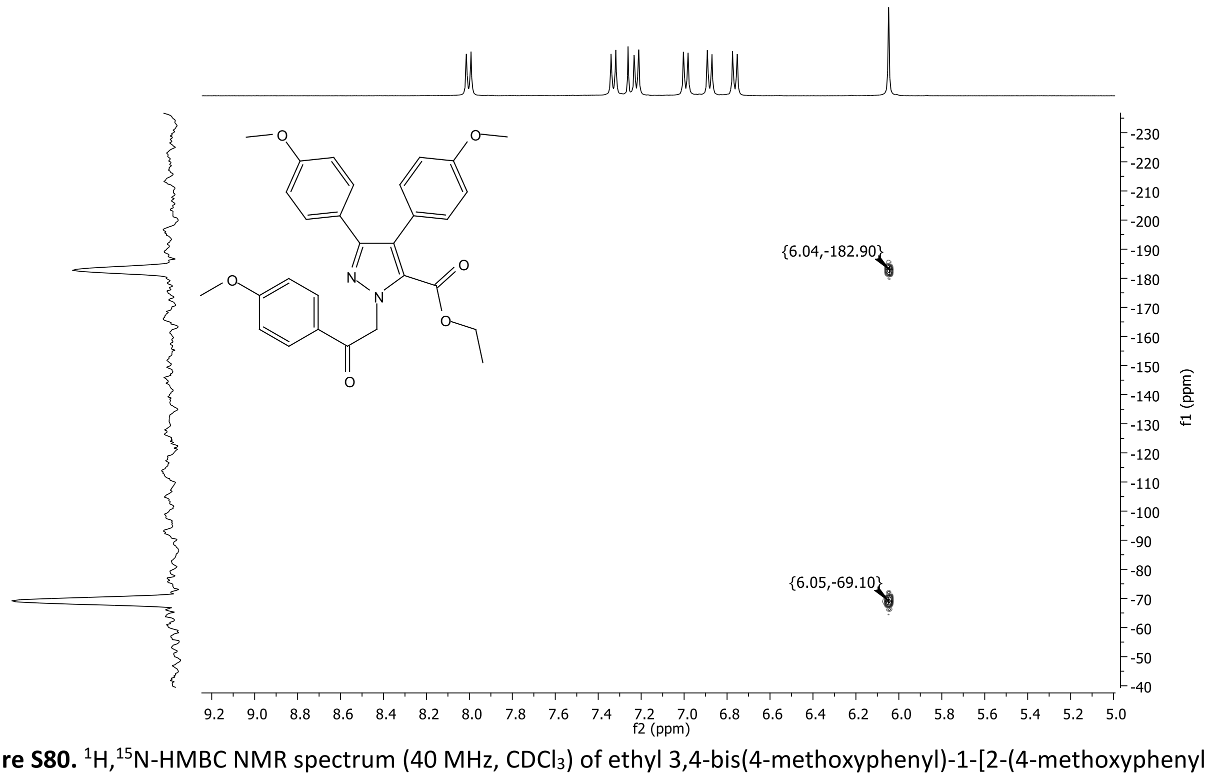

Figure S80. $^{1}H,^{15}N$-HMBC NMR spectrum (40 MHz, $CDCl_3$) of ethyl 3,4-bis(4-methoxyphenyl)-1-[2-(4-methoxyphenyl)-2-oxoethyl]-1*H*-pyrazole-5-carboxylate (**6g**).

Compound Spectrum SmartFormula Report

Analysis Info

Analysis Name	D:\Data\KDD-309-1.d	Acquisition Date	7/5/2022 11:55:00 PM
Method	DirectInfusion_TuneLow_pos.m	Operator	hplc
Sample Name	KDD-309-1	Instrument	micrOTOF-Q III 8228888.20448
Comment	AB		

Acquisition Parameter

Source Type	ESI	Ion Polarity	Positive	Set Nebulizer	0.4 Bar
Focus	Not active	Set Capillary	4500 V	Set Dry Heater	180 °C
Scan Begin	50 m/z	Set End Plate Offset	-500 V	Set Dry Gas	4.0 l/min
Scan End	1000 m/z	Set Collision Cell RF	140.0 Vpp	Set Divert Valve	Waste

#	RT [min]	Area	Int. Type	I	S/N	Chromatogram	Max. m/z	FWHM [min]
n.a.	3.4	n.a.	Single spectrum	n.a.	n.a.	n.a.	523.1840	n.a.

+MS, 3.4min #205

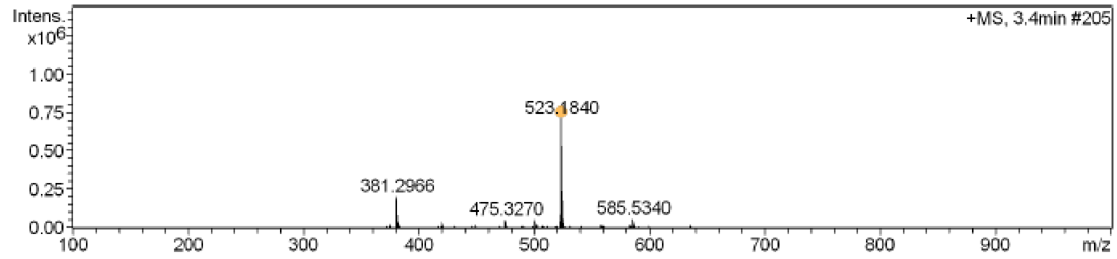

Meas. m/z	#	Ion Formula	m/z	err [ppm]	mSigma	# Sigma	Score	rdb	e⁻ Conf	N-Rule
523.1840	1	C29H28N2NaO6	523.1840	-0.1	6.3	2	100.00	16.5	even	ok

Figure S81. HRMS (ESI-TOF) spectrum of ethyl 3,4-bis(4-methoxyphenyl)-1-[2-(4-methoxyphenyl)-2-oxoethyl]-1*H*-pyrazole-5-carboxylate (**6g**).

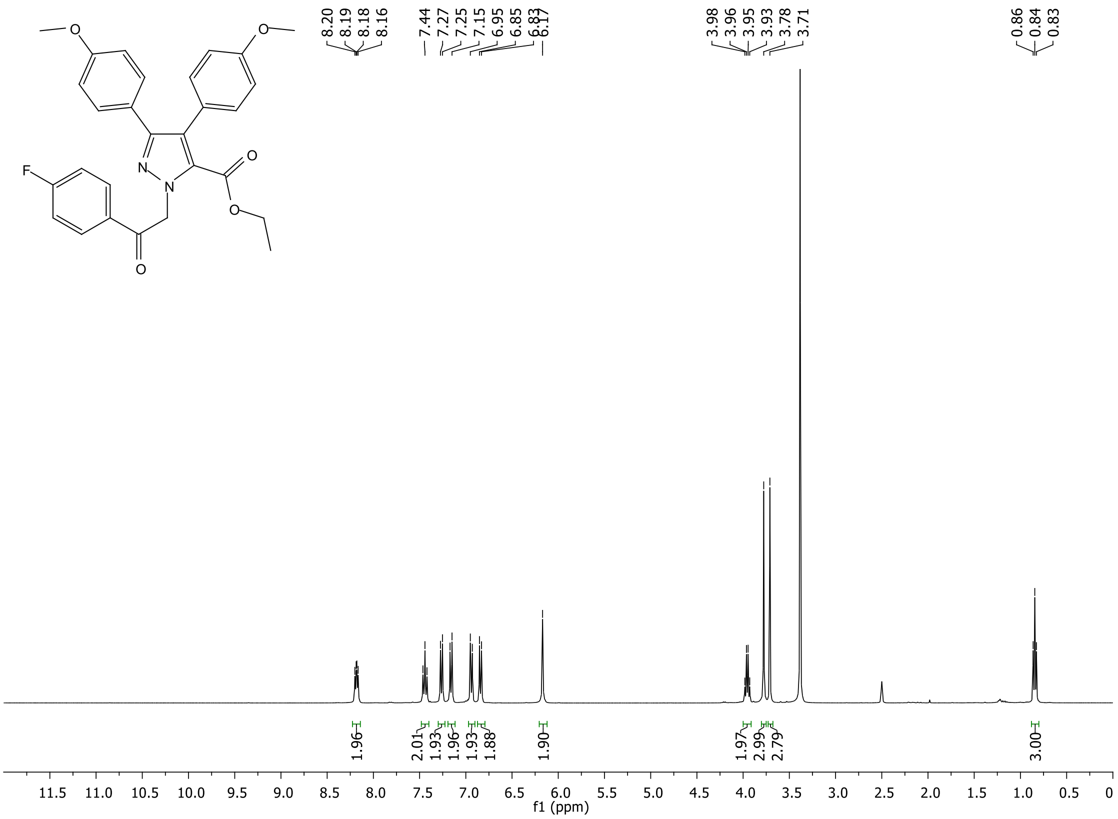

Figure S82. ^{1}H NMR spectrum (400 MHz, DMSO-d_6) of ethyl 1-[2-(4-fluorophenyl)-2-oxoethyl]-3,4-bis(4-methoxyphenyl)-1*H*-pyrazole-5-carboxylate (**6h**).

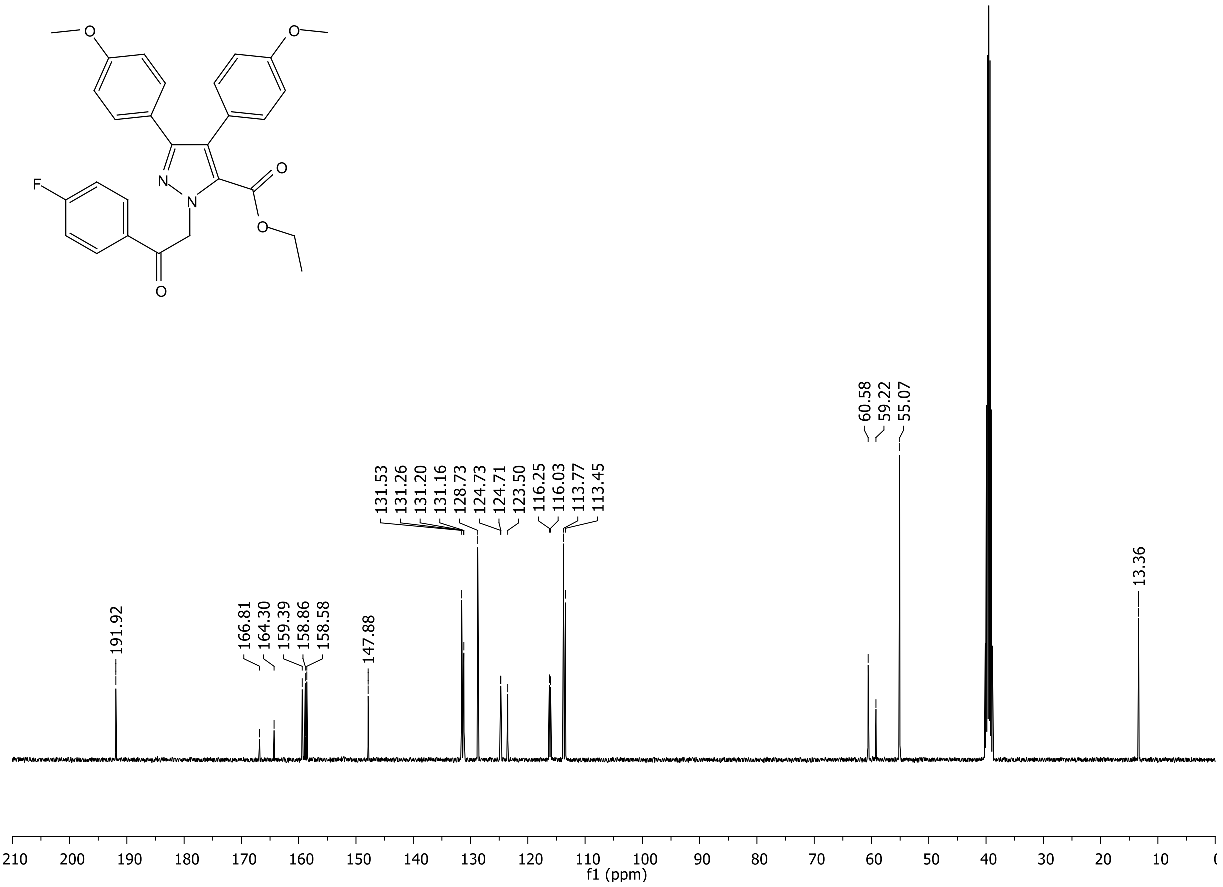

Figure S83. ^{13}C NMR spectrum (101 MHz, DMSO-*d*$_6$) of ethyl 1-[2-(4-fluorophenyl)-2-oxoethyl]-3,4-bis(4-methoxyphenyl)-1*H*-pyrazole-5-carboxylate (**6h**).

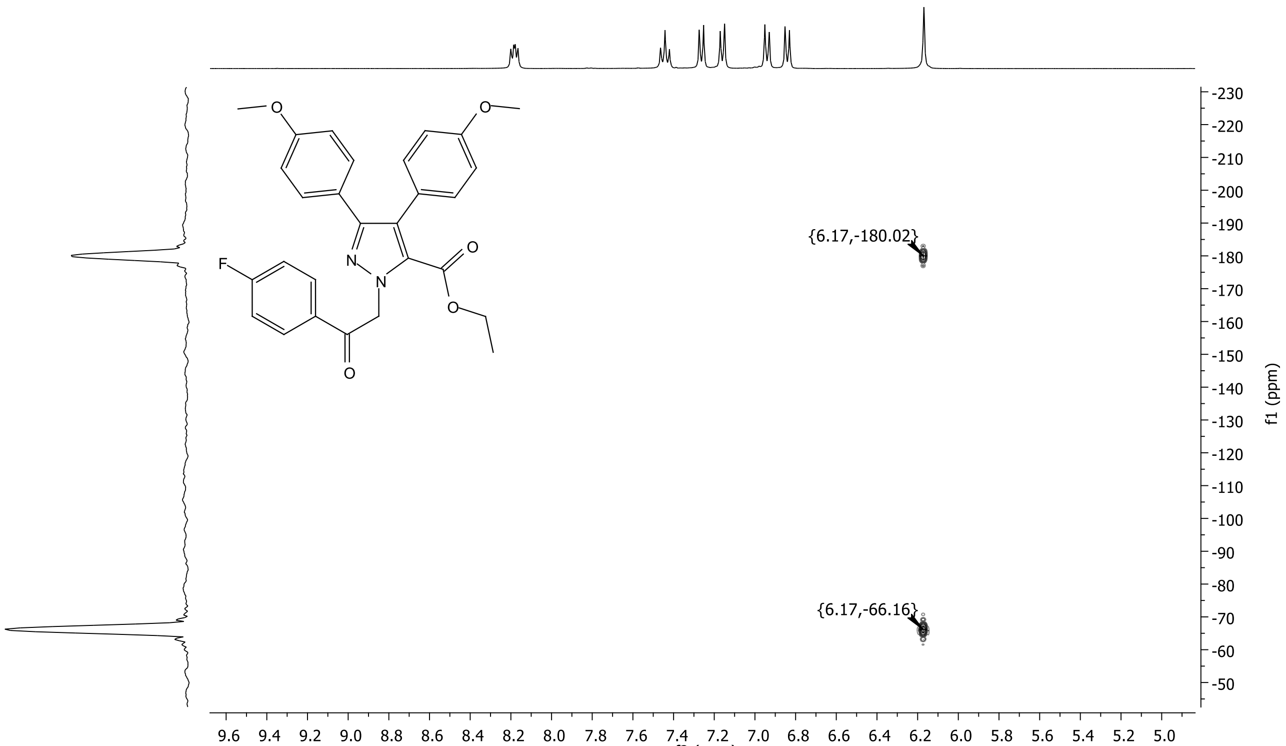

Figure S84. $^{1}H,^{15}N$-HMBC NMR spectrum (40 MHz, DMSO-*d*$_6$) of ethyl 1-[2-(4-fluorophenyl)-2-oxoethyl]-3,4-bis(4-methoxyphenyl)-1*H*-pyrazole-5-carboxylate (**6h**).

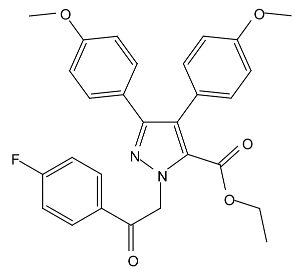

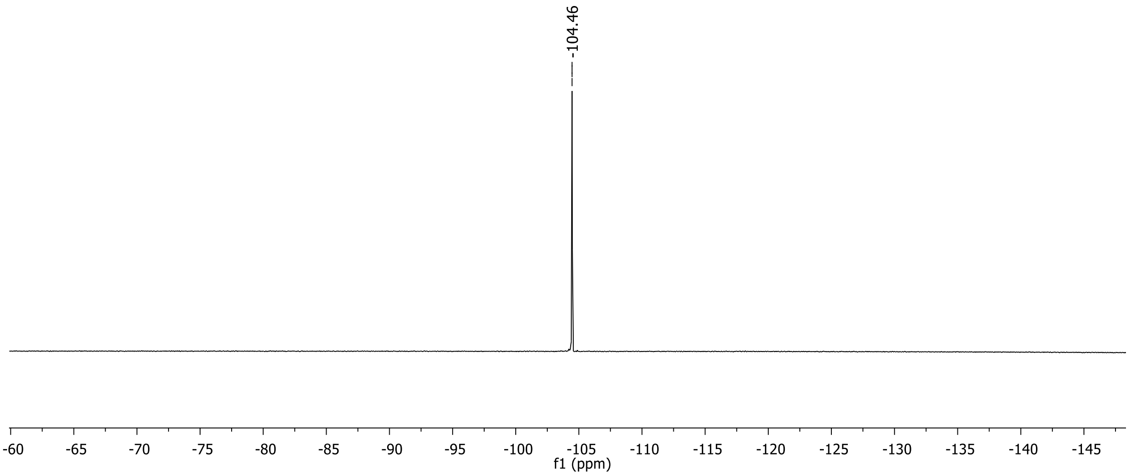

Figure S85. ^{19}F NMR spectrum (376 MHz, DMSO-d_6) of ethyl 1-[2-(4-fluorophenyl)-2-oxoethyl]-3,4-bis(4-methoxyphenyl)-1*H*-pyrazole-5-carboxylate (**6h**).

Compound Spectrum SmartFormula Report

Analysis Info

Analysis Name	D:\Data\KDD-336.d	Acquisition Date	7/29/2022 6:23:53 PM
Method	DirectInfusion_TuneLow_pos.m	Operator	hplc
Sample Name	KDD-336	Instrument	micrOTOF-Q III 8228888.20448
Comment	AB		

Acquisition Parameter

Source Type	ESI	Ion Polarity	Positive	Set Nebulizer	0.4 Bar
Focus	Not active	Set Capillary	4500 V	Set Dry Heater	180 °C
Scan Begin	50 m/z	Set End Plate Offset	-500 V	Set Dry Gas	4.0 l/min
Scan End	1000 m/z	Set Collision Cell RF	140.0 Vpp	Set Divert Valve	Waste

#	RT [min]	Area	Int. Type	I	S/N	Chromatogram	Max. m/z	FWHM [min]
n.a.	8.0	n.a.	Single spectrum	n.a.	n.a.	n.a.	511.1639	n.a.

+MS, 8.0min #480

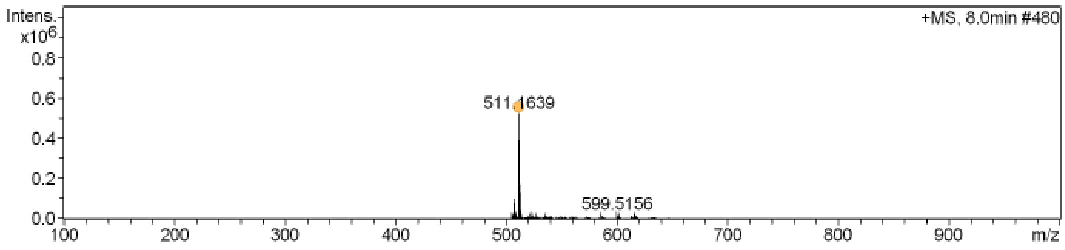

Meas. m/z	#	Ion Formula	m/z	err [ppm]	mSigma	# Sigma	Score	rdb	e⁻ Conf	N-Rule
511.1639	1	C28H25FN2NaO5	511.1640	0.2	5.8	2	100.00	16.5	even	ok

Figure S86. HRMS (ESI-TOF) spectrum of ethyl 1-[2-(4-fluorophenyl)-2-oxoethyl]-3,4-bis(4-methoxyphenyl)-1*H*-pyrazole-5-carboxylate (**6h**).

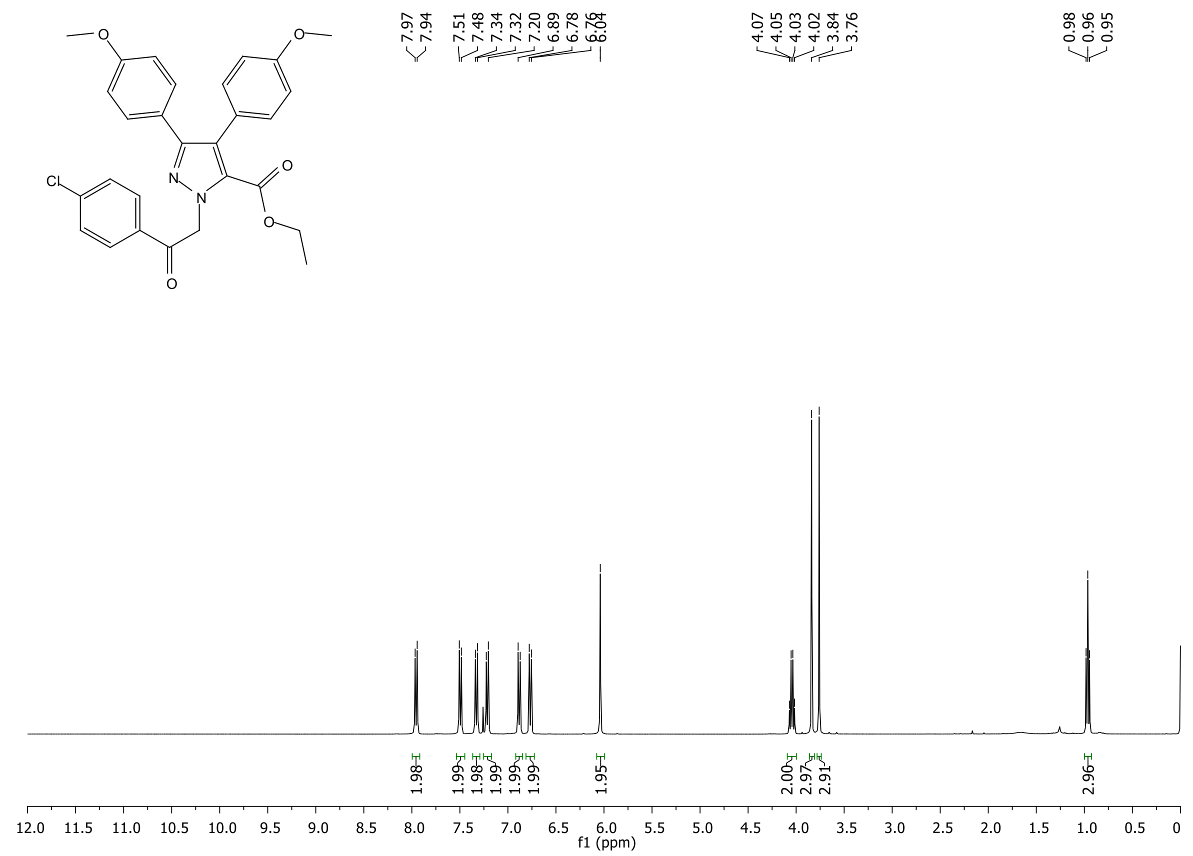

Figure S87. ^{1}H NMR spectrum (400 MHz, $CDCl_3$) of ethyl 1-[2-(4-chlorophenyl)-2-oxoethyl]-3,4-bis(4-methoxyphenyl)-1*H*-pyrazole-5-carboxylate (**6i**).

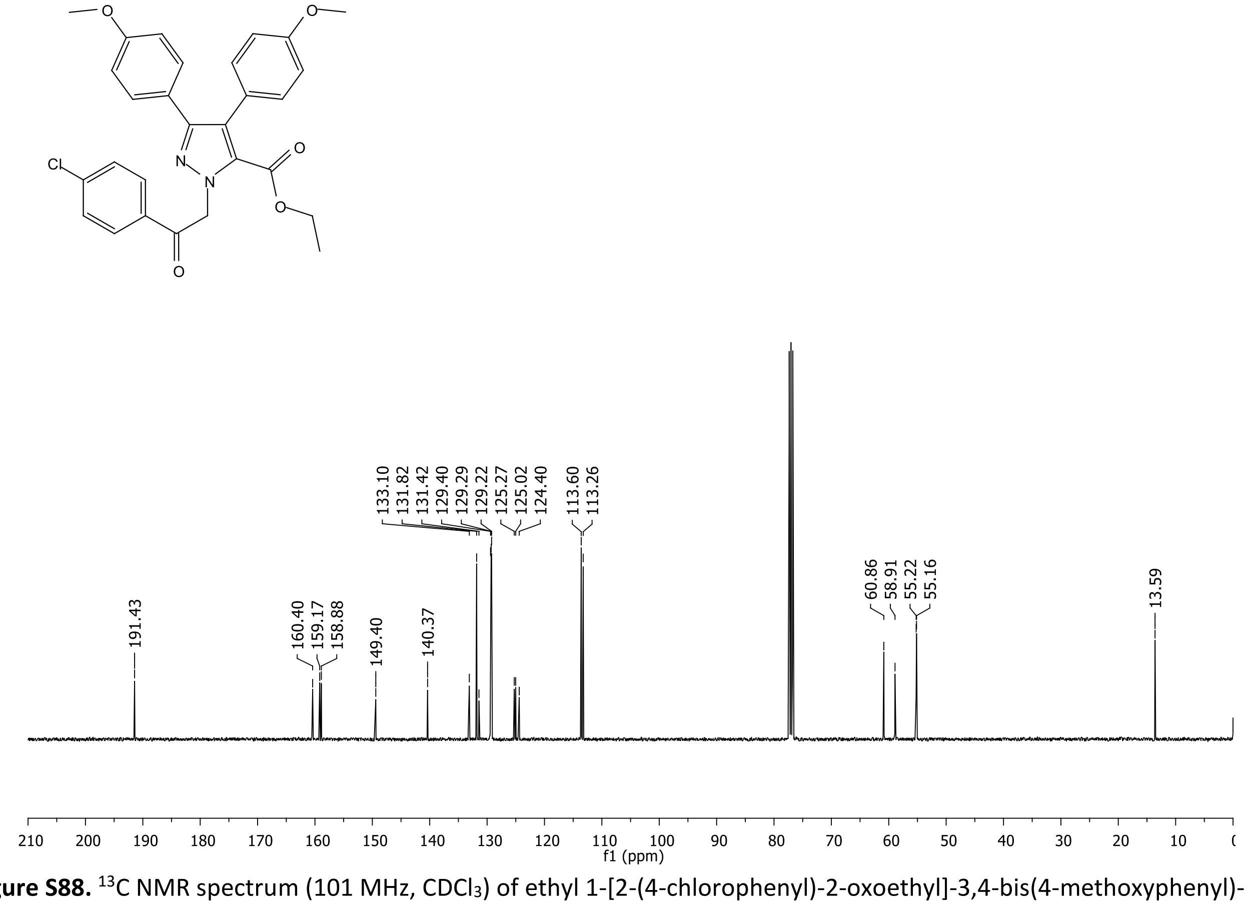

Figure S88. ^{13}C NMR spectrum (101 MHz, $CDCl_3$) of ethyl 1-[2-(4-chlorophenyl)-2-oxoethyl]-3,4-bis(4-methoxyphenyl)-1*H*-pyrazole-5-carboxylate (**6i**).

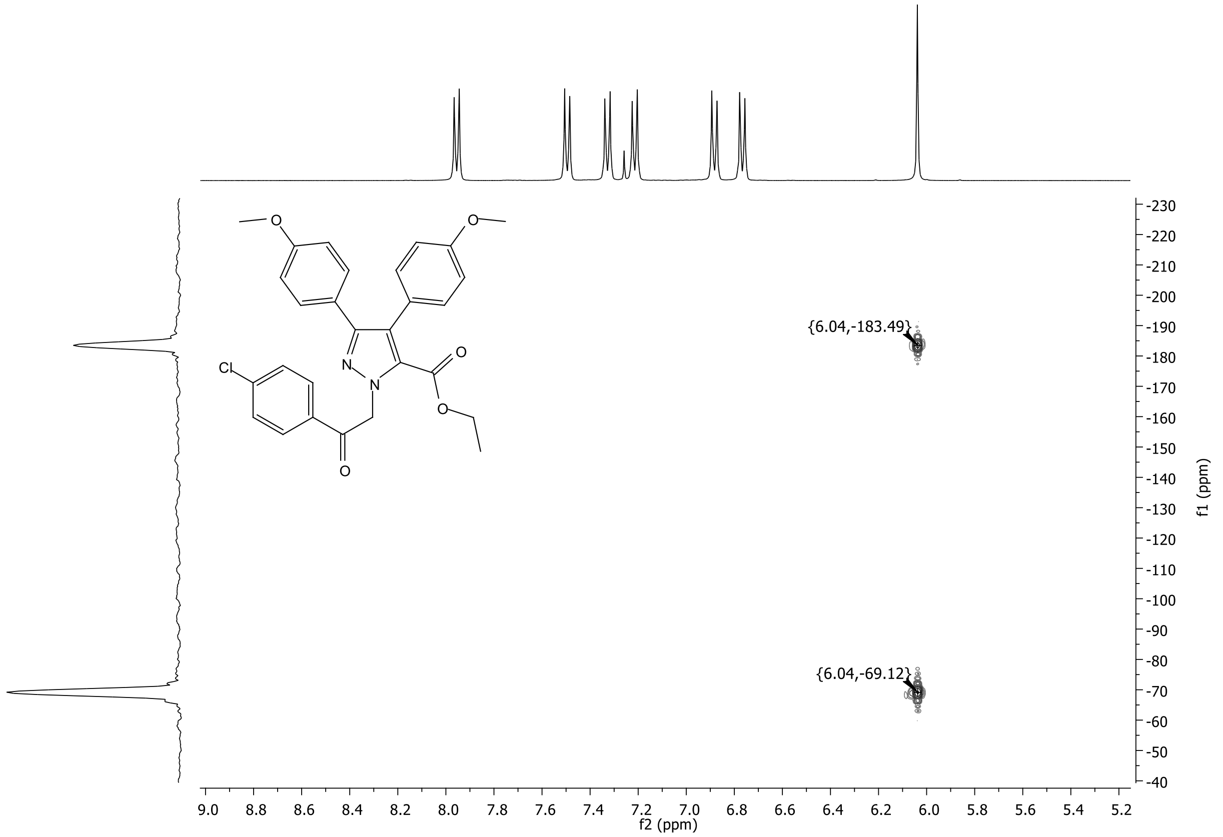

Figure S89. $^{1}H,^{15}N$-HMBC NMR spectrum (40 MHz, $CDCl_3$) of ethyl 1-[2-(4-chlorophenyl)-2-oxoethyl]-3,4-bis(4-methoxyphenyl)-1*H*-pyrazole-5-carboxylate (**6i**).

Compound Spectrum SmartFormula Report

Analysis Info

Analysis Name	D:\Data\KDD-310.d	Acquisition Date	7/29/2022 5:55:33 PM
Method	DirectInfusion_TuneLow_pos.m	Operator	hplc
Sample Name	KDD-310	Instrument	micrOTOF-Q III 8228888.20448
Comment	AB		

Acquisition Parameter

Source Type	ESI	Ion Polarity	Positive	Set Nebulizer	0.4 Bar
Focus	Not active	Set Capillary	4500 V	Set Dry Heater	180 °C
Scan Begin	50 m/z	Set End Plate Offset	-500 V	Set Dry Gas	4.0 l/min
Scan End	1000 m/z	Set Collision Cell RF	140.0 Vpp	Set Divert Valve	Waste

#	RT [min]	Area	Int. Type	I	S/N	Chromatogram	Max. m/z	FWHM [min]
n.a.	5.1	n.a.	Single spectrum	n.a.	n.a.	n.a.	527.1346	n.a.

+MS, 5.1min #305

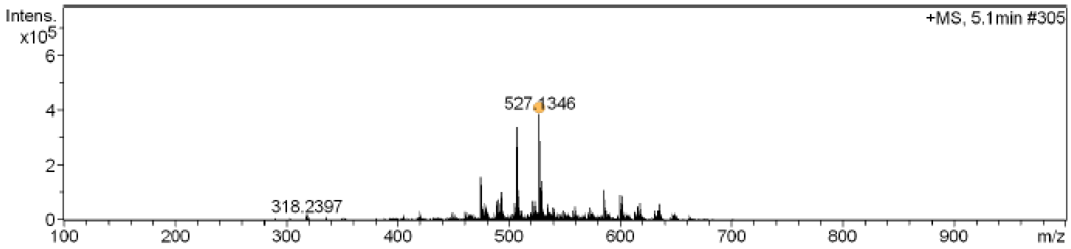

Meas. m/z	#	Ion Formula	m/z	err [ppm]	mSigma	# Sigma	Score	rdb	e⁻ Conf	N-Rule
527.1346	1	C28H25ClN2NaO5	527.1344	0.4	4.4	1	100.00	16.5	even	ok

Figure S90. HRMS (ESI-TOF) spectrum of ethyl 1-[2-(4-chlorophenyl)-2-oxoethyl]-3,4-bis(4-methoxyphenyl)-1*H*-pyrazole-5-carboxylate (**6i**).

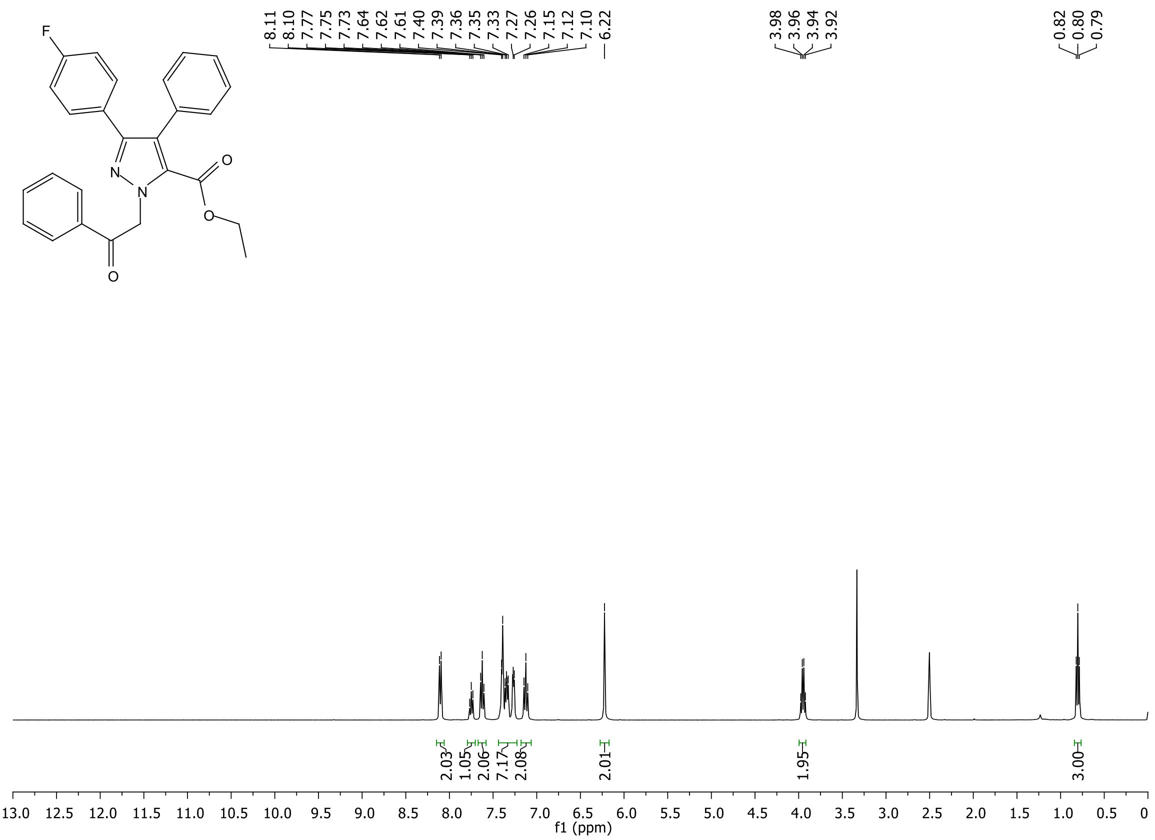

Figure S91. ^{1}H NMR spectrum (400 MHz, DMSO-d_6) of ethyl 3-(4-fluorophenyl)-1-(2-oxo-2-phenylethyl)-4-phenyl-1*H*-pyrazole-5-carboxylate (**6j**).

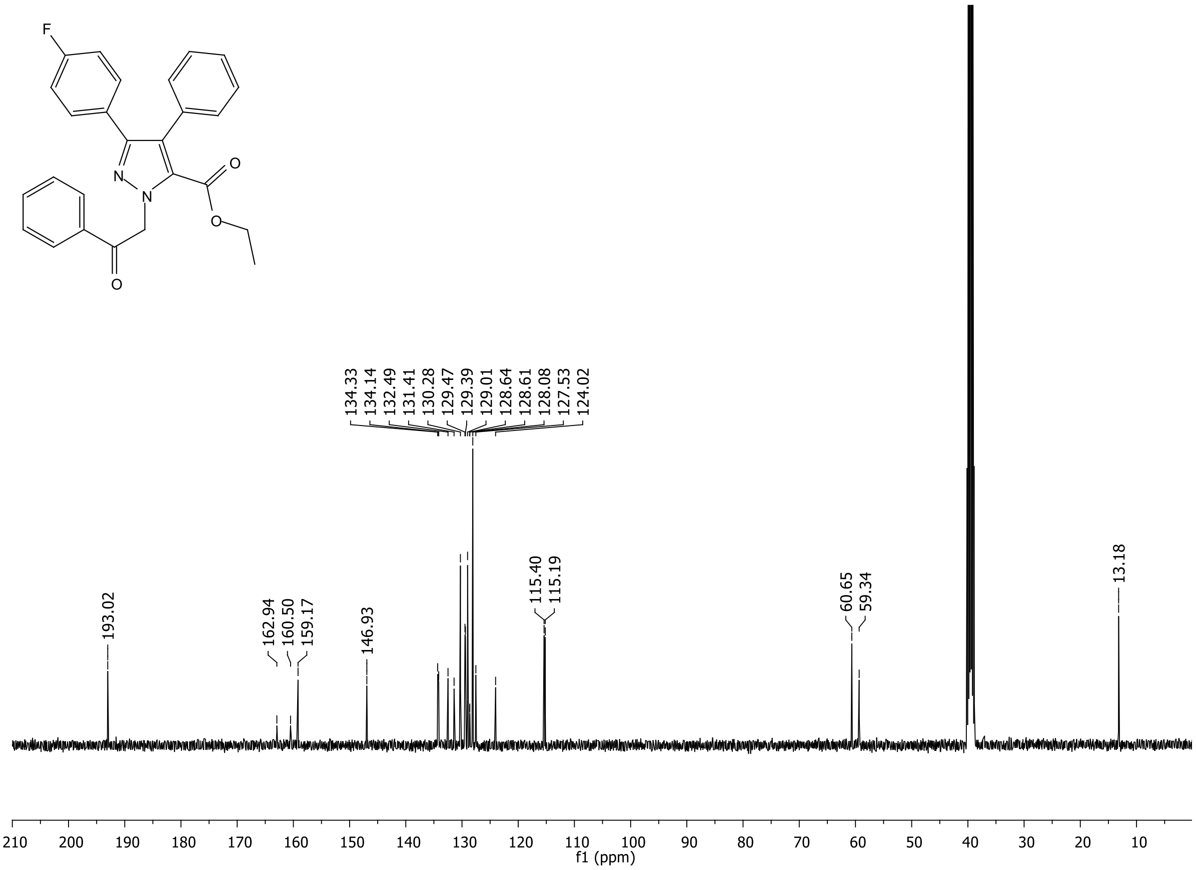

Figure S92. ^{13}C NMR spectrum (101 MHz, DMSO-d_6) of ethyl 3-(4-fluorophenyl)-1-(2-oxo-2-phenylethyl)-4-phenyl-1*H*-pyrazole-5-carboxylate (**6j**).

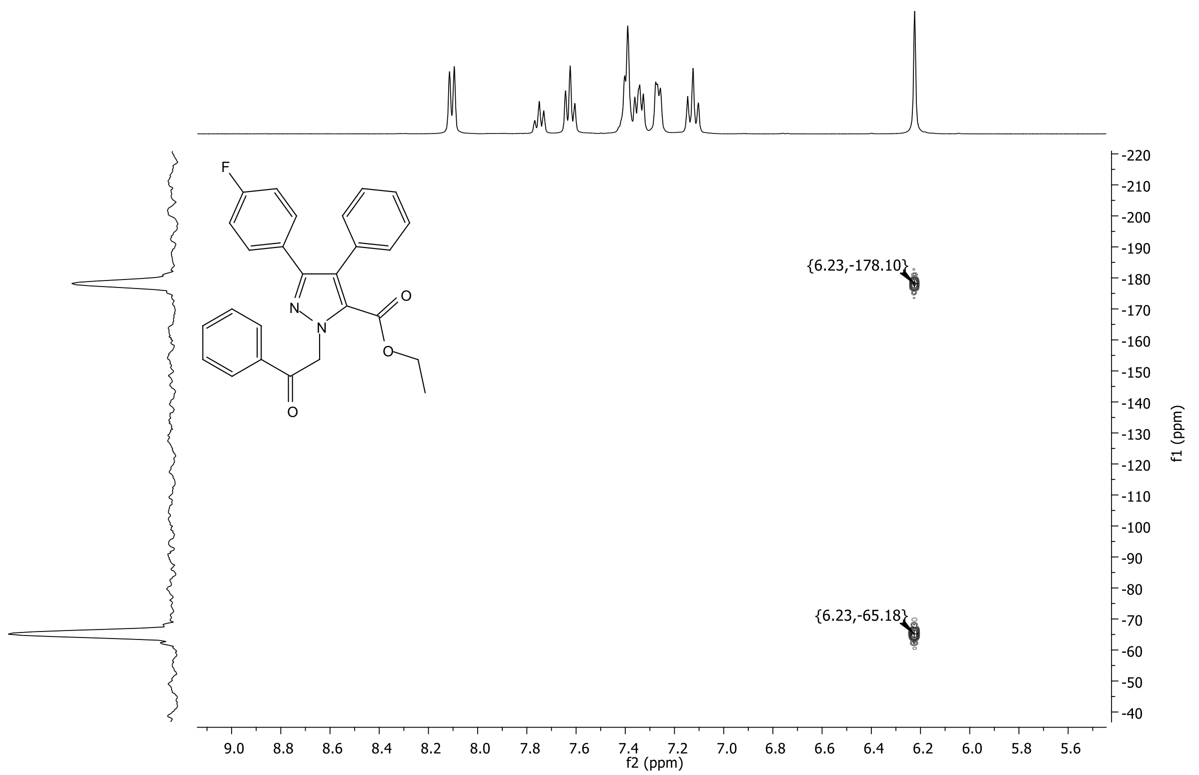

Figure S93. ^{15}N NMR spectrum (40 MHz, DMSO-d_6) of ethyl 3-(4-fluorophenyl)-1-(2-oxo-2-phenylethyl)-4-phenyl-1*H*-pyrazole-5-carboxylate (**6j**).

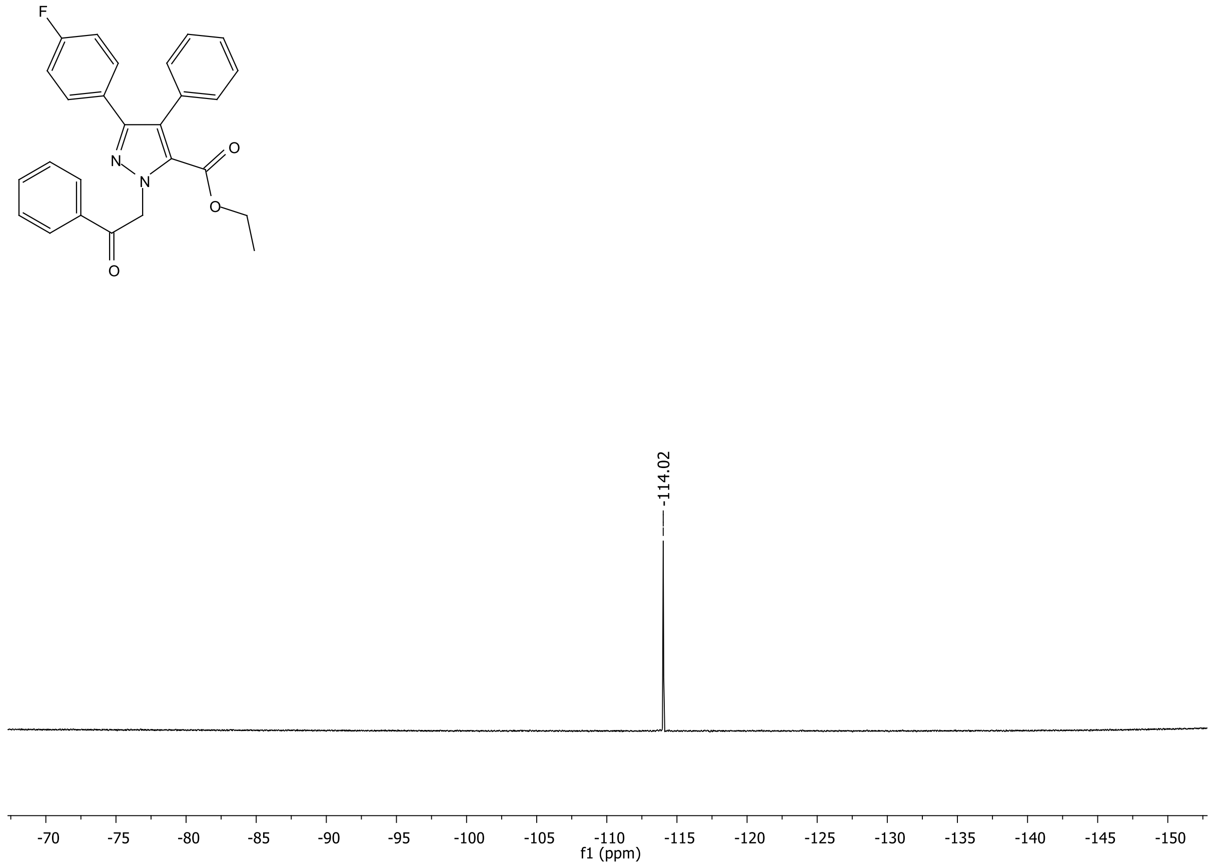

Figure S94. ^{19}F NMR spectrum (376 MHz, DMSO-d_6) of ethyl 3-(4-fluorophenyl)-1-(2-oxo-2-phenylethyl)-4-phenyl-1*H*-pyrazole-5-carboxylate (**6j**).

Compound Spectrum SmartFormula Report

Analysis Info

Analysis Name	D:\Data\KDD-376.d	Acquisition Date	7/6/2022 12:05:14 AM
Method	DirectInfusion_TuneLow_pos.m	Operator	hplc
Sample Name	KDD-376	Instrument	micrOTOF-Q III 8228888.20448
Comment	AB		

Acquisition Parameter

Source Type	ESI	Ion Polarity	Positive	Set Nebulizer	0.4 Bar
Focus	Not active	Set Capillary	4500 V	Set Dry Heater	180 °C
Scan Begin	50 m/z	Set End Plate Offset	-500 V	Set Dry Gas	4.0 l/min
Scan End	1000 m/z	Set Collision Cell RF	140.0 Vpp	Set Divert Valve	Waste

#	RT [min]	Area	Int. Type	I	S/N	Chromatogram	Max. m/z	FWHM [min]
n.a.	3.9	n.a.	Single spectrum	n.a.	n.a.	n.a.	451.1427	n.a.

+MS, 3.9min #231

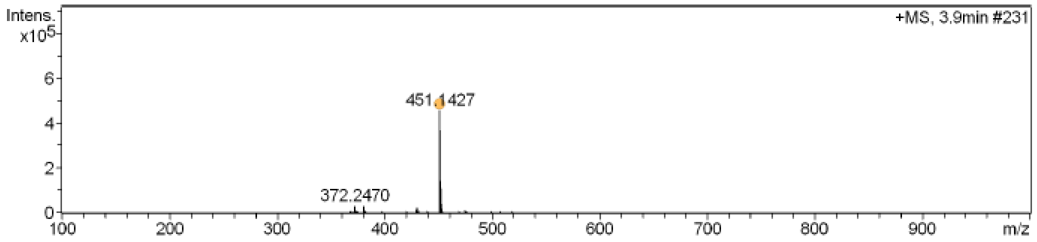

Meas. m/z	#	Ion Formula	m/z	err [ppm]	mSigma	# Sigma	Score	rdb	e⁻ Conf	N-Rule
451.1427	1	C26H21FN2NaO3	451.1428	-0.4	2.6	1	100.00	16.5	even	ok

Figure S95. HRMS (ESI-TOF) spectrum of ethyl 3-(4-fluorophenyl)-1-(2-oxo-2-phenylethyl)-4-phenyl-1*H*-pyrazole-5-carboxylate (**6j**).

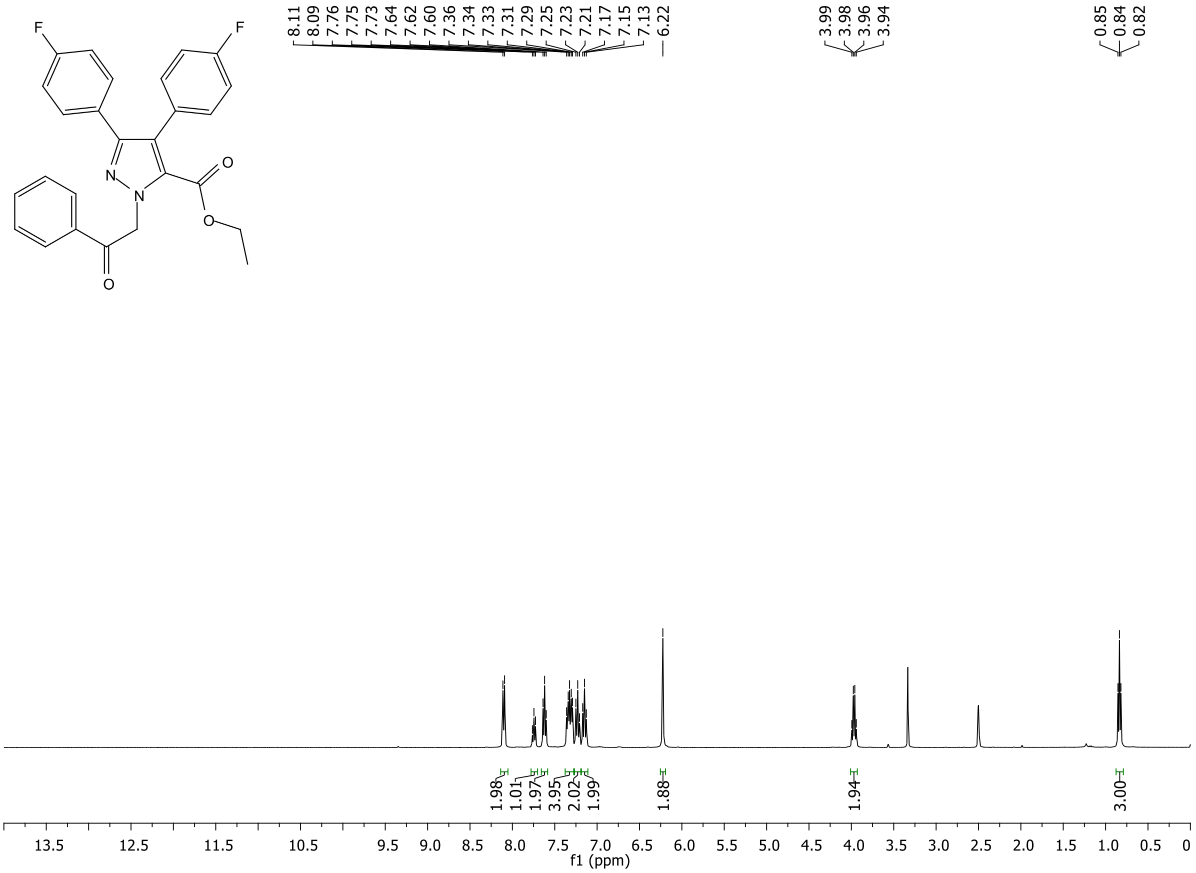

Figure S96. ^{1}H NMR spectrum (400 MHz, DMSO-d_6) of ethyl 3,4-bis(4-fluorophenyl)-1-(2-oxo-2-phenylethyl)-1*H*-pyrazole-5-carboxylate (**6k**).

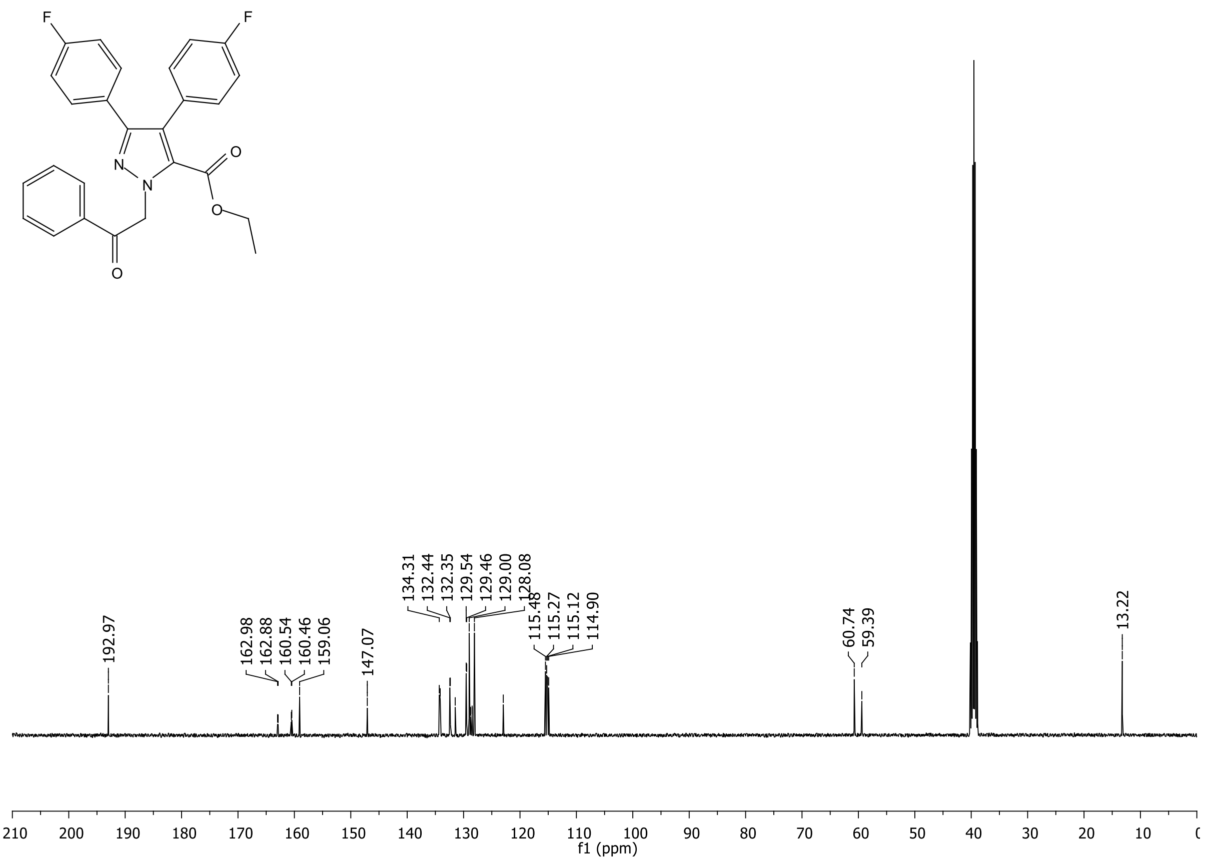

Figure S97. ^{13}C NMR spectrum (101 MHz, DMSO-d_6) of ethyl 3,4-bis(4-fluorophenyl)-1-(2-oxo-2-phenylethyl)-1*H*-pyrazole-5-carboxylate (**6k**).

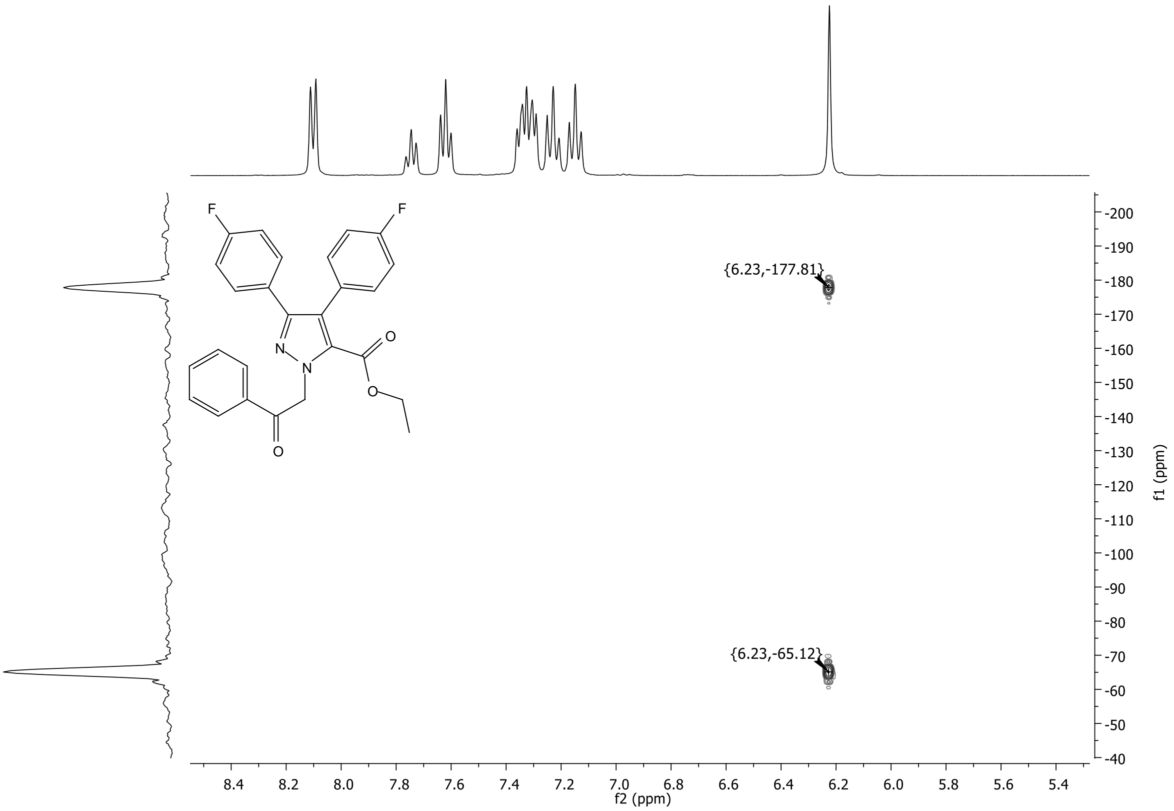

Figure S98. ^{15}N NMR spectrum (40 MHz, DMSO-d_6) of ethyl 3,4-bis(4-fluorophenyl)-1-(2-oxo-2-phenylethyl)-1*H*-pyrazole-5-carboxylate (**6k**).

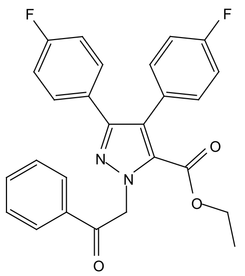

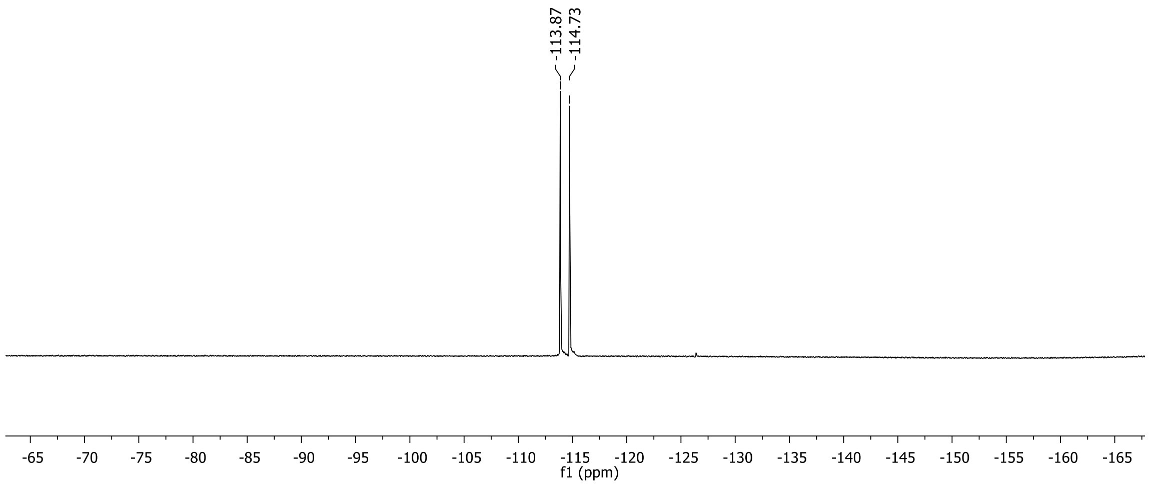

Figure S99. ^{19}F NMR spectrum (376 MHz, DMSO-d_6) of ethyl 3,4-bis(4-fluorophenyl)-1-(2-oxo-2-phenylethyl)-1*H*-pyrazole-5-carboxylate (**6k**).

Compound Spectrum SmartFormula Report

Analysis Info

Analysis Name	D:\Data\KDD-375.d	Acquisition Date	7/29/2022 7:30:19 PM
Method	DirectInfusion_TuneLow_pos.m	Operator	hplc
Sample Name	KDD-375	Instrument	micrOTOF-Q III 8228888.20448
Comment	AB		

Acquisition Parameter

Source Type	ESI	Ion Polarity	Positive	Set Nebulizer	0.4 Bar
Focus	Not active	Set Capillary	4500 V	Set Dry Heater	180 °C
Scan Begin	50 m/z	Set End Plate Offset	-500 V	Set Dry Gas	4.0 l/min
Scan End	1000 m/z	Set Collision Cell RF	140.0 Vpp	Set Divert Valve	Waste

#	RT [min]	Area	Int. Type	I	S/N	Chromatogram	Max. m/z	FWHM [min]
n.a.	3.3	n.a.	Single spectrum	n.a.	n.a.	n.a.	469.1334	n.a.

+MS, 3.3min #197

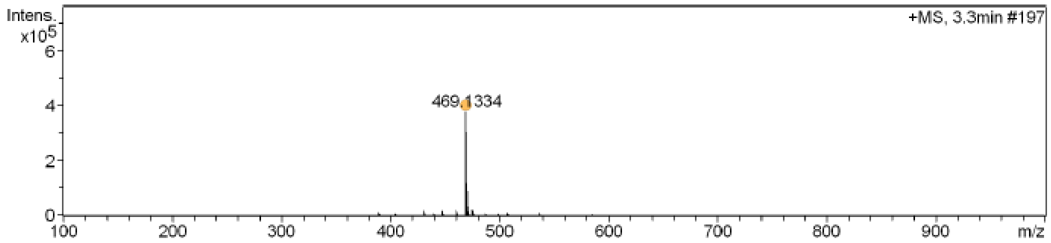

Meas. m/z	#	Ion Formula	m/z	err [ppm]	mSigma	# Sigma	Score	rdb	e⁻ Conf	N-Rule
469.1334	1	C26H20F2N2NaO3	469.1334	0.1	2.1	1	100.00	16.5	even	ok

Figure S100. HRMS (ESI-TOF) spectrum of ethyl 3,4-bis(4-fluorophenyl)-1-(2-oxo-2-phenylethyl)-1*H*-pyrazole-5-carboxylate (**6k**).

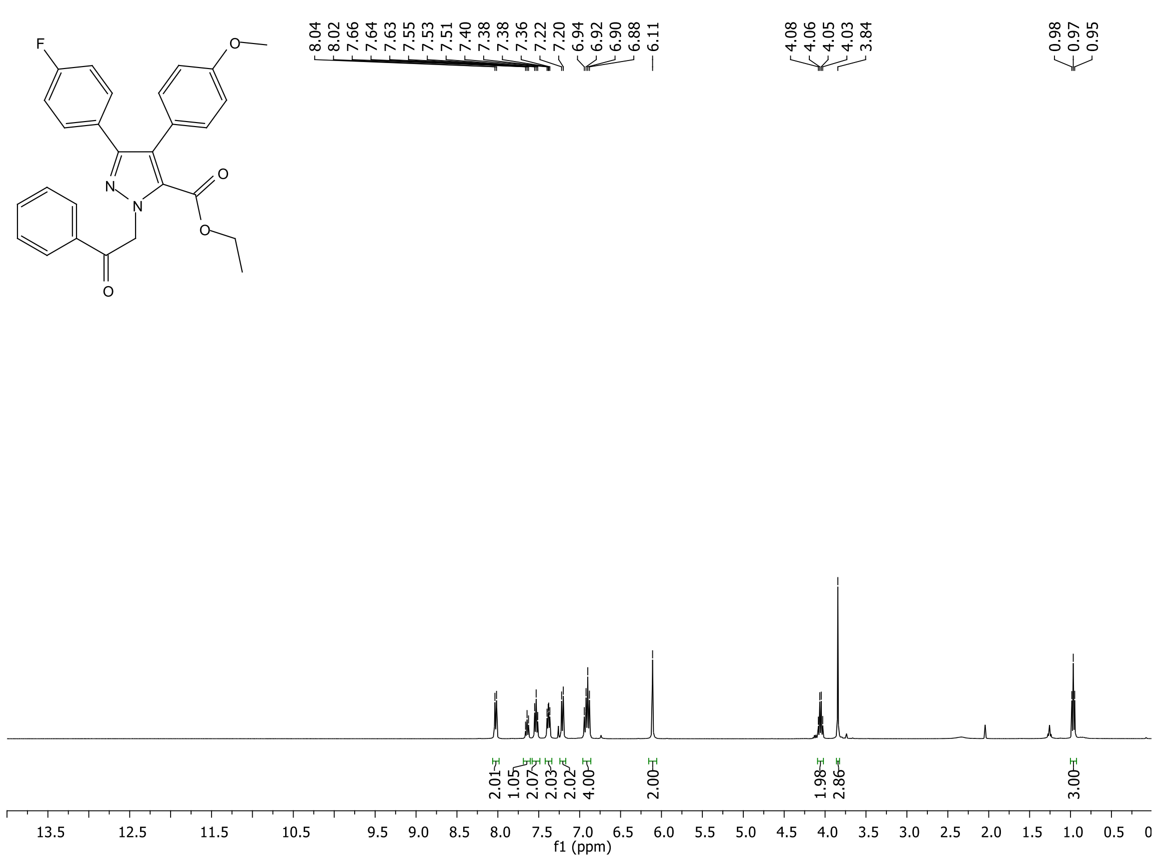

Figure S101. ^{1}H NMR spectrum (400 MHz, $CDCl_3$) of ethyl 3-(4-fluorophenyl)-4-(4-methoxyphenyl)-1-(2-oxo-2-phenylethyl)-1*H*-pyrazole-5-carboxylate (**6l**).

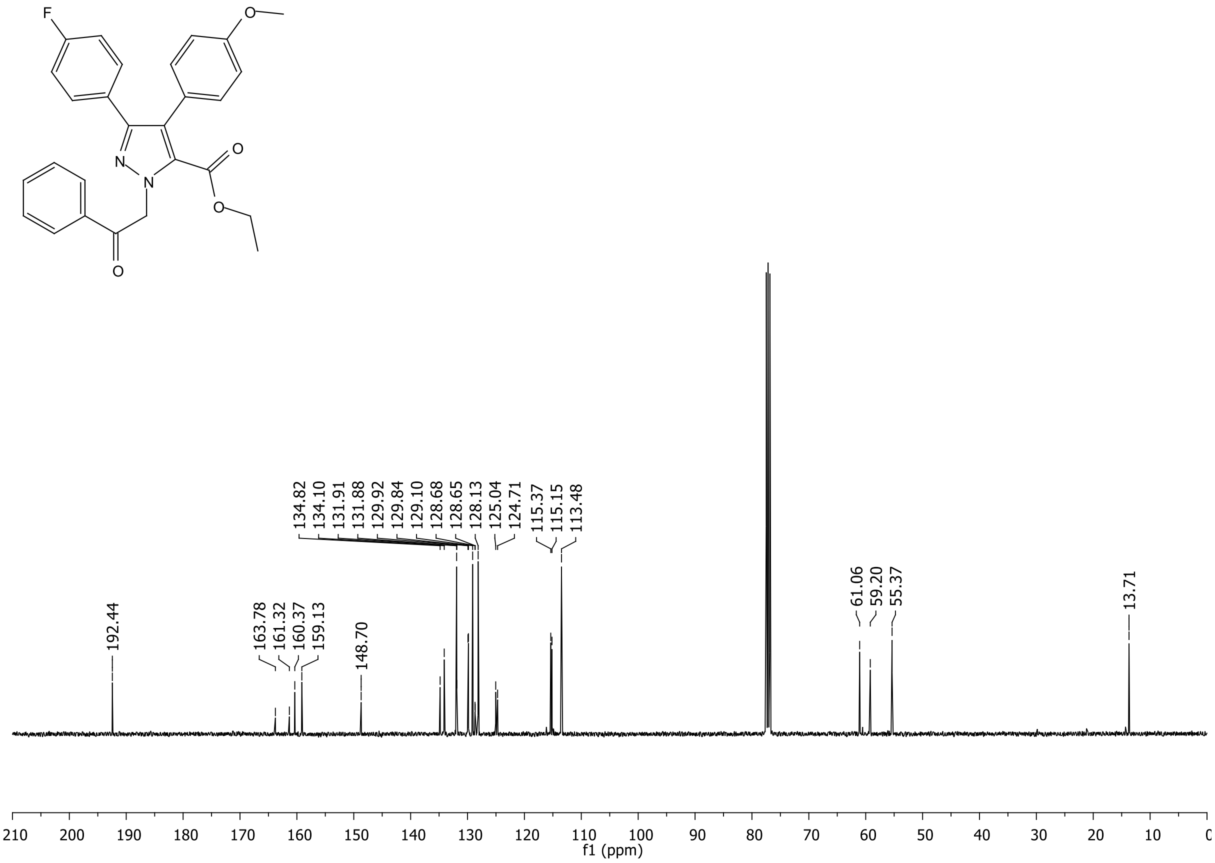

Figure S102. ^{13}C NMR spectrum (101 MHz, $CDCl_3$) of ethyl 3-(4-fluorophenyl)-4-(4-methoxyphenyl)-1-(2-oxo-2-phenylethyl)-1*H*-pyrazole-5-carboxylate (**6l**).

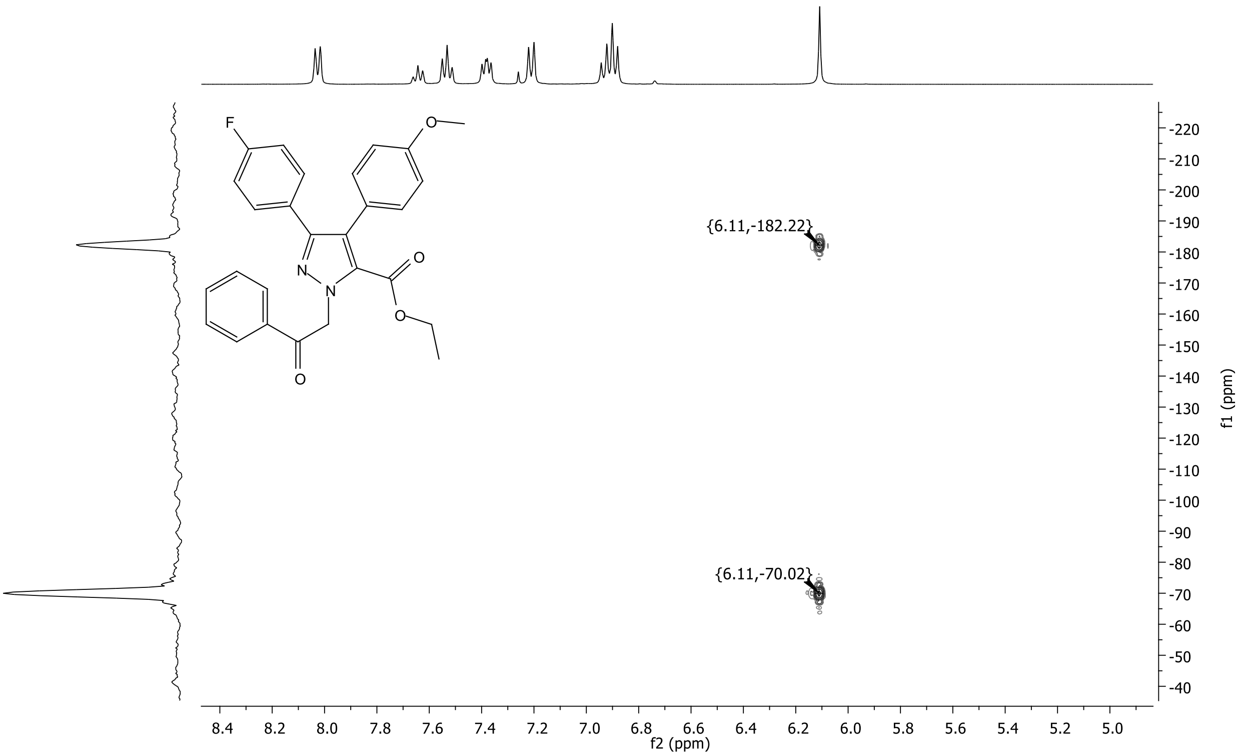

Figure S103. ^{15}N NMR spectrum (40 MHz, $CDCl_3$) of ethyl 3-(4-fluorophenyl)-4-(4-methoxyphenyl)-1-(2-oxo-2-phenylethyl)-1*H*-pyrazole-5-carboxylate (**6l**).

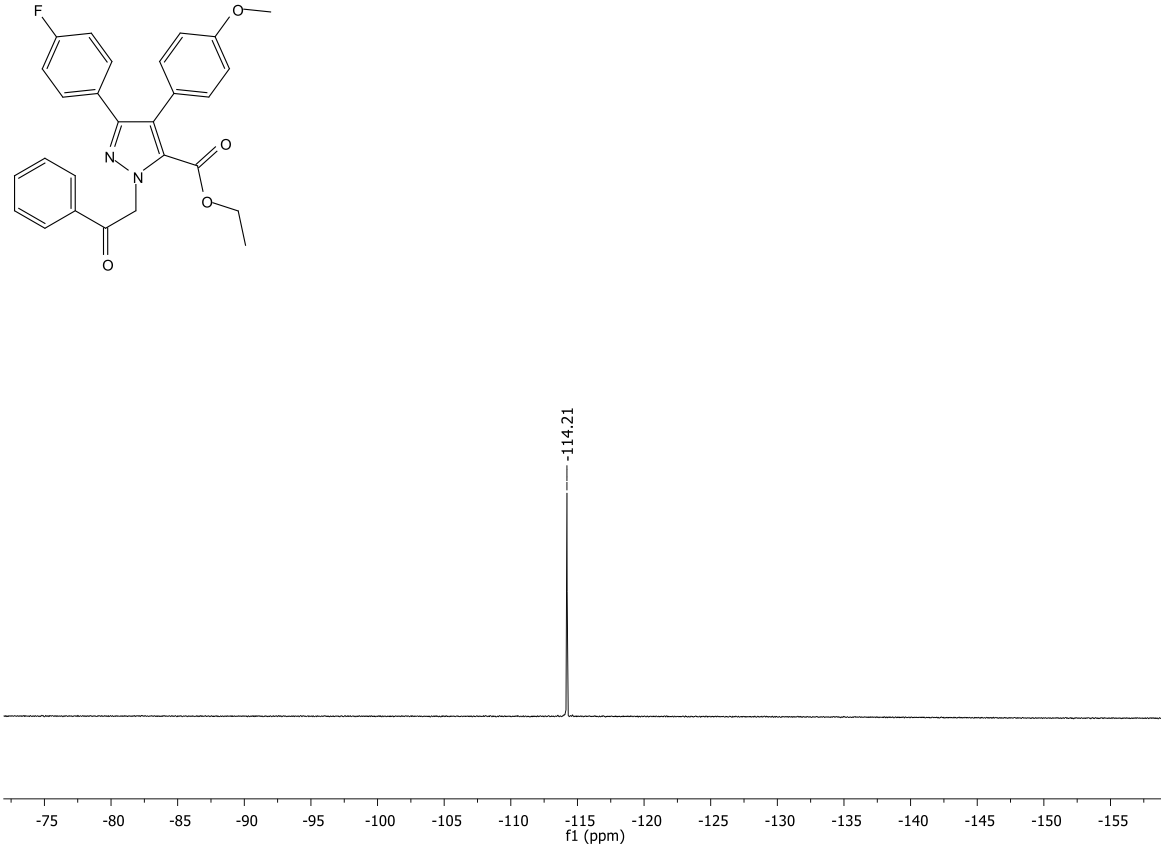

Figure S104. ^{19}F NMR spectrum (376 MHz, $CDCl_3$) of ethyl 3-(4-fluorophenyl)-4-(4-methoxyphenyl)-1-(2-oxo-2-phenylethyl)-1*H*-pyrazole-5-carboxylate (**6l**).

Compound Spectrum SmartFormula Report

Analysis Info

Analysis Name	D:\Data\KDD-382.d	Acquisition Date	7/6/2022 12:17:15 AM
Method	DirectInfusion_TuneLow_pos.m	Operator	hplc
Sample Name	KDD-382	Instrument	micrOTOF-Q III 8228888.20448
Comment	AB		

Acquisition Parameter

Source Type	ESI	Ion Polarity	Positive	Set Nebulizer	0.4 Bar
Focus	Not active	Set Capillary	4500 V	Set Dry Heater	180 °C
Scan Begin	50 m/z	Set End Plate Offset	-500 V	Set Dry Gas	4.0 l/min
Scan End	1000 m/z	Set Collision Cell RF	140.0 Vpp	Set Divert Valve	Waste

#	RT [min]	Area	Int. Type	I	S/N	Chromatogram	Max. m/z	FWHM [min]
n.a.	2.9	n.a.	Single spectrum	n.a.	n.a.	n.a.	481.1535	n.a.

+MS, 2.9min #174

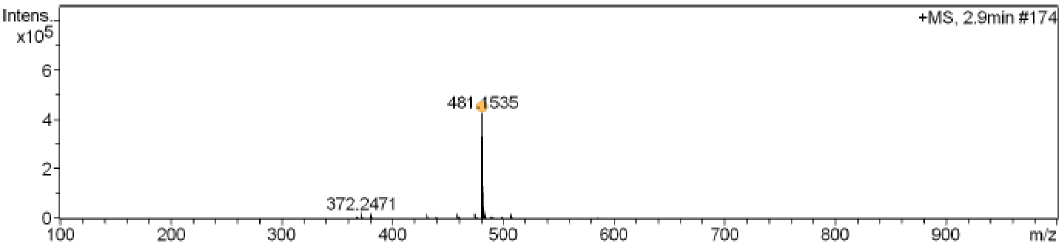

Meas. m/z	#	Ion Formula	m/z	err [ppm]	mSigma	# Sigma	Score	rdb	e⁻ Conf	N-Rule
481.1535	1	C27H23FN2NaO4	481.1534	0.1	3.1	2	100.00	16.5	even	ok

Figure S105. HRMS (ESI-TOF) spectrum of ethyl 3-(4-fluorophenyl)-4-(4-methoxyphenyl)-1-(2-oxo-2-phenylethyl)-1*H*-pyrazole-5-carboxylate (**6l**).

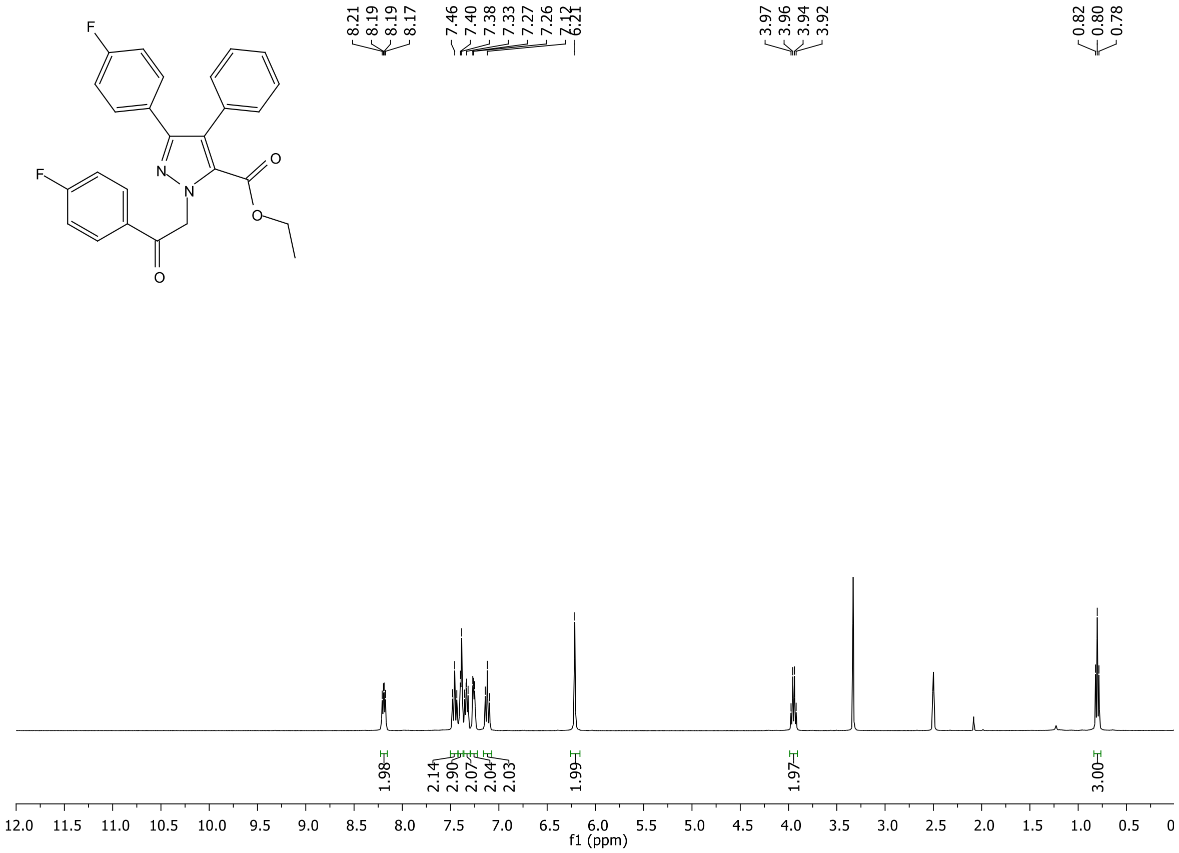

Figure S106. ^{1}H NMR spectrum (400 MHz, DMSO-d_6) of ethyl 3-(4-fluorophenyl)-1-[2-(4-fluorophenyl)-2-oxoethyl]-4-phenyl-1*H*-pyrazole-5-carboxylate (**6m**).

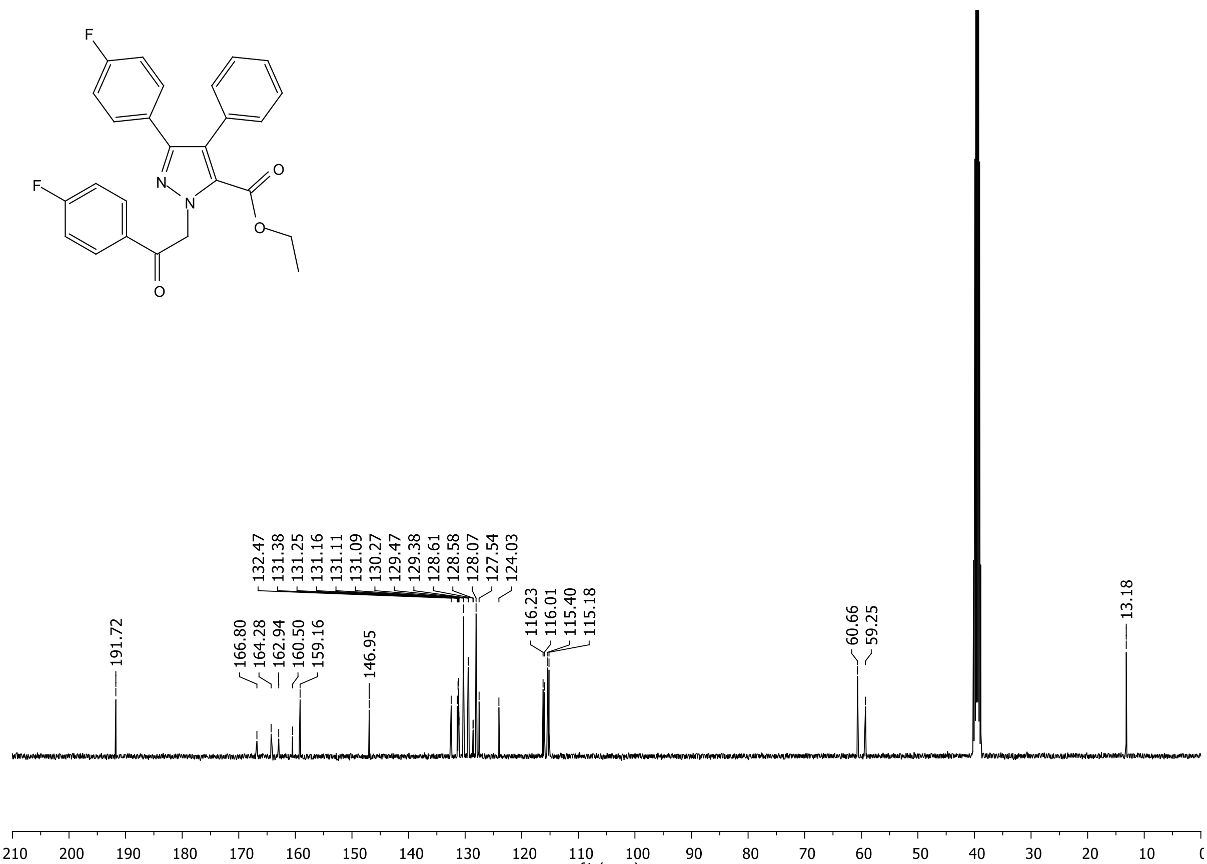

Figure S107. ^{13}C NMR spectrum (101 MHz, DMSO-d_6) of ethyl 3-(4-fluorophenyl)-1-[2-(4-fluorophenyl)-2-oxoethyl]-4-phenyl-1*H*-pyrazole-5-carboxylate (**6m**).

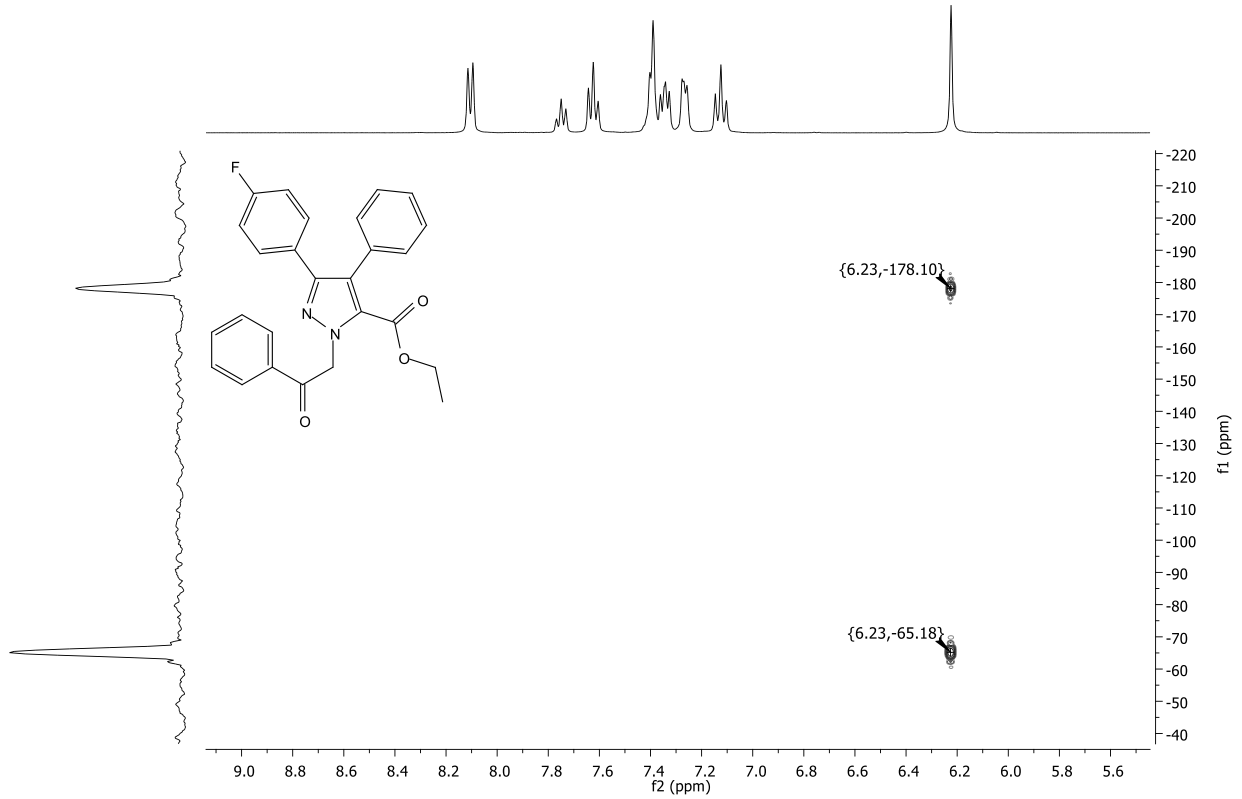

Figure S108. ^{15}N NMR spectrum (40 MHz, DMSO-d_6) of ethyl 3-(4-fluorophenyl)-1-[2-(4-fluorophenyl)-2-oxoethyl]-4-phenyl-1*H*-pyrazole-5-carboxylate (**6m**).

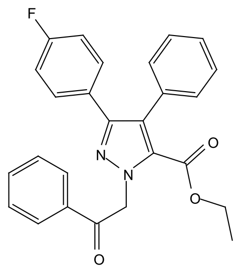

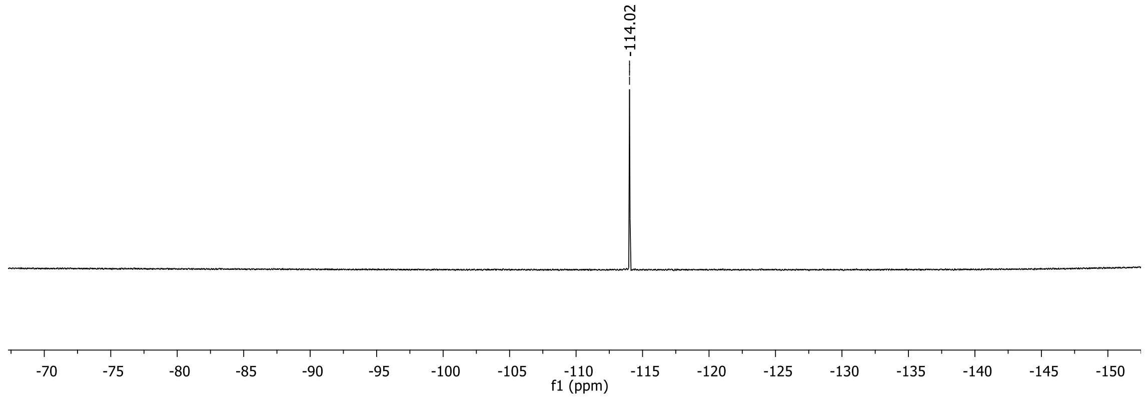

Figure S109. ^{19}F NMR spectrum (376 MHz, DMSO-d_6) of ethyl 3-(4-fluorophenyl)-1-[2-(4-fluorophenyl)-2-oxoethyl]-4-phenyl-1*H*-pyrazole-5-carboxylate (**6m**).

Compound Spectrum SmartFormula Report

Analysis Info

Analysis Name	D:\Data\KDD-384.d	Acquisition Date	7/29/2022 7:56:41 PM
Method	DirectInfusion_TuneLow_pos.m	Operator	hplc
Sample Name	KDD-384	Instrument	micrOTOF-Q III 8228888.20448
Comment	AB		

Acquisition Parameter

Source Type	ESI	Ion Polarity	Positive	Set Nebulizer	0.4 Bar
Focus	Not active	Set Capillary	4500 V	Set Dry Heater	180 °C
Scan Begin	50 m/z	Set End Plate Offset	-500 V	Set Dry Gas	4.0 l/min
Scan End	1000 m/z	Set Collision Cell RF	140.0 Vpp	Set Divert Valve	Waste

#	RT [min]	Area	Int. Type	I	S/N	Chromatogram	Max. m/z	FWHM [min]
n.a.	4.7	n.a.	Single spectrum	n.a.	n.a.	n.a.	469.1332	n.a.

+MS, 4.7min #282

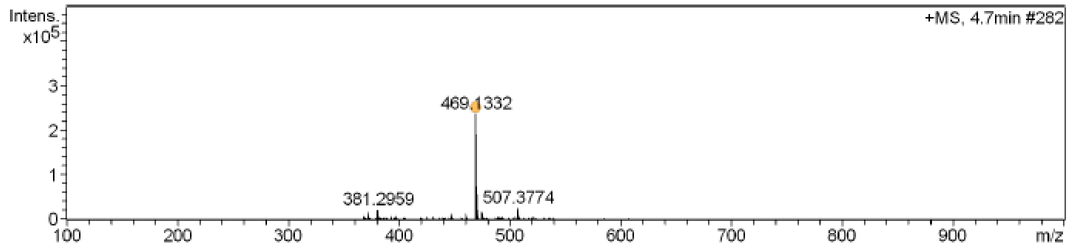

Meas. m/z	#	Ion Formula	m/z	err [ppm]	mSigma	# Sigma	Score	rdb	e⁻ Conf	N-Rule
469.1332	1	C26H20F2N2NaO3	469.1334	-0.5	2.4	1	100.00	16.5	even	ok

Figure S110. HRMS (ESI-TOF) spectrum of ethyl 3-(4-fluorophenyl)-1-[2-(4-fluorophenyl)-2-oxoethyl]-4-phenyl-1*H*-pyrazole-5-carboxylate (**6m**).

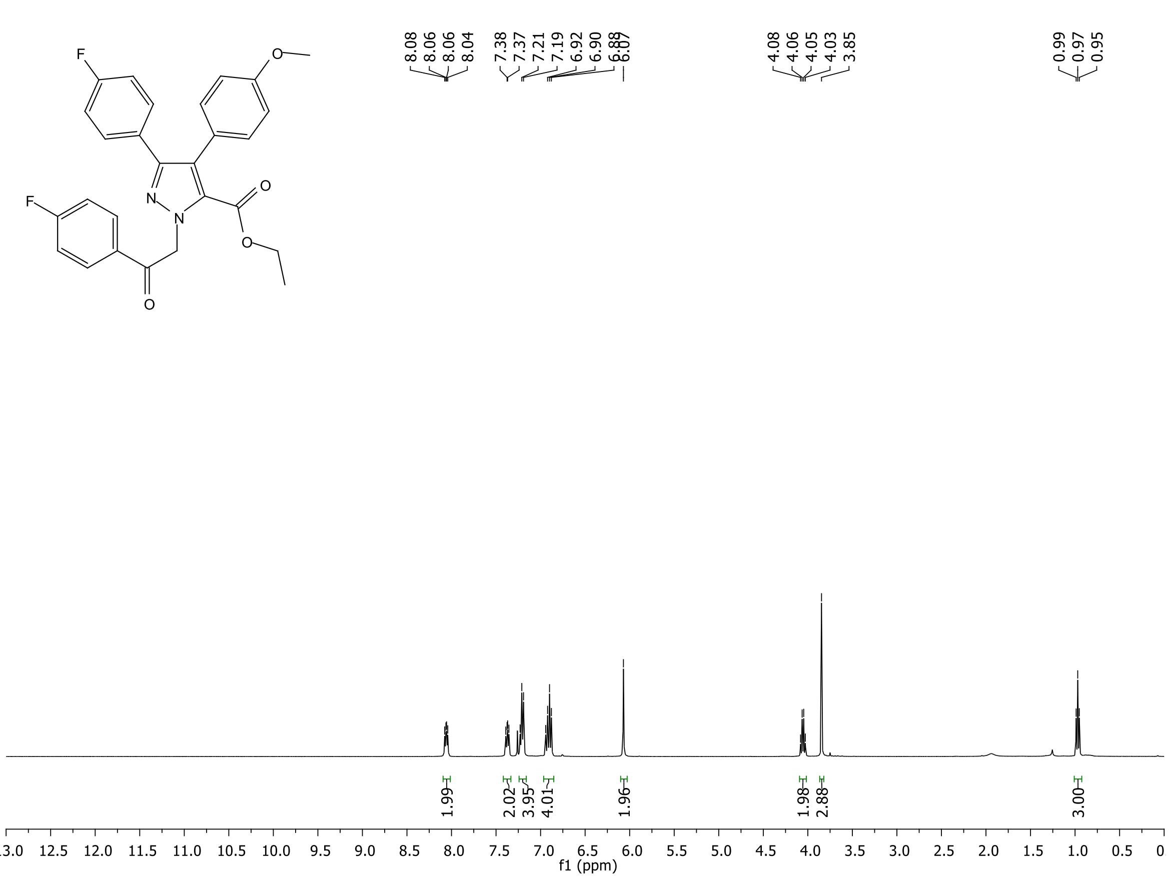

Figure S111. ^{1}H NMR spectrum (400 MHz, $CDCl_3$) of ethyl 3-(4-fluorophenyl)-1-[2-(4-fluorophenyl)-2-oxoethyl]-4-(4-methoxyphenyl)-1*H*-pyrazole-5-carboxylate (**6n**).

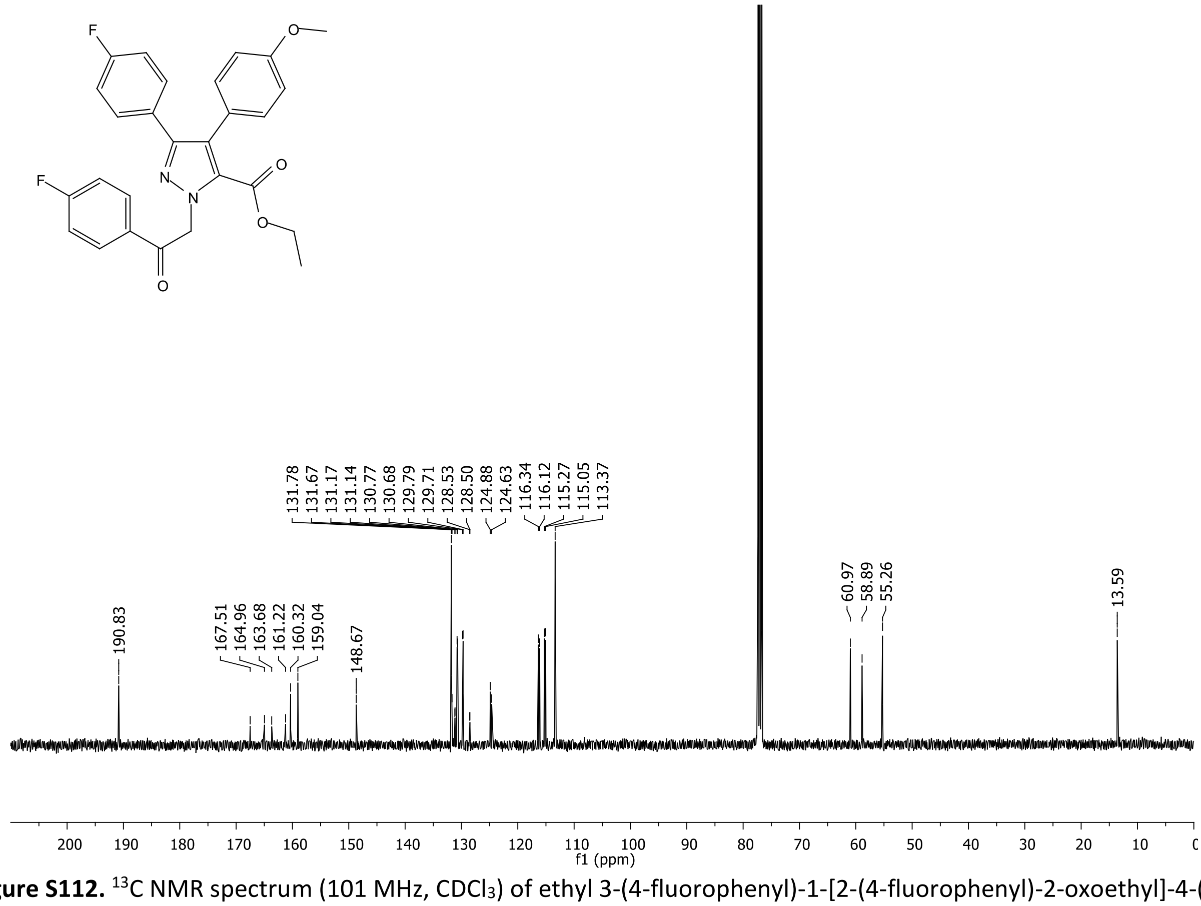

Figure S112. ^{13}C NMR spectrum (101 MHz, $CDCl_3$) of ethyl 3-(4-fluorophenyl)-1-[2-(4-fluorophenyl)-2-oxoethyl]-4-(4-methoxyphenyl)-1*H*-pyrazole-5-carboxylate (**6n**).

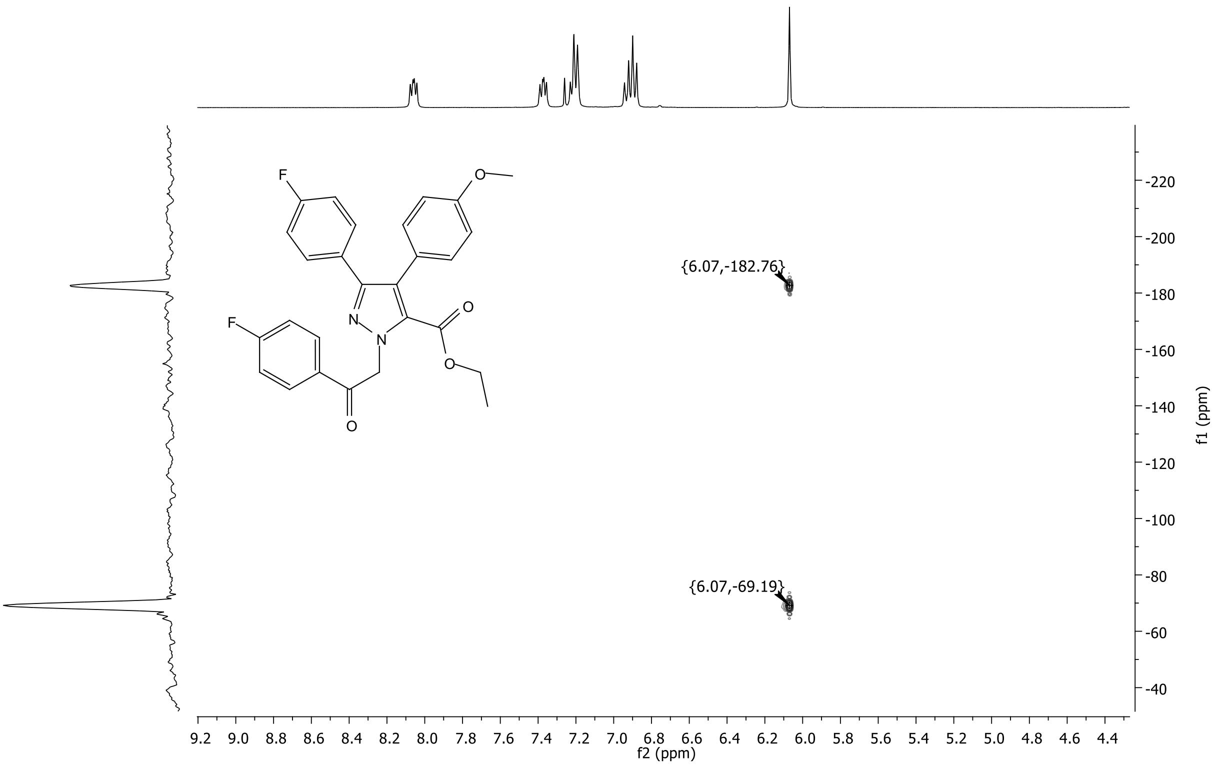

Figure S113. ^{15}N NMR spectrum (40 MHz, $CDCl_3$) of ethyl 3-(4-fluorophenyl)-1-[2-(4-fluorophenyl)-2-oxoethyl]-4-(4-methoxyphenyl)-1*H*-pyrazole-5-carboxylate (**6n**).

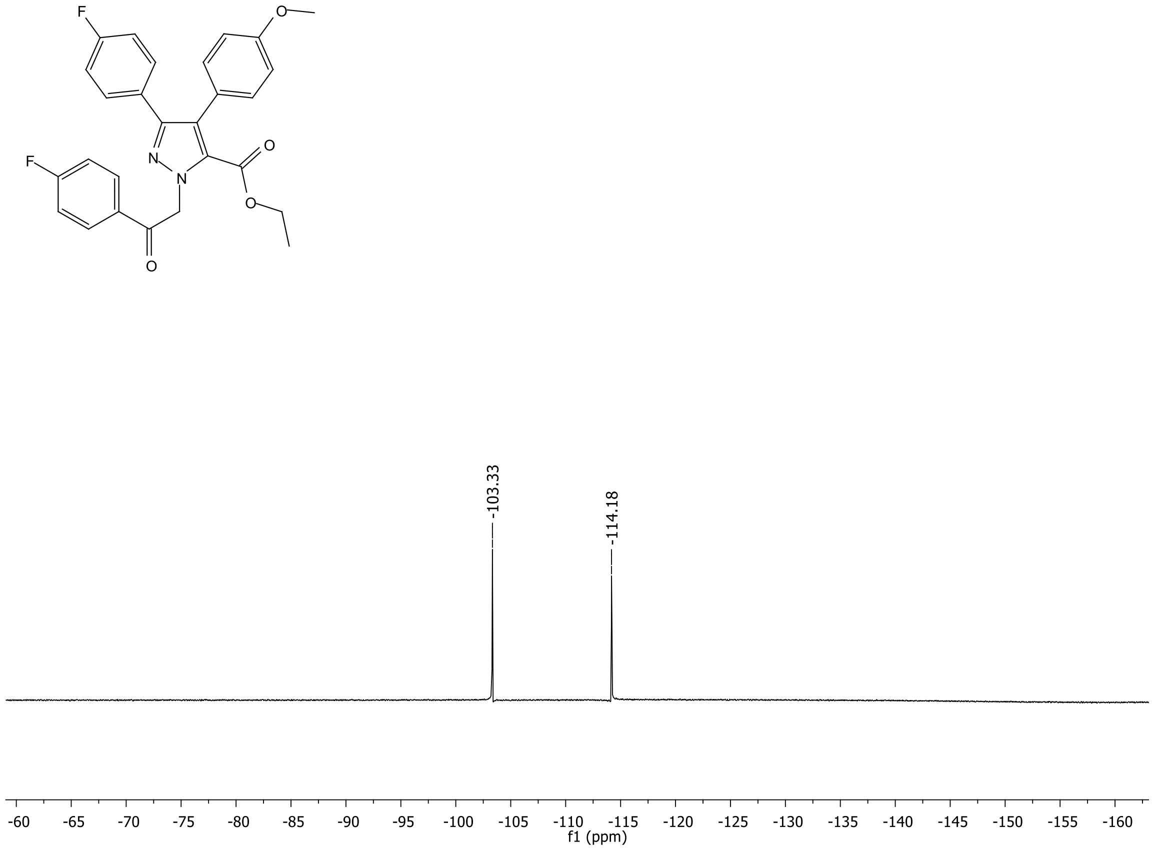

Figure S114. ^{19}F NMR spectrum (376 MHz, $CDCl_3$) of ethyl 3-(4-fluorophenyl)-1-[2-(4-fluorophenyl)-2-oxoethyl]-4-(4-methoxyphenyl)-1*H*-pyrazole-5-carboxylate (**6n**).

Compound Spectrum SmartFormula Report

Analysis Info

Analysis Name	D:\Data\KDD-385.d	Acquisition Date	7/4/2022 4:56:46 PM
Method	DirectInfusion_TuneLow_pos.m	Operator	hplc
Sample Name	KDD-385	Instrument	micrOTOF-Q III 8228888.20448
Comment	AB		

Acquisition Parameter

Source Type	ESI	Ion Polarity	Positive	Set Nebulizer	0.4 Bar
Focus	Not active	Set Capillary	4500 V	Set Dry Heater	180 °C
Scan Begin	50 m/z	Set End Plate Offset	-500 V	Set Dry Gas	4.0 l/min
Scan End	1000 m/z	Set Collision Cell RF	140.0 Vpp	Set Divert Valve	Waste

#	RT [min]	Area	Int. Type	I	S/N	Chromatogram	Max. m/z	FWHM [min]
n.a.	4.6	n.a.	Single spectrum	n.a.	n.a.	n.a.	499.1439	n.a.

+MS, 4.6min #273

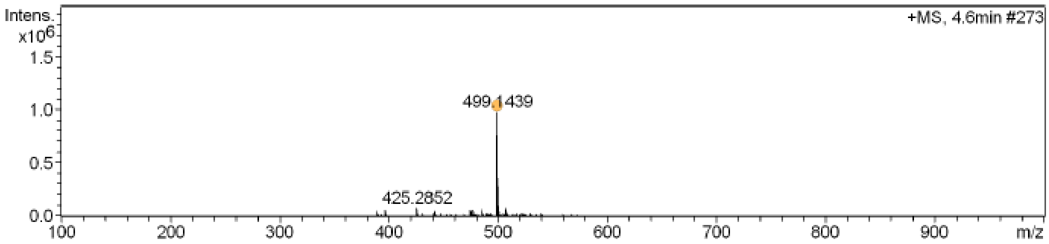

Meas. m/z	#	Ion Formula	m/z	err [ppm]	mSigma	# Sigma	Score	rdb	e⁻ Conf	N-Rule
499.1439	1	C27H22F2N2NaO4	499.1440	0.1	35.5	6	100.00	16.5	even	ok

Figure S115. HRMS (ESI-TOF) spectrum of ethyl 3-(4-fluorophenyl)-1-[2-(4-fluorophenyl)-2-oxoethyl]-4-(4-methoxyphenyl)-1*H*-pyrazole-5-carboxylate (**6n**).

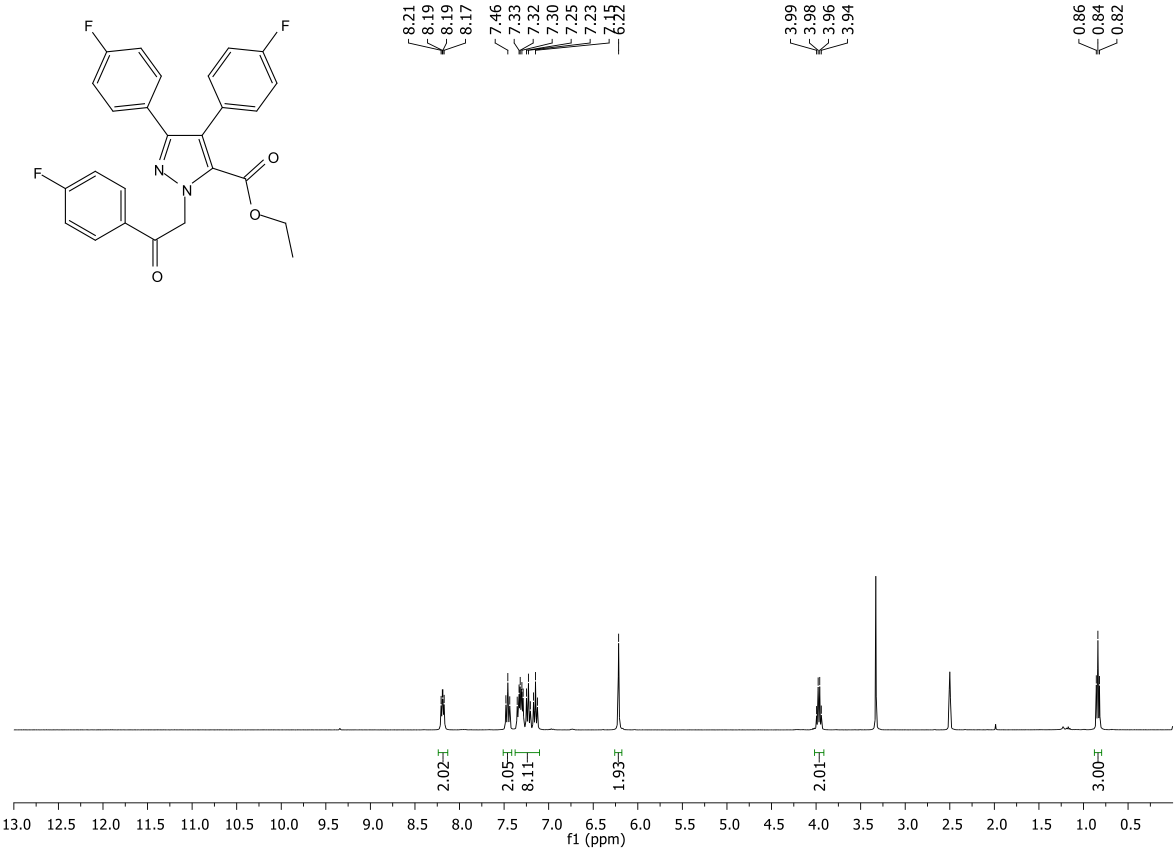

Figure S116. ^{1}H NMR spectrum (400 MHz, DMSO-d_6) of ethyl 3,4-bis(4-fluorophenyl)-1-[2-(4-fluorophenyl)-2-oxoethyl]-1*H*-pyrazole-5-carboxylate (**6o**).

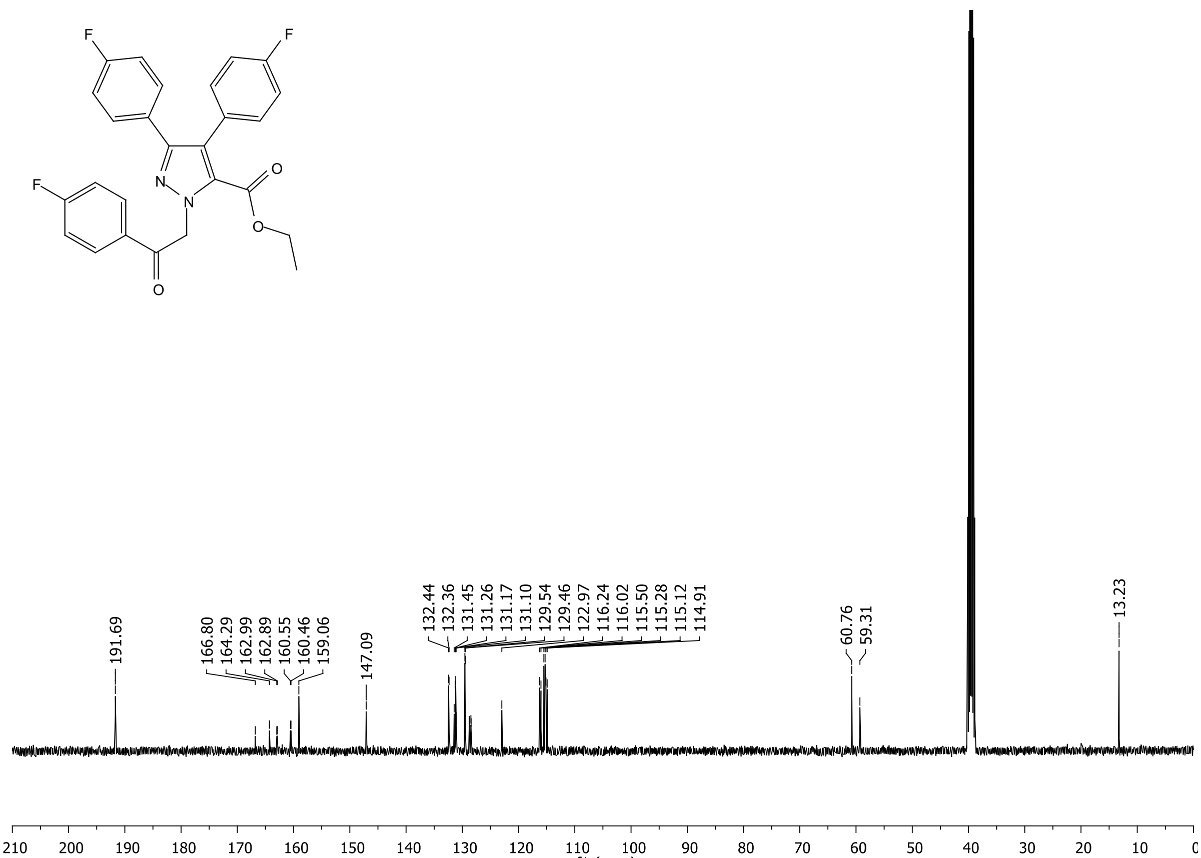

Figure S117. ^{13}C NMR spectrum (101 MHz, DMSO-d_6) of ethyl 3,4-bis(4-fluorophenyl)-1-[2-(4-fluorophenyl)-2-oxoethyl]-1*H*-pyrazole-5-carboxylate (**6o**).

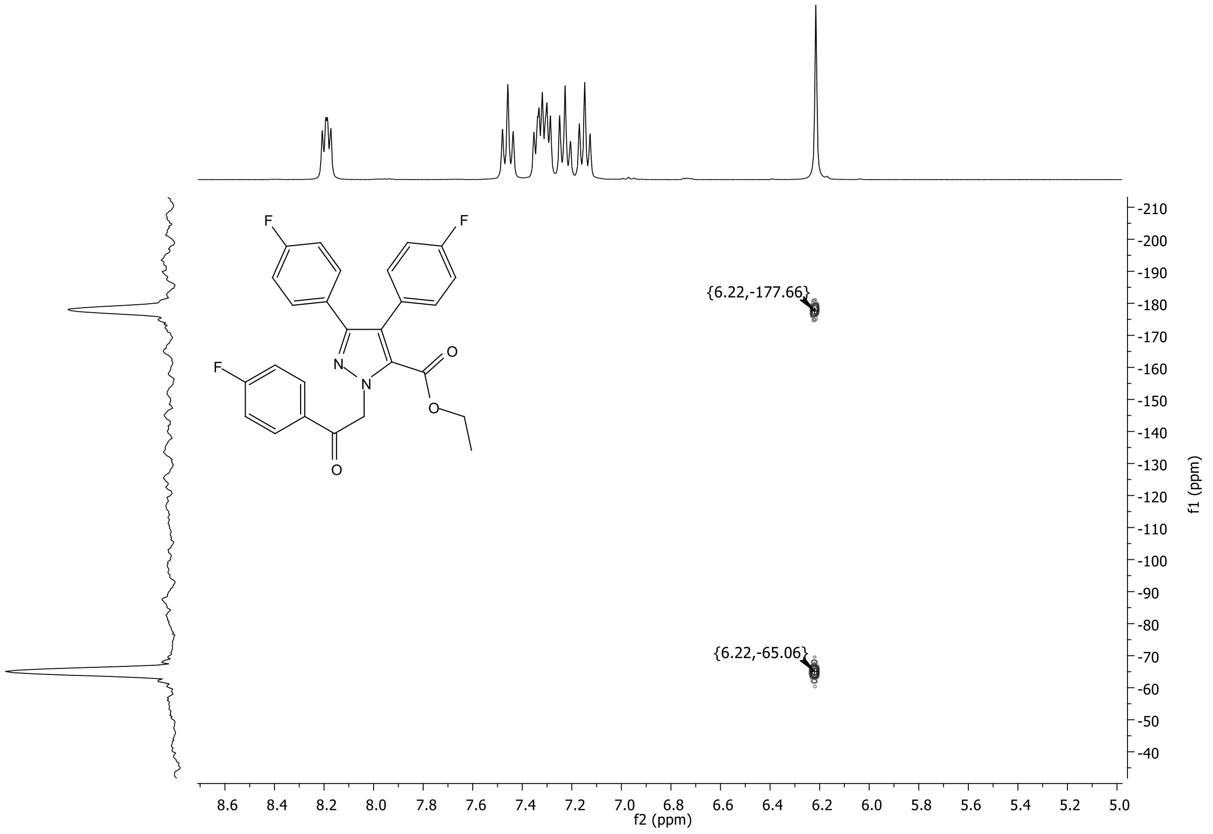

Figure S118. ^{15}N NMR spectrum (40 MHz, DMSO-d_6) of ethyl 3,4-bis(4-fluorophenyl)-1-[2-(4-fluorophenyl)-2-oxoethyl]-1*H*-pyrazole-5-carboxylate (**6o**).

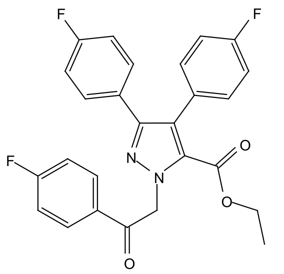

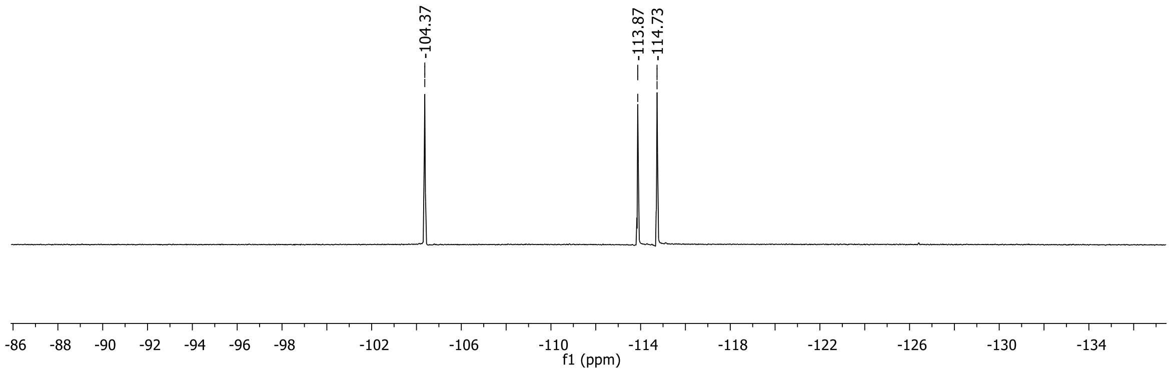

Figure S119. ^{19}F NMR spectrum (376 MHz, DMSO-d_6) of ethyl 3,4-bis(4-fluorophenyl)-1-[2-(4-fluorophenyl)-2-oxoethyl]-1*H*-pyrazole-5-carboxylate (**6o**).

Compound Spectrum SmartFormula Report

Analysis Info

Analysis Name	D:\Data\KDD-383.d	Acquisition Date	7/29/2022 7:43:24 PM
Method	DirectInfusion_TuneLow_pos.m	Operator	hplc
Sample Name	KDD-383	Instrument	micrOTOF-Q III 8228888.20448
Comment	AB		

Acquisition Parameter

Source Type	ESI	Ion Polarity	Positive	Set Nebulizer	0.4 Bar
Focus	Not active	Set Capillary	4500 V	Set Dry Heater	180 °C
Scan Begin	50 m/z	Set End Plate Offset	-500 V	Set Dry Gas	4.0 l/min
Scan End	1000 m/z	Set Collision Cell RF	140.0 Vpp	Set Divert Valve	Waste

#	RT [min]	Area	Int. Type	I	S/N	Chromatogram	Max. m/z	FWHM [min]
n.a.	4.0	n.a.	Single spectrum	n.a.	n.a.	n.a.	487.1238	n.a.

+MS, 4.0min #239

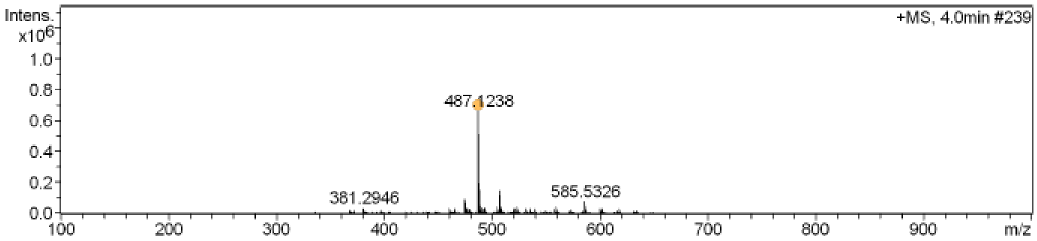

Meas. m/z	#	Ion Formula	m/z	err [ppm]	mSigma	# Sigma	Score	rdb	e⁻ Conf	N-Rule
487.1238	1	C26H19F3N2NaO3	487.1240	0.4	4.1	2	94.98	16.5	even	ok

Figure S120. HRMS (ESI-TOF) spectrum of ethyl 3,4-bis(4-fluorophenyl)-1-[2-(4-fluorophenyl)-2-oxoethyl]-1*H*-pyrazole-5-carboxylate (**6o**).

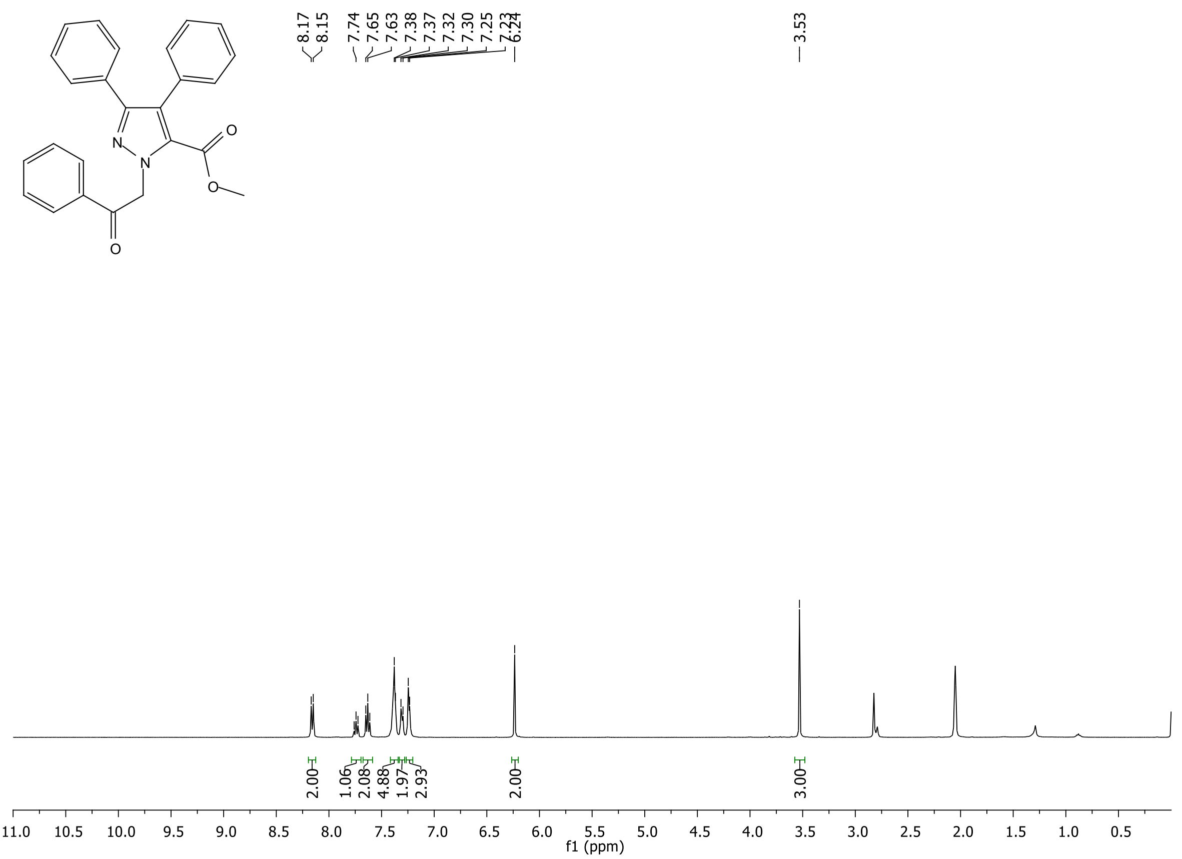

Figure S121. ^{1}H NMR spectrum (400 MHz, acetone-d_6) of methyl 1-(2-oxo-2-phenylethyl)-3,4-diphenyl-1*H*-pyrazole-5-carboxylate (**7a**).

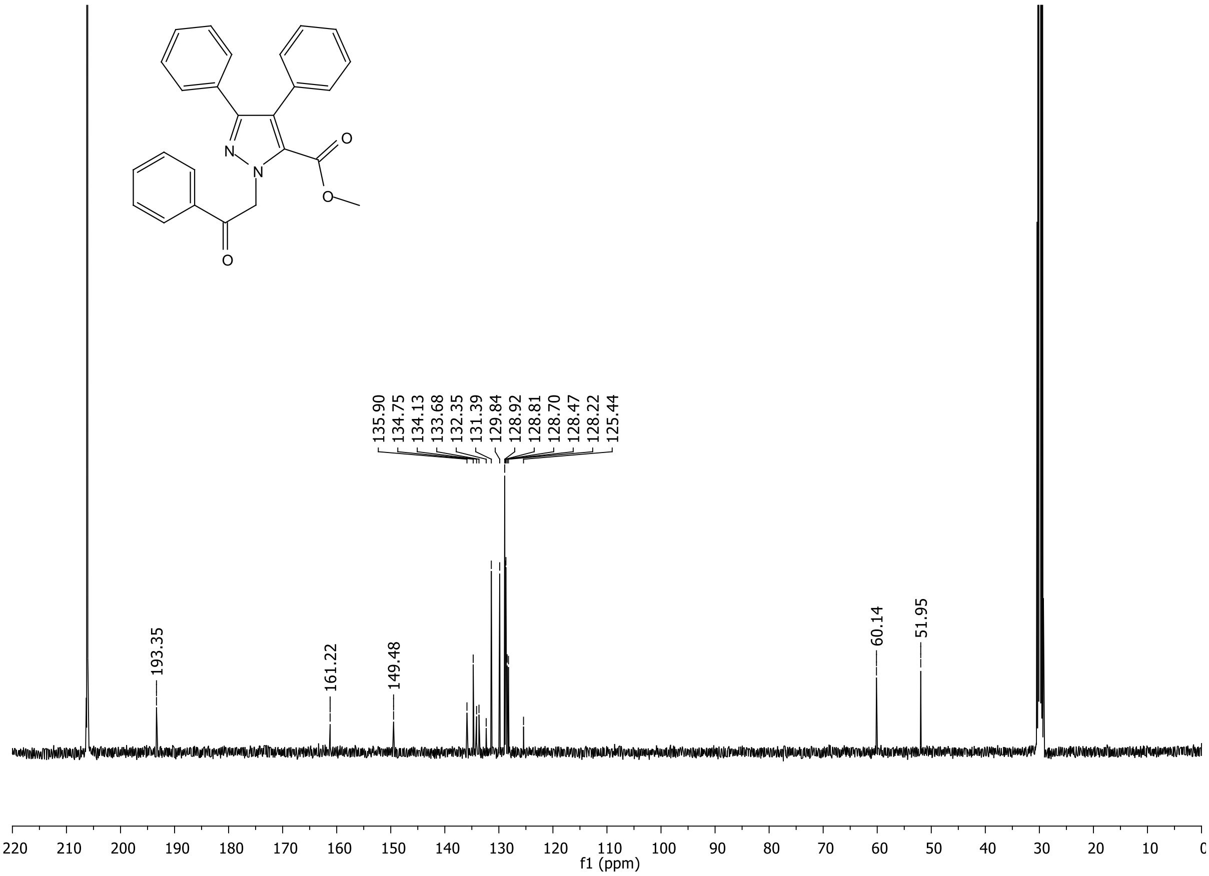

Figure S122. ^{13}C NMR spectrum (101 MHz, acetone-d_6 of methyl 1-(2-oxo-2-phenylethyl)-3,4-diphenyl-1*H*-pyrazole-5-carboxylate (**7a**).

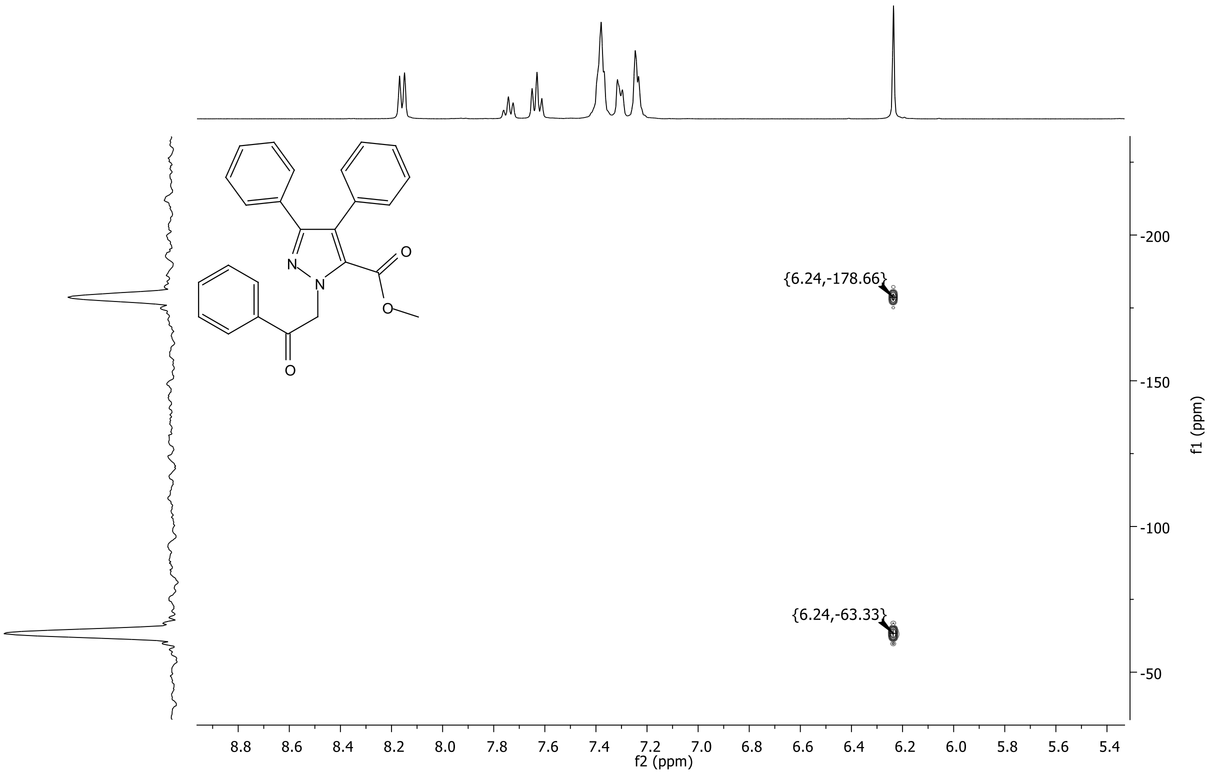

Figure S123. ^{15}N NMR spectrum (40 MHz, acetone-d_6) of methyl 1-(2-oxo-2-phenylethyl)-3,4-diphenyl-1*H*-pyrazole-5-carboxylate (**7a**).

Compound Spectrum SmartFormula Report

Analysis Info

Analysis Name	D:\Data\KDD-362.d	Acquisition Date	7/5/2022 6:11:31 PM
Method	DirectInfusion_TuneLow_pos.m	Operator	hplc
Sample Name	KDD-362	Instrument	micrOTOF-Q III 8228888.20448
Comment	AB		

Acquisition Parameter

Source Type	ESI	Ion Polarity	Positive	Set Nebulizer	0.4 Bar
Focus	Not active	Set Capillary	4500 V	Set Dry Heater	180 °C
Scan Begin	50 m/z	Set End Plate Offset	-500 V	Set Dry Gas	4.0 l/min
Scan End	1000 m/z	Set Collision Cell RF	140.0 Vpp	Set Divert Valve	Waste

#	RT [min]	Area	Int. Type	I	S/N	Chromatogram	Max. m/z	FWHM [min]
n.a.	1.6	n.a.	Single spectrum	n.a.	n.a.	n.a.	419.1367	n.a.

+MS, 1.6min #96

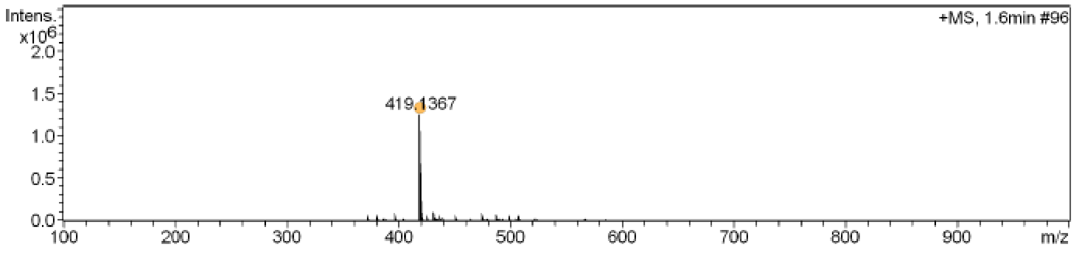

Meas. m/z	#	Ion Formula	m/z	err [ppm]	mSigma	# Sigma	Score	rdb	e⁻ Conf	N-Rule
419.1367	1	C25H20N2NaO3	419.1366	-0.1	132.9	2	100.00	16.5	even	ok

Figure S124. HRMS (ESI-TOF) spectrum of methyl 1-(2-oxo-2-phenylethyl)-3,4-diphenyl-1*H*-pyrazole-5-carboxylate (**7a**).

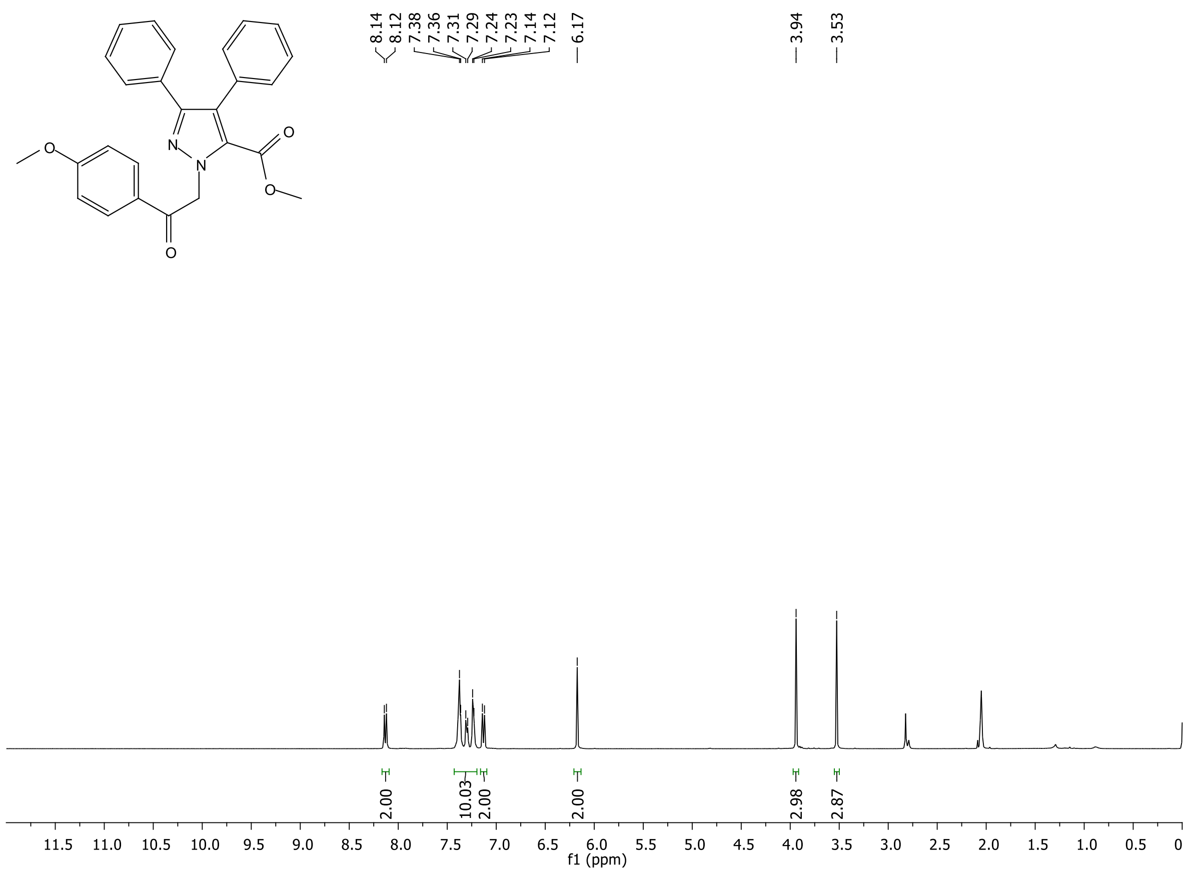

Figure S125. ^{1}H NMR spectrum (400 MHz, acetone-d_6) of methyl 1-[2-(4-methoxyphenyl)-2-oxoethyl]-3,4-diphenyl-1*H*-pyrazole-5-carboxylate (**7b**).

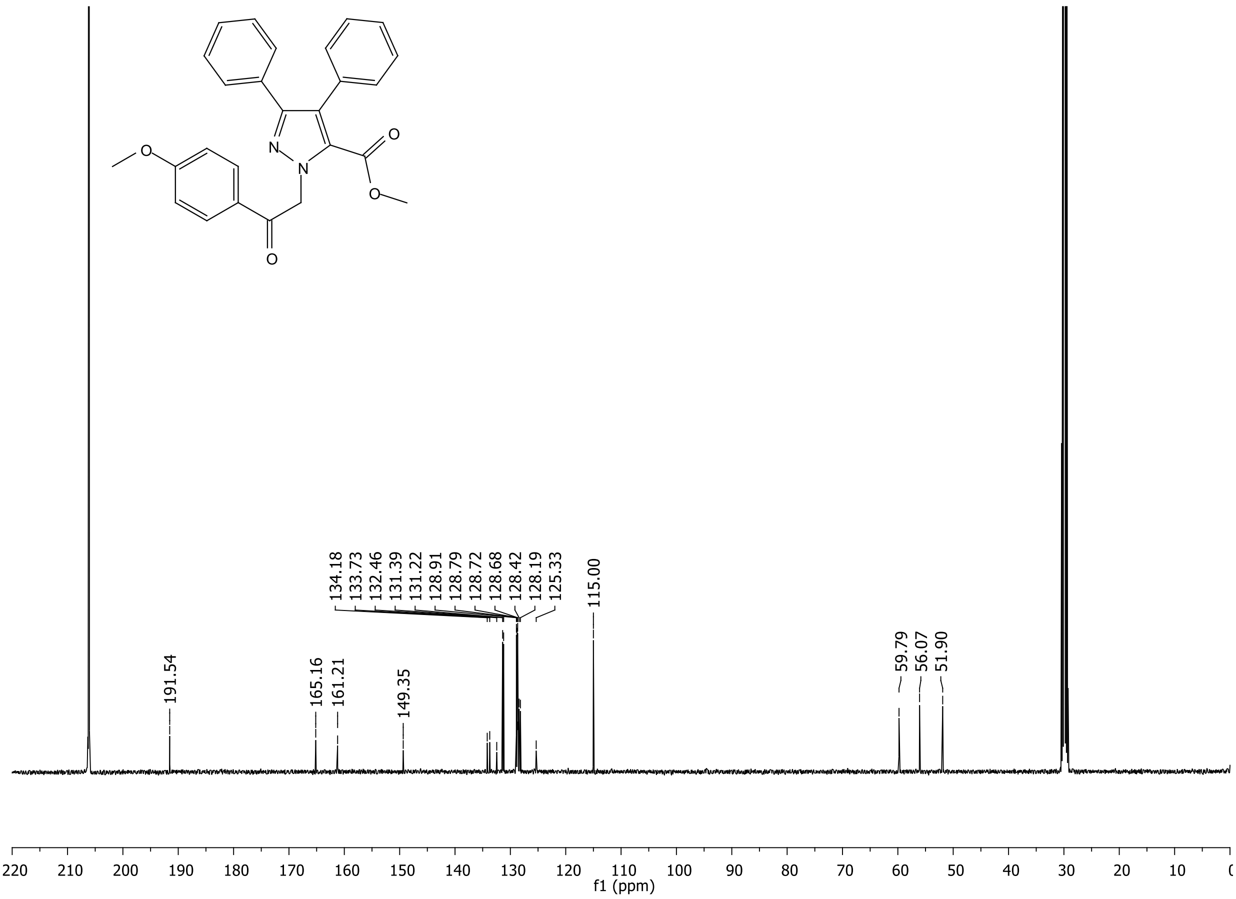

Figure S126. ^{13}C NMR spectrum (101 MHz, acetone-d_6) of methyl 1-[2-(4-methoxyphenyl)-2-oxoethyl]-3,4-diphenyl-1*H*-pyrazole-5-carboxylate (**7b**).

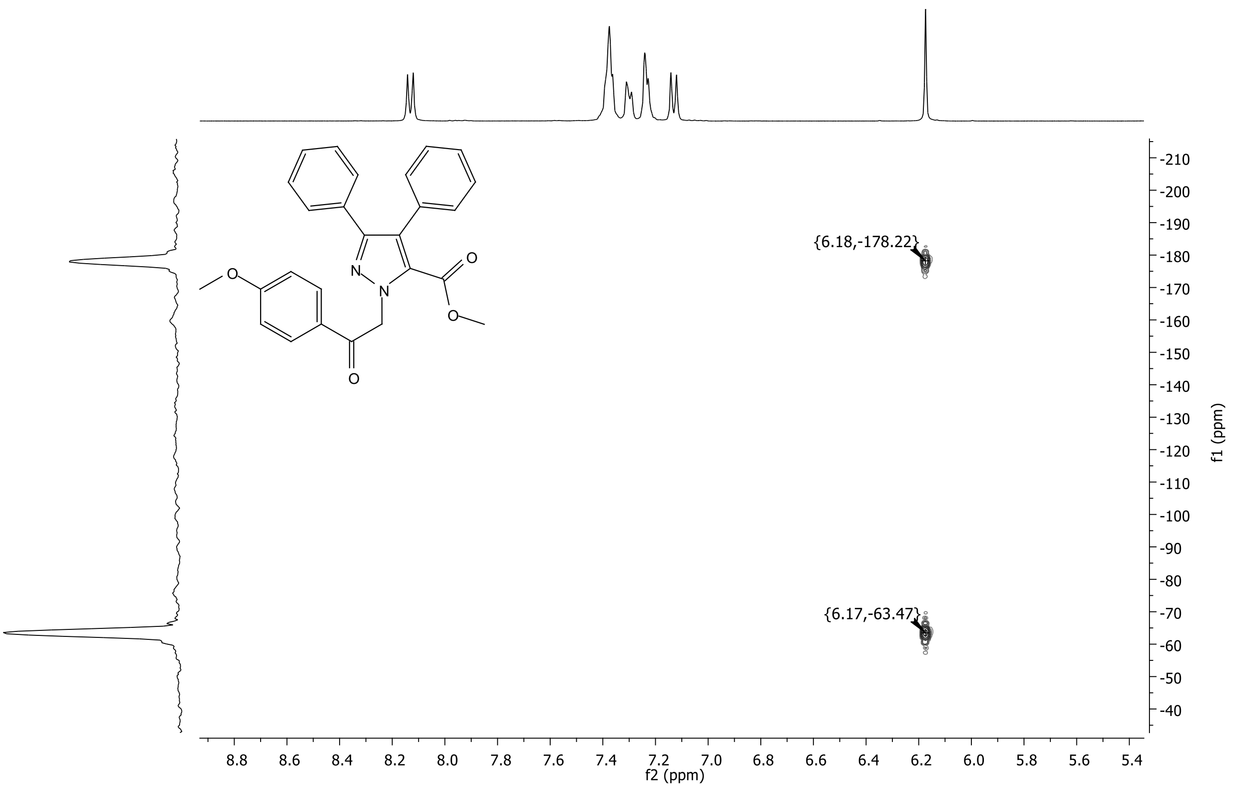

Figure S127. ^{15}N NMR spectrum (40 MHz, acetone-d_6) of methyl 1-[2-(4-methoxyphenyl)-2-oxoethyl]-3,4-diphenyl-1*H*-pyrazole-5-carboxylate (**7b**).

Compound Spectrum SmartFormula Report

Analysis Info

Analysis Name	D:\Data\KDD-366.d	Acquisition Date	7/29/2022 7:00:29 PM
Method	DirectInfusion_TuneLow_pos.m	Operator	hplc
Sample Name	KDD-366	Instrument	micrOTOF-Q III 8228888.20448
Comment	AB		

Acquisition Parameter

Source Type	ESI	Ion Polarity	Positive	Set Nebulizer	0.4 Bar
Focus	Not active	Set Capillary	4500 V	Set Dry Heater	180 °C
Scan Begin	50 m/z	Set End Plate Offset	-500 V	Set Dry Gas	4.0 l/min
Scan End	1000 m/z	Set Collision Cell RF	140.0 Vpp	Set Divert Valve	Waste

#	RT [min]	Area	Int. Type	I	S/N	Chromatogram	Max. m/z	FWHM [min]
n.a.	4.6	n.a.	Single spectrum	n.a.	n.a.	n.a.	449.1470	n.a.

+MS, 4.6min #277

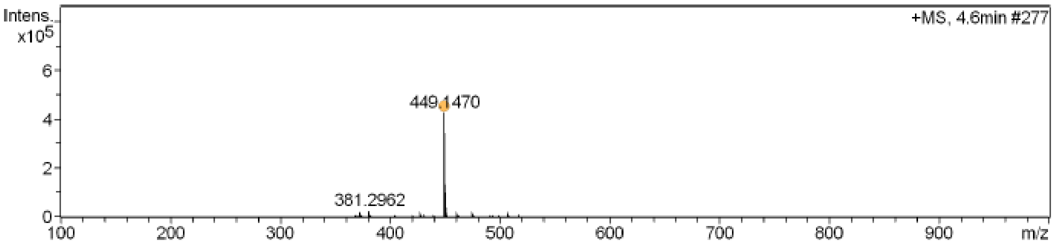

Meas. m/z	#	Ion Formula	m/z	err [ppm]	mSigma	# Sigma	Score	rdb	e⁻ Conf	N-Rule
449.1470	1	C26H22N2NaO4	449.1472	0.5	1.8	1	100.00	16.5	even	ok

Figure S128. HRMS (ESI-TOF) spectrum of methyl 1-[2-(4-methoxyphenyl)-2-oxoethyl]-3,4-diphenyl-1*H*-pyrazole-5-carboxylate (**7b**).

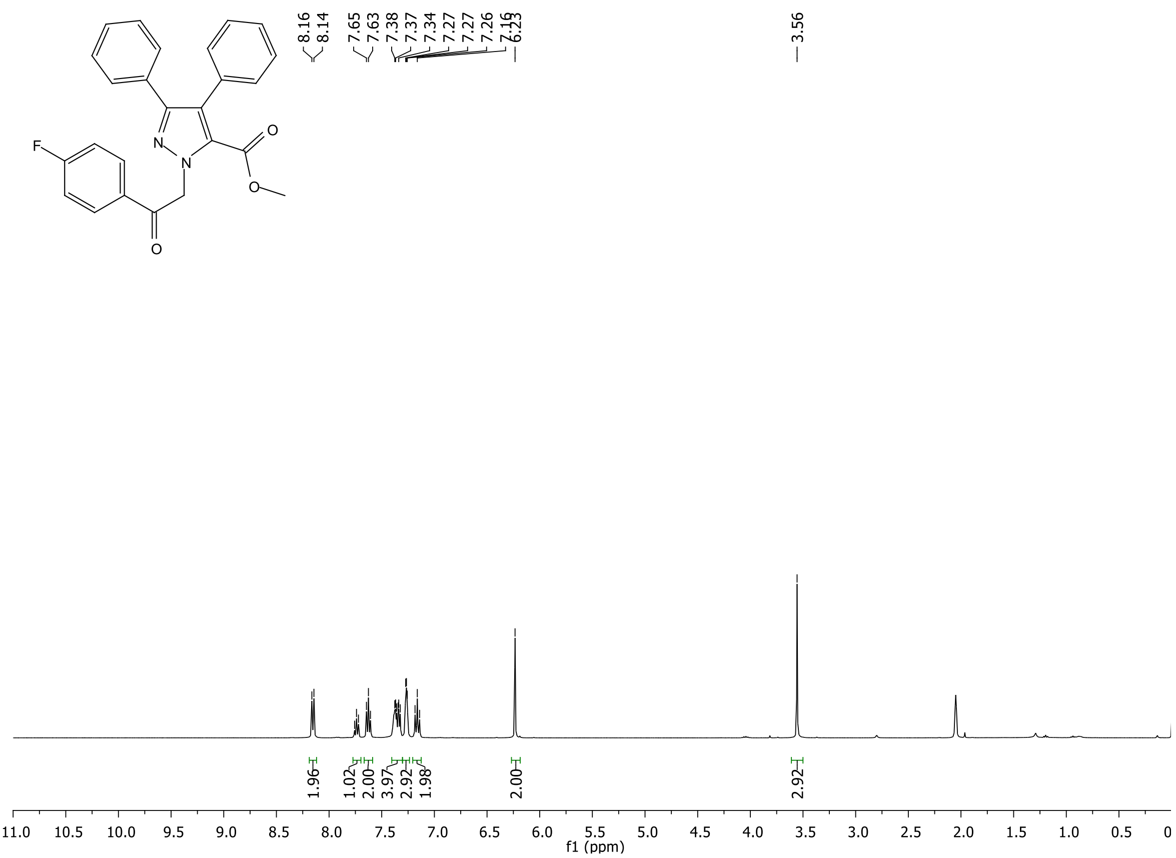

Figure S129. ^{1}H NMR spectrum (400 MHz, acetone-d_6) of methyl 1-[2-(4-fluorophenyl)-2-oxoethyl]-3,4-diphenyl-1*H*-pyrazole-5-carboxylate (**7c**).

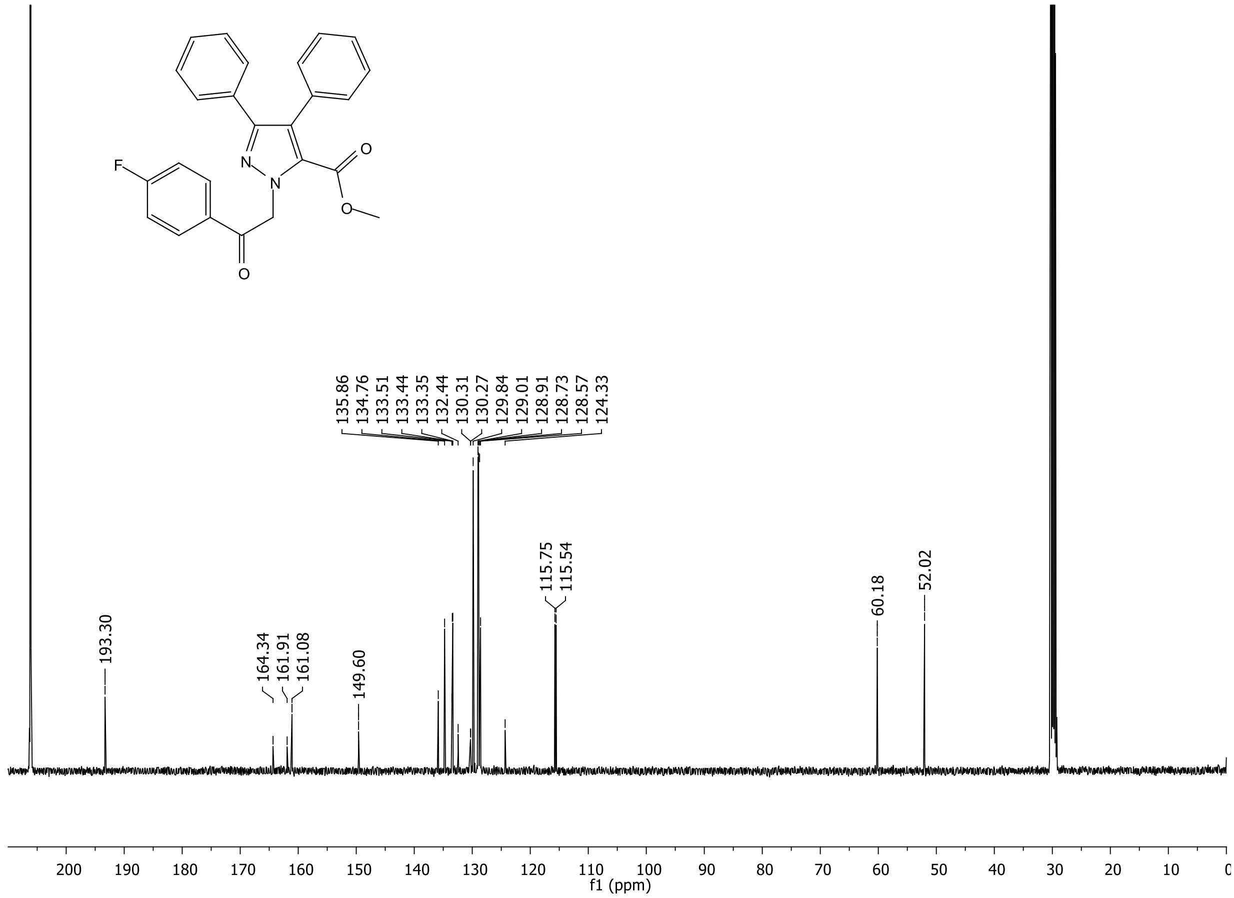

Figure S130. ^{13}C NMR spectrum (101 MHz, acetone-d_6) of methyl 1-[2-(4-fluorophenyl)-2-oxoethyl]-3,4-diphenyl-1*H*-pyrazole-5-carboxylate (**7c**).

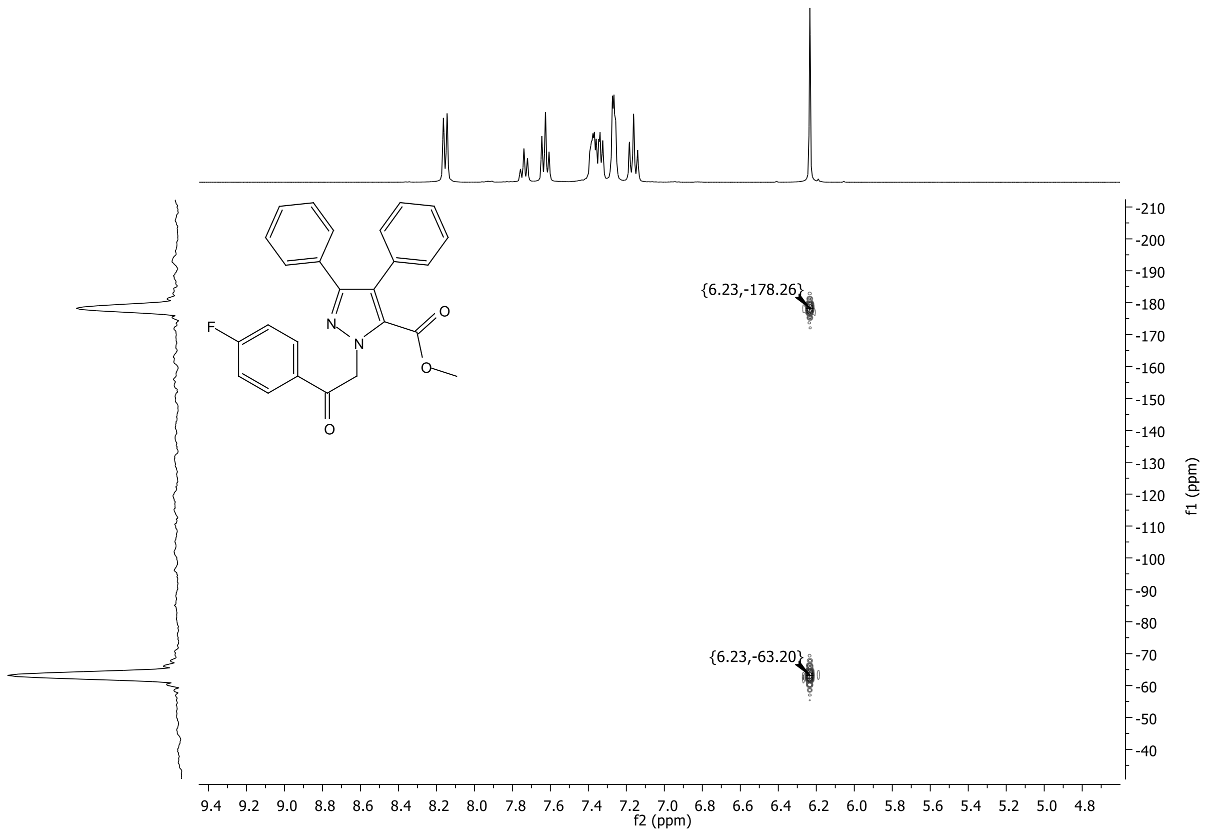

Figure S131. ^{15}N NMR spectrum (40 MHz, acetone-d_6) of methyl 1-[2-(4-fluorophenyl)-2-oxoethyl]-3,4-diphenyl-1*H*-pyrazole-5-carboxylate (**7c**).

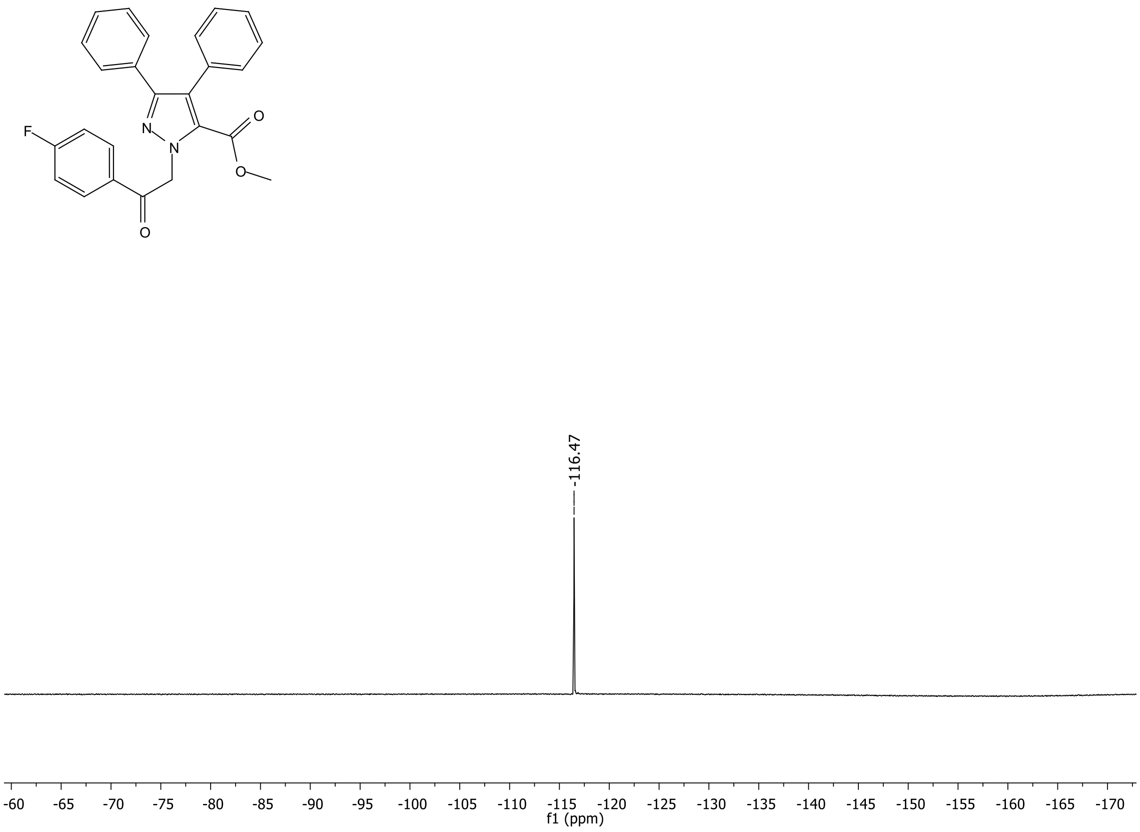

Figure S132. ^{19}F NMR spectrum (376 MHz, acetone-d_6) of methyl 1-[2-(4-fluorophenyl)-2-oxoethyl]-3,4-diphenyl-1*H*-pyrazole-5-carboxylate (**7c**).

Compound Spectrum SmartFormula Report

Analysis Info

Analysis Name	D:\Data\KDD-368.d	Acquisition Date	7/29/2022 7:15:07 PM
Method	DirectInfusion_TuneLow_pos.m	Operator	hplc
Sample Name	KDD-368	Instrument	micrOTOF-Q III 8228888.20448
Comment	AB		

Acquisition Parameter

Source Type	ESI	Ion Polarity	Positive	Set Nebulizer	0.4 Bar
Focus	Not active	Set Capillary	4500 V	Set Dry Heater	180 °C
Scan Begin	50 m/z	Set End Plate Offset	-500 V	Set Dry Gas	4.0 l/min
Scan End	1000 m/z	Set Collision Cell RF	140.0 Vpp	Set Divert Valve	Waste

#	RT [min]	Area	Int. Type	I	S/N	Chromatogram	Max. m/z	FWHM [min]
n.a.	3.5	n.a.	Single spectrum	n.a.	n.a.	n.a.	437.1269	n.a.

+MS, 3.5min #212

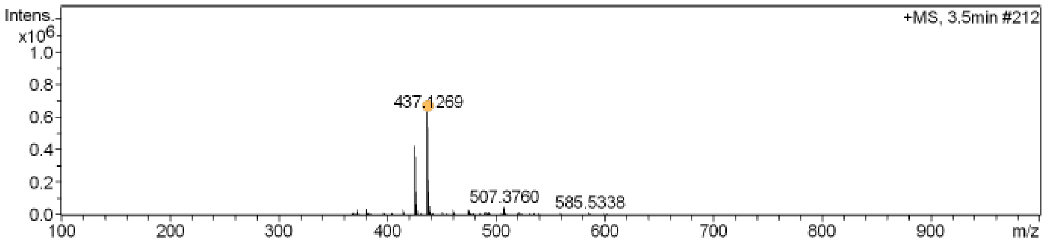

Meas. m/z	#	Ion Formula	m/z	err [ppm]	mSigma	# Sigma	Score	rdb	e⁻ Conf	N-Rule
437.1269	1	C25H19FN2NaO3	437.1272	-0.7	3.3	1	100.00	16.5	even	ok

Figure S133. HRMS (ESI-TOF) spectrum of methyl 1-[2-(4-fluorophenyl)-2-oxoethyl]-3,4-diphenyl-1*H*-pyrazole-5-carboxylate (**7c**).

4. Physicochemical parameters

Table S2. Calculated physicochemical parameters of natural lamellarins and synthesized compounds **6a–o**, **7a–c**. HBD – hydrogen bond donors, HBA – hydrogen bond acceptors, tPSA – topological polar surface area.

Compound	Lipinski's 5	HBD	HBA	tPSA [A]	strongest acidic pK_a	stongest basic pK_a
**lamellarin I**	x	1	8	106.84	9.16	
**lamellarin D**	∨	3	7	119.09	8.78	
**lamellarin O**	∨	2	5	97.99	9.33	
**lukianol A**	∨	3	4	91.92	8.51	
**6a**	x	0	3	61.19	14.65	0.27
**6b**	x	0	4	70.42	14.83	0.27
**6c**	x	0	3	61.19	14.7	0.27
**6d**	x	0	3	61.19	14.61	0.27
**6e**	x	1	4	81.42	7.71	0.27
**6f**	x	0	5	79.65	14.66	0.32
**6g**	x	0	6	88.88	14.84	0.32
**6h**	x	0	5	79.65	14.71	0.32
**6i**	x	0	5	79.65	14.62	0.32
**6j**	x	0	3	61.19	14.65	0.29
**6k**	x	0	3	61.19	14.66	0.29
**6l**	x	0	4	70.42	14.66	0.30
**6m**	x	0	3	61.19	14.70	0.29
**6n**	x	0	4	70.42	14.71	0.30
**6o**	x	0	3	61.19	14.71	0.29
**7a**	x	0	3	61.19	14.65	0.27
**7b**	x	0	4	70.42	14.83	0.27
**7c**	x	0	3	61.19	14.70	0.27

∨ = in agreement; x = not in agreement

5. Biological evaluation

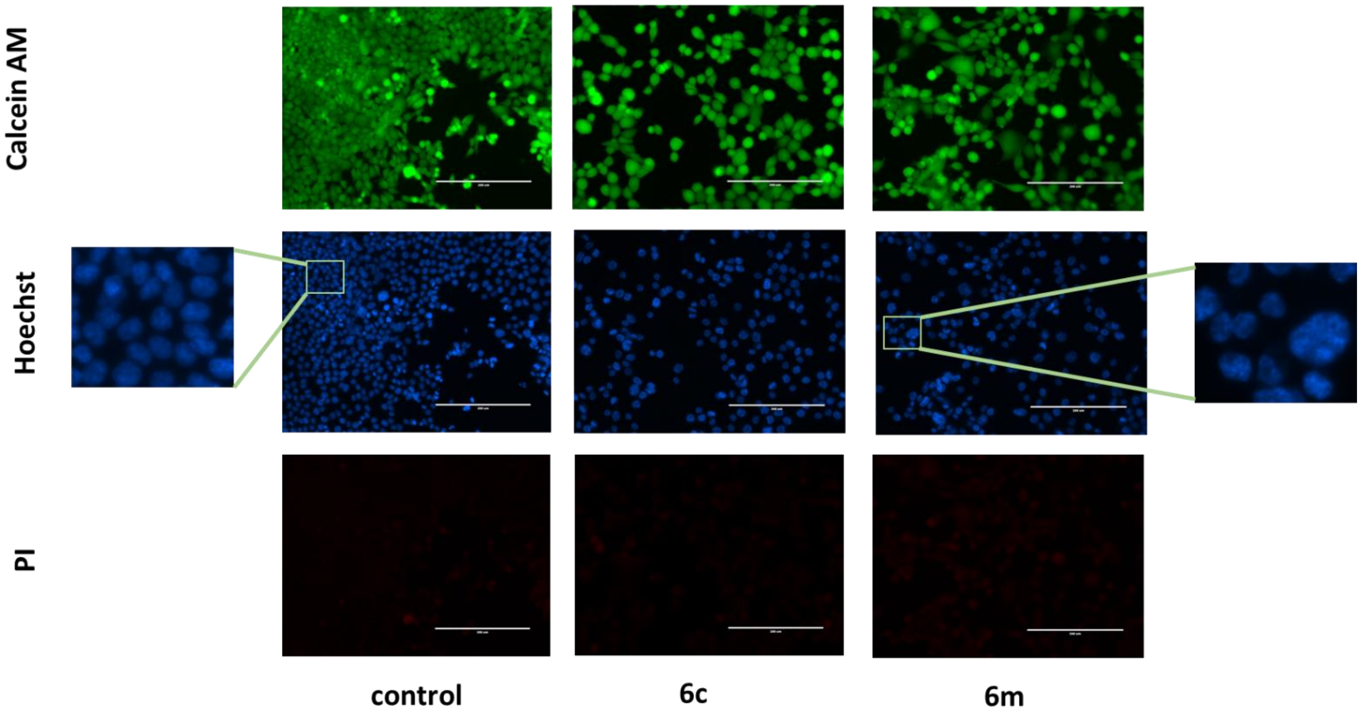

Figure S134. Calcein AM/Hoechst/PI assay results of HCT116 cells treated with compounds **6c** and **6m** (20 µM) for 24 h. Calcein AM staining confirmed differences in the morphology of HCT116. The cells increased in size with reduced cell count due to the inhibition of cell proliferation. Hoechst 33342 staining revealed a fragmented staining for the cells increased in size. The absence of PI staining indicated no involvement of necrosis in the mode of action of the compounds.

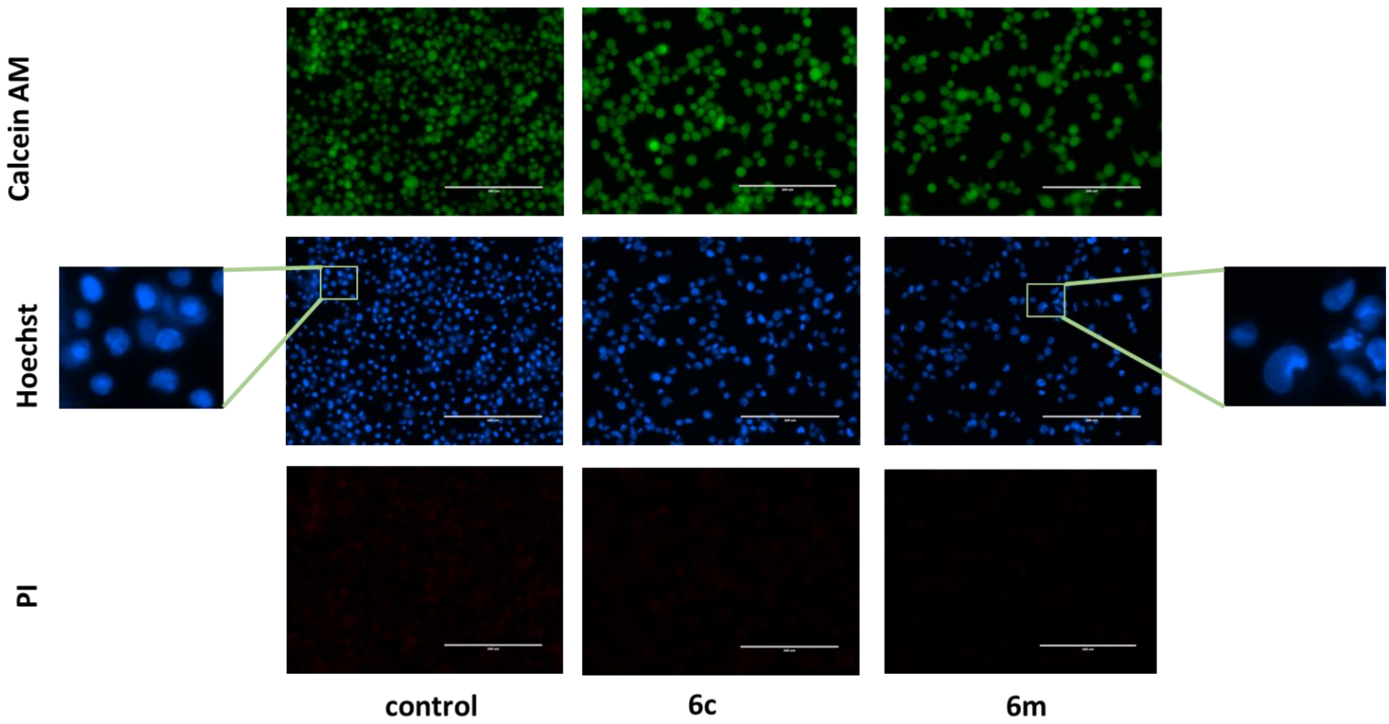

Figure S135. Calcein AM/Hoechst/PI assay results of SW480 cells treated with compounds **6c** and **6m** (20 µM) for 24 h. Calcein AM staining confirmed similar but less pronounced increase in cell size for SW480 cells. Hoechst 33342 staining revealed again a fragmented staining for the cells increased in size, which was more distinct in SW480 than in HCT116 cells. The absence of PI staining indicated no involvement of necrosis in the mode of action of the compounds.

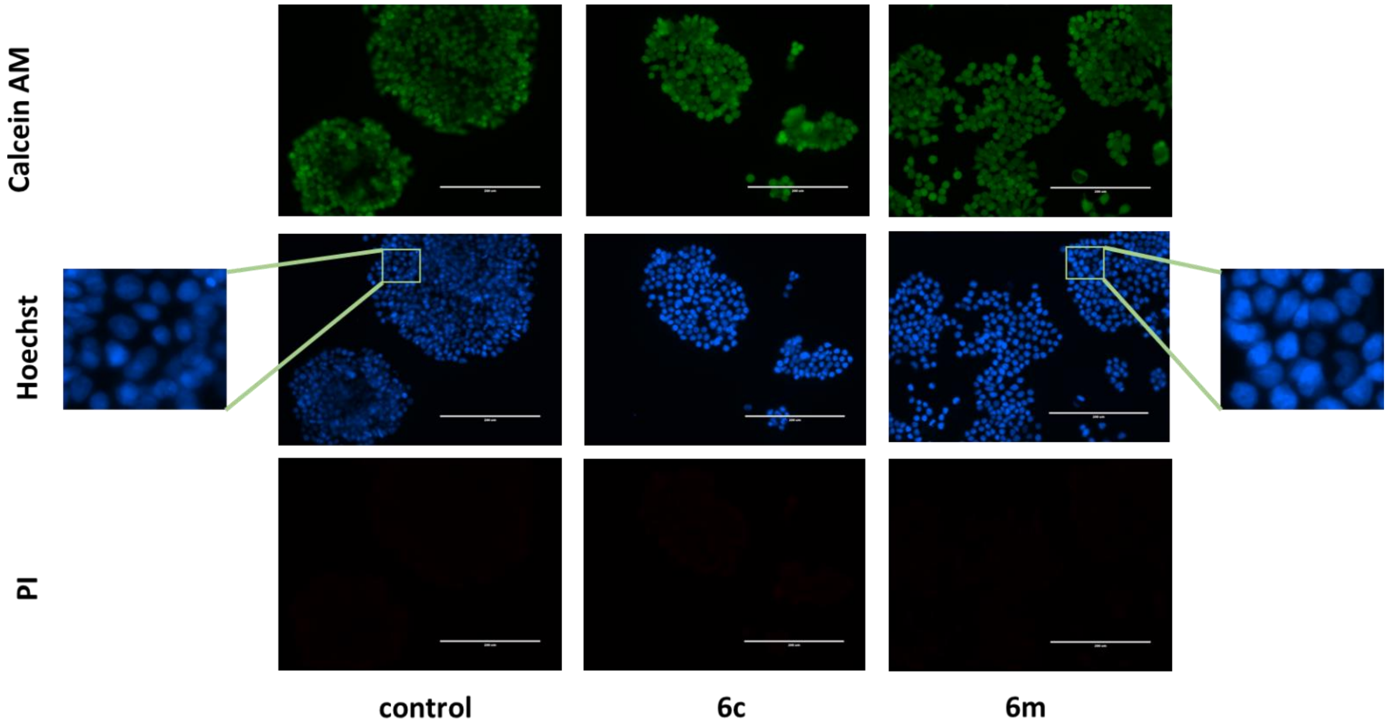

Figure S136. Calcein AM/Hoechst/PI assay results of HT29 cells treated with compounds **6c** and **6m** (20 µM) for 24 h. Calcein AM staining only minor differences in the morphology. The cells increased only minor in size with reduced cell count due to the inhibition of cell proliferation for **6c**. Hoechst 33342 staining revealed an increase in intensity of the staining. The absence of PI staining indicated no involvement of necrosis in the mode of action of the compounds.

Table S3. Correlation between PPB and logP values of selected compounds.

Compound	%PPB ± SD	HPLC-logP	clogP*
**6a**	95.4 ± 0.3	4.381 ± 0.002	5.28
**6c**	96.0 ± 0.3	4.453 ± 0.005	5.44
**6f**	93.9 ± 0.1	4.462 ± 0.005	5.02
**6h**	93.8 ± 0.2	4.531 ± 0.006	5.18
**6m**	96.6 ± 0.1	4.545 ± 0.006	5.59
**6n**	95.3 ± 0.3	4.564 ± 0.004	5.47
**6o**	97.1 ± 0.1	4.598 ± 0.004	5.75

*Calculated using ChemDraw 13.0.
